# Overexpression of 18S rRNA methyltransferase CrBUD23 enhances biomass and lutein content in *Chlamydomonas reinhardtii*

**DOI:** 10.3389/fbioe.2023.1102098

**Published:** 2023-02-03

**Authors:** Chenglong Liu, Haoze Guo, Xinmei Zhao, Bingxi Zou, Ting Sun, Jinwei Feng, Zhiyong Zeng, Xueer Wen, Jun Chen, Zhangli Hu, Sulin Lou, Hui Li

**Affiliations:** ^1^ Guangdong Engineering Research Center for Marine Algal Biotechnology, Guangdong Provincial Key Laboratory for Plant Epigenetics, Shenzhen Key Laboratory of Marine Bioresource and Eco-Environmental Science, Longhua Innovation Institute for Biotechnology, College of Life Sciences and Oceanography, Shenzhen University, Shenzhen, China; ^2^ College of Optoelectronic Engineering, Shenzhen University, Shenzhen, China

**Keywords:** overexpression, 18S rRNA methyltransferase, CrBUD23, CrTrm112, biomass, lutein, *Chlamydomonas reinhardtii*

## Abstract

Post-transcriptional modification of nucleic acids including transfer RNA (tRNA), ribosomal RNA (rRNA) and messenger RNA (mRNA) is vital for fine-tunning of mRNA translation. Methylation is one of the most widespread post-transcriptional modifications in both eukaryotes and prokaryotes. HsWBSCR22 and ScBUD23 encodes a 18S rRNA methyltransferase that positively regulates cell growth by mediating ribosome maturation in human and yeast, respectively. However, presence and function of 18S rRNA methyltransferase in green algae are still elusive. Here, through bioinformatic analysis, we identified CrBUD23 as the human WBSCR22 homolog in genome of the green algae model organism *Chlamydonomas reinhardtii*. CrBUD23 was a conserved putative 18S rRNA methyltransferase widely exited in algae, plants, insects and mammalians. Transcription of CrBUD23 was upregulated by high light and down-regulated by low light, indicating its role in photosynthesis and energy metabolism. To characterize its biological function, coding sequence of CrBUD23 fused with a green fluorescence protein (GFP) tag was derived by 35S promoter and stably integrated into Chlamydomonas genome by glass bead-mediated transformation. Compared to *C. reinhardtii* wild type CC-5325, transgenic strains overexpressing CrBUD23 resulted in accelerated cell growth, thereby leading to elevated biomass, dry weight and protein content. Moreover, overexpression of CrBUD23 increased content of photosynthetic pigments but not elicit the activation of antioxidative enzymes, suggesting CrBUD23 favors growth and proliferation in the trade-off with stress responses. Bioinformatic analysis revealed the G1177 was the putative methylation site in 18S rRNA of *C. reinhardtii* CC-849. G1177 was conserved in other Chlamydonomas isolates, indicating the conserved methyltransferase activity of BUD23 proteins. In addition, CrTrm122, the homolog of BUD23 interactor Trm112, was found involved in responses to high light as same as CrBUD23. Taken together, our study revealed that cell growth, protein content and lutein accumulation of Chlamydomonas were positively regulated by the 18S rRNA methyltransferase CrBUD23, which could serve as a promising candidate for microalgae genetic engineering.

## 1 Introduction

Microalgae have all the essential amino acids required by human beings and are rich in various vitamins and minerals (Kumar et al., 2020). As new resource food, the high nutritional value and good biological safety of microalgae products have attracted extensive attention worldwide. Especially, when the health industry has become a global development hotspot, microalgae has become the favorite food and nutrient supply (Ma et al., 2020). Based on the increasing demand, microalgae nutrition products represented by green algae such as *Chlamydomonas reinhardtii*, *Haematococcus pluvialis* and *Dunaliella salina* are more and more popular in the market (Lou et al., 2020; Sun et al., 2020; Tao et al., 2021).

As an important raw material for healthy food, *C*. *reinhardtii* is known as a microalgae contains rich protein, triglycerides, carotenoids and microelement combinations, which are beneficial to human health (Lou et al., 2018). Moreover, *C*. *reinhardtii* could be cultured heterotrophically at large-scale, so it is an optimal chassis algal strain with application prospects for bioproduction of carbon-rich compounds such as starch, oil and protein (Lou et al., 2018). Over the past decades, a large number of researchers have committed themselves to overcoming many scientific and technological difficulties in large-scale cultivation and molecular breeding (Rolland et al., 2009; Merchant et al., 2012; Saroussi et al., 2017; Umen, 2018). Nutrient supplement was revised to increase biomass and growth rate in *C. reinhardtii* (Kropat et al., 2011; Zheng et al., 2022). Light management was another important factor in improvement of cultivation and enhancement of the productivity of valued bioproducts (Kato et al., 2019; Li et al., 2021). It was also found that co-culture with the bacterium *Bradyrhizobium japonicum* or *Methylobacterium oryzae* could increase biomass and hydrogen production (Xu et al., 2016; Torres et al., 2022). Ectopic expression of *Escherichia coli* malate synthase enhanced biomass through upregulation of heterotrophic metabolism (Paik et al., 2019). And 1-Aminocyclopropane-1-carboxylate from *Pseudomonas putida* render Chlamydomonas elevated biomass and lipid content upon nitrogen deficiency (Srinivasan et al., 2021). Especially, the secrets embedded in the genome of *C. reinhardtii* was recovered in 2007 (Merchant et al., 2007). With the rapid development of study in genome, transcriptome and metabolome, functional genetics and molecular breeding help researchers manipulate the growth and metabolism more effectively and more efficiently. Interestingly, optimization of PSII antenna effectively improved photon conversion efficiency and increased growth rates under high light conditions (Beckmann et al., 2009). Besides, CrPDX2, a pyridoxal kinase involved in vitamin B6 biosynthesis and similar to putative ethylene-inducible protein, enhanced biomass and CO_2_ fixation by modulating the activity of carbonic anhydrase and reducing CO_2_ emission (Lin et al., 2021). In addition, knockdown of CrPsbA that is a key component of photosystem II complex through artificial miRNA efficiently increased hydrogen bioproduction (Li et al., 2018). Interestingly, a recent study showed starch synthesis in *C. reinhardtii* could be effectively promoted by perturbation on electron carriers (Zhou et al., 2022). Particularly, in-depth study of the energy metabolism and a comprehensive understanding of cell cycle are helpful for the improvement of the growth rate and the cell density.

Ribosomes are the molecular machines that synthesize proteins and are composed of ribosomal ribonucleic acid (rRNA), a large and a small subunit which carry out the essential functions of polypeptide synthesis and mRNA decoding, respectively (de la Cruz et al., 2015). Ribosome production is tightly linked to cellular growth as cells must produce enough ribosomes to meet their protein needs. The energetic coupling between ribosomes and other cellular compartments, such as chloroplasts, mitochondria and peroxisomes, is increasingly recognized as an important process involved in photosynthetic carbon fixation (Chen et al., 2022; Dahan et al., 2022). Recent studies have shown that the process of ribosomal protein polymerization not only affects the adversity adaptability and survival rate of cells, but also affects the content and composition of biomass (Lafita-Navarro et al., 2018). Therefore, artificial design and experimental optimization of ribosome in *C. reinhardtii* is a promising way to improve the biomass and bioproduction of high-value compounds such as oil, protein, and carotenoid.

Ribosomal RNA (rRNA) is the most abundant RNA in cells. Ribosomal RNA forms the core of ribosomes and provides the environment that enables tRNA to correspond to the codon on mRNA and synthesize proteins (Sharma and Lafontaine, 2015). In *Saccharomyces cerevisiae*, deletion of BUD23 gene caused severely impaired growth, reduced levels of the small (40S) ribosomal subunit (SSU), and blocked the late process in 40S maturation (White et al., 2008). ScBUD23 belongs to the S-adenosylmethionine-dependent Rossmann-fold methyltransferase superfamily and is related to small-molecule methyltransferases (White et al., 2008). In *Arabidopsis thaliana*, the mutant root initiation defective 2 (*rid2*) was defected in formation of adventitious root and accumulation of rRNA precursors (Ohbayashi et al., 2011). The mutant *rid2* could be complemented by the Arabidopsis BUD23 homolog and be suppressed by *sriw1* the mutant carrying a missense mutation site in the NAC transcription factor gene AtANAC082. Cytologically, the *rid2* mutant is characterized by cell proliferation defects accompanied by nucleolar enlargement (Shinohara et al., 2014; Ohbayashi et al., 2017). In *Homo sapiens*, WBSCR22 protein was upregulated in breast cancer and enhanced tumor cell survival in the vasculature (Ounap et al., 2013). Knockdown of WBSCR22 with siRNA resulted in slower growth, abnormal pre-rRNAs processing (Ounap et al., 2013). Although WBSCR22 is localized throughout the cell nucleus and is not stably associated with ribosomal subunits within the cell nucleus, WBSCR22 is involved in ribosome small subunit biosynthesis (Ounap et al., 2015). Together, BUD23 or WBSCR22 was a SSU processome-associated factor which is essential for ribosome biogenesis in yeast, plants and mammalians. However, the effects of BUD23 on growth of microalgae is still unknown.

Here, we identified the putative 18S rRNA methyltransferase CrBUD23 by bioinformatically searching the homolog of human WBSCR22 in genome of *C. reinhardtii*. Transcription analysis showed the CrBUD23 was induced by high light and repressed by low light, suggesting CrBUD23 was involved in energy metabolism. Furthermore, transgenic algae strains overexpressing CrBUD23 displayed increased growth rate, enhanced biomass and dry weight, promoted protein content and chlorophyll content, indicating CrBUD23 positively regulates growth and cell proliferation. Besides, overexpression of CrBUD23 lead to increased lutein accumulation but not activated oxidation. These results demonstrated that CrBUD23 was a positive regulator on growth of *C. reinhardtii* and that overexpressing CrBUD23 was a promising strategy to boost up biomass and lutein bioproduction through microalgae genetic engineering.

## 2 Materials and methods

### 2.1 Strain and cultivation conditions

*Chlamydomonas reinhardtii* wild type strain CC-5325 was obtained from Chlamydomonas Resource Center at University of Minnesota. *C. reinhardtii* CC-5325 and transgenic strains were cultured in TAP (Tris-acetate-phosphate) medium. The growth chambers were maintained at 22 °C and 25 μmol photons/m^2^s under cool-white fluorescent lamps with a 16 hrs-light/8 hrs-dark cycle. The microalgae cultured in regular growth chambers were considered as CK group. The microalgae under high light treatment and low light treatment were cultured in the growth chamber with 75 and 8 μmol photons/m^2^s, respectively. The microalgae under heavy metal treatments were cultured in the TAP medium with 0.01 mM CoCl_2_, NiCl_2_, and CdCl_2_, respectively.

### 2.2 Gene cloning

*Chlamydomonas reinhardtii* BUD23 (Cre09. g398104) sequence was obtained from Phytozome (https://phytozome-next.jgi.doe.gov/). The CDS sequence of CrBUD23 was amplified by reverse transcription PCR using specific primers (Supplementary Table S5). The CrBUD23 PCR-amplified product was cloned into the expression vector pCAMBIA2300-GFP by homozygous recombination using ClonExpress Ultra One Step Cloning Kit (Vazyme). The final construct consisted of the CaMV35S promotor, CrBUD23 fused with a GFP tag, and the NOS terminator along with the kanamycin resistance gene cassette for selection.

### 2.3 Genetic transformation and identification

*C. reinhardtii* CC-5325 served as the receptor strain. Algal cells (2 × 10^6^ cells ml^−1^) were centrifuged and resuspended to a final concentration of 2 × 10^8^ cells ml^−1^. A 300 μL volume of algal cells was transformed with 1 μg plasmid DNA using the glass bead-mediated method (Lou et al., 2021). The cells were placed in 15 mL fresh TAP medium under white light and selected by 200 mg L^−1^ kanamycin in TAP agar medium plates. Genomic DNA was extracted from CC-5325 and transgenic strains using Universal DNA Extraction Kit Ver.3.0 (Takara). Transgenic algal strains screened by kanamycin were verified using genomic DNA PCR analysis. PCR was performed with KOD fx (KFX-101) (TOYOUBO). All of the amplified products were verified by DNA Sanger sequencing (Tsingke Biotechnology).

### 2.4 RNA extraction and qRT-PCR assay

Total RNA was extracted using RNAiso Plus for Total RNA kit and DNA synthesis performed using PrimeScript™ Double-Strand cDNA Synthesis Kit (Takara). To quantitatively detect changes of CrBUD23 and CrTrm112 expression in both wild-type and transgenic strains, qRT-PCR was performed with Bio-Rad CFX Connect Optics Module or ABI QuantStudio 6 Flex, and the primers used are listed in Supplementary Table S5. The standard protocol was applied to CrBUD23 and CrTrm112 expression detection using KOD SYBR qPCR Mix (TOYOBO). PCR conditions were as follows: one step of 95°C for 30 s, followed by 40 cycles of 95°C for 5 s and 60°C for 30 s. The actin (Cre13.g603700) gene was used as the reference gene in qRT-PCR detection of CrBUD23 and CrTrm112. Data were analyzed using the 2^−ΔΔCT^ program. Three technical replicates and three biological replicates were done.

### 2.5 Growth measurement

To measure the cell density, numbers of algal cells were counted by using the hemocytometer (Improved Neubauer, United States). Dry weight of algal cells was measured according to the method described by Wu et al. (Wu et al., 2020a) with the following adjustments. The glass microfiber filters (Whatman GF/C, 47 mm, United Kingdom) were heated (105°C, 24 h) and weighed. After 10–20 mL of algal cells suspension was filtered through the filters, the filters were washed twice each with 20 mL of 0.5 M ammonium bicarbonate. Then the filters were weighed after drying at 105°C for 24 h. Dry weight of algal cells was calculated using the following equations:

$$Dw=\frac{W_{a}-W_{b}}{v}$$

where “Wa” and “Wb” were the weight of the filters at the end and start of cultivation, respectively. And “v” and “Dw” were the volume and the dry weight of the microalgae suspension collected for this measurement, respectively. The chlorophyll of algal cells were extracted by 95% ethanol and then quantified according to Spreitzer’s method, as described by Li et al. (Li et al., 2018). Total protein was measured by Bradford method using Total protein quantitative assay kit (Nanjing Jiancheng Bioengineering Institute). Total glucose was measured by glucose oxidase method using Glucose kit (Nanjing Jiancheng Bioengineering Institute).

### 2.6 Carotenoid quantification

Carotenoid extraction methods were carried out as described by Jiang et al. (Jiang et al., 2004)with the following adjustments. Twelve millilitres of microalgal solution were transferred into a 15 mL centrifuge tube and centrifuged (1200 × g, 4°C) for 10 min. The supernatant was removed and the precipitate was washed twice with distilled water, after which 5 mL of acetone was added. The samples were sonicated for 5 min, and then centrifuged (3200 × g, 4°C) for 3 min. The extract was transferred into another centrifuge tube to which 0.5 mL of 60% (v/v) KOH was added, and after shaking for 3 min, allowed to stand until the layers separated. The supernatant extract was filtered through a 0.22-μm pinhole filter and finally stored at −20°C. According to related previous studies (Wu et al., 2020b), a high-performance liquid chromatography (HPLC) (LC-20A, Shimadzu Corporation) method was used to analyse the β-carotene, lutein and zeaxanthin contents. Acetonitrile: deionized water (95:5, v: v) was used as the first mobile phase to separate lutein, then acetonitrile: methanol: dichloromethane (80:5:23, v: v: v) was used as the second mobile phase quickly to separate the β-carotene. For HPLC, the detection wavelength was 450 nm, the column temperature was 28°C, the injection amount was 50 μL, and the flow rate was 0.8 mL min^−1^. The β-carotene standard was configured as a series of standard solutions of 5, 10, 20, 40, and 80 mgL^−1^, whereas the lutein standard was designed as standard solutions of 6.25, 12.5, 25, 50, and 100 mgL^−1^, whereas the zeaxanthin standard was designed as standard solutions of 6.25, 12.5, 25, 50, and 100 mgL^−1^. Finally, the contents of β-carotene, lutein and zeaxanthin were determined according to the correlation equation between the carotenoid peak area and the standard curves.

### 2.7 Determination of oxidation and antioxidative activities

Catalase (CAT) activity was measured by ammonium molybdate method using Catalase assay kit (Nanjing Jiancheng Bioengineering Institute). Nitric oxide (NO) induction was measured by nitrate reductase method using Nitric Oxide assay kit (Nanjing Jiancheng Bioengineering Institute).

### 2.8 Statistical analysis

Experiments were carried out with biological replicates included from three separate cultures independently, and data were reported as the mean with standard deviation (mean ± SD). For gene expression experiments, quantitative real-time PCR analysis was performed using the BioRAD CFX Maestro 1.1 (Version 4.1.2433.1219). Statistical analyses were performed using the Student’s t-test and Tukey test. For all of the data analysis, a *p*-value <0.01 was considered highly significantly different, a *p*-value < 0.05 represents statistically significant, while *p* > 0.05 means not significant.

## 3 Results and discussion

### 3.1 CrBUD23 is a putative 18S rRNA methyltransferase

**FIGURE 1:**
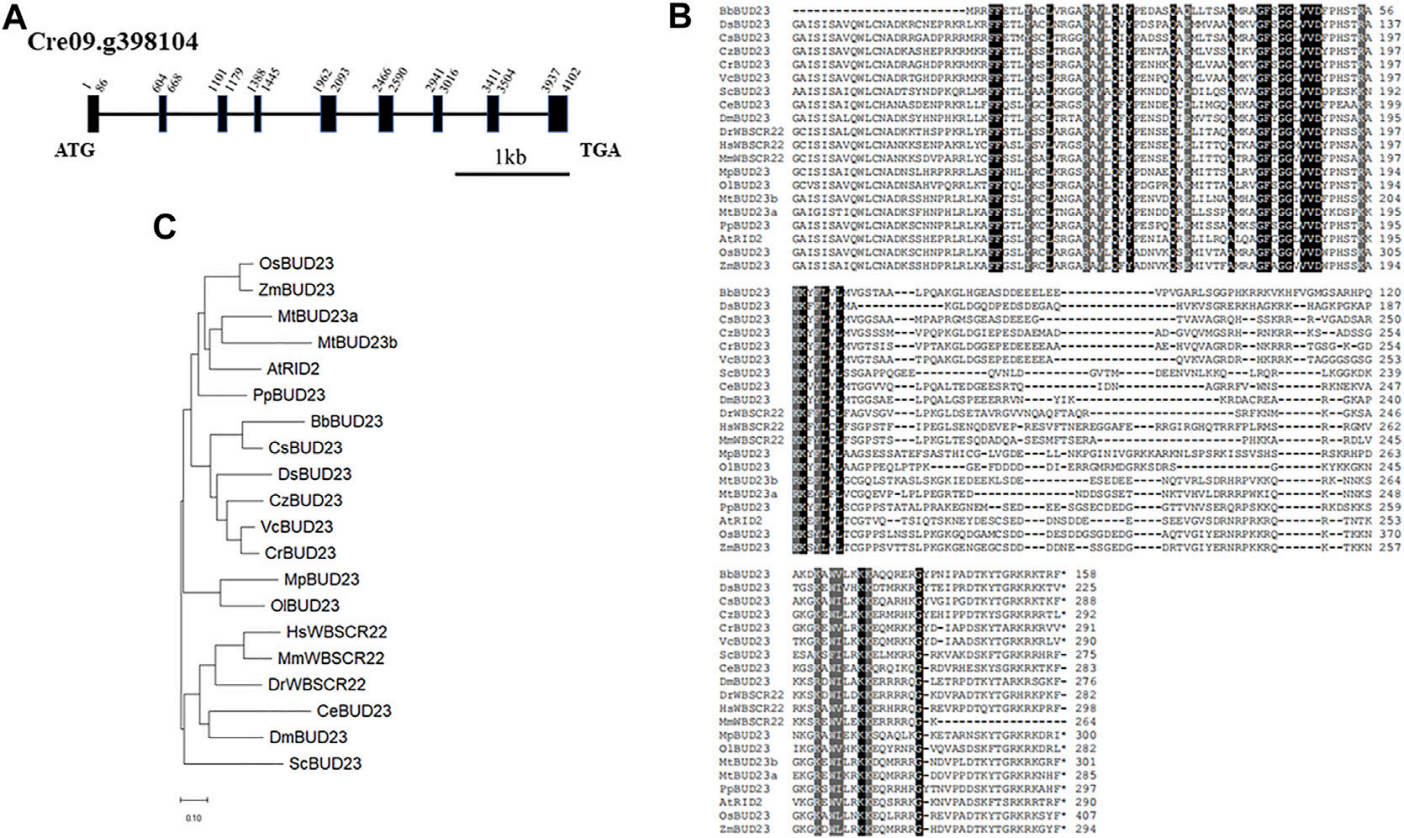
CrBUD23 is a 18S rRNA methyltransferase. **(A)** Structure of CrBUD23 (Cre09. g398104). **(B)** Conserved motifs in BUD23 homologs. **(C)** Phylogenetic trees of BUD23 homologs.

Nucleotide methylation in ribosomal RNA (rRNA) is a common feature in all living organisms. Up to now, 18S rRNA methylation have been identified experimentally in yeast, mouse and human. To identify the putative 18S rRNA methyltransferase genes in green algae, we first performed a sequence similarity search using BLAST analysis in the JGI-Phytozome (Goodstein et al., 2012) database for Chlorophyte *C. reinhardtii*, *Chromochloris zofingiensis*, *Coccomyxa subellipsoidea*, *Micromonas pusilla* CCMP1545, *Micromonas* sp. RCC299, *Ostreococcus lucimarinus* and *Volvox carteri*, Embryophyte *Arabidopsis thaliana*, *Medicago truncatula*, *Oryza sativa*, and *Zea mays*. Pairwise Sequence Alignment (https://www.ebi.ac.uk/Tools/psa/emboss_needle/) (McWilliam et al., 2013) results revealed that the candidate gene Cre09.g398104 showed the highest similarity (51.0% protein identity and 67.0% protein similarity) to the *Homo sapiens* WBSCR22. In the genomic sequence of *C. reinhardtii*, Cre09.g398104 has an 873 bp of coding sequence and consists of nine exons, nine introns, including one intron in 5’-UTR (Figure 1A). The complete amino acid sequence of CrBUD23 showed high similarity (74.6% and 63.0%, respectively) to that of other proteins of the dicotyledonous plant *A. thaliana* (At5g57280) and yeast *S. cerevisiae* (NP_009976.1) (Figure 1B). BUD23-related protein sequences were derived from the JGI database and Genbank in NCBI database using BLAST with ScBUD23, and a phylogenetic tree was constructed using the neighbor-joining method in the MEGA 11.0.13 (Tamura et al., 2021). The proteins located in the clade including human WBSCR22 were named as WBSCR22, and others were named as BUD23 refer to yeast BUD23. The phylogenetic tree indicated that CrBUD23 sequences had high similarity with those of the freshwater green algae *Volvox carteri* and generated one cluster with those of the halophilic microalgae *Dunaliella salina* and oil-producing microalgae *Chromochloris zofingiensis*, and they belonged to a clade different from those of higher plants and animals (Figure 1C). An interesting finding was that the BUD23 of two Chlorophyte organisms *Ostreococcus lucimarinus* and *Micromonas pusilla* formed a different clade from other green algae, land plants and animals. The characterization of the sequence of CrBUD23 protein implied that this putative 18S rRNA methyltransferase was conserved and closely related to those of other green algae, high plants and animals. The shortest BUD23 in green algae *Botryococcus braunii* also maintained the domain of methyltransferase activity (Figure 1B). Clearly, BUD23 is a conserved eukaryotic methyltransferase.

Both yeast and Arabidopsis knockout mutants demonstrated BUD23 is essential for pre-rRNA processing reactions leading to synthesis of 18S rRNA (White et al., 2008; Ohbayashi et al., 2011). Notably, a *Scbud23* mutant could be complemented by *bud23* mutations that possesses inactivated methyltransferase activity. Because both G57R and D77A mutants was able to rescue the phenotypes of the *bud23* null mutant, ribosome biogenesis requires the presence of this methyltransferase rather than its methyltransferase activity in both human cells and budding yeast (Lin et al., 2012). The corresponding sites of G57 and D77 in CrBUD23 were G63 and D82, respectively (Supplementary Table S1). Even though BbBUD23 and DsBUD23 lack the N terminal motif conserved in other BUD23 proteins (Supplementary Table S1), this conserved motif should be related with certain function of BUD23 protein. Otherwise, the conservation of G63/G57 and D82/D77 would disappear during evolution.

### 3.2 High light activates transcription of CrBUD23

**FIGURE 2:**
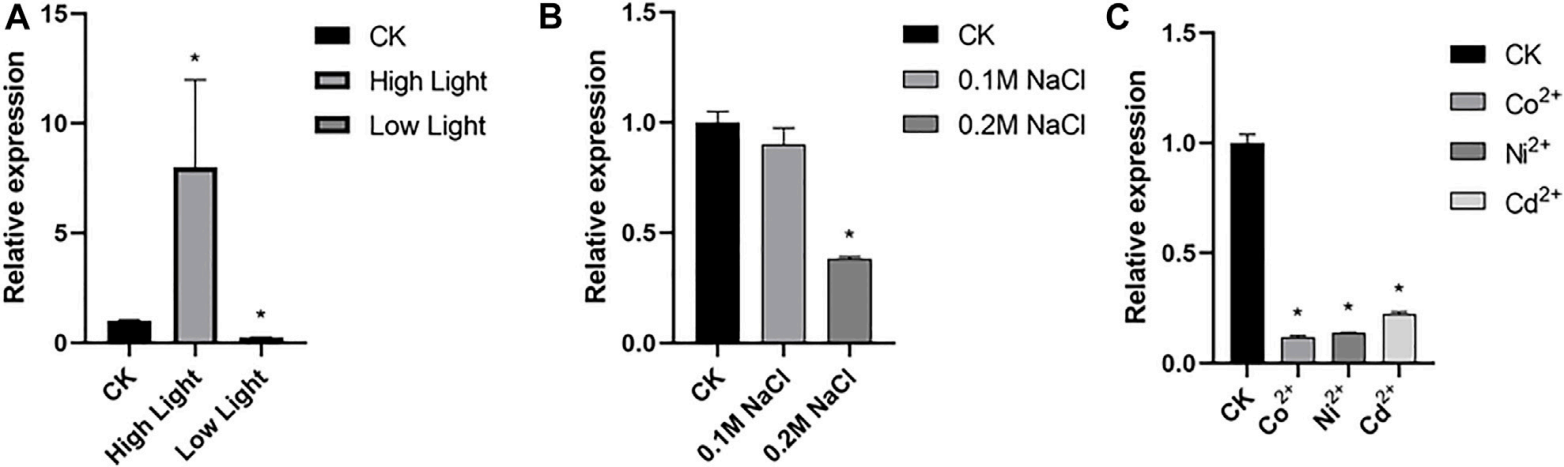
Transcription of CrBUD23 is upregulated by high light. **(A)** qRT-PCR analysis of CrBUD23 under high light and low light. CK, 25 μmol photons/m^2^s. High light, 75 μmol photons/m^2^s. Low light, 8 μmol photons/m^2^s. **(B)** qRT-PCR analysis of CrBUD23 under modulate (0.1 M NaCl) and severe (0.2 M NaCl) salt stresses. **(C)** qRT-PCR analysis of CrBUD23 under heavy metal stress. Error bars represent the mean value from three independent experiments. Statistically significant differences were determined by Student’s t-test (**p < 0.01, *p < 0.05).

Microalgae is widely distributed in waterbody worldwide and is a cell factory driven by sunlight. Through efficient photosynthesis, microalgae cells absorb CO_2_, convert light energy into chemical energy of carbohydrates such as starch or oil, and emit O_2_ (Allorent et al., 2013). qRT-PCR assay revealed transcription of CrBUD23 was upregulated by high light and down-regulated by low light (Figure 2A). *C. reinhardtii* was capable to grow in TAP medium plus 0.25 M NaCl but triggered cell death by treatment with 0.3 M NaCl. Transcription levels of CrBUD23 was unaffected by modulate salinity stress (0.1 M NaCl) (Figure 2B). While severe salinity stress (0.2 M NaCl) significantly negatively regulated abundance of CrBUD23 mRNA (Figure 2B). Moreover, heavy metal ion was applied into TAP medium to test the effectiveness of heavy metal ion on transcription of CrBUD23. Results of qRT-PCR assay showed that CrBUD23 was downregulated by heavy metal stresses (Figure 2C). Taken together, these results showed that the CrBUD23 protein were essential for responses to light but not necessary towards salinity stress or heavy metal stress.

### 3.3 Overexpression of CrBUD23 enhances cell growth and biomass of *C. reinhardtii*

**FIGURE 3:**
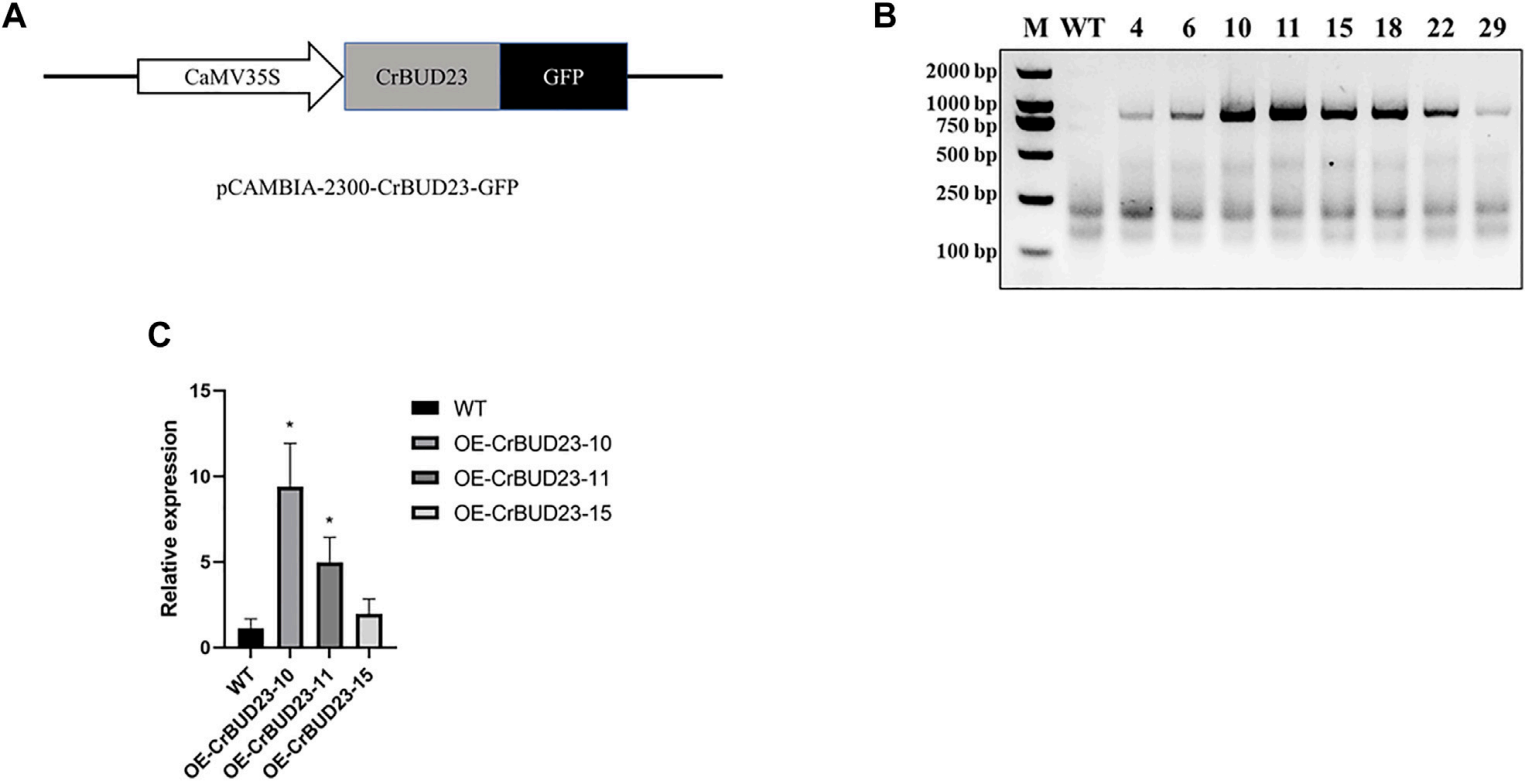
Generation of *C. reinhardtii* transgenic strains overexpressing CrBUD23. **(A)**. Schematic diagram of CrBUD23 overexpression cassettes in pCAMBIA2300-CaMV35S:CrBUD23-GFP. **(B)**. Genomic PCR of the wild-type (WT) and transgenic strains integrated with CrBUD23-GFP. M, Marker; WT, CC-5325; Arabic numerals, transformants with pCAMBIA2300-CaMV35S:CrBUD23-GFP. **(C)**. qRT-PCR analysis of transgenic strains OE-CrBUD23-10, OE-CrBUD23-11, OE-CrBUD23-15.

A number of studies showed BUD23 emerges as essential to development of budding yeast (Black et al., 2020) and growth and WBSCR22 is a positive regulator on growth of human cells (Ounap et al., 2013) and murine cardiomyocytes (Baxter et al., 2020), so it was hypothesized that overexpression of this putative 18S rRNA methyltransferase could promote the growth of microalgae through manipulating ribosome biogenesis pathway. To enhance CrBUD23 expression in *C. reinhardtii* CC-5325, we cloned the full-length of coding sequence (CDS) of CrBUD23 (Cre09. g398104) and constructed the vector pCAMBIA2300-CrBUD23-GFP, where CrBUD23 fused with a green fluorescence tag was driven by the CaMV35S promoter (Figure 3A). The widely-used wild type strain of *C. reinhardtii* CC-5325 was transformed with pCAMBIA2300-CrBUD23-GFP by glass bead mediated transformation method to yield transgenic strain OE-CrBUD23. The chromosomal integration of the full-length of CrBUD23 CDS in eight transgenic strains were confirmed by genomic PCR with the primers CrBUD23-F and CrBUD23-R (Figure 3B). Then, three of these eight transgenic strains were used for qRT-PCR assay to evaluate the transcription level of CrBUD23. qRT-PCR analysis showed higher transcription levels of CrBUD23 in the strains OE-CrBUD23-10 and OE-CrBUD23-11 compared to the parental strain expressing endogenous CrBUD23 (Figure 3C).

**FIGURE 4:**
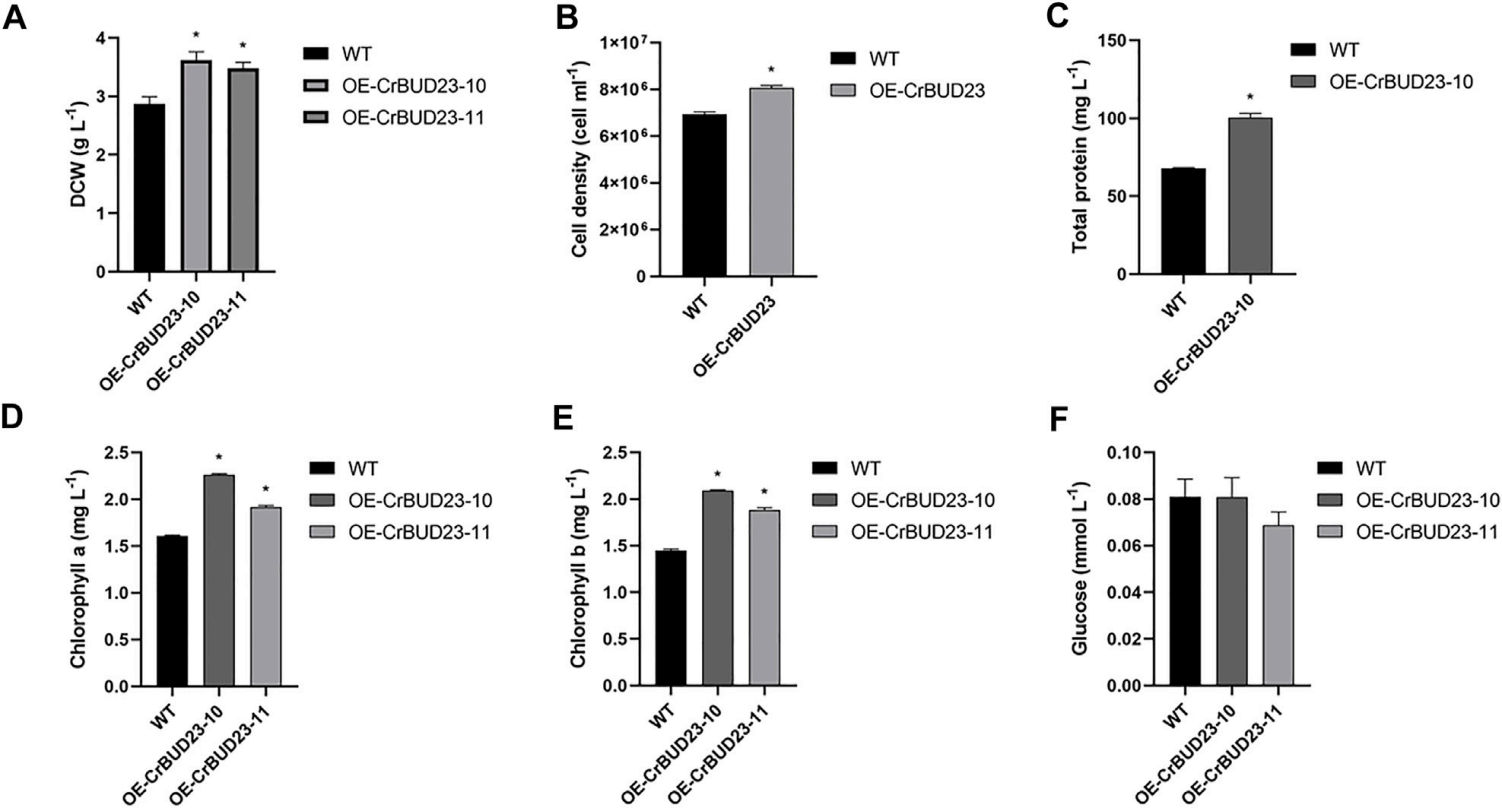
Overexpression of CrBUD23 enhances cell growth and biomass. **(A)**. Dry weight of WT, transgenic strains OE-CrBUD23-10 and OE-CrBUD23-11. **(B)**. Cell density of WT, transgenic strains OE-10. **(C)**. Total protein content in WT, transgenic strains OE-10. **(D)**. Chlorophyll a content in WT, transgenic strains OE-10 and OE-11. **(E)**. Chlorophyll b content in WT, transgenic strains OE-10 and OE-11. **(F)**. Glucose content in WT, transgenic strains OE-10 and OE-11. Different letters above the bars indicate significant difference at *p* < 0.01. Error bars indicate SD (*n* = 6). Letters above the bars indicate significant differences (*p* < 0.01). Similar results were obtained in three independent experiments.

Figure 4 showed the growth of wild type strain CC-5325 and BUD23-overexpressing strains cultivated at lab-scale. Under 16 h light and 8 h dark conditions, both OE-CrBUD23-10 and OE-CrBUD23-11 showed higher growth rates than CC-5325. After 1 week cultivation, the dry cell weight (DCW) of CC-5325 was 2.87 ± 0.12 g L^−1^, while OE-CrBUD23-10 and OE-CrBUD23-11 reached 3.62 ± 0.14 g L^−1^ and 3.48 ± 0.10 g L^−1^, respectively (Figure 4A). According to statistical analysis, the biomass and the cell density of transgenic strains was significantly higher than that of CC-5325 (Figures 4A, B). Measurements of total protein content showed that OE-CrBUD23-10 had a higher protein content (100.5 mg L^−1^) than that of control (67.8 mg L^−1^) (Figure 4C). Quantification results showed algal cells of overexpressing CrBUD23 strains contained more chlorophyll a (Figure 4D) and chlorophyll b (Figure 4E) than the wild type CC-5325. Moreover, the average contents of chlorophyll a and chlorophyll b in a single algal cell were calculated according to the cell number in Figure 4B. Analyzed results showed overexpressing CrBUD23 indeed enhanced the chlorophyll contents per cell in *C. reinhardtii*, compared with wild type (Supplementary Figures S1A,B). Similarly, the cells of transgenic strain OE-CrBUD23-10 contained more protein than those of CC-5325 (Supplementary Figure S1C), consistent with the trend observed in Figure 4C. However, in the quantification test of glucose, there was no significant differences between control and transgenic strains (Figure 4F). Together, overexpressing CrBUD23 caused enhanced accumulation of chlorophyll a and chlorophyll b, accelerated growth rate and higher biomass in *C. reinhardtii*, suggesting CrBUD23 positively regulated growth of microalgae. Notably, we found transcription of endogenous CrBUD23 was induced by high light (Figure 2A). Thus, it was speculated that the microalgae could make use of the excess energy provided by the high light through induction of CrBUD23 expression to regulate function of ribosome thereby reprogramming the translation globally or specifically.

### 3.4 Overexpression of CrBUD23 increases lutein content of *C. reinhardtii*

**FIGURE 5:**
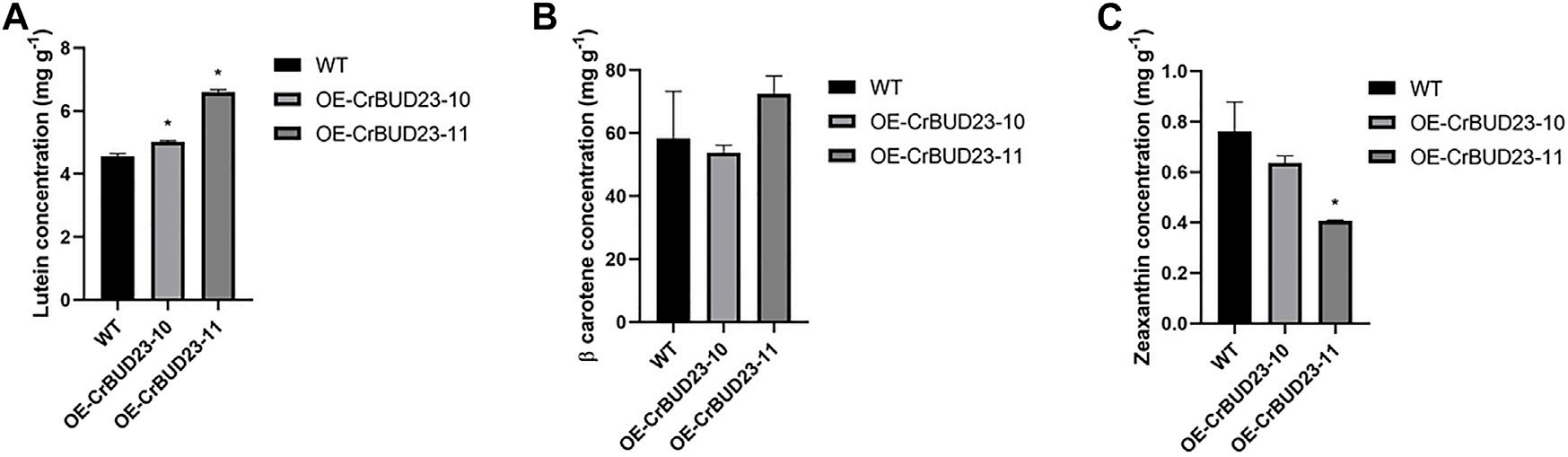
Overexpression of CrBUD23 increases lutein content. **(A)** Lutein content in WT, transgenic strains OE-10 and OE-11. **(B)** β-carotene content in WT, transgenic strains OE-10 and OE-11. **(C)** Zeaxanthin content in WT, transgenic strains OE-10 and OE-11.

As an antioxidative photosynthetic pigment, carotenoid protects microalgae from oxidative stress under high light and other extreme environmental conditions (Polle et al., 2001; Minhas et al., 2016). In *C. reinhardtii*, an increase in lutein production were observed under a number of abiotic stress conditions, especially under high light where the lutein content was significantly higher than that under the other stress conditions (Baroli et al., 2003; Bonente et al., 2011). Lutein plays a role in protecting the eyes and alleviating visual fatigue, but it cannot be synthesized in the human body (Lou et al., 2021). Importantly, lutein can only be obtained through diet, so *C. reinhardtii* and some other green algae are natural bioreactors for lutein production. Here, we used High Performance Purity Liquid Chromatography (HPLC) method to quantify the content of β-carotene, lutein and zeaxanthin. It was found that OE-CrBUD23-10 and OE-CrBUD23-11had 5.04 mg g^−1^ and 6.54 mg g^−1^ lutein after 1 week cultivation, which were 27.3% and 65.1% higher than that of the wild type CC-5325, respectively (Figure 5A) (*p* < 0.01). While the β-carotene content in OE-CrBUD23-10 and OE-CrBUD23-11 were almost unaltered, compared with that of CC-5325. On the other hand, the zeaxanthin content in OE-CrBUD23-10 and OE-CrBUD23-11 was 0.62 mg g^−1^ and 0.75 mg g^−1^, which was as similar as that of the control (Figure 5B) (*p* < 0.01). The zeaxanthin content in OE-CrBUD23-11 was significantly lower than that of the wild type CC-5325 and OE-CrBUD23-10 (Figure 5C). It was possible that the insertion of T-DNA in OE-CrBUD23-11 was correlated with some important regulators involved in zeaxanthin biosynthesis. Another possibility was that some variations happened during transformation and resulted in the altered accumulation of zeaxanthin. Overall, these results revealed that overexpression of CrBUD23 lead to increase of lutein content, indicating CrBUD23 was a positive regulator in lutein biosynthesis.

### 3.5 Overexpression of CrBUD23 maintains redox homeostasis

**FIGURE 6:**
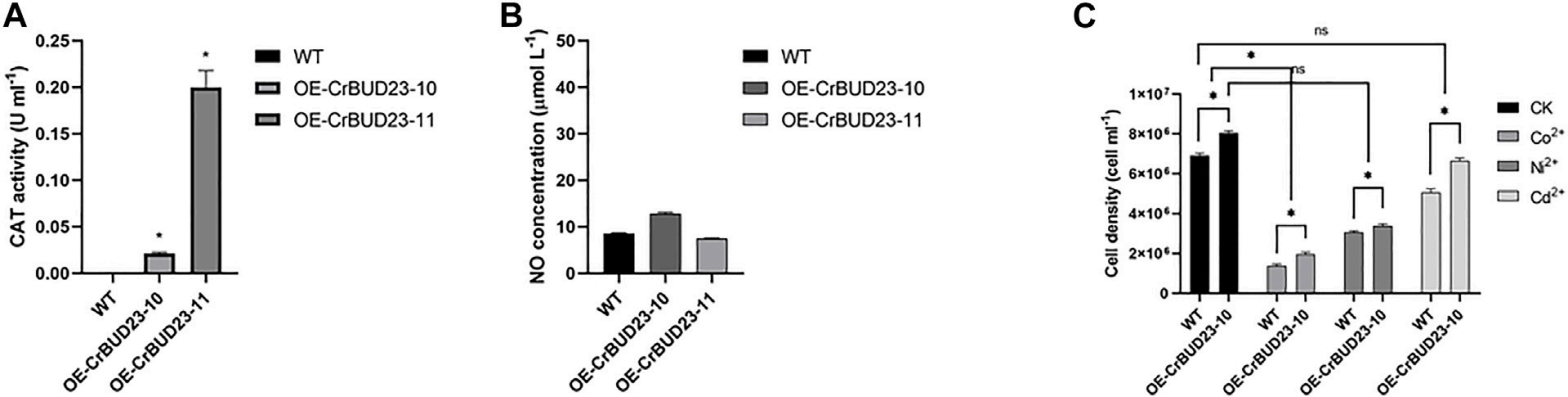
Overexpression of CrBUD23 maintains redox homeostasis. **(A)**. CAT activity of WT, transgenic strains OE-CrBUD23-10 and OE-CrBUD23-11. **(B)**. NO content in WT, transgenic strains OE-10 and OE-11. **(C)**. Cell density of WT, transgenic strains OE-10 and OE-11 under heavy metal stresses.

In microalgae, carotenoids containing numerous conjugated double bonds mainly achieve the role of antioxidation and cell protection by neutralizing peroxide free radicals (Christaki et al., 2013). Thus, altered pattern of carotenoid in transgenic strains might result from the redox homeostasis disturbed by overexpressed CrBUD23. Interestingly, results revealed the activity of antioxidative enzymes catalase (CAT) (Figure 6A) and the content of second massager Nitric oxide (NO) (Figure 6B) was unaltered in transgenic strains. Thus, the possibility that redox homeostasis alternations caused by overexpressing CrBUD23 lead to increased lutein content was excluded. In addition, overexpression of CrBUD23 did not cause morphological defects, abnormal movements and deteriorate tolerance toward salinity stress or heavy metal stress (Figure 6C). Taken together, the differences in biomass and lutein content between OE-CrBUD23 strains and WT microalgae were not be secondary effects caused by disruption of redox homeostasis, which could be elicited by overexpression of some proteins. It should be noted that the different effectiveness on specific carotenoid biosynthesis pathway and unaltered antioxidation status in algal cells also reflected that overexpression of CrBUD23 affected translation specifically but not globally.

### 3.6 Conserved G1177 is the putative methylation site in 18S rRNA of *C. reinhardtii*

**FIGURE 7:**
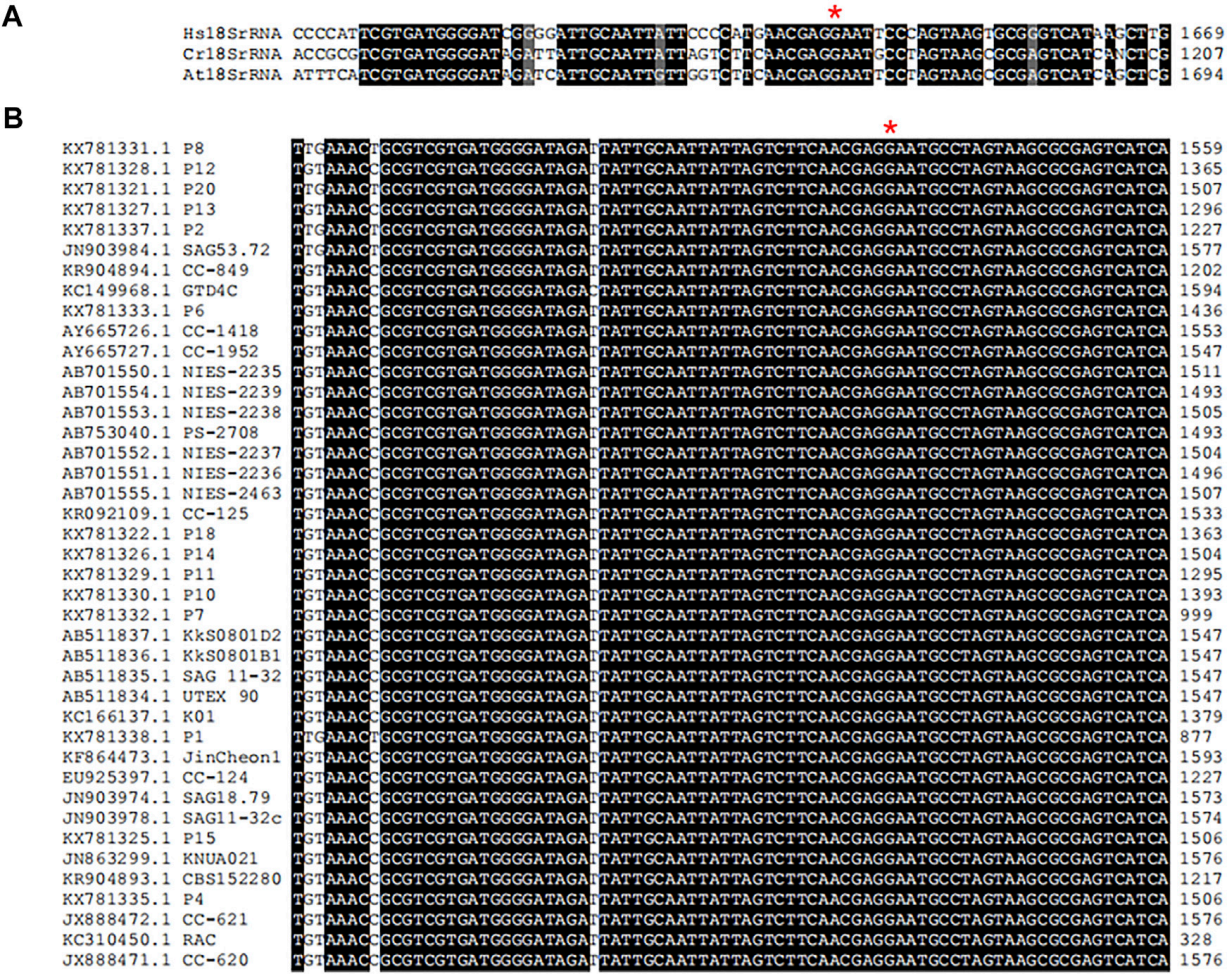
Conserved motif in 18S rRNA in Chlorophyte. **(A)** alignment of 18S rRNAs of *H. sapiens*, *C. renhardtii* and *A. thaliana*. **(B)** conserved G1177 in 41 *C. renhardtii* isolates.

In multicellular organisms, ribosome function is associated with development, growth and cell cycle (Barozzi et al., 2022). Eukaryotic rRNA are modified frequently, and twelve different types of rRNA modifications have been characterized in yeast (Sloan et al., 2017). In *S*. *cerevisiae*, it was found that a single ribosomal protein from the large subunit contacted with four modified rRNA base across the ribosomal subunit interface at the late stages of ribosome assembly (Letoquarta et al., 2014). HsWBSCR22 and ScBUD23 are 18S rRNA methyltransferases belonging to S-adenosylmethionine-dependent Rossmann-fold methyltransferase superfamily and account for N-7-methylation of G1639 (Haag et al., 2015) and G1575 (White et al., 2008) in human and yeast18S rRNA, respectively. Knockout of ScBUD23 gene in yeast can observe the reduction of cell growth, leading to the reduction of cell growth. The human WBSCR22 partially complements the growth of *bud23* deletion mutant suggesting that WBSCR22 is a functional homologue of yeast BUD23. BUD23-dependent ribosome generation in nucleus is related with modulation of nuclear protein translation. Researchers have gained a certain understanding of the structure of BUD23 and how it methylated 18S rRNA, but little was known about the process of 18S rRNA methylation in green algae. In this study, we identified the 18S rRNA sequence in the genome of *C. reinhardtii* CC-849 (KR904894.1) in NCBI database. Then, it was aligned with sequences of human 18S rRNA (XR_007090847.1) and Arabidopsis 18S rRNA (X16077.1) that was also from NCBI database (Supplementary Table S2). Alignment results showed that the G1177 in CC-849 was identical to G1639 in human (Figure 7A). Moreover, alignment of 18S rRNAs from 41 *C. reinhardtii* isolates in NCBI database showed that the G1177 in CC-849 is highly conserved in all other 18S rRNA (Figure 7B). Therefore, this guanosine conserved in *C. reinhardtii* could be the methylation site mediated by CrBUD23 as observed in human and yeast. However, further investigations were hold on due to the lack of appropriate genetic materials. The advanced genome editing technology should be applied to generate *Crbud23* mutant in the following research, and then the methylation status of G1177 could be detected and quantified.

The 18S rRNA methyltransferase in Arabidopsis has been characterized. AtRID2 (At5g57280), the homolog of BUD23, was temporarily annotated as a methyltransferase-like protein involved in pre-rRNA processing. The alignment in this study also pointed out that the G1664 in Arabidopsis 18S rRNA is identical to the G1639 in 18S rRNA of *H.* sapiens (Figure 7A), suggesting the homologous gene AtRID2 could add methyl to the fourth N on guanosine G of 1664 nucleotides in 18S rRNA. Different from wild type, the AtRID2 null mutant usually developed twisted and narrower leaves with deeper indentations, scarcely regenerated adventitious shoots and formed irregular mounds of cells at 25°C. Also, seedlings of *Atrid2* mutant displayed a pointed-leaf phenotype which is often detected in Arabidopsis mutants related to ribosomes (Shinohara et al., 2014). Cell proliferation in Arabidopsis *rid2* mutant was suppressed at 28°C, consistent with the similar phenotypes observed in yeast *bud23* mutant and human cells lacking functional WBSCR22. Recent advances in cryo-electron microscopy have led to high-resolution structures of many pre-ribosomal intermediates; however, these static snapshots do not capture the dynamic transitions between these intermediates. To this end, molecular genetics can be leveraged to reveal how the biogenesis factors drive these dynamic transitions. Thus, further investigations should pay attention to the patterns of rRNA precursors in CrBUD23 knockout mutant.

Besides, in view of the conservation on BUD23, 18S rRNA and carotenoid synthesis network, ribosomes may be involved in the intermediate link of carotenoid synthesis, which is mainly processed in chloroplast in algae and plants. It was also discovered that the yeast BUD23 specifically enhances ribosomal interaction with low GC-content 5′UTRs thereby promoting efficient translation of mRNA transcripts with low 5′UTR GC content (Baxter et al., 2020). Further investigations are required for in-depth understanding of mechanism of CrBUD23 in algae and plants.

### 3.7 CrTrm112 is involved in high light responses

**FIGURE 8:**
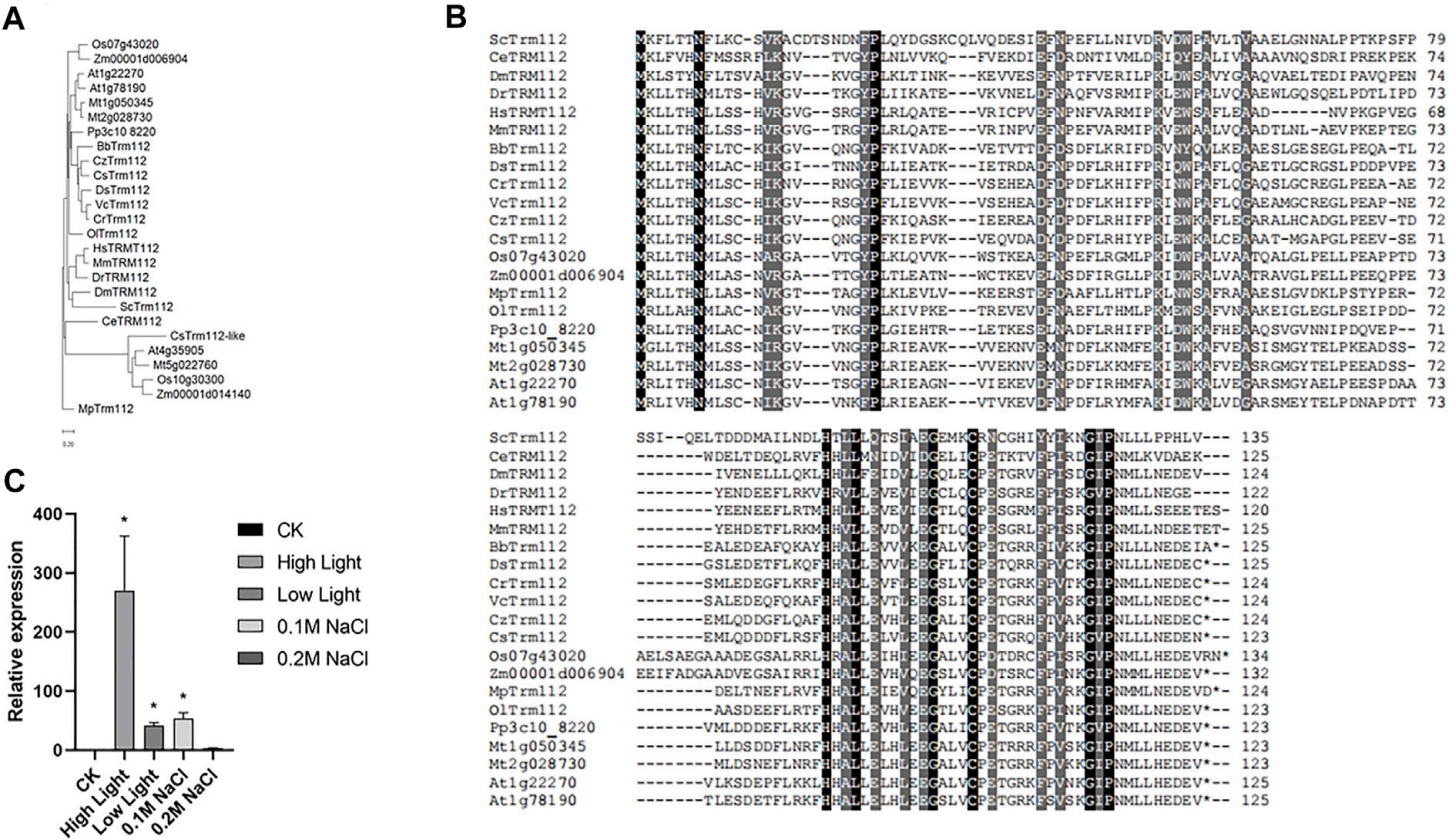
Transcription of CrTrm112 is upregulated by high light. **(A)**. Phylogenetic trees of Trm112 homologs. **(B)**. Alignment of Trm112 homologs. **(C)**. qRT-PCR assay of CrTrm112 under high light, low light, modulate (0.1 M NaCl) and severe (0.2 M NaCl) salt stresses.

Expression of ribosome proteins is tightly regulated in response to internal and external stimuli (Venema and Tollervey, 1999; Bassler and Hurt, 2019). In yeast, Trm112 is characterized as an interactor of BUD23, and interaction between BUD23 and Trm112 is required for ribosome maturation (Figaro et al., 2012). In *Sctrm112* mutant, ScBUD23 failure to bind nascent pre-ribosomes activates a nucleolar surveillance pathway involving the TRAMP complexes, leading to degradation of pre-ribosomes (Sardana and Johnson, 2012). Furthermore, structural and functional studies on BUD23-Trm112 complex reveal that BUD23 and Trm112 interact with each other by forming a beta-zipper and burying a hydrophobic surface which stabilizes this interaction (Letoquarta et al., 2014). The structures of BUD23-Trm112 indicates that the coactivator Trm112 undergoes an induced fit to accommodate its methyltransferase partner such as BUD23 (Letoquarta et al., 2014). Human WBSCR22-TRMT112 are the functional homologues of yeast Bud23-Trm112 (Zorbas et al., 2015). In this study, we identified Cre12. g512550 (CrTrm112) in the *C. reinhardtii* genome using BLAST with Trm112 proteins of *S. cerevisiae* as query (Supplementary Table S4). Notably, among the organisms present in phylogenetic tree, only alfalfa genome had two BUD23 genes (Figure 1B). Similarly, there were two Trm112 genes in alfalfa as well (Figure 8A). While Arabidopsis genome contained two Trm112 genes and only one BUD23 (Figure 1B; Figure 8A). In addition to the classical Trm112 clade, there was a clade consist of five Trm112-like proteins from one green algae *Coccomyxa subellipsoidea*, two dicotyledonous plants *Arabidopsis thaliana* and *Medicago truncatula*, and two monocotyledonous plants *Oryza sativa* and *Zea mays*. These results indicated that high plants had this Trm112-like subfamily. Interestingly, *Caenorhabditis elegans* BUD23 generated a cluster with that of *Drosophila melanogaster*, but *Caenorhabditis elegans* Trm112 formed a branch alone and was further away from *Drosophila melanogaster* Trm112 than ScTrm112. Alignments of all proteins in the classical Trm112 clade displayed the high similarity of Trm112 homologs, suggesting the conservation of the function of Trm112 protein (Figure 8B).

To evaluate the effects of different factors on transcription of CrTrm112, we performed qRT-PCR analysis. In wild type CC-5325, the expression of Trm112 was largely induced under high light conditions compared to expression under normal light conditions, indicating that CrTrm112 was regulated by high light at transcriptional level as well (Figure 8C). These results revealed that transcription of CrTrm112 was unaltered under several stresses as same as behaviors of CrBUD23, suggesting the BUD23-Trm112 complex is mainly induced through elevation of BUD23 abundance in microalgae.

Most of the factors involved in translation (tRNA, rRNA and proteins) are subject to post-transcriptional and post-translational modifications, which participate in the fine-tuning and tight control of ribosome and protein synthesis processes (Xue and Barna, 2012). In eukaryotes, Trm112 acts as an obligate activating platform for another three methyltransferases (MTase) involved in the modification of tRNA (Trm9 and Trm11) and translation termination factor eRF1 (Mtq2), except for BUD23 related with the 18S rRNA (Letoquart et al., 2015; Bourgeois et al., 2017a; Bourgeois et al., 2017b; Tran et al., 2018). Besides, although Trm112 is required for BUD23 stability, it was reported that Trm112 is not maintained in a stable complex with BUD23. Thus, it was proposed that Trm112 stabilizes its free methyltransferase partners not engaged with substrate and/or helps to deliver its methyltransferase partners to their substrates.

## 4 Conclusion

In summary, CrBUD23 encodes a putative 18S rRNA methyltransferase homologous to human WBSCR22 and positively regulates cell growth, as transgenic *C. reinhardtii* strains overexpressing CrBUD23 resulted in higher biomass and lutein bioproduction. Our findings will shed light on the study on microalgae productivity and may be useful to increase the yield of macroalgae and high plants. Furthermore, these insights will contribute to molecular breeding of a high-yield bioproduction platform for high-value chemicals and recombinant proteins in microalgae.

## Data Availability

The original contributions presented in the study are included in the article/Supplementary Material, further inquiries can be directed to the corresponding authors.
