# Supplementary material for: Overexpression of 18S rRNA methyltransferase CrBUD23 enhances biomass and lutein content in Chlamydomonas reinhardtii
Source: Front Bioeng Biotechnol. 2023 Feb 3;11:1102098. doi: 10.3389/fbioe.2023.1102098 (PMC9935685; doi:10.3389/fbioe.2023.1102098)
Supplement: Supplementary file 1 [file DataSheet2.PDF]

Color Align Conservation results

```
Hs18SrRNA -------------------------------------------------------------------------------- 0
Cr18SrRNA -------------------------------------------------------------------------------- 0
At18SrRNA AAAAGATGACGGTCAAGACCTCGTCCTTTCTCTCTTTCCATTGCGTTTGAGAGGATGTGGCGGGGAATTGCCGTGATCGA 80

Hs18SrRNA -------TACCTGGTTGATCCTGCC-AGTAGCATATGCTTGTCTCAAAGATTAAGCCATGCATGTCTAAGTACGCACGGC 72
Cr18SrRNA -------------------------------------------------------------------------------- 0
At18SrRNA TGAATGCTACCTGGTTGATCCTGCCAGTAGTCATATGCTTGTCTCAAAGATTAAGCCATGCATGTGTAAGTATGAACGAA 160

Hs18SrRNA C-GGTACAGTGAAACTGCGAATGGCTCATTAAATCAGTTATGGTTCCTTTGGTCGCTCGCTCCTCTCCTACTTGGATAAC 151
Cr18SrRNA -------------------------------------------------------------------------------- 0
At18SrRNA TTCAGACTGTGAAACTGCGAATGGCTCATTAAATCAGTTATAGTTTGTTTGATGGTAAC------TACTACTCGGATAAC 234

Hs18SrRNA TGTGGTAATTCTAGAGCTAATACATGCCGACGGGCGCTGACCCCCTTCGCGGGGGGGATGCGTGCATTTATCAGATCAAA 231
Cr18SrRNA -------------------------------------------------------------------------------- 0
At18SrRNA CGTAGTAATTCTAGAGCTAATACGTGCAACAAACCCCGACTTA----------TGGAAGGGACGCATTTATTAGATAAAA 304

Hs18SrRNA ACCAACCCGGTCAGCCCCTCTCCGGCCCCGGCCGGGGGGCGGGCGCCGGCGGCTTTGGTGACTCTAGATAACCTCGGGCC 311
Cr18SrRNA -------------------------------------------------------------------------------- 0
At18SrRNA GGTCG------------------------------ACGCGGGCTCTGGCTTGCTCTGATGATT--CATGATAACTCGACG 352

Hs18SrRNA GATCGCACGCCCCCCGTGGCGGCGACGACCCATTCGAACGTCTGCCCTATCAACTTTCGATGGTAGTCGCCGTGCCTACC 391
Cr18SrRNA -------------------------------------------------------------------------------- 0
At18SrRNA GATCGCATGGCCTCTGTGCTGGCGACGCATCATTCAAATTTCTGCCCTATCAACTTTCGATGGTAGGATAGTGGCCTACC 432

Hs18SrRNA ATGGTGACCACGGGTGACGGGGAATCAGGGTTCGATTCCGGAGAGGGAGCCTGAGAAACGGCTACCACATCCAAGGAAGG 471
Cr18SrRNA ---------------------------------------------------TGAGAGATGGCTACCACATCCAAGGAAGG 29
At18SrRNA ATGGTGGTAACGGGTGACGGAGAATTAGGGTTCGATTCCGGAGAGGGAGCCTGAGAAACGGCTACCACATCCAAGGAAGG 512

Hs18SrRNA CAGCAGGCGCGCAAATTACCCACTCCCGACCCGGGGAGGTAGTGACGAAAAATAACAATACAGGACTCTTTCGAGGCCCT 551
Cr18SrRNA CAGCAGGCGCGCAAATTACCCAATCCCGACACGGGGAGGTAGTGACAATAAATAACAATACCGGGCGC-TTCGCG-TCTG 107
At18SrRNA CAGCAGGCGCGCAAATTACCCAATCCTGACACGGGGAGGTAGTGACAATAAATAACAATACCGGGCTCTTTCGAG-TCTG 591

Hs18SrRNA GTAATTGGAATGAGTCCACTTTAAATCCTTTAACGAGGATCCATTGGAGGGCAAGTCTGGTGCCAGCAGCCGCGGTAATT 631
Cr18SrRNA GTAATTGGAATGAGTACAATCTAAATCCCTTAACGAGGATCCATTGGAGGGCAAGTCTGGTGCCAGCAGCCGCGGTAATT 187
At18SrRNA GTAATTGGAATGAGTACAATCTAAATCCCTTAACGAGGATCCATTGGAGGGCAAGTCTGGTGCCAGCAGCCGCGGTAATT 671

Hs18SrRNA CCAGCTCCAATAGCGTATATTAAAGTTGCTGCAGTTAAAAAGCTCGTAGTTGGATCTTGGGAGCGGGCGGGCGGTCCGCC 711
Cr18SrRNA CCAGCTCCAATAGCGTATATTTAAGTTGTTGCAGTTAAAAAGCTCGTAGTTGGATTTCGGGTGGGGTGGTGCGGTCCGCC 267
At18SrRNA CCAGCTCCAATAGCGTATATTTAAGTTGTTGCAGTTAAAAAGCTCGTAGTTGAACCTTGGGATGGGTCGGCCGGTCCGCC 751

Hs18SrRNA GCGAGGCGAGCCACCGCCCGTCCCCGCCCCTTGCCTCTCGGCGCCCCCTCGATGCTCTTAGCTGAGTGTCCCGCGGGGCC 791
Cr18SrRNA TCTGGTGTGCACTGCTCTGCTCCACCTTCCTG-----CCGGGGACGGGCTCCTGGGCTTCACTGTCTGGGA-CTCGGAGT 341
At18SrRNA TTTGGTGTGCATTGGTCGGCTTGTCCCTTCGG-----TCGGCGATACGCTCCTGGTCTTAATTGGCCGGGT-CGTGCCTC 825

Hs18SrRNA CGAAGCGTTTACTTTGAAAAAATTAGAGTGTTCAAAGCAGGCCCGAGCCGCCTGGATACCGCAGCTAGGAATAATGGAAT 871
Cr18SrRNA CGGCGAGGTTACTTTGAGTAAATTAGAGTGTTCAAAGCAGGCCTACGCT--CTGAATACATTAGCATGGAATAACACGAT 419
At18SrRNA CGGCGCTGTTACTTTGAAGAAATTAGAGTGCTCAAAGCAAGCCTACGCT--CTGGATACATTAGCATGGGATAACATCAT 903

Hs18SrRNA AGGACCGCGGTTCTATTTTGTTGGTTTTCGGAACTGAGGCCATGATTAAGAGGGACGGCCGGGGGCATTCGTATTGCGCC 951
Cr18SrRNA AGGACTCTGGC-CTATC-TGTTGGTCTGTGGGACCGGAGTAATGATTAAGAGGGGTAGTCGGGGGCATTCGTATTCCGTT 497
At18SrRNA AGGATTTCGATCCTATT-GTGTTGGCTTCGGGATCGGAGTAATGATTAACAGGGACAGTCGGGGGCATTCGTATTTCATA 982

Hs18SrRNA GCTAGAGGTGAAATTCTTGGACCGGCGCAAGACGGACCAGAGCGAAAGCATTTGCCAAGAATGTTTTCATTAATCAAGAA 1031
Cr18SrRNA GTCAGAGGTGAAATTCTTGGATTTACGGAAGACGAACATCTGCGAAAGCATTTGCCAAGGATACTTTCATTGATCAAGAA 577
At18SrRNA GTCAGAGGTGAAATTCTTGGATTTATGAAAGACGAACAACTGCGAAAGCATTTGCCAAGGATGTTTTCATTAATCAAGAA 1062

Hs18SrRNA CGAAAGTCGGAGGTTCGAAGACGATCAGATACCGTCGTAGTTCCGACCATAAACGATGCCGACCGGCGATGCGGCGGCGT 1111
Cr18SrRNA CGAAAGTTGGGGGCTCGAAGACGATTAGATACCGTCGTAGTCTCAACCATAAACGATGCCGACTAGGGATTGGCAGATGT 657
At18SrRNA CGAAAGTTGGGGGCTCGAAGACGATCAGATACCGTCCTAGTCTCAACCATAAACGATGCCGACCAGGGATCAGCGGATGT 1142

Hs18SrRNA TATTCCCATGACCCGCCGGGCAGCTTCCGGGAAACCAAAGTCTTTGGGTTCCGGGGGGAGTATGGTTGCAAAGCTGAAAC 1191
Cr18SrRNA TCTTTTGATGACTCTGCCAGCACCTTATGAGAAATCAAAGTTTTTGGGTTCCGGGGGGAGTATGGTCGCAAGGCTGAAAC 737
At18SrRNA TGCTTATAGGACTCCGCTGGCACCTTATGAGAAATCAAAGTTTTTGGGTTCCGGGGGGAGTATGGTCGCAAGGCTGAAAC 1222

Hs18SrRNA TTAAAGGAATTGACGGAAGGGCACCACCAGGAGTGGAGCCTGCGGCTTAATTTGACTCAACACGGGAAACCTCACCCGGC 1271
Cr18SrRNA TTAAAGGAATTGACGGAAGGGCACCACCAGGCGTGGAGCCTGCGGCTTAATTTGACTCAACACGGGGAAACTTACCAGGT 817
At18SrRNA TTAAAGGAATTGACGGAAGGGCACCACCAGGAGTGGAGCCTGCGGCTTAATTTGACTCAACACGGGGAAACTTACCAGGT 1302

Hs18SrRNA CCGGACACGGACAGGATTGACAGATTGATAGCTCTTTCTCGATTCCGTGGGTGGTGGTGCATGGCCGTTCTTAGTTGGTG 1351
Cr18SrRNA CCAGACACGGGAAGGATTGACAGATTGAGAGCTCTTTCTTGATTCTGTGGGTGGTGGTGCATGGCCGTTCTTAGTTGGTG 897
At18SrRNA CCAGACATAGTAAGGATTGACAGACTGAGAGCTCTTTCTTGATTCTATGGGTGGTGGTGCATGGCCGTTCTTAGTTGGTG 1382

Hs18SrRNA GAGCGATTTGTCTGGTTAATTCCGATAACGAACGAGACTCTGGCATGCTAACTAGTTACGCGACCCCCGAGCGGTCGGCG 1431
```

```
Cr18SrRNA GGTTGCCTTGTCAGGTTGATTCCGGTAACAAACGAGACCTCAGCCTGCTAAATAGTCAGCATCGCA--CC-----TGCGG 970
At18SrRNA GAGCGATTTGTCTGGTTAATTCCGTTAACGAACGAGACCTCAGCCTGCTAACTAGCTACGTGGAGGCATC-----CCTTC 1457

Hs18SrRNA TCCCCCAACTTCTTAGAGGGACAAGTGGCGTTCAGCCA--CCCGAGATTGAGCAATAACAGGTCTGTGATGCCCTTAGAT 1509
Cr18SrRNA TGCGCCGACTTCTTAGAGGGACTATTGGCGTTTAGCCAATGGAAGTATGAGGCGATAACAGGTCTGTGATGCCCTTAGAT 1050
At18SrRNA ACGGCCGGCTTCTTAGAGGGACTATGGCCGTTTAGGCCAAGGAAGTTTGAGGCAATAACAGGTCTGTGATGCCCTTAGAT 1537

Hs18SrRNA GTCCGGGGCTGCACGCGCGCTACACTGACTGGCTCAGCGTGTGCCTACCCTACGCCGGCAGGCGCGGGTAACCCGTTGAA 1589
Cr18SrRNA GTTCTGGGCCGCACGCGCGCTACACTGACGCGACCAACGAGCCTATCCTTGGC--CGAGAG-GCCCGGGTAATCTTGTAA 1127
At18SrRNA GTTCTGGGCCGCACGCGCGCTACACTGATGTATTCAACGAGTTCACACCTTGC--CGACAG-GCCCGGGTAATCTTTGAA 1614

Hs18SrRNA CCCCATTCGTGATGGGGATCGGGGATTGCAATTATTCCCCATGAACGAGGAATTCCCAGTAAGTGCGGGTCATAAGCTTG 1669
Cr18SrRNA ACCGCGTCGTGATGGGGATAGATTATTGCAATTATTAGTCTTCAACGAGGAATGCCTAGTAAGCGCGAGTCATCANCTCG 1207
At18SrRNA ATTTCATCGTGATGGGGATAGATCATTGCAATTGTTGGTCTTCAACGAGGAATTCCTAGTAAGCGCGAGTCATCAGCTCG 1694

Hs18SrRNA CGTTGATTAAGTCCCTGCCCTTTGTACACACCGCCCGTCGCTACTACCGATTGGATGGTTTAGTGAGGCCCTCGGATCGG 1749
Cr18SrRNA CGTTGATTACNTCCCTGCCCTTTGTACACACCGCCCGTCGCTCCTACCGATTGGGTGTGCTGGTGAAGTGTTCGGATTG- 1286
At18SrRNA CGTTGACTACGTCCCTGCCCTTTGTACACACCGCCCGTCGCTCCTACCGATTGAATGATCCGGTGAAGTGTTCGGATCGC 1774

Hs18SrRNA CCCCGCCGGGGTCGGCCCACGGCCCTGGCGGAGCGCTGAGAAGACGGTCGAACTTGACTATCTAGAGGAAGTAAAAGTCG 1829
Cr18SrRNA -------------------------------------------------------------------------------- 1286
At18SrRNA GGCGACGTGGGTGGTTCGCCGCCCGCGA---CGTCGCGAGAAGTCCACTAAACCTTATCATTTAGAGGAAGGAGAAGTCG 1851

Hs18SrRNA TAACAAGGTTTCCGTAGGTGAACCTGCGGAAGGATCATTA----------- 1869
Cr18SrRNA --------------------------------------------------- 1286
At18SrRNA TAACAAGGTTTCCGTAGGTGAACCTGCGGAAGGATCATTGTCGATACCTGT 1902
```
