# Supplementary material for: Overexpression of 18S rRNA methyltransferase CrBUD23 enhances biomass and lutein content in Chlamydomonas reinhardtii
Source: Front Bioeng Biotechnol. 2023 Feb 3;11:1102098. doi: 10.3389/fbioe.2023.1102098 (PMC9935685; doi:10.3389/fbioe.2023.1102098)
Supplement: Supplementary file 2 [file DataSheet4.PDF]

Color Align Conservation results

```
ScTrm112       MKFLTTNFLKC-SVKACDTSNDNFPLQYDGSKCQLVQDESIEFNPEFLLNIVDRVDWPAVLTVAAELGNNALPPTKPSFP 79
CeTRM112       MKLFVHNFMSSRFLKNV---TVGYPLNLVVKQ---FVEKDIEFDRDNTIVMLDRIQYEALIVAAAAVNQSDRIPREKPEK 74
DmTRM112       MKLSTYNFLTSVAIKGV---KVGFPLKLTINK---KEVVESEFNPTFVERILPKLDWSAVYGAAQVAELTEDIPAVQPEN 74
DrTRM112       MKLLTHNMLTS-HVKGV---TKGYPLIIKATE---VKVNELDFNAQFVSRMIPKLEWPALVQAAEWLGQSQELPDTLIPD 73
HsTRMT112      MKLLTHNLLSS-HVRGVG--SRGFPLRLQATE---VRICPVEFNPNFVARMIPKVEWSAFLEAAD------NVPKGPVEG 68
MmTRM112       MKLLTHNLLSS-HVRGVG--TRGFPLRLQATE---VRINPVEFNPEFVARMIPKVEWAALVQAADTLNL-AEVPKEPTEG 73
BbTrm112       MKLLTHNFLTC-KIKGV---QNGYPFKIVADK---VETVTTDFDSDFLKRIFDRVNYQVLKEAAESLGESEGLPEQA-TL 72
DsTrm112       MKLLTHNMLAC-HIKGI---TNNYPLLIEATK---IETRDADFNPDFLRHIFPRIQWPAFLQGAETLGCRGSLPDDPVPE 73
CrTrm112       MKLLTHNMLSC-HIKNV---RNGYPFLIEVVK---VSEHEADFDPDFLKHIFPRINWPAFLQGAQSLGCREGLPEEA-AE 72
VcTrm112       MKLLTHNMLSC-HIKGV---RSGYPFLIEVVK---VSEHEADFDTDFLKHIFPRINWPAFLQGAEAMGCREGLPEAP-NE 72
CzTrm112       MKLLTHNMLSC-HIKGV---QNGFPFKIQASK---IEEREADYDPDFLRHIFPKIEWKAFLEGARALHCADGLPEEV-TD 72
CsTrm112       MKLLTHNMLSC-HIKGV---QNGFPFKIEPVK---VEQVDADYDPDFLRHIYPRLEWKALCEAAAT-MGAPGLPEEV-SE 71
Os07g43020     MRLLTHNMLAS-NARGA---VTGYPLKLQVVK---WSTKEAEPNPEFLRGMLPKIDWPALVAATQALGLPELLPEAPPTD 73
Zm00001d006904 MRLLTHNMLAS-NVRGA---TTGYPLTLEATN---WCTKEVELNSDFIRGLLPKIDWRALVAATRAVGLPELLPEEQPPE 73
MpTrm112       MRLLTHNLLAS-NVKGT---TAGFPLKLEVLV---KEERSTEFDAAFLLHTLPKLNWSAFRAAAESLGVDKLPSTYPER- 72
OlTrm112       MRLLAHNMLAC-NAKGV---VNGFPLKIVPKE---TREVEVDFNAEFLTHMLPKMEWSAFVNAAKEIGLEGLPSEIPDD- 72
Pp3c10_8220    MRLLTHNMLAC-NIKGV---TKGFPLGIEHTR---LETKESELNADFLRHIFPKLDWKAFHEAAQSVGVNNIPDQVEP-- 71
Mt1g050345     MGLLTHNMLSS-NIRGV---VNGFPLRIEAVK---VVEKNVEMNTDFLKNMFEKIDWKAFVEASISMGYTELPKEADSS- 72
Mt2g028730     MRLLTHNMLSS-NIKGV---VNGFPLRIEAEK---VVEKNVEMNGDFLKKMFEKIEWKAFVEASRGMGYTELPEEADSS- 72
At1g22270      MRLITHNMLSC-NIKGV---TSGFPLRIEAGN---VIEKEVDFNPDFIRHMFAKIEWKALVEGARSMGYAELPEESPDAA 73
At1g78190      MRLIVHNMLSC-NIKGV---VNKFPLRIEAEK---VTVKEVDFNPDFLRYMFAKIDWKALVDGARSMEYTELPDNAPDTT 73

ScTrm112       SSI--QELTDDDMAILNDLHTLLLQTSIAEGEMKCRNCGHIYYIKNGIPNLLLPPHLV--- 135
CeTRM112       -------WDELTDEQLRVFHHLLMNIDVIDGELICPETKTVFPIRDGIPNMLKVDAEK--- 125
DmTRM112       --------IVENELLLQKLHHLLFEIDVLEGQLECPETGRVFPISDGIPNMLLNEDEV--- 124
DrTRM112       --------YENDEEFLRKVHRVLLEVEVIEGCLQCPESGREFPISKGVPNMLLNEGE---- 122
HsTRMT112      --------YEENEEFLRTMHHLLLEVEVIEGTLQCPESGRMFPISRGIPNMLLSEEETES- 120
MmTRM112       --------YEHDETFLRKMHHVLLEVDVLEGTLQCPESGRLFPISRGIPNMLLNDEETET- 125
BbTrm112       -------EALEDEAFQKAYHHALLEVVVKEGALVCPETGRRFIVKKGIPNLLLNEDEIA*- 125
DsTrm112       -------GSLEDETFLKQFHHALLEVVLEEGFLICPETQRRFPVCKGIPNLLLNEDEC*-- 125
CrTrm112       -------SMLEDEGFLKRFHHALLEVFLEEGSLVCPETGRKFPVTKGIPNMLLNEDEC*-- 124
VcTrm112       -------SALEDEQFQKAFHHALLEVTLEEGSLICPETGRKFPVSKGIPNMLLNEDEC*-- 124
CzTrm112       -------EMLQDDGFLQAFHHALLEVHLEEGALICPETGRHFTVAKGIPNLLLNEDEC*-- 124
CsTrm112       -------EMLQDDDFLRSFHHALLELVLEEGALVCPETGRQFPVHKGVPNLLLNEDEN*-- 123
Os07g43020     AELSAEGAAADEGSALRRLHRALLEIHIEEGALVCPDTDRCFPISRGVPNMLLHEDEVRN* 134
Zm00001d006904 EEIFADGAADVEGSAIRRIHHALLEVHVQEGSLVCPDTSRCFPINKGIPNMMLHEDEV*-- 132
MpTrm112       --------DELTNEFLRVFHHALLEIEVQEGYLICPETGRRFPVRKGIPNMMLNEDEVD*- 124
OlTrm112       --------AASDEEFLRTFHHALLEVHVEEGTLVCPESGRKFPINKGIPNMLLNEDEV*-- 123
Pp3c10_8220    -------VMLDDDEFLRKFHHALLEVHLEEGALICPETGRRFPVTKGVPNMLLNEDEV*-- 123
Mt1g050345     --------LLDSDDFLNRFHHALLELHLEEGALVCPETRRRFPVSKGIPHMLLHEDEV*-- 123
Mt2g028730     --------MLDSNEFLNRFHHALLELHLEEGALVCPETGRRFPVKKGIPNMLLHEDEV*-- 123
At1g22270      -------VLKSDEPFLKKLHHALLELHLEEGALVCPETGRKFPVNKGIPNMLLHEDEV*-- 125
At1g78190      -------TLESDETFLRKFHHALLELHLEEGSLVCPETGRKFSVSKGIPNMLLHEDEV*-- 125
```
