# Supplementary material for: Overexpression of 18S rRNA methyltransferase CrBUD23 enhances biomass and lutein content in Chlamydomonas reinhardtii
Source: Front Bioeng Biotechnol. 2023 Feb 3;11:1102098. doi: 10.3389/fbioe.2023.1102098 (PMC9935685; doi:10.3389/fbioe.2023.1102098)
Supplement: Supplementary file 3 [file Table1.DOCX]

>HsWBSCR22

MASRGRRPEHGGPPELFYDETEARKYVRNSRMIDIQTRMAGRALELLYLPENKPCYLLDIGCGTGLSGSYLSDEGHYWVGLDISPAMLDEAVDREIEGDLLLGDMGQGIPFKPGTFDGCISISAVQWLCNANKKSENPAKRLYCFFASLFSVLVRGSRAVLQLYPENSEQLELITTQATKAGFSGGMVVDYPNSAKAKKFYLCLFSGPSTFIPEGLSENQDEVEPRESVFTNEREGGAFERRGIRGHQTRRFPLRMSRRGMVRKSRAWVLEKKERHRRQGREVRPDTQYTGRKRKPRF

>ScBUD23

MSRPEELAPPEIFYNDSEAHKYTGSTRVQHIQAKMTLRALELLNLQPCSFILDIGCGSGLSGEILTQEGDHVWCGLDISPSMLATGLSRELEGDLMLQDMGTGIPFRAGSFDAAISISAIQWLCNADTSYNDPKQRLMRFFNTLYAALKKGGKFVAQFYPKNDDQVDDILQSAKVAGFSGGLVVDDPESKKNKKYYLVLSSGAPPQGEEQVNLDGVTMDEENVNLKKQLRQRLKGGKDKESAKSFILRKKELMKRRGRKVAKDSKFTGRKRRHRF

>MmWBSCR22

MASRSRRPEHSGPPELFYDQNEARKYVRNSRMIDIQTKMTERALELLCLPEGQPSYLLDIGCGSGLSGDYISEEGHYWVGIDISPAMLDAALDRDTEGDLLLGDMGQGVPFRPGSFDGCISISAVQWLCNANKKSDVPARRLYCFFSSLYSALVRGARAVLQLYPENSEQLELITTQATRAGFTGGVVVDFPNSAKAKKFYLCLFSGPSTSLPKGLTESQDADQASESMFTSERAPHKKARRDLVKKSREWVLEKKERRRRQGK

>DrWBSCR22

MSSSCRRPEHMAPPEVFYNEEEAKKYSQNSRMIEIQTQMSERAVELLNLPEDQPCYLLDVGCGSGLSGDYLSEAGHYWVGVDISTAMLDVALEREVEGDLLLGDMGEGMPFRPGMFDGCISISALQWLCNADKKTHSPPKRLYRFFSTLYSSLARGARAVFQIYPENSEQLELITAQAMKAGFTGGMVVDYPNSSKAKKFFLCLFAGVSGVLPKGLDSETAVRGVVNQAQFTAQRSRFKNMKGKSAKKSKDWILDKKERRRRQGKDVRADTKYTGRHRKP

KF

>CeBUD23

MASFKVKPEHTGPPDLYYNETEAAKYASNSHITAIQHEMAERALELLALPEGKSGFLLDIGCGTGMSSEVILDAGHMFVGVDVSRPMLEIARQDEDLESGDFIHQDMGLGMPFRPGSFDGAISISAIQWLCHANASDENPRKRLLFFFQSLYGCLGRGSRAVFQFYPENDEQCDLIMGQAHKAGFNGGLVVDFPEAAKRKKVYLVLMTGGVVQLPQALTEDGEESRTQIDNAGRRFVWNSRKNEKVAKGSKAWIEAKRQRQIKQGRDVRHESKYSGRKRK

TKF

>DmBUD23

MARRPEHSAPPEIFYNDDEAKKYSTNTRIIEIQVEMAERALELLALPDDDESRLILDIGCGSGLSGSVLEDSEHMWIGIDISKSMLDIAVEREVAGDVILGDMGEGMPFKPGTFDGAISISALQWLCNADKSYHNPHKRLLKFFTTLFSCLTRTARAVFQFYPENSDQIEMVTSQAMKAGFYGGLVVDYPNSAKAKKYYLVLMTGGSAELPQALGSPEEERRVNYIKKRDACREARGKAPKKSRDWILAKKERRRRQGLETRPDTKYTARKRSGKF

>BbBUD23

MRRFFETLYACLVRGARAVLQIYPEDASQAQLLTSAAMRAGFSGGLVVDFPHSTRAKKYFLVLMVGSTAALPQAKGLHGEASDDEEELEEVPVGARLSGGPHKRRKVKHFVGMGSARHPQAKDKAWVLKKKAQQRERGYPNIPADTKYTGRKRKTRF*

>CrBUD23

MGKGERPEHMAPPDIFYNEDEARKYTTNSRMINIQSTLTERALELLALPQDGLPRLLLDLGCGSGLSGEALSEAGQVWVGLDISAAMLDVAHEREVEGDLVLGDLGHGLPLRPGSFDGAISISAVQWLCNADRAGHDPRKRMKRFFETLYMSLRRGARAVLQIYPENHKQAEMLVAAAMKVGFSGGLVVDYPHSTRAKKYFLVLMVGTSISVPTAKGLDGGEPEDEEEEAAAEHVQVAGRDRNKRRRTGSGKGDGKGREWVLRKKEQMRKKGYDIAPDSKYTARKRKRVV*

>CzBUD23

MGKSERPEHTAPPEIFYNEDEARKYTTNSRMINIQSALTQRALELLALPDDGTPRLLLDLGCGSGLSGEALTEAGHMWIGMDISPAMLDVAIEREVEGDVALHDLGHGLPLRMGTFDGAISISAVQWLCNADKASHEPRKRMKRFFETLYSSLTRGARAVLQIYPENTAQAEMLVSSAIKVGFSGGLVVDYPHSTRAKKYFLVLMVGSSSMVPQPKGLDGIEPESDAEMADADGVQVMGSRHRNKRRKSADSSGGKGKEWLLKKKERMRHKGYEHIPPDTKYSGRKRRRTL*

>CsBUD23

MTRGDRPENAAPPEVFYNEQEARKYTTNSRMMAIQAQLTERALELLALPDDGRMKMLLDLGCGSGLSGEALTEQGHCWVGMDISEAMLDVAREREVEGDLCLHDLGDGLPLRTGAFDGAISISAVQWLCNADRRGADPRRRMRRFFETMYSCLTRGGRAVLQIYPADSSQAEMLTSAAMRAGFSGGLVVDFPHSTRAKKYFLVLMVGGSAAMPAPRGMSGEASDEEEGTVAVAGRQHSSKRRRVGADSARAKGKAWILKKKEQARHKGYVGIPGDTKYTGRKRKTKF*

>DsBUD23

MGKGERPEHQAPPEIFYSETEAAKYTSNSRIMQIQAEGDLALHDLGHGLPFRPGTFDGAISISAVQWLCNADKRCNEPRKRLKRFFETLYACLNRGARAVLQVYPDSPAQAEMMVAAAMKVGFSGGLVVDYPHSTRAKKFFLVLMAKGLDGQDPEDSDEGAQHVKVSGRERKHAGKRKHAGKPGKAPTGSKEWIVHKKDTMRKRGYTEIPRDTKYTGRKRKKTV*

>MpBUD23

MSRPEFTAPPQIFYNDVEARKYTHSSRVVEIQERLTERAVELLNIPDDGVPRLLLDVGCGSGLSGERLTSLGHEWIGTDISMNMLEVAQEREVLGGVVQYDMGHGCPFRPGVFDGCISISAIQWLCNADNSLHRPRRRLASFFNHLYRCLKRGSKAVLQFYPDNAEQVEMITTSALRVGFSGGLVVDYPNSTRAKKYFLVLAAGSESSATEFSASTHICGLVGDELLNKPGINIVGRKKARKNLSPSRKISSVSHSRSKRHPDNKGRAWIEKKKSQAQLKGKETARNSKYTGRKRKDRI*

>OlBUD23

MSRPELTAPADVFYNDTEARKYSQSSRVVEIQERLTERAVELLNFPDDGVPRLLLDVGCGSGLSGDRLTELGHEWIGMDLSASMLEVAKEREVEGDVLRNDMGHGVPFRPGVFDGCVSISAVQWLCNADNSAHVPQRRLKTFFTQLYKSLKRGAKAILQIYPDGPRQAEMITTAALRVGFSGGLVVDYPNSTRAKKYFLALAAGPPEQLPTPKGEFDDDDDIERRGMRMDGRKSDRSGKYKKGKNIKGKAWVHKKKEQYRNRGVQVASDSKFTGRKRKDRL*

>VcBUD23

MGKGERPEHMAPPDIFYNADEARKYTTNSRMIAIQSSLTERALELLALPQDGIPRLLLDLGCGSGLSGETLSEAGHMWLGVDISEAMLDVAVEREIEGDLVLGDLGHGLPLRPGAFDGAISISAVQWLCNADRTGHDPRKRMKRFFETLYMSLRRGARAVLQIYPENPQQAEMLVAAAMKVGFSGGLVVDYPHSTRAKKYFLVLMVGTSAATPQAKGLDGSEPEDEEEEAQVKVAGRDRHKRRKTAGGGSGSGTKGREWILKKKEQMRKKGYDIAADSKYTGRKRKRLV*

>AtRID2

MSNRPELLAPPEIFYDDTEARKYTSSSRIVEIQAKLSERALELLALPEDGVPRFLLDIGCGSGLSGETLSEDGHHWIGLDISASMLHVAVEREVEGDLLLGDMGQGLGLRSGVIDGAISISAVQWLCNADKSSHEPRLRLKAFFGSLYRCLSRGARAVFQVYPENIAQRELILRQALQAGFGGGLVVDYPHSTKKRKEFLVLTCGTVQTSIQTSKNEYDESCSEDDNSDDEESEEVGVSDRNRPRKRQRTNTKVKGREWVLRKKEQSRRKGKNVPADSKFTSRKRRTRF*

>MtBUD23a

MGSRPEAVAPPEIFYDDDTARKYTSNSRNIQIQTSMTERALELLNLPKDGVPKLLLDIGCGSGLSGEVITESGHHWVGLDIAPSMLDIALDREVEGDLLLGDMGQGLGLRFGMFDGAIGISTIQWLCNADKSFHNPHLRLKAFFTSLYKCLTNGARAVFQVYPENDDQRELLSSPAMKAGFSGGIVVDYKDSPKKRKEYLFLVCGQEVPLPLPEGRTEDNDDSGSETNKTVHVLDRRRPWKIQKNNKSEKGREWIKRKKEQMRRRGDDVPPDTKYTGRKRKNHF*

>MtBUD23b

MIPKLASILLLPVLSIFRPHSQNEHWSYLLYLKTVFPNYSLTLVLYFNFNSFSHFFFHFQLSCLIFVGCGSGLSGETLSEEGHHWIGLDISPSMLNIALEREVEGDLLLSDMGQGLGLRPGVIDGAISISAVQWLCNADRSSHNPRLRLKAFFTSLYRCLANGAKAVFQVYPENVDQRELILNAAMHAGFSGGIVVDFPHSSKKRKEFLVLGCGQLSTKASLSKGKIEDEEKLSDEESEDEENQTVRLSDRHRPVKKQRKNNKSGKGKEWILRKKDQMRRRGNDVPLDTKYTGRKRKGRF*

>OsBUD23

MVTHGGGGGGGARTIWDDEAAAAAGVMRRSPRCVGEFIGDPLLGRDIVLGLWDSRPISRVSLHRRPMKSPVERRSVVGTLGESRREHTTDLCGGGEERRGEKPRRRQRGERMPRPEVQAPPEIFYNESEARKYTTSSRIIEIQSRITERALELLALPNDGVPKLLLDIGCGSGLSGETLTEQGHHWIGYDISKSMLDVALEREAEGDLLLADMGQGLGLRPGVIDGAISISAVQWLCNADKSCHNPRLRLKAFFGSLYRCLARGARAVLQFYADNVKQSEMIVTAAMRAGFAGGVVVDWPHSSKAKKSYLVLTCGPPSLNSSLPKGKGQDGAMCSDDDESDDGSGDEDGAQTVGIYERNRPKKRQKTKKNGKGKAWLLNKKEQLRRRGREVPADTKYTGRKRKSYF*

>PpBUD23

MSIRPERQAPPEIFYNDTEARKYTTSSRIVNIQAKLSERALELLALPDDGVSRLLLDIGCGSGLSGETLSENGHHWIGMDISEAMLDVALERETEGDLLLSDIGQGMPFRPGTLDGAISISAVQWLCNADKSCNNPRLRLKAFFGTLYKCLARGARAVLQIYPESPQQLEMISSAAMKSGFSGGLVVDYPHSTRAKKYFLVLSCGPPSTATALPRAKEGNEMSEDEESGSECDEDGGTTVNVSERQRPSKKQRKDSKKSGKGRSWILKKKEQRRHRGYTNVPDDSKYTGRKRKAHF*

>ZmBUD23

MPRPEFQAPPDVFYNESEARKYTTSSRIIEIQSRISERALELLALPNDGVPKLLLDIGCGSGLSGETLTEHGHHWIGYDISKSMLDVALERETEGDLLLADMGQGLGLRPGVIDGAISISAIQWLCNADKSSHDPRLRLKAFFGSLYRCLARGARAVLQFYADNVKQSEMIVTFAMRAGFAGGVVVDWPHSSKAKKSYLVLTCGPPSVTTSLPKGKGENGEGCSDDDDNESSGEDGDRTVGIYERNRPKKRQKTKKNGKGKDWLLRKKEQMRRRGHDVPADTKYTGRKRKGYF*
