# Supplementary figures and images for: Overexpression of 18S rRNA methyltransferase CrBUD23 enhances biomass and lutein content in Chlamydomonas reinhardtii
Source: Front Bioeng Biotechnol. 2023 Feb 3;11:1102098. doi: 10.3389/fbioe.2023.1102098 (PMC9935685; doi:10.3389/fbioe.2023.1102098)

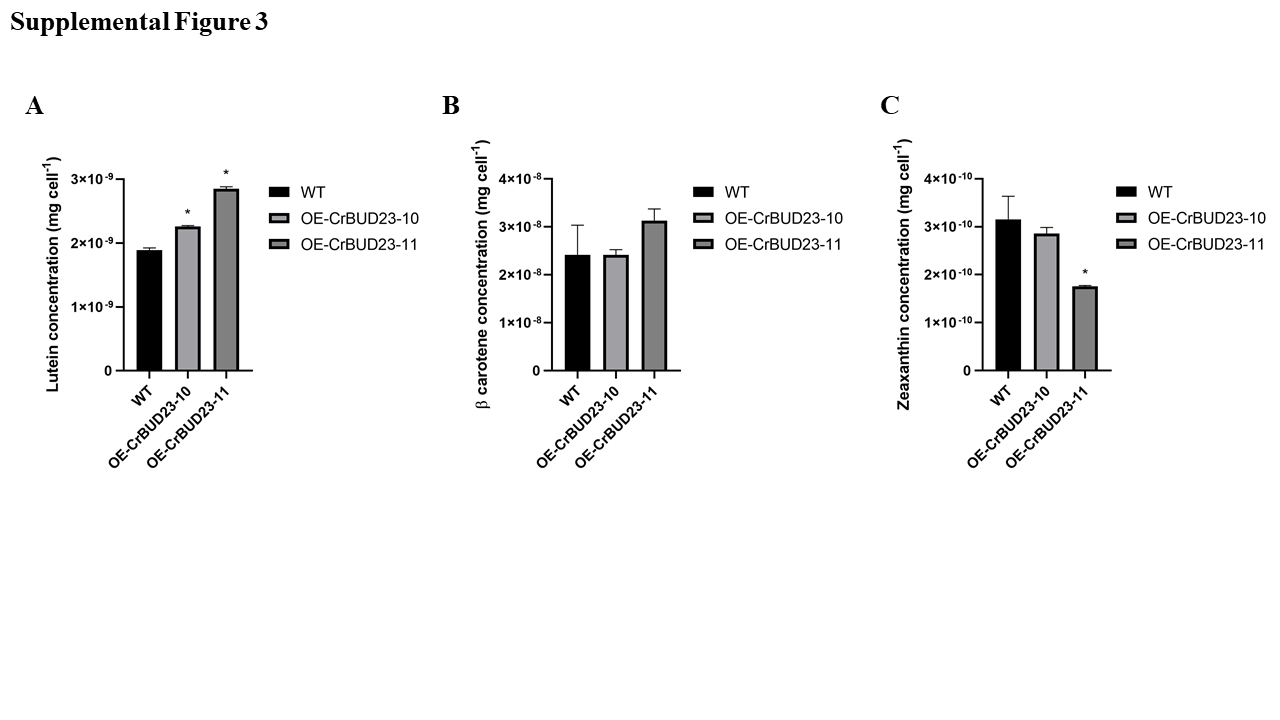

Supplement: Supplementary file 4 [file Image2.TIF]

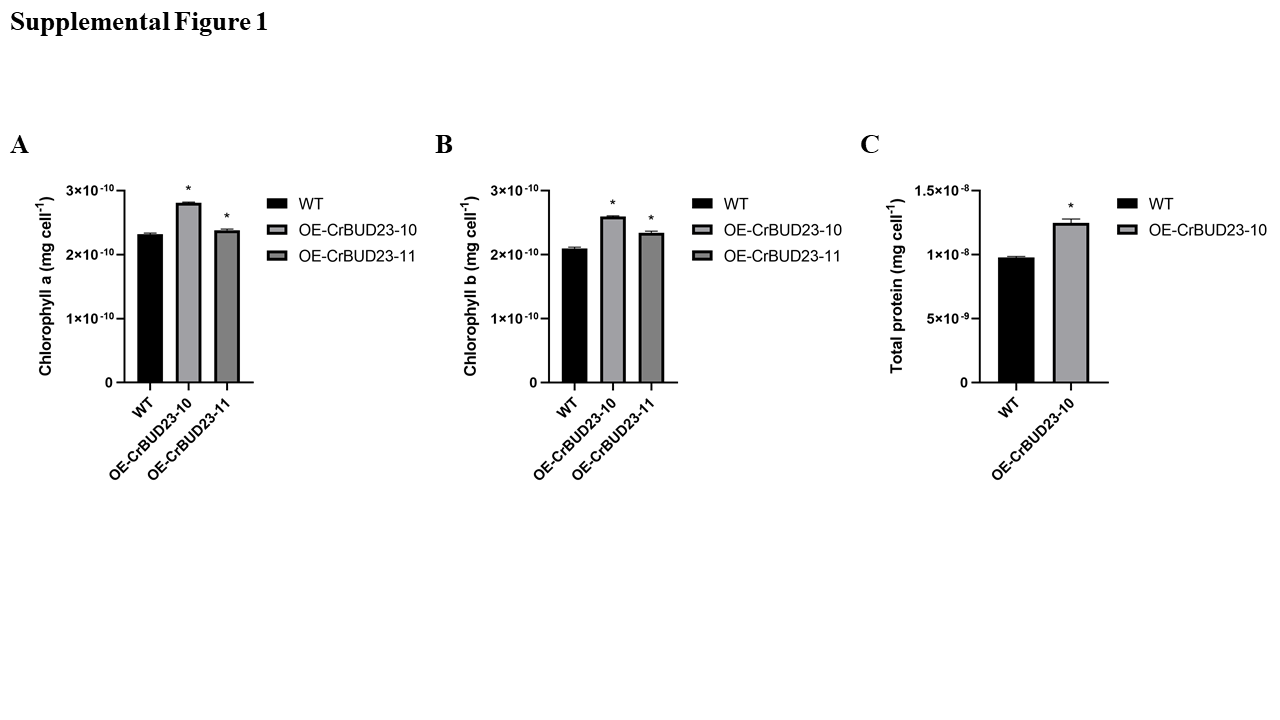

Supplement: Supplementary file 5 [file Image1.TIF]
