# Supplementary material for: Overexpression of 18S rRNA methyltransferase CrBUD23 enhances biomass and lutein content in Chlamydomonas reinhardtii
Source: Front Bioeng Biotechnol. 2023 Feb 3;11:1102098. doi: 10.3389/fbioe.2023.1102098 (PMC9935685; doi:10.3389/fbioe.2023.1102098)
Supplement: Supplementary file 6 [file DataSheet3.PDF]

Color Align Conservation results

```
KX781331.1 P8        --------------------------------------AAGATTAAGCCATGCATGTCTAAGTATAAACTGCTTTATACT 42
KX781328.1 P12       -------------------------------------------------------------------------------- 0
KX781321.1 P20       -------------------------------------------------------------------------------- 0
KX781327.1 P13       -------------------------------------------------------------------------------- 0
KX781337.1 P2        -------------------------------------------------------------------------------- 0
JN903984.1 SAG53.72  --------------------AGTCATATGCTTGTCTCAAAGATTAAGCCATGCATGTCTAAGTATAAACTGCTTTATACT 60
KR904894.1 CC-849    -------------------------------------------------------------------------------- 0
KC149968.1 GTD4C     ACCTGGTTGATCCTGCCAGTAGTCATATGCTTGTCTCAAAGATTAAGCCATGCATGTCTAAGTATAAACTGCTT-ATACT 79
KX781333.1 P6        -------------------------------------------------------------------------------- 0
AY665726.1 CC-1418   -----------------------------------------ATTAAGCCATGCATGTCTAAGTATAAACTGCTT-ATACT 38
AY665727.1 CC-1952   -----------------------------------------------CCATGCATGTCTAAGTATAAACTGCTT-ATACT 32
AB701550.1 NIES-2235 -------------------------------------------------------------------------------- 0
AB701554.1 NIES-2239 -------------------------------------------------------------------------------- 0
AB701553.1 NIES-2238 -------------------------------------------------------------------------------- 0
AB753040.1 PS-2708   -------------------------------------------------------------------------------- 0
AB701552.1 NIES-2237 -------------------------------------------------------------------------------- 0
AB701551.1 NIES-2236 -------------------------------------------------------------------------------- 0
AB701555.1 NIES-2463 -------------------------------------------------------------------------------- 0
KR092109.1 CC-125    -------------------------------------------------------------GTATAAACTGCTT-ATACT 18
KX781322.1 P18       -------------------------------------------------------------------------------- 0
KX781326.1 P14       -------------------------------------------------------------------------------- 0
KX781329.1 P11       -------------------------------------------------------------------------------- 0
KX781330.1 P10       -------------------------------------------------------------------------------- 0
KX781332.1 P7        -------------------------------------------------------------------------------- 0
AB511837.1 KkS0801D2 -----------------------------------------------CCATGCATGTCTAAGTATAAACTGCTT-ATACT 32
AB511836.1 KkS0801B1 -----------------------------------------------CCATGCATGTCTAAGTATAAACTGCTT-ATACT 32
AB511835.1 SAG 11-32 -----------------------------------------------CCATGCATGTCTAAGTATAAACTGCTT-ATACT 32
AB511834.1 UTEX 90   -----------------------------------------------CCATGCATGTCTAAGTATAAACTGCTT-ATACT 32
KC166137.1 K01       -------------------------------------------------------------------------------- 0
KX781338.1 P1        -------------------------------------------------------------------------------- 0
KF864473.1 JinCheon1 -CCTGGTTGATCCTGCCAGTAGTCATATGCTTGTCTCAAAGATTAAGCCATGCATGTCTAAGTATAAACTGCTT-ATACT 78
EU925397.1 CC-124    -------------------------------------------------------------------------------- 0
JN903974.1 SAG18.79  --------------------AGTCATATGC-TGTCTCAAAGATTAAGCCATGCATGTCTAAGTATAAACTGCTT-ATACT 58
JN903978.1 SAG11-32c --------------------AGTCATATGCTTGTCTCAAAGATTAAGCCATGCATGTCTAAGTATAAACTGCTT-ATACT 59
KX781325.1 P15       -------------------------------------------------------------------------------- 0
JN863299.1 KNUA021   ------------------GTAGTCATATGCTTGTCTCAAAGATTAAGCCATGCATGTCTAAGTATAAACTGCTT-ATACT 61
KR904893.1 CBS152280 -------------------------------------------------------------------------------- 0
KX781335.1 P4        -------------------------------------------------------------------------------- 0
JX888472.1 CC-621    ------------------GTAGTCATATGCTTGTCTCAAAGATTAAGCCATGCATGTCTAAGTATAAACTGCTT-ATACT 61
KC310450.1 RAC       -------------------------------------------------------------------------------- 0
JX888471.1 CC-620    ------------------GTAGTCATATGCTTGTCTCAAAGATTAAGCCATGCATGTCTAAGTATAAACTGCTT-ATACT 61

KX781331.1 P8        GTGAAACTGCGAATGGCTCATTAAATCAGTTATAGTTTATTTGATGGTACCTACTACTCGGATAACCGTAGTAATTCTAG 122
KX781328.1 P12       -------------------------------------------------------------------------------- 0
KX781321.1 P20       ----------GAATGGCTCATTAAATCAGTTATAGTTTATTTGATGGTACCTACTACTCGGATAACCGTAGTAATTCTAG 70
KX781327.1 P13       -------------------------------------------------------------------------------- 0
KX781337.1 P2        -------------------------------------------------------------------------------- 0
JN903984.1 SAG53.72  GTGAAACTGCGAATGGCTCATTAAATCAGTTATAGTTTATTTGATGGTACCTACTACTCGGATAACCGTAGTAATTCTAG 140
KR904894.1 CC-849    -------------------------------------------------------------------------------- 0
KC149968.1 GTD4C     GTGAAACTGCGAATGGCTCATTAAATCAGTTATAGTTTATTTGATGGTACCTACTACTCGGATAACCGTAGTAATTCTAG 159
KX781333.1 P6        -------------------------------------------------------------------------------G 1
AY665726.1 CC-1418   GTGAAACTGCGAATGGCTCATTAAATCAGTTATAGTTTATTTGATGGTACCTACTACTCGGATAACCGTAGTAATTCTAG 118
AY665727.1 CC-1952   GTGAAACTGCGAATGGCTCATTAAATCAGTTATAGTTTATTTGATGGTACCTACTACTCGGATAACCGTAGTAATTCTAG 112
AB701550.1 NIES-2235 ----CCTTGCGAATGGCTCATTATATCAGTTATAGTTTATTTGATGGTACCTACTACTCGGATAACCGTAGTAATTCTAG 76
AB701554.1 NIES-2239 ----------------------ATATCAGTTATAGTTTATTTGATGGTACCTACTACTCGGATAACCGTAGTAATTCTAG 58
AB701553.1 NIES-2238 ----------GAATGGCTCATTATATCAGTTATAGTTTATTTGATGGTACCTACTACTCGGATAACCGTAGTAATTCTAG 70
AB753040.1 PS-2708   ----------------------ATATCAGTTATAGTTTATTTGATGGTACCTACTACTCGGATAACCGTAGTAATTCTAG 58
AB701552.1 NIES-2237 -----------AATGGCTCATTATATCAGTTATAGTTTATTTGATGGTACCTACTACTCGGATAACCGTAGTAATTCTAG 69
AB701551.1 NIES-2236 -------------------AATATATCAGTTATAGTTTATTTGATGGTACCTACTACTCGGATAACCGTAGTAATTCTAG 61
AB701555.1 NIES-2463 --------GCGAATGGCTCATTATATCAGTTATAGTTTATTTGATGGTACCTACTACTCGGATAACCGTAGTAATTCTAG 72
KR092109.1 CC-125    GTGAAACTGCGAATGGCTCATTAAATCAGTTATAGTTTATTTGATGGTACCTACTACTCGGATAACCGTAGTAATTCTAG 98
KX781322.1 P18       -------------------------------------------------------------------------------- 0
KX781326.1 P14       -----------AATGGCTCATTATATCAGTTATAGTTTATTTGATGGTACCTACTACTCGGATAACCGTAGTAATTCTAG 69
KX781329.1 P11       -------------------------------------------------------------------------------- 0
KX781330.1 P10       -------------------------------------------------------------------------------- 0
KX781332.1 P7        -------------------------------------------------------------------------------- 0
AB511837.1 KkS0801D2 GTGAAACTGCGAATGGCTCATTAAATCAGTTATAGTTTATTTGATGGTACCTACTACTCGGATAACCGTAGTAATTCTAG 112
AB511836.1 KkS0801B1 GTGAAACTGCGAATGGCTCATTAAATCAGTTATAGTTTATTTGATGGTACCTACTACTCGGATAACCGTAGTAATTCTAG 112
AB511835.1 SAG 11-32 GTGAAACTGCGAATGGCTCATTAAATCAGTTATAGTTTATTTGATGGTACCTACTACTCGGATAACCGTAGTAATTCTAG 112
AB511834.1 UTEX 90   GTGAAACTGCGAATGGCTCATTAAATCAGTTATAGTTTATTTGATGGTACCTACTACTCGGATAACCGTAGTAATTCTAG 112
KC166137.1 K01       -------------------------------------------------------------------------------- 0
KX781338.1 P1        -------------------------------------------------------------------------------- 0
KF864473.1 JinCheon1 GTGAAACTGCGAATGGCTCATTAAATCAGTTATAGTTTATTTGATGGTACCTACTACTCGGATAACCGTAGTAATTCTAG 158
EU925397.1 CC-124    -------------------------------------------------------------------------------- 0
JN903974.1 SAG18.79  GTGAAACTGCGAATGGCTCATTAAATCAGTTATAGTTTATTTGATGGTACCTACTACTCGGATAACCGTAGTAATTCTAG 138
JN903978.1 SAG11-32c GTGAAACTGCGAATGGCTCATTAAATCAGTTATAGTTTATTTGATGGTACCTACTACTCGGATAACCGTAGTAATTCTAG 139
KX781325.1 P15       ---------CGAATGGCTCATTAAATCAGTTATAGTTTATTTGATGGTACCTACTACTCGGATAACCGTAGTAATTCTAG 71
JN863299.1 KNUA021   GTGAAACTGCGAATGGCTCATTAAATCAGTTATAGTTTATTTGATGGTACCTACTACTCGGATAACCGTAGTAATTCTAG 141
KR904893.1 CBS152280 -------------------------------------------------------------------------------- 0
KX781335.1 P4        ---------CGAATGGCTCATTAAATCAGTTATAGTTTATTTGATGGTACCTACTACTCGGATAACCGTAGTAATTCTAG 71
JX888472.1 CC-621    GTGAAACTGCGAATGGCTCATTAAATCAGTTATAGTTTATTTGATGGTACCTACTACTCGGATAACCGTAGTAATTCTAG 141
KC310450.1 RAC       -------------------------------------------------------------------------------- 0
JX888471.1 CC-620    GTGAAACTGCGAATGGCTCATTAAATCAGTTATAGTTTATTTGATGGTACCTACTACTCGGATAACCGTAGTAATTCTAG 141

KX781331.1 P8        AGCTAATACGTGCGTAAATCCCGACTTCTGGAAGGGACGTATTTATTAGATAAAAGGCCAGCCGGGCTTTGCCCGACCTG 202
KX781328.1 P12       ----------------------------------------------------------------------GCCCGACCTG 10
KX781321.1 P20       AGCTAATACGTGCGTAAATCCCGACTTCTGGAAGGGACGTATTTATTAGATAAAAGGCCAGCCGGGCTTTGCCCGACCTG 150
KX781327.1 P13       ---------------------------------------------------------------------TGCCCGACCTG 11
KX781337.1 P2        -------------------------------------------------------------------------------- 0
JN903984.1 SAG53.72  AGCTAATACGTGCGTAAATCCCGACTTCTGGAAGGGACGTATTTATTAGATAAAAGGCCAGCCGGGCTTTGCCCGACCTG 220
KR904894.1 CC-849    -------------------------------------------------------------------------------- 0
KC149968.1 GTD4C     AGCTAATACGTGCGCACAACCCGACTTCTGGAAGGGTCGTATTTATTAGATAAAAGGCCAGCCGGGCTCTGCCCGACCTG 239
```

```
KX781333.1 P6        AGCTAATACGTGCGCACAACCCGACTTCTGGAAGGGTCGTATTTATTAGATAAAAGGCCAGCCGGGCTCTGCCCGACCTG 81
AY665726.1 CC-1418   AGCTAATACGTGCGCACAACCCGACTTCTGGAAGGGTCGTATTTATTAGATAAAAGGCCAGCCGGGCTCTGCCCGACCTG 198
AY665727.1 CC-1952   AGCTAATACGTGCGCACAACCCGACTTCTGGAAGGGTCGTATTTATTAGATAAAAGGCCAGCCGGGCTCTGCCCGACCTG 192
AB701550.1 NIES-2235 AGCTAATACGTGCGCACAACCCGACTTCTGGAAGGGTCGTATTTATTAGATAAAAGGCCAGCCGGGCTCTGCCCGACCTG 156
AB701554.1 NIES-2239 AGCTAATACGTGCGCACAACCCGACTTCTGGAAGGGTCGTATTTATTAGATAAAAGGCCAGCCGGGCTCTGCCCGACCTG 138
AB701553.1 NIES-2238 AGCTAATACGTGCGCACAACCCGACTTCTGGAAGGGTCGTATTTATTAGATAAAAGGCCAGCCGGGCTCTGCCCGACCTG 150
AB753040.1 PS-2708   AGCTAATACGTGCGCACAACCCGACTTCTGGAAGGGTCGTATTTATTAGATAAAAGGCCAGCCGGGCTCTGCCCGACCTG 138
AB701552.1 NIES-2237 AGCTAATACGTGCGCACAACCCGACTTCTGGAAGGGTCGTATTTATTAGATAAAAGGCCAGCCGGGCTCTGCCCGACCTG 149
AB701551.1 NIES-2236 AGCTAATACGTGCGCACAACCCGACTTCTGGAAGGGTCGTATTTATTAGATAAAAGGCCAGCCGGGCTCTGCCCGACCTG 141
AB701555.1 NIES-2463 AGCTAATACGTGCGCACAACCCGACTTCTGGAAGGGTCGTATTTATTAGATAAAAGGCCAGCCGGGCTCTGCCCGACCTG 152
KR092109.1 CC-125    AGCTAATACGTGCGCACAACCCGACTTCTGGAAGGGTCGTATTTATTAGATAAAAGGCCAGCCGGGCTCTGCCCGACCTG 178
KX781322.1 P18       ------------------------------------------------------------------------CCGACCTG 8
KX781326.1 P14       AGCTAATACGTGCGCACAACCCGACTTCTGGAAGGGTCGTATTTATTAGATAAAAGGCCAGCCGGGCTCTGCCCGACCTG 149
KX781329.1 P11       -------------------------------------------------------------------------------- 0
KX781330.1 P10       ------------------------------------------TTATTAGATAAAAGGCCAGCCGGGCTCTGCCCGACCTG 38
KX781332.1 P7        -------------------------------------------------------------------------------- 0
AB511837.1 KkS0801D2 AGCTAATACGTGCGCACAACCCGACTTCTGGAAGGGTCGTATTTATTAGATAAAAGGCCAGCCGGGCTCTGCCCGACCTG 192
AB511836.1 KkS0801B1 AGCTAATACGTGCGCACAACCCGACTTCTGGAAGGGTCGTATTTATTAGATAAAAGGCCAGCCGGGCTCTGCCCGACCTG 192
AB511835.1 SAG 11-32 AGCTAATACGTGCGCACAACCCGACTTCTGGAAGGGTCGTATTTATTAGATAAAAGGCCAGCCGGGCTCTGCCCGACCTG 192
AB511834.1 UTEX 90   AGCTAATACGTGCGCACAACCCGACTTCTGGAAGGGTCGTATTTATTAGATAAAAGGCCAGCCGGGCTCTGCCCGACCTG 192
KC166137.1 K01       ---------------------------------------------------------GCAGNCGGGCTCTGCCCGACCTG 23
KX781338.1 P1        -------------------------------------------------------------------------------- 0
KF864473.1 JinCheon1 AGCTAATACGTGCGCACAACCCGACTTCTGGAAGGGTCGTATTTATTAGATAAAAGGTCAGCCGGGCTCTGCCCGACCTG 238
EU925397.1 CC-124    -------------------------------------------------------------------------------- 0
JN903974.1 SAG18.79  AGCTAATACGTGCGCACAACCCGACTTCTGGAAGGGTCGTATTTATTAGATAAAAGGCCAGCCGGGCTCTGCCCGACCTG 218
JN903978.1 SAG11-32c AGCTAATACGTGCGCACAACCCGACTTCTGGAAGGGTCGTATTTATTAGATAAAAGGCCAGCCGGGCTCTGCCCGACCTG 219
KX781325.1 P15       AGCTAATACGTGCGCACAACCCGACTTCTGGAAGGGTCGTATTTATTAGATAAAAGGCCAGCCGGGCTCTGCCCGACCTG 151
JN863299.1 KNUA021   AGCTAATACGTGCGCACAACCCGACTTCTGGAAGGGTCGTATTTATTAGATAAAAGGCCAGCCGGGCTCTGCCCGACCTG 221
KR904893.1 CBS152280 -------------------------------------------------------------------------------- 0
KX781335.1 P4        AGCTAATACGTGCGCACAACCCGACTTCTGGAAGGGTCGTATTTATTAGATAAAAGGCCAGCCGGGCTCTGCCCGACCTG 151
JX888472.1 CC-621    AGCTAATACGTGCGCACAACCCGACTTCTGGAAGGGTCGTATTTATTAGATAAAAGGCCAGCCGGGCTCTGCCCGACCTG 221
KC310450.1 RAC       -------------------------------------------------------------------------------- 0
JX888471.1 CC-620    AGCTAATACGTGCGCACAACCCGACTTCTGGAAGGGTCGTATTTATTAGATAAAAGGCCAGCCGGGCTCTGCCCGACCTG 221

KX781331.1 P8        CGGTGAATCATGATAACTTCACGAATCGCATGGCCTTGCGCCGGCGATGTTTCATTCAAATTTCTGCCCTATCAACTTTC 282
KX781328.1 P12       CGGTGAATCATGATAACTTCACGAATCGTATGGGCTCGTCCCGACGATGTTTCATTCAAATTTCTGCCCTATCAACTTTC 90
KX781321.1 P20       CGGTGAATCATGATAACTTCACGAATCGCATGGCCTTGCGCCGGCGATGTTTCATTCAAATTTCTGCCCTATCAACTTTC 230
KX781327.1 P13       CGGTGAATCATGATAACTTCACGAATCGTATGGGCTCGTCCCGACGATGTTTCATTCAAATTTCTGCCCTATCAACTTTC 91
KX781337.1 P2        -------------------------------------------------------------------------------- 0
JN903984.1 SAG53.72  CGGTGAATCATGATAACTTCACGAATCGCATGGCCTTGCGCCGGCGATGTTTCATTCAAATTTCTGCCCTATCAACTTTC 300
KR904894.1 CC-849    -------------------------------------------------------------------------------- 0
KC149968.1 GTD4C     CGGTGAATCATGATAACTTCACGAATCGTATGGCCTTGTGCCGACGATGTTTCATTCAAATTTCTGCCCTATCAACTTTC 319
KX781333.1 P6        CGGTGAATCATGATAACTTCACGAATCGTATGGGCTCGTCCCGACGATGTTTCATTCAAATTTCTGCCCTATCAACTTTC 161
AY665726.1 CC-1418   CGGTGAATCATGATAACTTCACGAATCGTATGGGCTCGTCCCGACGATGTTTCATTCAAATTTCTGCCCTATCAACTTTC 278
AY665727.1 CC-1952   CGGTGAATCATGATAACTTCACGAATCGTATGGGCTCGTCCCGACGATGTTTCATTCAAATTTCTGCCCTATCAACTTTC 272
AB701550.1 NIES-2235 CGGTGAATCATGATAACTTCACGAATCGTATGGGCTCGTCCCGACGATGTTTCATTCAAATTTCTGCCCTATCAACTTTC 236
AB701554.1 NIES-2239 CGGTGAATCATGATAACTTCACGAATCGTATGGGCTCGTCCCGACGATGTTTCATTCAAATTTCTGCCCTATCAACTTTC 218
AB701553.1 NIES-2238 CGGTGAATCATGATAACTTCACGAATCGTATGGGCTCGTCCCGACGATGTTTCATTCAAATTTCTGCCCTATCAACTTTC 230
AB753040.1 PS-2708   CGGTGAATCATGATAACTTCACGAATCGTATGGGCTCGTCCCGACGATGTTTCATTCAAATTTCTGCCCTATCAACTTTC 218
AB701552.1 NIES-2237 CGGTGAATCATGATAACTTCACGAATCGTATGGGCTCGTCCCGACGATGTTTCATTCAAATTTCTGCCCTATCAACTTTC 229
AB701551.1 NIES-2236 CGGTGAATCATGATAACTTCACGAATCGTATGGGCTCGTCCCGACGATGTTTCATTCAAATTTCTGCCCTATCAACTTTC 221
AB701555.1 NIES-2463 CGGTGAATCATGATAACTTCACGAATCGTATGGGCTCGTCCCGACGATGTTTCATTCAAATTTCTGCCCTATCAACTTTC 232
KR092109.1 CC-125    CGGTGAATCATGATAACTTCACGAATCGTATGGGCTCGTCCCGACGATGTTTCATTCAAATTTCTGCCCTATCAACTTTC 258
KX781322.1 P18       CGGTGAATCATGATAACTTCACGAATCGTATGGGCTCGTCCCGACGATGTTTCATTCAAATTTCTGCCCTATCAACTTTC 88
KX781326.1 P14       CGGTGAATCATGATAACTTCACGAATCGTATGGGCTCGTCCCGACGATGTTTCATTCAAATTTCTGCCCTATCAACTTTC 229
KX781329.1 P11       ------------------------------------------------------------TTTCTGCCCTATCAACTTTC 20
KX781330.1 P10       CGGTGAATCATGATAACTTCACGAATCGTATGGGCTCGTCCCGACGATGTTTCATTCAAATTTCTGCCCTATCAACTTTC 118
KX781332.1 P7        -------------------------------------------------------------------------------- 0
AB511837.1 KkS0801D2 CGGTGAATCATGATAACTTCACGAATCGTATGGGCTCGTCCCGACGATGTTTCATTCAAATTTCTGCCCTATCAACTTTC 272
AB511836.1 KkS0801B1 CGGTGAATCATGATAACTTCACGAATCGTATGGGCTCGTCCCGACGATGTTTCATTCAAATTTCTGCCCTATCAACTTTC 272
AB511835.1 SAG 11-32 CGGTGAATCATGATAACTTCACGAATCGTATGGGCTCGTCCCGACGATGTTTCATTCAAATTTCTGCCCTATCAACTTTC 272
AB511834.1 UTEX 90   CGGTGAATCATGATAACTTCACGAATCGTATGGGCTCGTCCCGACGATGTTTCATTCAAATTTCTGCCCTATCAACTTTC 272
KC166137.1 K01       CGGTGAATCATGATAACTTCACGAATCGTATGGGCTCGTCCCGACGATGTTTCATTCAAATTTCTGCCCTATCAACTTTC 103
KX781338.1 P1        -------------------------------------------------------------------------------- 0
KF864473.1 JinCheon1 CGGTGAATCATGATAACTTCACGAATCGTATGGCCTCGTGCCGACGATGTTTCATTCAAATTTCTGCCCTATCAACTTTC 318
EU925397.1 CC-124    -------------------------------------------------------------------------------- 0
JN903974.1 SAG18.79  CGGTGAATCATGATAACTTCACGAATCGTATGGGCTCGTCCCGACGATGTTTCATTCAAATTTCTGCCCTATCAACTTTC 298
JN903978.1 SAG11-32c CGGTGAATCATGATAACTTCACGAATCGTATGGGCTCGTCCCGACGATGTTTCATTCAAATTTCTGCCCTATCAACTTTC 299
KX781325.1 P15       CGGTGAATCATGATAACTTCACGAATCGTATGGGCTCGTCCCGACGATGTTTCATTCAAATTTCTGCCCTATCAACTTTC 231
JN863299.1 KNUA021   CGGTGAATCATGATAACTTCACGAATCGTATGGGCTCGTCCCGACGATGTTTCATTCAAATTTCTGCCCTATCAACTTTC 301
KR904893.1 CBS152280 -------------------------------------------------------------------------------- 0
KX781335.1 P4        CGGTGAATCATGATAACTTCACGAATCGTATGGGCTCGTCCCGACGATGTTTCATTCAAATTTCTGCCCTATCAACTTTC 231
JX888472.1 CC-621    CGGTGAATCATGATAACTTCACGAATCGTATGGGCTCGTCCCGACGATGTTTCATTCAAATTTCTGCCCTATCAACTTTC 301
KC310450.1 RAC       -------------------------------------------------------------------------------- 0
JX888471.1 CC-620    CGGTGAATCATGATAACTTCACGAATCGTATGGGCTCGTCCCGACGATGTTTCATTCAAATTTCTGCCCTATCAACTTTC 301

KX781331.1 P8        GATGGTAGGATAGAGGCCTACCATGGTGGTAACGGGTGACGGAGGATTAGGGTTCGATT-CCGGAG-AGGGAGCCTGAGA 360
KX781328.1 P12       GATGGTAGGATAGAGGCCTACCATGGTGGTAACGGGTGACGGAGGATTAGGGTTCGATT-CCGGAG-AGGGAGCCTGAGA 168
KX781321.1 P20       GATGGTAGGATAGAGGCCTACCATGGTGGTAACGGGTGACGGAGGATTAGGGTTCGATT-CCGGAG-AGGGAGCCTGAGA 308
KX781327.1 P13       GATGGTAGGATAGAGGCCTACCATGGTGGTAACGGGTGACGGAGGATTAGGGTTCGATT-CCGGAG-AGGGAGCCTGAGA 169
KX781337.1 P2        --------------------------------------------------GGTTCGATT-CCGGAG-AGGGAGCCTGAGA 28
JN903984.1 SAG53.72  GATGGTAGGATAGAGGCCTACCATGGTGGTAACGGGTGACGGAGGATTAGGGTTCGATT-CCGGAG-AGGGAGCCTGAGA 378
KR904894.1 CC-849    ---------------------------------------------------------------------------TGAGA 5
KC149968.1 GTD4C     GATGGTAGGATAGAGGCCTACCATGGTGGTAACGGGTGACGGAGGATTAGGGTTCGATT-CCGGAG-AGGGAGCCTGAGA 397
KX781333.1 P6        GATGGTAGGATAGAGGCCTACCATGGTGGTAACGGGTGACGGAGGATTAGGGTTCGATT-CCGGAG-AGGGAGCCTGAGA 239
AY665726.1 CC-1418   GATGGTAGGATAGAGGCCTACCATGGTGGTAACGGGTGACGGAGGATTAGGGTTCGATT-CCGGAG-AGGGAGCCTGAGA 356
AY665727.1 CC-1952   GATGGTAGGATAGAGGCCTACCATGGTGGTAACGGGTGACGGAGGATTAGGGTTCGATT-CCGGAG-AGGGAGCCTGAGA 350
AB701550.1 NIES-2235 GATGGTAGGATAGAGGCCTACCATGGTGGTAACGGGTGACGGAGGATTAGGGTTCGATT-CCGGAG-AGGGAGCCTGAGA 314
AB701554.1 NIES-2239 GATGGTAGGATAGAGGCCTACCATGGTGGTAACGGGTGACGGAGGATTAGGGTTCGATT-CCGGAG-AGGGAGCCTGAGA 296
AB701553.1 NIES-2238 GATGGTAGGATAGAGGCCTACCATGGTGGTAACGGGTGACGGAGGATTAGGGTTCGATT-CCGGAG-AGGGAGCCTGAGA 308
AB753040.1 PS-2708   GATGGTAGGATAGAGGCCTACCATGGTGGTAACGGGTGACGGAGGATTAGGGTTCGATT-CCGGAG-AGGGAGCCTGAGA 296
AB701552.1 NIES-2237 GATGGTAGGATAGAGGCCTACCATGGTGGTAACGGGTGACGGAGGATTAGGGTTCGATT-CCGGAG-AGGGAGCCTGAGA 307
AB701551.1 NIES-2236 GATGGTAGGATAGAGGCCTACCATGGTGGTAACGGGTGACGGAGGATTAGGGTTCGATT-CCGGAG-AGGGAGCCTGAGA 299
AB701555.1 NIES-2463 GATGGTAGGATAGAGGCCTACCATGGTGGTAACGGGTGACGGAGGATTAGGGTTCGATT-CCGGAG-AGGGAGCCTGAGA 310
```

```
KR092109.1 CC-125    GATGGTAGGATAGAGGCCTACCATGGTGGTAACGGGTGACGGAGGATTAGGGTTCGATT-CCGGAG-AGGGAGCCTGAGA 336
KX781322.1 P18       GATGGTAGGATAGAGGCCTACCATGGTGGTAACGGGTGACGGAGGATTAGGGTTCGATT-CCGGAG-AGGGAGCCTGAGA 166
KX781326.1 P14       GATGGTAGGATAGAGGCCTACCATGGTGGTAACGGGTGACGGAGGATTAGGGTTCGATT-CCGGAG-AGGGAGCCTGAGA 307
KX781329.1 P11       GATGGTAGGATAGAGGCCTACCATGGTGGTAACGGGTGACGGAGGATTAGGGTTCGATT-CCGGAG-AGGGAGCCTGAGA 98
KX781330.1 P10       GATGGTAGGATAGAGGCCTACCATGGTGGTAACGGGTGACGGAGGATTAGGGTTCGATT-CCGGAG-AGGGAGCCTGAGA 196
KX781332.1 P7        -------------------------------------------------------------------------------- 0
AB511837.1 KkS0801D2 GATGGTAGGATAGAGGCCTACCATGGTGGTAACGGGTGACGGAGGATTAGGGTTCGATT-CCGGAG-AGGGAGCCTGAGA 350
AB511836.1 KkS0801B1 GATGGTAGGATAGAGGCCTACCATGGTGGTAACGGGTGACGGAGGATTAGGGTTCGATT-CCGGAG-AGGGAGCCTGAGA 350
AB511835.1 SAG 11-32 GATGGTAGGATAGAGGCCTACCATGGTGGTAACGGGTGACGGAGGATTAGGGTTCGATT-CCGGAG-AGGGAGCCTGAGA 350
AB511834.1 UTEX 90   GATGGTAGGATAGAGGCCTACCATGGTGGTAACGGGTGACGGAGGATTAGGGTTCGATT-CCGGAG-AGGGAGCCTGAGA 350
KC166137.1 K01       GATGGTAGGATAGAGGCCTACCATGGTGGTAACGGGTGACGGAGGATTAGGGTTCGATT-CCGGAG-AGGGAGCCTGAGA 181
KX781338.1 P1        -------------------------------------------------------------------------------- 0
KF864473.1 JinCheon1 GATGGTAGGATAGAGGCCTACCATGGTGGTAACGGGTGACGGAGGATTAGGGTTCGAT-TCCGGAG-AGGGAGCCTGAGA 396
EU925397.1 CC-124    -------------------------------------------------CGATTCGCCCTTCGGAG-AGGGAGCATGAGA 30
JN903974.1 SAG18.79  GATGGTAGGATAGAGGCCTACCATGGTGGTAACGGGTGACGGAGGATTAGGGTTCGAT-TCCGGAGAG-GGAGCCTGAGA 376
JN903978.1 SAG11-32c GATGGTAGGATAGAGGCCTACCATGGTGGTAACGGGTGACGGAGGATTAGGGTTCGAT-TCCGGAGAG-GGAGCCTGAGA 377
KX781325.1 P15       GATGGTAGGATAGAGGCCTACCATGGTGGTAACGGGTGACGGAGGATTAGGGTTCGAT-TCCGGAGAG-GGAGCCTGAGA 309
JN863299.1 KNUA021   GATGGTAGGATAGAGGCCTACCATGGTGGTAACGGGTGACGGAGGATTAGGGTTCGAT-TCCGGAGAG-GGAGCCTGAGA 379
KR904893.1 CBS152280 ------------------------------------------------------------TCGGAGAGGGAAGCATGAGA 20
KX781335.1 P4        GATGGTAGGATAGAGGCCTACCATGGTGGTAACGGGTGACGGAGGATTAGGGTTCGAT-TCCGGAGA-GGGAGCCTGAGA 309
JX888472.1 CC-621    GATGGTAGGATAGAGGCCTACCATGGTGGTAACGGGTGACGGAGGATTAGGGTTCGAT-TCCGGAGA-GGGAGCCTGAGA 379
KC310450.1 RAC       -------------------------------------------------------------------------------- 0
JX888471.1 CC-620    GATGGTAGGATAGAGGCCTACCATGGTGGTAACGGGTGACGGAGGATTAGGGTTCGAT-TCCGGAGA-GGGAGCCTGAGA 379

KX781331.1 P8        GATGGCTACCACATCCAAGGAAGGCAGCAGGCGCGCAAATTACCCAATCCCAACACGGGGAGGTAGTGACAATAAATAAC 440
KX781328.1 P12       GATGGCTACCACATCCAAGGAAGGCAGCAGGCGCGCAAATTACCCAATCCCGACACGGGGAGGTAGTGACAATAAATAAC 248
KX781321.1 P20       GATGGCTACCACATCCAAGGAAGGCAGCAGGCGCGCAAATTACCCAATCCCAACACGGGGAGGTAGTGACAATAAATAAC 388
KX781327.1 P13       GATGGCTACCACATCCAAGGAAGGCAGCAGGCGCGCAAATTACCCAATCCCGACACGGGGAGGTAGTGACAATAAATAAC 249
KX781337.1 P2        GATGGCTACCACATCCAAGGAAGGCAGCAGGCGCGCAAATTACCCAATCCCAACACGGGGAGGTAGTGACAATAAATAAC 108
JN903984.1 SAG53.72  GATGGCTACCACATCCAAGGAAGGCAGCAGGCGCGCAAATTACCCAATCCCAACACGGGGAGGTAGTGACAATAAATAAC 458
KR904894.1 CC-849    GATGGCTACCACATCCAAGGAAGGCAGCAGGCGCGCAAATTACCCAATCCCGACACGGGGAGGTAGTGACAATAAATAAC 85
KC149968.1 GTD4C     GATGGCTACCACATCCAAGGAAGGCAGCAGGCGCGCAAATTACCCAATCCCGACACGGGGAGGTAGTGACAATAAATAAC 477
KX781333.1 P6        GATGGCTACCACATCCAAGGAAGGCAGCAGGCGCGCAAATTACCCAATCCCGACACGGGGAGGTAGTGACAATAAATAAC 319
AY665726.1 CC-1418   GATGGCTACCACATCCAAGGAAGGCAGCAGGCGCGCAAATTACCCAATCCCGACACGGGGAGGTAGTGACAATAAATAAC 436
AY665727.1 CC-1952   GATGGCTACCACATCCAAGGAAGGCAGCAGGCGCGCAAATTACCCAATCCCGACACGGGGAGGTAGTGACAATAAATAAC 430
AB701550.1 NIES-2235 GATGGCTACCACATCCAAGGAAGGCAGCAGGCGCGCAAATTACCCAATCCCGACACGGGGAGGTAGTGACAATAAATAAC 394
AB701554.1 NIES-2239 GATGGCTACCACATCCAAGGAAGGCAGCAGGCGCGCAAATTACCCAATCCCGACACGGGGAGGTAGTGACAATAAATAAC 376
AB701553.1 NIES-2238 GATGGCTACCACATCCAAGGAAGGCAGCAGGCGCGCAAATTACCCAATCCCGACACGGGGAGGTAGTGACAATAAATAAC 388
AB753040.1 PS-2708   GATGGCTACCACATCCAAGGAAGGCAGCAGGCGCGCAAATTACCCAATCCCGACACGGGGAGGTAGTGACAATAAATAAC 376
AB701552.1 NIES-2237 GATGGCTACCACATCCAAGGAAGGCAGCAGGCGCGCAAATTACCCAATCCCGACACGGGGAGGTAGTGACAATAAATAAC 387
AB701551.1 NIES-2236 GATGGCTACCACATCCAAGGAAGGCAGCAGGCGCGCAAATTACCCAATCCCGACACGGGGAGGTAGTGACAATAAATAAC 379
AB701555.1 NIES-2463 GATGGCTACCACATCCAAGGAAGGCAGCAGGCGCGCAAATTACCCAATCCCGACACGGGGAGGTAGTGACAATAAATAAC 390
KR092109.1 CC-125    GATGGCTACCACATCCAAGGAAGGCAGCAGGCGCGCAAATTACCCAATCCCGACACGGGGAGGTAGTGACAATAAATAAC 416
KX781322.1 P18       GATGGCTACCACATCCAAGGAAGGCAGCAGGCGCGCAAATTACCCAATCCCGACACGGGGAGGTAGTGACAATAAATAAC 246
KX781326.1 P14       GATGGCTACCACATCCAAGGAAGGCAGCAGGCGCGCAAATTACCCAATCCCGACACGGGGAGGTAGTGACAATAAATAAC 387
KX781329.1 P11       GATGGCTACCACATCCAAGGAAGGCAGCAGGCGCGCAAATTACCCAATCCCGACACGGGGAGGTAGTGACAATAAATAAC 178
KX781330.1 P10       GATGGCTACCACATCCAAGGAAGGCAGCAGGCGCGCAAATTACCCAATCCCGACACGGGGAGGTAGTGACAATAAATAAC 276
KX781332.1 P7        -------------------------------------------------------------------------------- 0
AB511837.1 KkS0801D2 GATGGCTACCACATCCAAGGAAGGCAGCAGGCGCGCAAATTACCCAATCCCGACACGGGGAGGTAGTGACAATAAATAAC 430
AB511836.1 KkS0801B1 GATGGCTACCACATCCAAGGAAGGCAGCAGGCGCGCAAATTACCCAATCCCGACACGGGGAGGTAGTGACAATAAATAAC 430
AB511835.1 SAG 11-32 GATGGCTACCACATCCAAGGAAGGCAGCAGGCGCGCAAATTACCCAATCCCGACACGGGGAGGTAGTGACAATAAATAAC 430
AB511834.1 UTEX 90   GATGGCTACCACATCCAAGGAAGGCAGCAGGCGCGCAAATTACCCAATCCCGACACGGGGAGGTAGTGACAATAAATAAC 430
KC166137.1 K01       GATGGCTACCACATCCAAGGAAGGCAGCAGGCGCGCAAATTACCCAATCCCGACACGGGGAGGTAGTGACAATAAATAAC 261
KX781338.1 P1        -------------------------------------------------------------------------------- 0
KF864473.1 JinCheon1 GATGGCTACCACATCCAAGGAAGGCAGCAGGCGCGCAAATTACCCAATCCCGACACGGGGAGGTAGTGACAATAAATAAC 476
EU925397.1 CC-124    GATGGCTACCACATCCAAGGAAGGCAGCAGGCGCGCAAATTACCCAATCCCGACACGGGGAGGTAGTGACAATAAATAAC 110
JN903974.1 SAG18.79  GATGGCTACCACATCCAAGGAAGGCAGCAGGCGCGCAAATTACCCAATCCCGACACGGGGAGGTAGTGACAATAAATAAC 456
JN903978.1 SAG11-32c GATGGCTACCACATCCAAGGAAGGCAGCAGGCGCGCAAATTACCCAATCCCGACACGGGGAGGTAGTGACAATAAATAAC 457
KX781325.1 P15       GATGGCTACCACATCCAAGGAAGGCAGCAGGCGCGCAAATTACCCAATCCCGACACGGGGAGGTAGTGACAATAAATAAC 389
JN863299.1 KNUA021   GATGGCTACCACATCCAAGGAAGGCAGCAGGCGCGCAAATTACCCAATCCCGACACGGGGAGGTAGTGACAATAAATAAC 459
KR904893.1 CBS152280 GATGGCTACCACATCCAAGGAAGGCAGCAGGCGCGCAAATTACCCAATCCCGACACGGGGAGGTAGTGACAATAAATAAC 100
KX781335.1 P4        GATGGCTACCACATCCAAGGAAGGCAGCAGGCGCGCAAATTACCCAATCCCGACACGGGGAGGTAGTGACAATAAATAAC 389
JX888472.1 CC-621    GATGGCTACCACATCCAAGGAAGGCAGCAGGCGCGCAAATTACCCAATCCCGACACGGGGAGGTAGTGACAATAAATAAC 459
KC310450.1 RAC       -------------------------------------------------------------------------------- 0
JX888471.1 CC-620    GATGGCTACCACATCCAAGGAAGGCAGCAGGCGCGCAAATTACCCAATCCCGACACGGGGAGGTAGTGACAATAAATAAC 459

KX781331.1 P8        AATACCGGGCATTTCATGTCTGGTAATTGGAATGAGTACAATCTAAATCCCTTAACGAGGATCCATTGGAGGGCAAGTCT 520
KX781328.1 P12       AATACCGGGCGCTTCGCGTCTGGTAATTGGAATGAGTACAATCTAAATCCCTTAACGAGGATCCATTGGAGGGCAAGTCT 328
KX781321.1 P20       AATACCGGGCATTTCATGTCTGGTAATTGGAATGAGTACAATCTAAATCCCTTAACGAGGATCCATTGGAGGGCAAGTCT 468
KX781327.1 P13       AATACCGGGCGCTTCGCGTCTGGTAATTGGAATGAGTACAATCTAAATCCCTTAACGAGGATCCATTGGAGGGCAAGTCT 329
KX781337.1 P2        AATACCGGGCATTTCATGTCTGGTAATTGGAATGAGTACAATCTAAATCCCTTAACGAGGATCCATTGGAGGGCAAGTCT 188
JN903984.1 SAG53.72  AATACCGGGCATTTCATGTCTGGTAATTGGAATGAGTACAATCTAAATCCCTTAACGAGGATCCATTGGAGGGCAAGTCT 538
KR904894.1 CC-849    AATACCGGGCGCTTCGCGTCTGGTAATTGGAATGAGTACAATCTAAATCCCTTAACGAGGATCCATTGGAGGGCAAGTCT 165
KC149968.1 GTD4C     AATACCGGGCGCTTAGCGTCTGGTAATTGGAATGAGTACAATCTAAATCCCTTAACGAGGATCCATTGGAGGGCAAGTCT 557
KX781333.1 P6        AATACCGGGCGCTTCGCGTCTGGTAATTGGAATGAGTACAATCTAAATCCCTTAACGAGGATCCATTGGAGGGCAAGTCT 399
AY665726.1 CC-1418   AATACCGGGCGCTTCGCGTCTGGTAATTGGAATGAGTACAATCTAAATCCCTTAACGAGGATCCATTGGAGGGCAAGTCT 516
AY665727.1 CC-1952   AATACCGGGCGCTTCGCGTCTGGTAATTGGAATGAGTACAATCTAAATCCCTTAACGAGGATCCATTGGAGGGCAAGTCT 510
AB701550.1 NIES-2235 AATACCGGGCGCTTCGCGTCTGGTAATTGGAATGAGTACAATCTAAATCCCTTAACGAGGATCCATTGGAGGGCAAGTCT 474
AB701554.1 NIES-2239 AATACCGGGCGCTTCGCGTCTGGTAATTGGAATGAGTACAATCTAAATCCCTTAACGAGGATCCATTGGAGGGCAAGTCT 456
AB701553.1 NIES-2238 AATACCGGGCGCTTCGCGTCTGGTAATTGGAATGAGTACAATCTAAATCCCTTAACGAGGATCCATTGGAGGGCAAGTCT 468
AB753040.1 PS-2708   AATACCGGGCGCTTCGCGTCTGGTAATTGGAATGAGTACAATCTAAATCCCTTAACGAGGATCCATTGGAGGGCAAGTCT 456
AB701552.1 NIES-2237 AATACCGGGCGCTTCGCGTCTGGTAATTGGAATGAGTACAATCTAAATCCCTTAACGAGGATCCATTGGAGGGCAAGTCT 467
AB701551.1 NIES-2236 AATACCGGGCGCTTCGCGTCTGGTAATTGGAATGAGTACAATCTAAATCCCTTAACGAGGATCCATTGGAGGGCAAGTCT 459
AB701555.1 NIES-2463 AATACCGGGCGCTTCGCGTCTGGTAATTGGAATGAGTACAATCTAAATCCCTTAACGAGGATCCATTGGAGGGCAAGTCT 470
KR092109.1 CC-125    AATACCGGGCGCTTCGCGTCTGGTAATTGGAATGAGTACAATCTAAATCCCTTAACGAGGATCCATTGGAGGGCAAGTCT 496
KX781322.1 P18       AATACCGGGCGCTTCGCGTCTGGTAATTGGAATGAGTACAATCTAAATCCCTTAACGAGGATCCATTGGAGGGCAAGTCT 326
KX781326.1 P14       AATACCGGGCGCTTCGCGTCTGGTAATTGGAATGAGTACAATCTAAATCCCTTAACGAGGATCCATTGGAGGGCAAGTCT 467
KX781329.1 P11       AATACCGGGCGCTTCGCGTCTGGTAATTGGAATGAGTACAATCTAAATCCCTTAACGAGGATCCATTGGAGGGCAAGTCT 258
KX781330.1 P10       AATACCGGGCGCTTCGCGTCTGGTAATTGGAATGAGTACAATCTAAATCCCTTAACGAGGATCCATTGGAGGGCAAGTCT 356
KX781332.1 P7        -------------------------------------------------------------------------------- 0
AB511837.1 KkS0801D2 AATACCGGGCGCTTCGCGTCTGGTAATTGGAATGAGTACAATCTAAATCCCTTAACGAGGATCCATTGGAGGGCAAGTCT 510
AB511836.1 KkS0801B1 AATACCGGGCGCTTCGCGTCTGGTAATTGGAATGAGTACAATCTAAATCCCTTAACGAGGATCCATTGGAGGGCAAGTCT 510
AB511835.1 SAG 11-32 AATACCGGGCGCTTCGCGTCTGGTAATTGGAATGAGTACAATCTAAATCCCTTAACGAGGATCCATTGGAGGGCAAGTCT 510
AB511834.1 UTEX 90   AATACCGGGCGCTTCGCGTCTGGTAATTGGAATGAGTACAATCTAAATCCCTTAACGAGGATCCATTGGAGGGCAAGTCT 510
```

```
KC166137.1 K01       AATACCGGGCGCTTCGCGTCTGGTAATTGGAATGAGTACAATCTAAATCCCTTAACGAGGATCCATTGGAGGGCAAGTCT 341
KX781338.1 P1        -------------------------------------------------------------------------------- 0
KF864473.1 JinCheon1 AATACCGGGCGCTTCGCGTCTGGTAATTGGAATGAGTACAATCTAAATCCCTTAACGAGGATCCATTGGAGGGCAAGTCT 556
EU925397.1 CC-124    AATACCGGGCGCTTCGCGTCTGGTAATTGGAATGAGTACAATCTAAATCCCTTAACGAGGATCCATTGGAGGGCAAGTCT 190
JN903974.1 SAG18.79  AATACCGGGCGCTTCGCGTCTGGTAATTGGAATGAGTACAATCTAAATCCCTTAACGAGGATCCATTGGAGGGCAAGTCT 536
JN903978.1 SAG11-32c AATACCGGGCGCTTCGCGTCTGGTAATTGGAATGAGTACAATCTAAATCCCTTAACGAGGATCCATTGGAGGGCAAGTCT 537
KX781325.1 P15       AATACCGGGCGCTTCGCGTCTGGTAATTGGAATGAGTACAATCTAAATCCCTTAACGAGGATCCATTGGAGGGCAAGTCT 469
JN863299.1 KNUA021   AATACCGGGCGCTTCGCGTCTGGTAATTGGAATGAGTACAATCTAAATCCCTTAACGAGGATCCATTGGAGGGCAAGTCT 539
KR904893.1 CBS152280 AATACCGGGCGCTTCGCGTCTGGTAATTGGAATGAGTACAATCTAAATCCCTTAACGAGGATCCATTGGAGGGCAAGTCT 180
KX781335.1 P4        AATACCGGGCGCTTCGCGTCTGGTAATTGGAATGAGTACAATCTAAATCCCTTAACGAGGATCCATTGGAGGGCAAGTCT 469
JX888472.1 CC-621    AATACCGGGCGCTTCGCGTCTGGTAATTGGAATGAGTACAATCTAAATCCCTTAACGAGGATCCATTGGAGGGCAAGTCT 539
KC310450.1 RAC       -------------------------------------------------------------------------------- 0
JX888471.1 CC-620    AATACCGGGCGCTTCGCGTCTGGTAATTGGAATGAGTACAATCTAAATCCCTTAACGAGGATCCATTGGAGGGCAAGTCT 539

KX781331.1 P8        GGTGCCAGCAGCCGCGGTAATTCCAGCTCCAATAGCGTATATTTAAGTTGTTGCAGTTAAAAAGCTCGTAGTTGGATTTC 600
KX781328.1 P12       GGTGCCAGCAGCCGCGGTAATTCCAGCTCCAATAGCGTATATTTAAGTTGTTGCAGTTAAAAAGCTCGTAGTTGGATTTC 408
KX781321.1 P20       GGTGCCAGCAGCCGCGGTAATTCCAGCTCCAATAGCGTATATTTAAGTTGTTGCAGTTAAAAAGCTCGTAGTTGGATTTC 548
KX781327.1 P13       GGTGCCAGCAGCCGCGGTAATTCCAGCTCCAATAGCGTATATTTAAGTTGTTGCAGTTAAAAAGCTCGTAGTTGGATTTC 409
KX781337.1 P2        GGTGCCAGCAGCCGCGGTAATTCCAGCTCCAATAGCGTATATTTAAGTTGTTGCAGTTAAAAAGCTCGTAGTTGGATTTC 268
JN903984.1 SAG53.72  GGTGCCAGCAGCCGCGGTAATTCCAGCTCCAATAGCGTATATTTAAGTTGTTGCAGTTAAAAAGCTCGTAGTTGGATTTC 618
KR904894.1 CC-849    GGTGCCAGCAGCCGCGGTAATTCCAGCTCCAATAGCGTATATTTAAGTTGTTGCAGTTAAAAAGCTCGTAGTTGGATTTC 245
KC149968.1 GTD4C     GGTGCCAGCAGCCGCGGTAATTCCAGCTCCAATAGCGTATATTTAAGTTGTTGCAGTTAAAAAGCTCGTAGTTGGATTTC 637
KX781333.1 P6        GGTGCCAGCAGCCGCGGTAATTCCAGCTCCAATAGCGTATATTTAAGTTGTTGCAGTTAAAAAGCTCGTAGTTGGATTTC 479
AY665726.1 CC-1418   GGTGCCAGCAGCCGCGGTAATTCCAGCTCCAATAGCGTATATTTAAGTTGTTGCAGTTAAAAAGCTCGTAGTTGGATTTC 596
AY665727.1 CC-1952   GGTGCCAGCAGCCGCGGTAATTCCAGCTCCAATAGCGTATATTTAAGTTGTTGCAGTTAAAAAGCTCGTAGTTGGATTTC 590
AB701550.1 NIES-2235 GGTGCCAGCAGCCGCGGTAATTCCAGCTCCAATAGCGTATATTTAAGTTGTTGCAGTTAAAAAGCTCGTAGTTGGATTTC 554
AB701554.1 NIES-2239 GGTGCCAGCAGCCGCGGTAATTCCAGCTCCAATAGCGTATATTTAAGTTGTTGCAGTTAAAAAGCTCGTAGTTGGATTTC 536
AB701553.1 NIES-2238 GGTGCCAGCAGCCGCGGTAATTCCAGCTCCAATAGCGTATATTTAAGTTGTTGCAGTTAAAAAGCTCGTAGTTGGATTTC 548
AB753040.1 PS-2708   GGTGCCAGCAGCCGCGGTAATTCCAGCTCCAATAGCGTATATTTAAGTTGTTGCAGTTAAAAAGCTCGTAGTTGGATTTC 536
AB701552.1 NIES-2237 GGTGCCAGCAGCCGCGGTAATTCCAGCTCCAATAGCGTATATTTAAGTTGTTGCAGTTAAAAAGCTCGTAGTTGGATTTC 547
AB701551.1 NIES-2236 GGTGCCAGCAGCCGCGGTAATTCCAGCTCCAATAGCGTATATTTAAGTTGTTGCAGTTAAAAAGCTCGTAGTTGGATTTC 539
AB701555.1 NIES-2463 GGTGCCAGCAGCCGCGGTAATTCCAGCTCCAATAGCGTATATTTAAGTTGTTGCAGTTAAAAAGCTCGTAGTTGGATTTC 550
KR092109.1 CC-125    GGTGCCAGCAGCCGCGGTAATTCCAGCTCCAATAGCGTATATTTAAGTTGTTGCAGTTAAAAAGCTCGTAGTTGGATTTC 576
KX781322.1 P18       GGTGCCAGCAGCCGCGGTAATTCCAGCTCCAATAGCGTATATTTAAGTTGTTGCAGTTAAAAAGCTCGTAGTTGGATTTC 406
KX781326.1 P14       GGTGCCAGCAGCCGCGGTAATTCCAGCTCCAATAGCGTATATTTAAGTTGTTGCAGTTAAAAAGCTCGTAGTTGGATTTC 547
KX781329.1 P11       GGTGCCAGCAGCCGCGGTAATTCCAGCTCCAATAGCGTATATTTAAGTTGTTGCAGTTAAAAAGCTCGTAGTTGGATTTC 338
KX781330.1 P10       GGTGCCAGCAGCCGCGGTAATTCCAGCTCCAATAGCGTATATTTAAGTTGTTGCAGTTAAAAAGCTCGTAGTTGGATTTC 436
KX781332.1 P7        --------------------------------------ATATTTAAGTTGTTGCAGTTAAAAAGCTCGTAGTTGGATTTC 42
AB511837.1 KkS0801D2 GGTGCCAGCAGCCGCGGTAATTCCAGCTCCAATAGCGTATATTTAAGTTGTTGCAGTTAAAAAGCTCGTAGTTGGATTTC 590
AB511836.1 KkS0801B1 GGTGCCAGCAGCCGCGGTAATTCCAGCTCCAATAGCGTATATTTAAGTTGTTGCAGTTAAAAAGCTCGTAGTTGGATTTC 590
AB511835.1 SAG 11-32 GGTGCCAGCAGCCGCGGTAATTCCAGCTCCAATAGCGTATATTTAAGTTGTTGCAGTTAAAAAGCTCGTAGTTGGATTTC 590
AB511834.1 UTEX 90   GGTGCCAGCAGCCGCGGTAATTCCAGCTCCAATAGCGTATATTTAAGTTGTTGCAGTTAAAAAGCTCGTAGTTGGATTTC 590
KC166137.1 K01       GGTGCCAGCAGCCGCGGTAATTCCAGCTCCAATAGCGTATATTTAAGTTGTTGCAGTTAAAAAGCTCGTAGTTGGATTTC 421
KX781338.1 P1        -------------------------------------------------------------------------------- 0
KF864473.1 JinCheon1 GGTGCCAGCAGCCGCGGTAATTCCAGCTCCAATAGCGTATATTTAAGTTGTTGCAGTTAAAAAGCTCGTAGTTGGATTTC 636
EU925397.1 CC-124    GGTGCCAGCAGCCGCGGTAATTCCAGCTCCAATAGCGTATATTTAAGTTGTTGCAGTTAAAAAGCTCGTAGTTGGATTTC 270
JN903974.1 SAG18.79  GGTGCCAGCAGCCGCGGTAATTCCAGCTCCAATAGCGTATATTTAAGTTGTTGCAGTTAAAAAGCTCGTAGTTGGATTTC 616
JN903978.1 SAG11-32c GGTGCCAGCAGCCGCGGTAATTCCAGCTCCAATAGCGTATATTTAAGTTGTTGCAGTTAAAAAGCTCGTAGTTGGATTTC 617
KX781325.1 P15       GGTGCCAGCAGCCGCGGTAATTCCAGCTCCAATAGCGTATATTTAAGTTGTTGCAGTTAAAAAGCTCGTAGTTGGATTTC 549
JN863299.1 KNUA021   GGTGCCAGCAGCCGCGGTAATTCCAGCTCCAATAGCGTATATTTAAGTTGTTGCAGTTAAAAAGCTCGTAGTTGGATTTC 619
KR904893.1 CBS152280 GGTGCCAGCAGCCGCGGTAATTCCAGCTCCAATAGCGTATATTTAAGTTGTTGCAGTTAAAAAGCTCGTAGTTGGATTTC 260
KX781335.1 P4        GGTGCCAGCAGCCGCGGTAATTCCAGCTCCAATAGCGTATATTTAAGTTGTTGCAGTTAAAAAGCTCGTAGTTGGATTTC 549
JX888472.1 CC-621    GGTGCCAGCAGCCGCGGTAATTCCAGCTCCAATAGCGTATATTTAAGTTGTTGCAGTTAAAAAGCTCGTAGTTGGATTTC 619
KC310450.1 RAC       -------------------------------------------------------------------------------- 0
JX888471.1 CC-620    GGTGCCAGCAGCCGCGGTAATTCCAGCTCCAATAGCGTATATTTAAGTTGTTGCAGTTAAAAAGCTCGTAGTTGGATTTC 619

KX781331.1 P8        GGGTGGG-TCTTAGCGGTCCGCCTCTGGTGTGTACTGCTAGGGCCTATCTTTCTGCCGGGGACGGCTCCTGGGTTTAAT 679
KX781328.1 P12       GGGTGGG-GTGGTGCGGTCCGCCTCTGGTGTGCACTGCTCTGCTCCACCTTCCTGCCGGGGACGGCTCCTGGGCTTCAC 487
KX781321.1 P20       GGGTGGG-TCTTAGCGGTCCGCCTCTGGTGTGTACTGCTAGGGCCTATCTTTCTGCCGGGGACGGCTCCTGGGTTTAAT 627
KX781327.1 P13       GGGTGG-GGTGGTGCGGTCCGCCTCTGGTGTGCACTGCTCTGCTCCACCTTCCTGCCGGGGACGGCTCCTGGGCTTCAC 488
KX781337.1 P2        GGGTGG-GTCTTAGCGGTCCGCCTCTGGTGTGTACTGCTAGGGCCTATCTTTCTGCCGGGGACGGCTCCTGGGTTTAAT 347
JN903984.1 SAG53.72  GGGTGGG-TCTTAGCGGTCCGCCTCTGGTGTGTACTGCTAGGGCCTATCTTTCTGCCGGGGACGGCTCCTGGGTTTAAT 697
KR904894.1 CC-849    GGGTGG-GGTGGTGCGGTCCGCCTCTGGTGTGCACTGCTCTGCTCCACCTTCCTGCCGGGGACGGCTCCTGGGCTTCAC 324
KC149968.1 GTD4C     GGGTGG-GGTGGTGCGGTCCGCCTCTGGTGTGCACTGCTCCGCTCCACCTTCCTGCCGGGGACGGCTCCTGGGCTTAAC 716
KX781333.1 P6        GGGTGG-GGTGGTGCGGTCCGCCTCTGGTGTGCACTGCTCTGCTCCACCTTCCTGCCGGGGACGGCTCCTGGGCTTCAC 558
AY665726.1 CC-1418   GGGTGG-GGTGGTGCGGTCCGCCTCTGGTGTGCACTGCTCTGCTCCACCTTCCTGCCGGGGACGGCTCCTGGGCTTCAC 675
AY665727.1 CC-1952   GGGTGG-GGTGGTGCGGTCCGCCTCTGGTGTGCACTGCTCTGCTCCACCTTCCTGCCGGGGACGGCTCCTGGGCTTCAC 669
AB701550.1 NIES-2235 GGGTGG-GGTGGTGCGGTCCGCCTCTGGTGTGCACTGCTCTGCTCCACCTTCCTGCCGGGGACGGCTCCTGGGCTTCAC 633
AB701554.1 NIES-2239 GGGTGG-GGTGGTGCGGTCCGCCTCTGGTGTGCACTGCTCTGCTCCACCTTCCTGCCGGGGACGGCTCCTGGGCTTCAC 615
AB701553.1 NIES-2238 GGGTGG-GGTGGTGCGGTCCGCCTCTGGTGTGCACTGCTCTGCTCCACCTTCCTGCCGGGGACGGCTCCTGGGCTTCAC 627
AB753040.1 PS-2708   GGGTGG-GGTGGTGCGGTCCGCCTCTGGTGTGCACTGCTCTGCTCCACCTTCCTGCCGGGGACGGCTCCTGGGCTTCAC 615
AB701552.1 NIES-2237 GGGTGG-GGTGGTGCGGTCCGCCTCTGGTGTGCACTGCTCTGCTCCACCTTCCTGCCGGGGACGGCTCCTGGGCTTCAC 626
AB701551.1 NIES-2236 GGGTGG-GGTGGTGCGGTCCGCCTCTGGTGTGCACTGCTCTGCTCCACCTTCCTGCCGGGGACGGCTCCTGGGCTTCAC 618
AB701555.1 NIES-2463 GGGTGG-GGTGGTGCGGTCCGCCTCTGGTGTGCACTGCTCTGCTCCACCTTCCTGCCGGGGACGGCTCCTGGGCTTCAC 629
KR092109.1 CC-125    GGGTGG-GGTGGTGCGGTCCGCCTCTGGTGTGCACTGCTCTGCTCCACCTTCCTGCCGGGGACGGCTCCTGGGCTTCAC 655
KX781322.1 P18       GGGTGG-GGTGGTGCGGTCCGCCTCTGGTGTGCACTGCTCTGCTCCACCTTCCTGCCGGGGACGGCTCCTGGGCTTCAC 485
KX781326.1 P14       GGGTGG-GGTGGTGCGGTCCGCCTCTGGTGTGCACTGCTCTGCTCCACCTTCCTGCCGGGGACGGCTCCTGGGCTTCAC 626
KX781329.1 P11       GGGTGG-GGTGGTGCGGTCCGCCTCTGGTGTGCACTGCTCTGCTCCACCTTCCTGCCGGGGACGGCTCCTGGGCTTCAC 417
KX781330.1 P10       GGGTGG-GGTGGTGCGGTCCGCCTCTGGTGTGCACTGCTCTGCTCCACCTTCCTGCCGGGGACGGCTCCTGGGCTTCAC 515
KX781332.1 P7        GGGTGG-GGTGGTGCGGTCCGCCTCTGGTGTGCACTGCTCTGCTCCACCTTCCTGCCGGGGACGGCTCCTGGGCTTCAC 121
AB511837.1 KkS0801D2 GGGTGG-GGTGGTGCGGTCCGCCTCTGGTGTGCACTGCTCTGCTCCACCTTCCTGCCGGGGACGGCTCCTGGGCTTCAC 669
AB511836.1 KkS0801B1 GGGTGG-GGTGGTGCGGTCCGCCTCTGGTGTGCACTGCTCTGCTCCACCTTCCTGCCGGGGACGGCTCCTGGGCTTCAC 669
AB511835.1 SAG 11-32 GGGTGG-GGTGGTGCGGTCCGCCTCTGGTGTGCACTGCTCTGCTCCACCTTCCTGCCGGGGACGGCTCCTGGGCTTCAC 669
AB511834.1 UTEX 90   GGGTGG-GGTGGTGCGGTCCGCCTCTGGTGTGCACTGCTCTGCTCCACCTTCCTGCCGGGGACGGCTCCTGGGCTTCAC 669
KC166137.1 K01       GGGTGGGGGTGGTGCGGTCCGCCTCTGGTGTGCACTGCTCTGCTCCACCTTCCTGCCGGGGACGGCTCCTGGGCTTCAC 501
KX781338.1 P1        -------------------------------------------------------------------------------- 0
KF864473.1 JinCheon1 GGGTGG-GGTGGTGCGGTCCGCCTCTGGTGTGCACTGCTCTGCTCCACCTTCCTGCCGGGGACGGCTCCTGGGCTTAAC 715
EU925397.1 CC-124    GGGTGG-GGTGGTGCGGTCCGCCTCTGGTGTGCACTGCTCTGCTCCACCTTCCTGCCGGGGACGGCTCCTGGGCTTCAC 349
JN903974.1 SAG18.79  GGGTGG-GGTGGTGCGGTCCGCCTCTGGTGTGCACTGCTCTGCTCCACCTTCCTGCCGGGGACGGCTCCTGGGCTTCAC 695
JN903978.1 SAG11-32c GGGTGG-GGTGGTGCGGTCCGCCTCTGGTGTGCACTGCTCTGCTCCACCTTCCTGCCGGGGACGGCTCCTGGGCTTCAC 696
KX781325.1 P15       GGGTGG-GGTGGTGCGGTCCGCCTCTGGTGTGCACTGCTCTGCTCCACCTTCCTGCCGGGGACGGCTCCTGGGCTTCAC 628
JN863299.1 KNUA021   GGGTGG-GGTGGTGCGGTCCGCCTCTGGTGTGCACTGCTCTGCTCCACCTTCCTGCCGGGGACGGCTCCTGGGCTTCAC 698
KR904893.1 CBS152280 GGGTGG-GGTGGTGCGGTCCGCCTCTGGTGTGCACTGCTCTGCTCCACCTTCCTGCCGGGGACGGCTCCTGGGCTTCAC 339
KX781335.1 P4        GGGTGG-GGTGGTGCGGTCCGCCTCTGGTGTGCACTGCTCTGCTCCACCTTCCTGCCGGGGACGGCTCCTGGGCTTCAC 628
```

```
JX888472.1 CC-621    GGGTGG-GGTGGTGCGGTCCGCCTCTGGTGTGCACTGCTCTGCTCCACCTTCCTGCCGGGGACGGGCTCCTGGGCTTCAC 698
KC310450.1 RAC       -------------------------------------------------------------------------------- 0
JX888471.1 CC-620    GGGTGG-GGTGGTGCGGTCCGCCTCTGGTGTGCACTGCTCTGCTCCACCTTCCTGCCGGGGACGGGCTCCTGGGCTTCAC 698

KX781331.1 P8        CGCCTGGGACTCGGAGTCGGCGAGGTTACTTTGAGTAAATTAGAGTGTTCAAAGCAAGCCTACGCTCTGAATACATTAGC 759
KX781328.1 P12       TGTCTGGGACTCGGAGTCGGCGAGGTTACTTTGAGTAAATTAGAGTGTTCAAAGCAGGCCTACGCTCTGAATACATTAGC 567
KX781321.1 P20       CGCCTGGGACTCGGAGTCGGCGAGGTTACTTTGAGTAAATTAGAGTGTTCAAAGCAAGCCTACGCTCTGAATACATTAGC 707
KX781327.1 P13       TGTCTGGGACTCGGAGTCGGCGAGGTTACTTTGAGTAAATTAGAGTGTTCAAAGCAGGCCTACGCTCTGAATACATTAGC 568
KX781337.1 P2        CGCCTGGGACTCGGAGTCGGCGAGGTTACTTTGAGTAAATTAGAGTGTTCAAAGCAAGCCTACGCTCTGAATACATTAGC 427
JN903984.1 SAG53.72  CGCCTGGGACTCGGAGTCGGCGAGGTTACTTTGAGTAAATTAGAGTGTTCAAAGCAAGCCTACGCTCTGAATACATTAGC 777
KR904894.1 CC-849    TGTCTGGGACTCGGAGTCGGCGAGGTTACTTTGAGTAAATTAGAGTGTTCAAAGCAGGCCTACGCTCTGAATACATTAGC 404
KC149968.1 GTD4C     TGTCCGGGACTCGGAGTCGGCGAGGTTACTTTGAGTAAATTAGAGTGTTCAAAGCAGGCCTACGCTCTGAATACATTAGC 796
KX781333.1 P6        TGTCTGGGACTCGGAGTCGGCGAGGTTACTTTGAGTAAATTAGAGTGTTCAAAGCAGGCCTACGCTCTGAATACATTAGC 638
AY665726.1 CC-1418   TGTCTGGGACTCGGAGTCGGCGAGGTTACTTTGAGTAAATTAGAGTGTTCAAAGCAGGCCTACGCTCTGAATACATTAGC 755
AY665727.1 CC-1952   TGTCTGGGACTCGGAGTCGGCGAGGTTACTTTGAGTAAATTAGAGTGTTCAAAGCAGGCCTACGCTCTGAATACATTAGC 749
AB701550.1 NIES-2235 TGTCTGGGACTCGGAGTCGGCGAGGTTACTTTGAGTAAATTAGAGTGTTCAAAGCAGGCCTACGCTCTGAATACATTAGC 713
AB701554.1 NIES-2239 TGTCTGGGACTCGGAGTCGGCGAGGTTACTTTGAGTAAATTAGAGTGTTCAAAGCAGGCCTACGCTCTGAATACATTAGC 695
AB701553.1 NIES-2238 TGTCTGGGACTCGGAGTCGGCGAGGTTACTTTGAGTAAATTAGAGTGTTCAAAGCAGGCCTACGCTCTGAATACATTAGC 707
AB753040.1 PS-2708   TGTCTGGGACTCGGAGTCGGCGAGGTTACTTTGAGTAAATTAGAGTGTTCAAAGCAGGCCTACGCTCTGAATACATTAGC 695
AB701552.1 NIES-2237 TGTCTGGGACTCGGAGTCGGCGAGGTTACTTTGAGTAAATTAGAGTGTTCAAAGCAGGCCTACGCTCTGAATACATTAGC 706
AB701551.1 NIES-2236 TGTCTGGGACTCGGAGTCGGCGAGGTTACTTTGAGTAAATTAGAGTGTTCAAAGCAGGCCTACGCTCTGAATACATTAGC 698
AB701555.1 NIES-2463 TGTCTGGGACTCGGAGTCGGCGAGGTTACTTTGAGTAAATTAGAGTGTTCAAAGCAGGCCTACGCTCTGAATACATTAGC 709
KR092109.1 CC-125    TGTCTGGGACTCGGAGTCGGCGAGGTTACTTTGAGTAAATTAGAGTGTTCAAAGCAGGCCTACGCTCTGAATACATTAGC 735
KX781322.1 P18       TGTCTGGGACTCGGAGTCGGCGAGGTTACTTTGAGTAAATTAGAGTGTTCAAAGCAGGCCTACGCTCTGAATACATTAGC 565
KX781326.1 P14       TGTCTGGGACTCGGAGTCGGCGAGGTTACTTTGAGTAAATTAGAGTGTTCAAAGCAGGCCTACGCTCTGAATACATTAGC 706
KX781329.1 P11       TGTCTGGGACTCGGAGTCGGCGAGGTTACTTTGAGTAAATTAGAGTGTTCAAAGCAGGCCTACGCTCTGAATACATTAGC 497
KX781330.1 P10       TGTCTGGGACTCGGAGTCGGCGAGGTTACTTTGAGTAAATTAGAGTGTTCAAAGCAGGCCTACGCTCTGAATACATTAGC 595
KX781332.1 P7        TGTCTGGGACTCGGAGTCGGCGAGGTTACTTTGAGTAAATTAGAGTGTTCAAAGCAGGCCTACGCTCTGAATACATTAGC 201
AB511837.1 KkS0801D2 TGTCTGGGACTCGGAGTCGGCGAGGTTACTTTGAGTAAATTAGAGTGTTCAAAGCAGGCCTACGCTCTGAATACATTAGC 749
AB511836.1 KkS0801B1 TGTCTGGGACTCGGAGTCGGCGAGGTTACTTTGAGTAAATTAGAGTGTTCAAAGCAGGCCTACGCTCTGAATACATTAGC 749
AB511835.1 SAG 11-32 TGTCTGGGACTCGGAGTCGGCGAGGTTACTTTGAGTAAATTAGAGTGTTCAAAGCAGGCCTACGCTCTGAATACATTAGC 749
AB511834.1 UTEX 90   TGTCTGGGACTCGGAGTCGGCGAGGTTACTTTGAGTAAATTAGAGTGTTCAAAGCAGGCCTACGCTCTGAATACATTAGC 749
KC166137.1 K01       TGTCTGGGACTCGGAGTCGGCGAGGTTACTTTGAGTAAATTAGAGTGTTCAAAGCAGGCCTACGCTCTGAATACATTAGC 581
KX781338.1 P1        ---CTGGGACTCGGAGTCGGCGAGGTTACTTTGAGTAAATTAGAGTGTTCAAAGCAAGCCTACGCTCTGAATACATTAGC 77
KF864473.1 JinCheon1 TGTCTGGGACTCGGAGTCGGCGAGGTTACTTTGAGTAAATTAGAGTGTTCAAAGCAGGCCTACGCTCTGAATACATTAGC 795
EU925397.1 CC-124    TGTCTGGGACTCGGAGTCGGCGAGGTTACTTTGAGTAAATTAGAGTGTTCAAAGCAGGCCTACGCTCTGAATACATTAGC 429
JN903974.1 SAG18.79  TGTCTGGGACTCGGAGTCGGCGAGGTTACTTTGAGTAAATTAGAGTGTTCAAAGCAGGCCTACGCTCTGAATACATTAGC 775
JN903978.1 SAG11-32c TGTCTGGGACTCGGAGTCGGCGAGGTTACTTTGAGTAAATTAGAGTGTTCAAAGCAGGCCTACGCTCTGAATACATTAGC 776
KX781325.1 P15       TGTCTGGGACTCGGAGTCGGCGAGGTTACTTTGAGTAAATTAGAGTGTTCAAAGCAGGCCTACGCTCTGAATACATTAGC 708
JN863299.1 KNUA021   TGTCTGGGACTCGGAGTCGGCGAGGTTACTTTGAGTAAATTAGAGTGTTCAAAGCAGGCCTACGCTCTGAATACATTAGC 778
KR904893.1 CBS152280 TGTCTGGGACTCGGAGTCGGCGAGGTTACTTTGAGTAAATTAGAGTGTTCAAAGCAGGCCTACGCTCTGAATACATTAGC 419
KX781335.1 P4        TGTCTGGGACTCGGAGTCGGCGAGGTTACTTTGAGTAAATTAGAGTGTTCAAAGCAGGCCTACGCTCTGAATACATTAGC 708
JX888472.1 CC-621    TGTCTGGGACTCGGAGTCGGCGAGGTTACTTTGAGTAAATTAGAGTGTTCAAAGCAGGCCTACGCTCTGAATACATTAGC 778
KC310450.1 RAC       -------------------------------------------------------------------------------- 0
JX888471.1 CC-620    TGTCTGGGACTCGGAGTCGGCGAGGTTACTTTGAGTAAATTAGAGTGTTCAAAGCAGGCCTACGCTCTGAATACATTAGC 778

KX781331.1 P8        ATGGAATAACACGATAGGACTCTGGCCTATCTTGTTGGTCTGTAGGACCGGAGTAATGATTAAGAGGGACAGTCGGGGGC 839
KX781328.1 P12       ATGGAATAACACGATAGGACTCTGGCCTATCT-GTTGGTCTGTGGGACCGGAGTAATGATTAAGAGGGGTAGTCGGGGGC 646
KX781321.1 P20       ATGGAATAACACGATAGGACTCTGGCCTATCTTGTTGGTCTGTAGGACCGGAGTAATGATTAAGAGGGACAGTCGGGGGC 787
KX781327.1 P13       ATGGAATAACACGATAGGACTCTGGCCTATCT-GTTGGTCTGTGGGACCGGAGTAATGATTAAGAGGGGTAGTCGGGGGC 647
KX781337.1 P2        ATGGAATAACACGATAGGACTCTGGCCTATCTTGTTGGTCTGTAGGACCGGAGTAATGATTAAGAGGGACAGTCGGGGGC 507
JN903984.1 SAG53.72  ATGGAATAACACGATAGGACTCTGGCCTATCTTGTTGGTCTGTAGGACCGGAGTAATGATTAAGAGGGACAGTCGGGGGC 857
KR904894.1 CC-849    ATGGAATAACACGATAGGACTCTGGCCTATCT-GTTGGTCTGTGGGACCGGAGTAATGATTAAGAGGGGTAGTCGGGGGC 483
KC149968.1 GTD4C     ATGGAATAACACGATAGGACTCTGGCCTATCT-GTTGGTCTGTGGGACCGGAGTAATGATTAAGAGGGGTAGTCGGGGGC 875
KX781333.1 P6        ATGGAATAACACGATAGGACTCTGGCCTATCT-GTTGGTCTGTGGGACCGGAGTAATGATTAAGAGGGGTAGTCGGGGGC 717
AY665726.1 CC-1418   ATGGAATAACACGATAGGACTCTGGCCTATCT-GTTGGTCTGTGGGACCGGAGTAATGATTAAGAGGGGTAGTCGGGGGC 834
AY665727.1 CC-1952   ATGGAATAACACGATAGGACTCTGGCCTATCT-GTTGGTCTGTGGGACCGGAGTAATGATTAAGAGGGGTAGTCGGGGGC 828
AB701550.1 NIES-2235 ATGGAATAACACGATAGGACTCTGGCCTATCT-GTTGGTCTGTGGGACCGGAGTAATGATTAAGAGGGGTAGTCGGGGGC 792
AB701554.1 NIES-2239 ATGGAATAACACGATAGGACTCTGGCCTATCT-GTTGGTCTGTGGGACCGGAGTAATGATTAAGAGGGGTAGTCGGGGGC 774
AB701553.1 NIES-2238 ATGGAATAACACGATAGGACTCTGGCCTATCT-GTTGGTCTGTGGGACCGGAGTAATGATTAAGAGGGGTAGTCGGGGGC 786
AB753040.1 PS-2708   ATGGAATAACACGATAGGACTCTGGCCTATCT-GTTGGTCTGTGGGACCGGAGTAATGATTAAGAGGGGTAGTCGGGGGC 774
AB701552.1 NIES-2237 ATGGAATAACACGATAGGACTCTGGCCTATCT-GTTGGTCTGTGGGACCGGAGTAATGATTAAGAGGGGTAGTCGGGGGC 785
AB701551.1 NIES-2236 ATGGAATAACACGATAGGACTCTGGCCTATCT-GTTGGTCTGTGGGACCGGAGTAATGATTAAGAGGGGTAGTCGGGGGC 777
AB701555.1 NIES-2463 ATGGAATAACACGATAGGACTCTGGCCTATCT-GTTGGTCTGTGGGACCGGAGTAATGATTAAGAGGGGTAGTCGGGGGC 788
KR092109.1 CC-125    ATGGAATAACACGATAGGACTCTGGCCTATCT-GTTGGTCTGTGGGACCGGAGTAATGATTAAGAGGGGTAGTCGGGGGC 814
KX781322.1 P18       ATGGAATAACACGATAGGACTCTGGCCTATCT-GTTGGTCTGTGGGACCGGAGTAATGATTAAGAGGGGTAGTCGGGGGC 644
KX781326.1 P14       ATGGAATAACACGATAGGACTCTGGCCTATCT-GTTGGTCTGTGGGACCGGAGTAATGATTAAGAGGGGTAGTCGGGGGC 785
KX781329.1 P11       ATGGAATAACACGATAGGACTCTGGCCTATCT-GTTGGTCTGTGGGACCGGAGTAATGATTAAGAGGGGTAGTCGGGGGC 576
KX781330.1 P10       ATGGAATAACACGATAGGACTCTGGCCTATCT-GTTGGTCTGTGGGACCGGAGTAATGATTAAGAGGGGTAGTCGGGGGC 674
KX781332.1 P7        ATGGAATAACACGATAGGACTCTGGCCTATCT-GTTGGTCTGTGGGACCGGAGTAATGATTAAGAGGGGTAGTCGGGGGC 280
AB511837.1 KkS0801D2 ATGGAATAACACGATAGGACTCTGGCCTATCT-GTTGGTCTGTGGGACCGGAGTAATGATTAAGAGGGGTAGTCGGGGGC 828
AB511836.1 KkS0801B1 ATGGAATAACACGATAGGACTCTGGCCTATCT-GTTGGTCTGTGGGACCGGAGTAATGATTAAGAGGGGTAGTCGGGGGC 828
AB511835.1 SAG 11-32 ATGGAATAACACGATAGGACTCTGGCCTATCT-GTTGGTCTGTGGGACCGGAGTAATGATTAAGAGGGGTAGTCGGGGGC 828
AB511834.1 UTEX 90   ATGGAATAACACGATAGGACTCTGGCCTATCT-GTTGGTCTGTGGGACCGGAGTAATGATTAAGAGGGGTAGTCGGGGGC 828
KC166137.1 K01       ATGGAATAACACGATAGGACTCTGGCCTATCT-GTTGGTCTGTGGGACCGGAGTAATGATTAAGAGGGGTAGTCGGGGGC 660
KX781338.1 P1        ATGGAATAACACGATAGGACTCTGGCCTATCTTGTTGGTCTGTAGGACCGGAGTAATGATTAAGAGGGACAGTCGGGGGC 157
KF864473.1 JinCheon1 ATGGAATAACACGATAGGACTCTGGCCTATCT-GTTGTTCTGTGGGACCGGAGTAATGATTAAGAGGGGTAGTCGGGGGC 874
EU925397.1 CC-124    ATGGAATAACACGATAGGACTCTGGCCTATCT-GTTGGTCTGTGGGACCGGAGTAATGATTAAGAGGGGTAGTCGGGGGC 508
JN903974.1 SAG18.79  ATGGAATAACACGATAGGACTCTGGCCTATCT-GTTGGTCTGTGGGACCGGAGTAATGATTAAGAGGGGTAGTCGGGGGC 854
JN903978.1 SAG11-32c ATGGAATAACACGATAGGACTCTGGCCTATCT-GTTGGTCTGTGGGACCGGAGTAATGATTAAGAGGGGTAGTCGGGGGC 855
KX781325.1 P15       ATGGAATAACACGATAGGACTCTGGCCTATCT-GTTGGTCTGTGGGACCGGAGTAATGATTAAGAGGGGTAGTCGGGGGC 787
JN863299.1 KNUA021   ATGGAATAACACGATAGGACTCTGGCCTATCT-GTTGGTCTGTGGGACCGGAGTAATGATTAAGAGGGGTAGTCGGGGGC 857
KR904893.1 CBS152280 ATGGAATAACACGATAGGACTCTGGCCTATCT-GTTGGTCTGTGGGACCGGAGTAATGATTAAGAGGGGTAGTCGGGGGC 498
KX781335.1 P4        ATGGAATAACACGATAGGACTCTGGCCTATCT-GTTGGTCTGTGGGACCGGAGTAATGATTAAGAGGGGTAGTCGGGGGC 787
JX888472.1 CC-621    ATGGAATAACACGATAGGACTCTGGCCTATCT-GTTGGTCTGTGGGACCGGAGTAATGATTAAGAGGGGTAGTCGGGGGC 857
KC310450.1 RAC       -------------------------------------------------------------------------------- 0
JX888471.1 CC-620    ATGGAATAACACGATAGGACTCTGGCCTATCT-GTTGGTCTGTGGGACCGGAGTAATGATTAAGAGGGGTAGTCGGGGGC 857

KX781331.1 P8        ATTCGTATTTCATTGTCAGAGGTGAAATTCTTGGATTTATGAAAGACGAACTTCTGCGAAAGCATTTGCCAAGGATGTTT 919
KX781328.1 P12       ATTCGTATTCCGTTGTCAGAGGTGAAATTCTTGGATTTACGGAAGACGAACATCTGCGAAAGCATTTGCCAAGGATACTT 726
KX781321.1 P20       ATTCGTATTTCATTGTCAGAGGTGAAATTCTTGGATTTATGAAAGACGAACTTCTGCGAAAGCATTTGCCAAGGATGTTT 867
KX781327.1 P13       ATTCGTATTCCGTTGTCAGAGGTGAAATTCTTGGATTTACGGAAGACGAACA---------------------------- 699
KX781337.1 P2        ATTCGTATTTCATTGTCAGAGGTGAAATTCTTGGATTTATGAAAGACGAACTTCTGCGAAAGCATTTGCCAAGGATGTTT 587
JN903984.1 SAG53.72  ATTCGTATTTCATTGTCAGAGGTGAAATTCTTGGATTTATGAAAGACGAACTTCTGCGAAAGCATTTGCCAAGGATGTTT 937
```

```
KR904894.1 CC-849     ATTCGTATTCCGTTGTCAGAGGTGAAATTCTTGGATTTACGGAAGACGAACATCTGCGAAAGCATTTGCCAAGGATACTT 563
KC149968.1 GTD4C      ATTCGTATTCCGTTGTCAGAGGTGAAATTCTTGGATTTACGGAAGACGAACATCTGCGAAAGCATTTGCCAAGGATACTT 955
KX781333.1 P6         ATTCGTATTCCGTTGTCAGAGGTGAAATTCTTGGATTTACGGAAGACGAACATCTGCGAAAGCATTTGCCAAGGATACTT 797
AY665726.1 CC-1418    ATTCGTATTCCGTTGTCAGAGGTGAAATTCTTGGATTTACGGAAGACGAACATCTGCGAAAGCATTTGCCAAGGATACTT 914
AY665727.1 CC-1952    ATTCGTATTCCGTTGTCAGAGGTGAAATTCTTGGATTTACGGAAGACGAACATCTGCGAAAGCATTTGCCAAGGATACTT 908
AB701550.1 NIES-2235 ATTCGTATTCCGTTGTCAGAGGTGAAATTCTTGGATTTACGGAAGACGAACATCTGCGAAAGCATTTGCCAAGGATACTT 872
AB701554.1 NIES-2239 ATTCGTATTCCGTTGTCAGAGGTGAAATTCTTGGATTTACGGAAGACGAACATCTGCGAAAGCATTTGCCAAGGATACTT 854
AB701553.1 NIES-2238 ATTCGTATTCCGTTGTCAGAGGTGAAATTCTTGGATTTACGGAAGACGAACATCTGCGAAAGCATTTGCCAAGGATACTT 866
AB753040.1 PS-2708    ATTCGTATTCCGTTGTCAGAGGTGAAATTCTTGGATTTACGGAAGACGAACATCTGCGAAAGCATTTGCCAAGGATACTT 854
AB701552.1 NIES-2237 ATTCGTATTCCGTTGTCAGAGGTGAAATTCTTGGATTTACGGAAGACGAACATCTGCGAAAGCATTTGCCAAGGATACTT 865
AB701551.1 NIES-2236 ATTCGTATTCCGTTGTCAGAGGTGAAATTCTTGGATTTACGGAAGACGAACATCTGCGAAAGCATTTGCCAAGGATACTT 857
AB701555.1 NIES-2463 ATTCGTATTCCGTTGTCAGAGGTGAAATTCTTGGATTTACGGAAGACGAACATCTGCGAAAGCATTTGCCAAGGATACTT 868
KR092109.1 CC-125     ATTCGTATTCCGTTGTCAGAGGTGAAATTCTTGGATTTACGGAAGACGAACATCTGCGAAAGCATTTGCCAAGGATACTT 894
KX781322.1 P18        ATTCGTATTCCGTTGTCAGAGGTGAAATTCTTGGATTTACGGAAGACGAACATCTGCGAAAGCATTTGCCAAGGATACTT 724
KX781326.1 P14        ATTCGTATTCCGTTGTCAGAGGTGAAATTCTTGGATTTACGGAAGACGAACATCTGCGAAAGCATTTGCCAAGGATACTT 865
KX781329.1 P11        ATTCGTATTCCGTTGTCAGAGGTGAAATTCTTGGATTTACGGAAGACGAACATCTGCGAAAGCATTTGCCAAGGATACTT 656
KX781330.1 P10        ATTCGTATTCCGTTGTCAGAGGTGAAATTCTTGGATTTACGGAAGACGAACATCTGCGAAAGCATTTGCCAAGGATACTT 754
KX781332.1 P7         ATTCGTATTCCGTTGTCAGAGGTGAAATTCTTGGATTTACGGAAGACGAACATCTGCGAAAGCATTTGCCAAGGATACTT 360
AB511837.1 KkS0801D2 ATTCGTATTCCGTTGTCAGAGGTGAAATTCTTGGATTTACGGAAGACGAACATCTGCGAAAGCATTTGCCAAGGATACTT 908
AB511836.1 KkS0801B1 ATTCGTATTCCGTTGTCAGAGGTGAAATTCTTGGATTTACGGAAGACGAACATCTGCGAAAGCATTTGCCAAGGATACTT 908
AB511835.1 SAG 11-32 ATTCGTATTCCGTTGTCAGAGGTGAAATTCTTGGATTTACGGAAGACGAACATCTGCGAAAGCATTTGCCAAGGATACTT 908
AB511834.1 UTEX 90   ATTCGTATTCCGTTGTCAGAGGTGAAATTCTTGGATTTACGGAAGACGAACATCTGCGAAAGCATTTGCCAAGGATACTT 908
KC166137.1 K01        ATTCGTATTCCGTTGTCAGAGGTGAAATTCTTGGATTTACGGAAGACGAACATCTGCGAAAGCATTTGCCAAGGATACTT 740
KX781338.1 P1         ATTCGTATTTCATTGTCAGAGGTGAAATTCTTGGATTTATGAAAGACGAACTTCTGCGAAAGCATTTGCCAAGGATGTTT 237
KF864473.1 JinCheon1 ATTCGTATTCCGTTGTCAGAGGTGAAATTCTTGGATTTACGGAAGACGAACATCTGCGAAAGCATTTGCCAAGGATACTT 954
EU925397.1 CC-124     ATTCGTATTCCGTTGTCAGAGGTGAAATTCTTGGATTTACGGAAGACGAACATCTGCGAAAGCATTTGCCAAGGATACTT 588
JN903974.1 SAG18.79  ATTCGTATTCCGTTGTCAGAGGTGAAATTCTTGGATTTACGGAAGACGAACATCTGCGAAAGCATTTGCCAAGGATACTT 934
JN903978.1 SAG11-32c ATTCGTATTCCGTTGTCAGAGGTGAAATTCTTGGATTTACGGAAGACGAACATCTGCGAAAGCATTTGCCAAGGATACTT 935
KX781325.1 P15        ATTCGTATTCCGTTGTCAGAGGTGAAATTCTTGGATTTACGGAAGACGAACATCTGCGAAAGCATTTGCCAAGGATACTT 867
JN863299.1 KNUA021    ATTCGTATTCCGTTGTCAGAGGTGAAATTCTTGGATTTACGGAAGACGAACATCTGCGAAAGCATTTGCCAAGGATACTT 937
KR904893.1 CBS152280 ATTCGTATTCCGTTGTCAGAGGTGAAATTCTTGGATTTACGGAAGACGAACATCTGCGAAAGCATTTGCCAAGGATACTT 578
KX781335.1 P4         ATTCGTATTCCGTTGTCAGAGGTGAAATTCTTGGATTTACGGAAGACGAACATCTGCGAAAGCATTTGCCAAGGATACTT 867
JX888472.1 CC-621     ATTCGTATTCCGTTGTCAGAGGTGAAATTCTTGGATTTACGGAAGACGAACATCTGCGAAAGCATTTGCCAAGGATACTT 937
KC310450.1 RAC        -------------------------------------------------------------------------------- 0
JX888471.1 CC-620     ATTCGTATTCCGTTGTCAGAGGTGAAATTCTTGGATTTACGGAAGACGAACATCTGCGAAAGCATTTGCCAAGGATACTT 937

KX781331.1 P8         TCATTAATCAAGAACGAAAGTTGGGGGCTCGAAGACGATTAGATACCGTCGTAGTCTCAACCATAAACGATGCCGACTAG 999
KX781328.1 P12        TCATTGATCAAGAACGAAAGTTGGGGGCTCGAAGACGATTAGATACCGTCGTAGTCTCAACCATAAACGATGCCGACTAG 806
KX781321.1 P20        TCATTAATCAAGAACGAAAGTTGGGGGCTCGAAGACGATTAGATACCGTCGTAGTCTCAACCATAAACGATGCCGACTAG 947
KX781327.1 P13        ------------------------------------------TTACCGTCGTAGTCTCAACCATAAACGATGCCGACTAG 737
KX781337.1 P2         TCATTAATCAAGAACGAAAGTTGGGGGCTCGAAGACGATTAGATACCGTCGTAGTCTCAACCATAAACGATGCCGACTAG 667
JN903984.1 SAG53.72  TCATTAATCAAGAACGAAAGTTGGGGGCTCGAAGACGATTAGATACCGTCGTAGTCTCAACCATAAACGATGCCGACTAG 1017
KR904894.1 CC-849     TCATTGATCAAGAACGAAAGTTGGGGGCTCGAAGACGATTAGATACCGTCGTAGTCTCAACCATAAACGATGCCGACTAG 643
KC149968.1 GTD4C      TCATTGATCAAGAACGAAAGTTGGGGGCTCGAAGACGATTAGATACCGTCGTAGTCTCAACCATAAACGATGCCGACTAG 1035
KX781333.1 P6         TCATTGATCAAGAACGAAAGTTGGGGGCTCGAAGACGATTAGATACCGTCGTAGTCTCAACCATAAACGATGCCGACTAG 877
AY665726.1 CC-1418    TCATTGATCAAGAACGAAAGTTGGGGGCTCGAAGACGATTAGATACCGTCGTAGTCTCAACCATAAACGATGCCGACTAG 994
AY665727.1 CC-1952    TCATTGATCAAGAACGAAAGTTGGGGGCTCGAAGACGATTAGATACCGTCGTAGTCTCAACCATAAACGATGCCGACTAG 988
AB701550.1 NIES-2235 TCATTGATCAAGAACGAAAGTTGGGGGCTCGAAGACGATTAGATACCGTCGTAGTCTCAACCATAAACGATGCCGACTAG 952
AB701554.1 NIES-2239 TCATTGATCAAGAACGAAAGTTGGGGGCTCGAAGACGATTAGATACCGTCGTAGTCTCAACCATAAACGATGCCGACTAG 934
AB701553.1 NIES-2238 TCATTGATCAAGAACGAAAGTTGGGGGCTCGAAGACGATTAGATACCGTCGTAGTCTCAACCATAAACGATGCCGACTAG 946
AB753040.1 PS-2708    TCATTGATCAAGAACGAAAGTTGGGGGCTCGAAGACGATTAGATACCGTCGTAGTCTCAACCATAAACGATGCCGACTAG 934
AB701552.1 NIES-2237 TCATTGATCAAGAACGAAAGTTGGGGGCTCGAAGACGATTAGATACCGTCGTAGTCTCAACCATAAACGATGCCGACTAG 945
AB701551.1 NIES-2236 TCATTGATCAAGAACGAAAGTTGGGGGCTCGAAGACGATTAGATACCGTCGTAGTCTCAACCATAAACGATGCCGACTAG 937
AB701555.1 NIES-2463 TCATTGATCAAGAACGAAAGTTGGGGGCTCGAAGACGATTAGATACCGTCGTAGTCTCAACCATAAACGATGCCGACTAG 948
KR092109.1 CC-125     TCATTGATCAAGAACGAAAGTTGGGGGCTCGAAGACGATTAGATACCGTCGTAGTCTCAACCATAAACGATGCCGACTAG 974
KX781322.1 P18        TCATTGATCAAGAACGAAAGTTGGGGGCTCGAAGACGATTAGATACCGTCGTAGTCTCAACCATAAACGATGCCGACTAG 804
KX781326.1 P14        TCATTGATCAAGAACGAAAGTTGGGGGCTCGAAGACGATTAGATACCGTCGTAGTCTCAACCATAAACGATGCCGACTAG 945
KX781329.1 P11        TCATTGATCAAGAACGAAAGTTGGGGGCTCGAAGACGATTAGATACCGTCGTAGTCTCAACCATAAACGATGCCGACTAG 736
KX781330.1 P10        TCATTGATCAAGAACGAAAGTTGGGGGCTCGAAGACGATTAGATACCGTCGTAGTCTCAACCATAAACGATGCCGACTAG 834
KX781332.1 P7         TCATTGATCAAGAACGAAAGTTGGGGGCTCGAAGACGATTAGATACCGTCGTAGTCTCAACCATAAACGATGCCGACTAG 440
AB511837.1 KkS0801D2 TCATTGATCAAGAACGAAAGTTGGGGGCTCGAAGACGATTAGATACCGTCGTAGTCTCAACCATAAACGATGCCGACTAG 988
AB511836.1 KkS0801B1 TCATTGATCAAGAACGAAAGTTGGGGGCTCGAAGACGATTAGATACCGTCGTAGTCTCAACCATAAACGATGCCGACTAG 988
AB511835.1 SAG 11-32 TCATTGATCAAGAACGAAAGTTGGGGGCTCGAAGACGATTAGATACCGTCGTAGTCTCAACCATAAACGATGCCGACTAG 988
AB511834.1 UTEX 90   TCATTGATCAAGAACGAAAGTTGGGGGCTCGAAGACGATTAGATACCGTCGTAGTCTCAACCATAAACGATGCCGACTAG 988
KC166137.1 K01        TCATTGATCAAGAACGAAAGTTGGGGGCTCGAAGACGATTAGATACCGTCGTAGTCTCAACCATAAACGATGCCGACTAG 820
KX781338.1 P1         TCATTAATCAAGAACGAAAGTTGGGGGCTCGAAGACGATTAGATACCGTCGTAGTCTCAACCATAAACGATGCCGACTAG 317
KF864473.1 JinCheon1 TCATTGACCAAGAACGAAAGTTGGGGGCTCGAAGACGATTAGATACCGTCGTAGTCTCAACCATAAACGATGCCGACTAG 1034
EU925397.1 CC-124     TCATTGATCAAGAACGAAAGTTGGGGGCTCGAAGACGATTAGATACCGTCGTAGTCTCAACCATAAACGATGCCGACTAG 668
JN903974.1 SAG18.79  TCATTGATCAAGAACGAAAGTTGGGGGCTCGAAGACGATTAGATACCGTCGTAGTCTCAACCATAAACGATGCCGACTAG 1014
JN903978.1 SAG11-32c TCATTGATCAAGAACGAAAGTTGGGGGCTCGAAGACGATTAGATACCGTCGTAGTCTCAACCATAAACGATGCCGACTAG 1015
KX781325.1 P15        TCATTGATCAAGAACGAAAGTTGGGGGCTCGAAGACGATTAGATACCGTCGTAGTCTCAACCATAAACGATGCCGACTAG 947
JN863299.1 KNUA021    TCATTGATCAAGAACGAAAGTTGGGGGCTCGAAGACGATTAGATACCGTCGTAGTCTCAACCATAAACGATGCCGACTAG 1017
KR904893.1 CBS152280 TCATTGATCAAGAACGAAAGTTGGGGGCTCGAAGACGATTAGATACCGTCGTAGTCTCAACCATAAACGATGCCGACTAG 658
KX781335.1 P4         TCATTGATCAAGAACGAAAGTTGGGGGCTCGAAGACGATTAGATACCGTCGTAGTCTCAACCATAAACGATGCCGACTAG 947
JX888472.1 CC-621     TCATTGATCAAGAACGAAAGTTGGGGGCTCGAAGACGATTAGATACCGTCGTAGTCTCAACCATAAACGATGCCGACTAG 1017
KC310450.1 RAC        -------------------------------------------------------------------------------- 0
JX888471.1 CC-620     TCATTGATCAAGAACGAAAGTTGGGGGCTCGAAGACGATTAGATACCGTCGTAGTCTCAACCATAAACGATGCCGACTAG 1017

KX781331.1 P8         GGATTGGCAGATGTTTCATTGATGACTCTGCCAGCACCTTATGAGAAATCAAAGTTTTTGGGTTCCGGGGGGAGTATGGT 1079
KX781328.1 P12        GGATTGGCAGATGTTCTTTTGATGACTCTGCCAGCACCTTATGAGAAATCAAAGTTTTTGGGTTCCGGGGGGAGTATGGT 886
KX781321.1 P20        GGATTGGCAGATGTTTCATTGATGACTCTGCCAGCACCTTATGAGAAATCAAAGTTTTTGGGTTCCGGGGGGAGTATGGT 1027
KX781327.1 P13        GGATTGGCAGATGTTCTTTTGATGACTCTGCCAGCACCTTATGAGAAATCAAAGTTTTTGGGTTCCGGGGGGAGTATGGT 817
KX781337.1 P2         GGATTGGCAGATGTTTCATTGATGACTCTGCCAGCACCTTATGAGAAATCAAAGTTTTTGGGTTCCGGGGGGAGTATGGT 747
JN903984.1 SAG53.72  GGATTGGCAGATGTTTCATTGATGACTCTGCCAGCACCTTATGAGAAATCAAAGTTTTTGGGTTCCGGGGGGAGTATGGT 1097
KR904894.1 CC-849     GGATTGGCAGATGTTCTTTTGATGACTCTGCCAGCACCTTATGAGAAATCAAAGTTTTTGGGTTCCGGGGGGAGTATGGT 723
KC149968.1 GTD4C      GGATTGGCAGATGTTCTTTTGATGACTCTGCCAGCACCTTATGAGAAATCAAAGTTTTTGGGTTCCGGGGGGAGTATGGT 1115
KX781333.1 P6         GGATTGGCAGATGTTCTTTTGATGACTCTGCCAGCACCTTATGAGAAATCAAAGTTTTTGGGTTCCGGGGGGAGTATGGT 957
AY665726.1 CC-1418    GGATTGGCAGATGTTCTTTTGATGACTCTGCCAGCACCTTATGAGAAATCAAAGTTTTTGGGTTCCGGGGGGAGTATGGT 1074
AY665727.1 CC-1952    GGATTGGCAGATGTTCTTTTGATGACTCTGCCAGCACCTTATGAGAAATCAAAGTTTTTGGGTTCCGGGGGGAGTATGGT 1068
AB701550.1 NIES-2235 GGATTGGCAGATGTTCTTTTGATGACTCTGCCAGCACCTTATGAGAAATCAAAGTTTTTGGGTTCCGGGGGGAGTATGGT 1032
AB701554.1 NIES-2239 GGATTGGCAGATGTTCTTTTGATGACTCTGCCAGCACCTTATGAGAAATCAAAGTTTTTGGGTTCCGGGGGGAGTATGGT 1014
AB701553.1 NIES-2238 GGATTGGCAGATGTTCTTTTGATGACTCTGCCAGCACCTTATGAGAAATCAAAGTTTTTGGGTTCCGGGGGGAGTATGGT 1026
AB753040.1 PS-2708    GGATTGGCAGATGTTCTTTTGATGACTCTGCCAGCACCTTATGAGAAATCAAAGTTTTTGGGTTCCGGGGGGAGTATGGT 1014
AB701552.1 NIES-2237 GGATTGGCAGATGTTCTTTTGATGACTCTGCCAGCACCTTATGAGAAATCAAAGTTTTTGGGTTCCGGGGGGAGTATGGT 1025
```

```
AB701551.1 NIES-2236 GGATTGGCAGATGTTCTTTTGATGACTCTGCCAGCACCTTATGAGAAATCAAAGTTTTTGGGTTCCGGGGGGAGTATGGT 1017
AB701555.1 NIES-2463 GGATTGGCAGATGTTCTTTTGATGACTCTGCCAGCACCTTATGAGAAATCAAAGTTTTTGGGTTCCGGGGGGAGTATGGT 1028
KR092109.1 CC-125    GGATTGGCAGATGTTCTTTTGATGACTCTGCCAGCACCTTATGAGAAATCAAAGTTTTTGGGTTCCGGGGGGAGTATGGT 1054
KX781322.1 P18       GGATTGGCAGATGTTCTTTTGATGACTCTGCCAGCACCTTATGAGAAATCAAAGTTTTTGGGTTCCGGGGGGAGTATGGT 884
KX781326.1 P14       GGATTGGCAGATGTTCTTTTGATGACTCTGCCAGCACCTTATGAGAAATCAAAGTTTTTGGGTTCCGGGGGGAGTATGGT 1025
KX781329.1 P11       GGATTGGCAGATGTTCTTTTGATGACTCTGCCAGCACCTTATGAGAAATCAAAGTTTTTGGGTTCCGGGGGGAGTATGGT 816
KX781330.1 P10       GGATTGGCAGATGTTCTTTTGATGACTCTGCCAGCACCTTATGAGAAATCAAAGTTTTTGGGTTCCGGGGGGAGTATGGT 914
KX781332.1 P7        GGATTGGCAGATGTTCTTTTGATGACTCTGCCAGCACCTTATGAGAAATCAAAGTTTTTGGGTTCCGGGGGGAGTATGGT 520
AB511837.1 KkS0801D2 GGATTGGCAGATGTTCTTTTGATGACTCTGCCAGCACCTTATGAGAAATCAAAGTTTTTGGGTTCCGGGGGGAGTATGGT 1068
AB511836.1 KkS0801B1 GGATTGGCAGATGTTCTTTTGATGACTCTGCCAGCACCTTATGAGAAATCAAAGTTTTTGGGTTCCGGGGGGAGTATGGT 1068
AB511835.1 SAG 11-32 GGATTGGCAGATGTTCTTTTGATGACTCTGCCAGCACCTTATGAGAAATCAAAGTTTTTGGGTTCCGGGGGGAGTATGGT 1068
AB511834.1 UTEX 90   GGATTGGCAGATGTTCTTTTGATGACTCTGCCAGCACCTTATGAGAAATCAAAGTTTTTGGGTTCCGGGGGGAGTATGGT 1068
KC166137.1 K01       GGATTGGCAGATGTTCTTTTGATGACTCTGCCAGCACCTTATGAGAAATCAAAGTTTTTGGGTTCCGGGGGGAGTATGGT 900
KX781338.1 P1        GGATTGGCAGATGTTTCATTGATGACTCTGCCAGCACCTTATGAGAAATCAAAGTTTTTGGGTTCCGGGGGGAGTATGGT 397
KF864473.1 JinCheon1 GGATTGGCAGATGTTCCTTTGATGACTCTGCCAGCACCTTATGAGAAATCAAAGTTTTTGGGTTCCGGGGGGAGTATGGT 1114
EU925397.1 CC-124    GGATTGGCAGATGTTCTTTTGATGACTCTGCCAGCACCTTATGAGAAATCAAAGTTTTTGGGTTCCGGGGGGAGTATGGT 748
JN903974.1 SAG18.79  GGATTGGCAGATGTTCTTTTGATGACTCTGCCAGCACCTTATGAGAAATCAAAGTTTTTGGGTTCCGGGGGGAGTATGGT 1094
JN903978.1 SAG11-32c GGATTGGCAGATGTTCTTTTGATGACTCTGCCAGCACCTTATGAGAAATCAAAGTTTTTGGGTTCCGGGGGGAGTATGGT 1095
KX781325.1 P15       GGATTGGCAGATGTTCTTTTGATGACTCTGCCAGCACCTTATGAGAAATCAAAGTTTTTGGGTTCCGGGGGGAGTATGGT 1027
JN863299.1 KNUA021   GGATTGGCAGATGTTCTTTTGATGACTCTGCCAGCACCTTATGAGAAATCAAAGTTTTTGGGTTCCGGGGGGAGTATGGT 1097
KR904893.1 CBS152280 GGATTGGCAGATGTTCTTTTGATGACTCTGCCAGCACCTTATGAGAAATCAAAGTTTTTGGGTTCCGGGGGGAGTATGGT 738
KX781335.1 P4        GGATTGGCAGATGTTCTTTTGATGACTCTGCCAGCACCTTATGAGAAATCAAAGTTTTTGGGTTCCGGGGGGAGTATGGT 1027
JX888472.1 CC-621    GGATTGGCAGATGTTCTTTTGATGACTCTGCCAGCACCTTATGAGAAATCAAAGTTTTTGGGTTCCGGGGGGAGTATGGT 1097
KC310450.1 RAC       -------------------------------------------------------------------------------- 0
JX888471.1 CC-620    GGATTGGCAGATGTTCTTTTGATGACTCTGCCAGCACCTTATGAGAAATCAAAGTTTTTGGGTTCCGGGGGGAGTATGGT 1097

KX781331.1 P8        CGCAAGGCTGAAACTTAAAGGAATTGACGGAAGGGCACCACCAGGCGTGGAGCCTGCGGCTTAATTTGACTCAACACGGG 1159
KX781328.1 P12       CGCAAGGCTGAAACTTAAAGGAATTGACGGAAGGGCACCACCAGGCGTGGAGCCTGCGGCTTAATTTGACTCAACACGGG 966
KX781321.1 P20       CGCAAGGCTGAAACTTAAAGGAATTGACGGAAGGGCACCACCAGGCGTGGAGCCTGCGGCTTAATTTGACTCAACACGGG 1107
KX781327.1 P13       CGCAAGGCTGAAACTTAAAGGAATTGACGGAAGGGCACCACCAGGCGTGGAGCCTGCGGCTTAATTTGACTCAACACGGG 897
KX781337.1 P2        CGCAAGGCTGAAACTTAAAGGAATTGACGGAAGGGCACCACCAGGCGTGGAGCCTGCGGCTTAATTTGACTCAACACGGG 827
JN903984.1 SAG53.72  CGCAAGGCTGAAACTTAAAGGAATTGACGGAAGGGCACCACCAGGCGTGGAGCCTGCGGCTTAATTTGACTCAACACGGG 1177
KR904894.1 CC-849    CGCAAGGCTGAAACTTAAAGGAATTGACGGAAGGGCACCACCAGGCGTGGAGCCTGCGGCTTAATTTGACTCAACACGGG 803
KC149968.1 GTD4C     CGCAAGGCTGAAACTTAAAGGAATTGACGGAAGGGCACCACCAGGCGTGGAGCCTGCGGCTTAATTTGACTCAACACGGG 1195
KX781333.1 P6        CGCAAGGCTGAAACTTAAAGGAATTGACGGAAGGGCACCACCAGGCGTGGAGCCTGCGGCTTAATTTGACTCAACACGGG 1037
AY665726.1 CC-1418   CGCAAGGCTGAAACTTAAAGGAATTGACGGAAGGGCACCACCAGGCGTGGAGCCTGCGGCTTAATTTGACTCAACACGGG 1154
AY665727.1 CC-1952   CGCAAGGCTGAAACTTAAAGGAATTGACGGAAGGGCACCACCAGGCGTGGAGCCTGCGGCTTAATTTGACTCAACACGGG 1148
AB701550.1 NIES-2235 CGCAAGGCTGAAACTTAAAGGAATTGACGGAAGGGCACCACCAGGCGTGGAGCCTGCGGCTTAATTTGACTCAACACGGG 1112
AB701554.1 NIES-2239 CGCAAGGCTGAAACTTAAAGGAATTGACGGAAGGGCACCACCAGGCGTGGAGCCTGCGGCTTAATTTGACTCAACACGGG 1094
AB701553.1 NIES-2238 CGCAAGGCTGAAACTTAAAGGAATTGACGGAAGGGCACCACCAGGCGTGGAGCCTGCGGCTTAATTTGACTCAACACGGG 1106
AB753040.1 PS-2708   CGCAAGGCTGAAACTTAAAGGAATTGACGGAAGGGCACCACCAGGCGTGGAGCCTGCGGCTTAATTTGACTCAACACGGG 1094
AB701552.1 NIES-2237 CGCAAGGCTGAAACTTAAAGGAATTGACGGAAGGGCACCACCAGGCGTGGAGCCTGCGGCTTAATTTGACTCAACACGGG 1105
AB701551.1 NIES-2236 CGCAAGGCTGAAACTTAAAGGAATTGACGGAAGGGCACCACCAGGCGTGGAGCCTGCGGCTTAATTTGACTCAACACGGG 1097
AB701555.1 NIES-2463 CGCAAGGCTGAAACTTAAAGGAATTGACGGAAGGGCACCACCAGGCGTGGAGCCTGCGGCTTAATTTGACTCAACACGGG 1108
KR092109.1 CC-125    CGCAAGGCTGAAACTTAAAGGAATTGACGGAAGGGCACCACCAGGCGTGGAGCCTGCGGCTTAATTTGACTCAACACGGG 1134
KX781322.1 P18       CGCAAGGCTGAAACTTAAAGGAATTGACGGAAGGGCACCACCAGGCGTGGAGCCTGCGGCTTAATTTGACTCAACACGGG 964
KX781326.1 P14       CGCAAGGCTGAAACTTAAAGGAATTGACGGAAGGGCACCACCAGGCGTGGAGCCTGCGGCTTAATTTGACTCAACACGGG 1105
KX781329.1 P11       CGCAAGGCTGAAACTTAAAGGAATTGACGGAAGGGCACCACCAGGCGTGGAGCCTGCGGCTTAATTTGACTCAACACGGG 896
KX781330.1 P10       CGCAAGGCTGAAACTTAAAGGAATTGACGGAAGGGCACCACCAGGCGTGGAGCCTGCGGCTTAATTTGACTCAACACGGG 994
KX781332.1 P7        CGCAAGGCTGAAACTTAAAGGAATTGACGGAAGGGCACCACCAGGCGTGGAGCCTGCGGCTTAATTTGACTCAACACGGG 600
AB511837.1 KkS0801D2 CGCAAGGCTGAAACTTAAAGGAATTGACGGAAGGGCACCACCAGGCGTGGAGCCTGCGGCTTAATTTGACTCAACACGGG 1148
AB511836.1 KkS0801B1 CGCAAGGCTGAAACTTAAAGGAATTGACGGAAGGGCACCACCAGGCGTGGAGCCTGCGGCTTAATTTGACTCAACACGGG 1148
AB511835.1 SAG 11-32 CGCAAGGCTGAAACTTAAAGGAATTGACGGAAGGGCACCACCAGGCGTGGAGCCTGCGGCTTAATTTGACTCAACACGGG 1148
AB511834.1 UTEX 90   CGCAAGGCTGAAACTTAAAGGAATTGACGGAAGGGCACCACCAGGCGTGGAGCCTGCGGCTTAATTTGACTCAACACGGG 1148
KC166137.1 K01       CGCAAGGCTGAAACTTAAAGGAATTGACGGAAGGGCACCACCAGGCGTGGAGCCTGCGGCTTAATTTGACTCAACACGGG 980
KX781338.1 P1        CGCAAGGCTGAAACTTAAAGGAATTGACGGAAGGGCACCACCAGGCGTGGAGCCTGCGGCTTAATTTGACTCAACACGGG 477
KF864473.1 JinCheon1 CGCAAGGCTGAAACTTAAAGGAATTGACGGAAGGGCACCACCAGGCGTGGAGCCTGCGGCTTAATTTGACTCAACACGGG 1194
EU925397.1 CC-124    CGCAAGGCTGAAACTTAAAGGAATTGACGGAAGGGCACCACCAGGCGTGGAGCCTGCGGCTTAATTTGACTCAACACGGG 828
JN903974.1 SAG18.79  CGCAAGGCTGAAACTTAAAGGAATTGACGGAAGGGCACCACCAGGCGTGGAGCCTGCGGCTTAATTTGACTCAACACGGG 1174
JN903978.1 SAG11-32c CGCAAGGCTGAAACTTAAAGGAATTGACGGAAGGGCACCACCAGGCGTGGAGCCTGCGGCTTAATTTGACTCAACACGGG 1175
KX781325.1 P15       CGCAAGGCTGAAACTTAAAGGAATTGACGGAAGGGCACCACCAGGCGTGGAGCCTGCGGCTTAATTTGACTCAACACGGG 1107
JN863299.1 KNUA021   CGCAAGGCTGAAACTTAAAGGAATTGACGGAAGGGCACCACCAGGCGTGGAGCCTGCGGCTTAATTTGACTCAACACGGG 1177
KR904893.1 CBS152280 CGCAAGGCTGAAACTTAAAGGAATTGACGGAAGGGCACCACCAGGCGTGGAGCCTGCGGCTTAATTTGACTCAACACGGG 818
KX781335.1 P4        CGCAAGGCTGAAACTTAAAGGAATTGACGGAAGGGCACCACCAGGCGTGGAGCCTGCGGCTTAATTTGACTCAACACGGG 1107
JX888472.1 CC-621    CGCAAGGCTGAAACTTAAAGGAATTGACGGAAGGGCACCACCAGGCGTGGAGCCTGCGGCTTAATTTGACTCAACACGGG 1177
KC310450.1 RAC       -------------------------------------------------------------------------------- 0
JX888471.1 CC-620    CGCAAGGCTGAAACTTAAAGGAATTGACGGAAGGGCACCACCAGGCGTGGAGCCTGCGGCTTAATTTGACTCAACACGGG 1177

KX781331.1 P8        AAAACTTACCAGGTCCAGACACGGGAAGGATTGACAGATTGAGAGCTCTTTCTTGATTCTGTGGGTGGTGGTGCATGGCC 1239
KX781328.1 P12       GAAACTTACCAGGTCCAGACACGGGAAGGATTGACAGATTGAGAGCTCTTTCTTGATTCTGTGGGTGGTGGTGCATGGCC 1046
KX781321.1 P20       AAAACTTACCAGGTCCAGACACGGGAAGGATTGACAGATTGAGAGCTCTTTCTTGATTCTGTGGGTGGTGGTGCATGGCC 1187
KX781327.1 P13       GAAACTTACCAGGTCCAGACACGGGAAGGATTGACAGATTGAGAGCTCTTTCTTGATTCTGTGGGTGGTGGTGCATGGCC 977
KX781337.1 P2        AAAACTTACCAGGTCCAGACACGGGAAGGATTGACAGATTGAGAGCTCTTTCTTGATTCTGTGGGTGGTGGTGCATGGCC 907
JN903984.1 SAG53.72  AAAACTTACCAGGTCCAGACACGGGAAGGATTGACAGATTGAGAGCTCTTTCTTGATTCTGTGGGTGGTGGTGCATGGCC 1257
KR904894.1 CC-849    GAAACTTACCAGGTCCAGACACGGGAAGGATTGACAGATTGAGAGCTCTTTCTTGATTCTGTGGGTGGTGGTGCATGGCC 883
KC149968.1 GTD4C     GAAACTTACCAGGTCCAGACACGGGAAGGATTGACAGATTGAGAGCTCTTTCTTGATTCTGTGGGTGGTGGTGCATGGCC 1275
KX781333.1 P6        GAAACTTACCAGGTCCAGACACGGGAAGGATTGACAGATTGAGAGCTCTTTCTTGATTCTGTGGGTGGTGGTGCATGGCC 1117
AY665726.1 CC-1418   GAAACTTACCAGGTCCAGACACGGGAAGGATTGACAGATTGAGAGCTCTTTCTTGATTCTGTGGGTGGTGGTGCATGGCC 1234
AY665727.1 CC-1952   GAAACTTACCAGGTCCAGACACGGGAAGGATTGACAGATTGAGAGCTCTTTCTTGATTCTGTGGGTGGTGGTGCATGGCC 1228
AB701550.1 NIES-2235 GAAACTTACCAGGTCCAGACACGGGAAGGATTGACAGATTGAGAGCTCTTTCTTGATTCTGTGGGTGGTGGTGCATGGCC 1192
AB701554.1 NIES-2239 GAAACTTACCAGGTCCAGACACGGGAAGGATTGACAGATTGAGAGCTCTTTCTTGATTCTGTGGGTGGTGGTGCATGGCC 1174
AB701553.1 NIES-2238 GAAACTTACCAGGTCCAGACACGGGAAGGATTGACAGATTGAGAGCTCTTTCTTGATTCTGTGGGTGGTGGTGCATGGCC 1186
AB753040.1 PS-2708   GAAACTTACCAGGTCCAGACACGGGAAGGATTGACAGATTGAGAGCTCTTTCTTGATTCTGTGGGTGGTGGTGCATGGCC 1174
AB701552.1 NIES-2237 GAAACTTACCAGGTCCAGACACGGGAAGGATTGACAGATTGAGAGCTCTTTCTTGATTCTGTGGGTGGTGGTGCATGGCC 1185
AB701551.1 NIES-2236 GAAACTTACCAGGTCCAGACACGGGAAGGATTGACAGATTGAGAGCTCTTTCTTGATTCTGTGGGTGGTGGTGCATGGCC 1177
AB701555.1 NIES-2463 GAAACTTACCAGGTCCAGACACGGGAAGGATTGACAGATTGAGAGCTCTTTCTTGATTCTGTGGGTGGTGGTGCATGGCC 1188
KR092109.1 CC-125    GAAACTTACCAGGTCCAGACACGGGAAGGATTGACAGATTGAGAGCTCTTTCTTGATTCTGTGGGTGGTGGTGCATGGCC 1214
KX781322.1 P18       GAAACTTACCAGGTCCAGACACGGGAAGGATTGACAGATTGAGAGCTCTTTCTTGATTCTGTGGGTGGTGGTGCATGGCC 1044
KX781326.1 P14       GAAACTTACCAGGTCCAGACACGGGAAGGATTGACAGATTGAGAGCTCTTTCTTGATTCTGTGGGTGGTGGTGCATGGCC 1185
KX781329.1 P11       GAAACTTACCAGGTCCAGACACGGGAAGGATTGACAGATTGAGAGCTCTTTCTTGATTCTGTGGGTGGTGGTGCATGGCC 976
KX781330.1 P10       GAAACTTACCAGGTCCAGACACGGGAAGGATTGACAGATTGAGAGCTCTTTCTTGATTCTGTGGGTGGTGGTGCATGGCC 1074
KX781332.1 P7        GAAACTTACCAGGTCCAGACACGGGAAGGATTGACAGATTGAGAGCTCTTTCTTGATTCTGTGGGTGGTGGTGCATGGCC 680
AB511837.1 KkS0801D2 GAAACTTACCAGGTCCAGACACGGGAAGGATTGACAGATTGAGAGCTCTTTCTTGATTCTGTGGGTGGTGGTGCATGGCC 1228
AB511836.1 KkS0801B1 GAAACTTACCAGGTCCAGACACGGGAAGGATTGACAGATTGAGAGCTCTTTCTTGATTCTGTGGGTGGTGGTGCATGGCC 1228
```

```
AB511835.1 SAG 11-32 GAAACTTACCAGGTCCAGACACGGGAAGGATTGACAGATTGAGAGCTCTTTCTTGATTCTGTGGGTGGTGGTGCATGGCC 1228
AB511834.1 UTEX 90   GAAACTTACCAGGTCCAGACACGGGAAGGATTGACAGATTGAGAGCTCTTTCTTGATTCTGTGGGTGGTGGTGCATGGCC 1228
KC166137.1 K01       GAAACTTACCAGGTCCAGACACGGGAAGGATTGACAGATTGAGAGCTCTTTCTTGATTCTGTGGGTGGTGGTGCATGGCC 1060
KX781338.1 P1        AAAACTTACCAGGTCCAGACACGGGAAGGATTGACAGATTGAGAGCTCTTTCTTGATTCTGTGGGTGGTGGTGCATGGCC 557
KF864473.1 JinCheon1 GAAACTTACCAGGTCCAGACACGGGAAGGATTGACAGATTGAGAGCTCTTTCTTGATTCTGTGGGTGGTGGTGCATGGCC 1274
EU925397.1 CC-124    GAAACTTACCAGGTCCAGACACGGGAAGGATTGACAGATTGAGAGCTCTTTCTTGATTCTGTGGGTGGTGGTGCATGGCC 908
JN903974.1 SAG18.79  GAAACTTACCAGGTCCAGACACGGGAAGGATTGACAGATTGAGAGCTCTTTCTTGATTCTGTGGGTGGTGGTGCATGGCC 1254
JN903978.1 SAG11-32c GAAACTTACCAGGTCCAGACACGGGAAGGATTGACAGATTGAGAGCTCTTTCTTGATTCTGTGGGTGGTGGTGCATGGCC 1255
KX781325.1 P15       GAAACTTACCAGGTCCAGACACGGGAAGGATTGACAGATTGAGAGCTCTTTCTTGATTCTGTGGGTGGTGGTGCATGGCC 1187
JN863299.1 KNUA021   GAAACTTACCAGGTCCAGACACGGGAAGGATTGACAGATTGAGAGCTCTTTCTTGATTCTGTGGGTGGTGGTGCATGGCC 1257
KR904893.1 CBS152280 GAAACTTACCAGGTCCAGACACGGGAAGGATTGACAGATTGAGAGCTCTTTCTTGATTCTGTGGGTGGTGGTGCATGGCC 898
KX781335.1 P4        GAAACTTACCAGGTCCAGACACGGGAAGGATTGACAGATTGAGAGCTCTTTCTTGATTCTGTGGGTGGTGGTGCATGGCC 1187
JX888472.1 CC-621    GAAACTTACCAGGTCCAGACACGGGAAGGATTGACAGATTGAGAGCTCTTTCTTGATTCTGTGGGTGGTGGTGCATGGCC 1257
KC310450.1 RAC       -----------------------------------------------------------------------TGCATGGCC 9
JX888471.1 CC-620    GAAACTTACCAGGTCCAGACACGGGAAGGATTGACAGATTGAGAGCTCTTTCTTGATTCTGTGGGTGGTGGTGCATGGCC 1257

KX781331.1 P8        GTTCTTAGTTGGTGGGTTGCCTTGTCAGGTTGATTCCGGTAACGAACGAGACCTCAGCCTGCTAAATAGTCACGACTGCT 1319
KX781328.1 P12       GTTCTTAGTTGGTGGGTTGCCTTGTCAGGTTGATTCCGGTAACGAACGAGACCTCAGCCTGCTAAATAGTCAGCATCGCA 1126
KX781321.1 P20       GTTCTTAGTTGGTGGGTTGCCTTGTCAGGTTGATTCCGGTAACGAACGAGACCTCAGCCTGCTAAATAGTCACGACTGCT 1267
KX781327.1 P13       GTTCTTAGTTGGTGGGTTGCCTTGTCAGGTTGATTCCGGTAACGAACGAGACCTCAGCCTGCTAAATAGTCAGCATCGCA 1057
KX781337.1 P2        GTTCTTAGTTGGTGGGTTGCCTTGTCAGGTTGATTCCGGTAACGAACGAGACCTCAGCCTGCTAAATAGTCACGACTGCT 987
JN903984.1 SAG53.72  GTTCTTAGTTGGTGGGTTGCCTTGTCAGGTTGATTCCGGTAACGAACGAGACCTCAGCCTGCTAAATAGTCACGACTGCT 1337
KR904894.1 CC-849    GTTCTTAGTTGGTGGGTTGCCTTGTCAGGTTGATTCCGGTAACAAACGAGACCTCAGCCTGCTAAATAGTCAGCATCGCA 963
KC149968.1 GTD4C     GTTCTTAGTTGGTGGGTTGCCTTGTCAGGTTGATTCCGGTAACGAACGAGACCTCAGCCTGCTAAATAGTCAGCATCGCA 1355
KX781333.1 P6        GTTCTTAGTTGGTGGGTTGCCTTGTCAGGTTGATTCCGGTAACGAACGAGACCTCAGCCTGCTAAATAGTCAGCATCGCA 1197
AY665726.1 CC-1418   GTTCTTAGTTGGTGGGTTGCCTTGTCAGGTTGATTCCGGTAACGAACGAGACCTCAGCCTGCTAAATAGTCAGCATCGCA 1314
AY665727.1 CC-1952   GTTCTTAGTTGGTGGGTTGCCTTGTCAGGTTGATTCCGGTAACGAACGAGACCTCAGCCTGCTAAATAGTCAGCATCGCA 1308
AB701550.1 NIES-2235 GTTCTTAGTTGGTGGGTTGCCTTGTCAGGTTGATTCCGGTAACGAACGAGACCTCAGCCTGCTAAATAGTCAGCATCGCA 1272
AB701554.1 NIES-2239 GTTCTTAGTTGGTGGGTTGCCTTGTCAGGTTGATTCCGGTAACGAACGAGACCTCAGCCTGCTAAATAGTCAGCATCGCA 1254
AB701553.1 NIES-2238 GTTCTTAGTTGGTGGGTTGCCTTGTCAGGTTGATTCCGGTAACGAACGAGACCTCAGCCTGCTAAATAGTCAGCATCGCA 1266
AB753040.1 PS-2708   GTTCTTAGTTGGTGGGTTGCCTTGTCAGGTTGATTCCGGTAACGAACGAGACCTCAGCCTGCTAAATAGTCAGCATCGCA 1254
AB701552.1 NIES-2237 GTTCTTAGTTGGTGGGTTGCCTTGTCAGGTTGATTCCGGTAACGAACGAGACCTCAGCCTGCTAAATAGTCAGCATCGCA 1265
AB701551.1 NIES-2236 GTTCTTAGTTGGTGGGTTGCCTTGTCAGGTTGATTCCGGTAACGAACGAGACCTCAGCCTGCTAAATAGTCAGCATCGCA 1257
AB701555.1 NIES-2463 GTTCTTAGTTGGTGGGTTGCCTTGTCAGGTTGATTCCGGTAACGAACGAGACCTCAGCCTGCTAAATAGTCAGCATCGCA 1268
KR092109.1 CC-125    GTTCTTAGTTGGTGGGTTGCCTTGTCAGGTTGATTCCGGTAACGAACGAGACCTCAGCCTGCTAAATAGTCAGCATCGCA 1294
KX781322.1 P18       GTTCTTAGTTGGTGGGTTGCCTTGTCAGGTTGATTCCGGTAACGAACGAGACCTCAGCCTGCTAAATAGTCAGCATCGCA 1124
KX781326.1 P14       GTTCTTAGTTGGTGGGTTGCCTTGTCAGGTTGATTCCGGTAACGAACGAGACCTCAGCCTGCTAAATAGTCAGCATCGCA 1265
KX781329.1 P11       GTTCTTAGTTGGTGGGTTGCCTTGTCAGGTTGATTCCGGTAACGAACGAGACCTCAGCCTGCTAAATAGTCAGCATCGCA 1056
KX781330.1 P10       GTTCTTAGTTGGTGGGTTGCCTTGTCAGGTTGATTCCGGTAACGAACGAGACCTCAGCCTGCTAAATAGTCAGCATCGCA 1154
KX781332.1 P7        GTTCTTAGTTGGTGGGTTGCCTTGTCAGGTTGATTCCGGTAACGAACGAGACCTCAGCCTGCTAAATAGTCAGCATCGCA 760
AB511837.1 KkS0801D2 GTTCTTAGTTGGTGGGTTGCCTTGTCAGGTTGATTCCGGTAACGAACGAGACCTCAGCCTGCTAAATAGTCAGCATCGCA 1308
AB511836.1 KkS0801B1 GTTCTTAGTTGGTGGGTTGCCTTGTCAGGTTGATTCCGGTAACGAACGAGACCTCAGCCTGCTAAATAGTCAGCATCGCA 1308
AB511835.1 SAG 11-32 GTTCTTAGTTGGTGGGTTGCCTTGTCAGGTTGATTCCGGTAACGAACGAGACCTCAGCCTGCTAAATAGTCAGCATCGCA 1308
AB511834.1 UTEX 90   GTTCTTAGTTGGTGGGTTGCCTTGTCAGGTTGATTCCGGTAACGAACGAGACCTCAGCCTGCTAAATAGTCAGCATCGCA 1308
KC166137.1 K01       GTTCTTAGTTGGTGGGTTGCCTTGTCAGGTTGATTCCGGTAACGAACGAGACCTCAGCCTGCTAAATAGTCAGCATCGCA 1140
KX781338.1 P1        GTTCTTAGTTGGTGGGTTGCCTTGTCAGGTTGATTCCGGTAACGAACGAGACCTCAGCCTGCTAAATAGTCACGACTGCT 637
KF864473.1 JinCheon1 GTTCTTAGTTGGTGGGTTGCCTTGTCAGGTTGATTCCGGTAACGAACGAGACCTCAGCCTGCTAAATAGTCAGCATCGCA 1354
EU925397.1 CC-124    GTTCTTAGTTGGTGGGTTGCCTTGTCAGGTTGATTCCGGTAACGAACGAGACCTCAGCCTGCTAAATAGTCAGCATCGCA 988
JN903974.1 SAG18.79  GTTCTTAGTTGGTGGGTTGCCTTGTCAGGTTGATTCCGGTAACGAACGAGACCTCAGCCTGCTAAATAGTCAGCATCGCA 1334
JN903978.1 SAG11-32c GTTCTTAGTTGGTGGGTTGCCTTGTCAGGTTGATTCCGGTAACGAACGAGACCTCAGCCTGCTAAATAGTCAGCATCGCA 1335
KX781325.1 P15       GTTCTTAGTTGGTGGGTTGCCTTGTCAGGTTGATTCCGGTAACGAACGAGACCTCAGCCTGCTAAATAGTCAGCATCGCA 1267
JN863299.1 KNUA021   GTTCTTAGTTGGTGGGTTGCCTTGTCAGGTTGATTCCGGTAACGAACGAGACCTCAGCCTGCTAAATAGTCAGCATCGCA 1337
KR904893.1 CBS152280 GTTCTTAGTTGGTGGGTTGCCTTGTCAGGTTGATTCCGGTAACGAACGAGACCTCAGCCTGCTAAATAGTCAGCATCGCA 978
KX781335.1 P4        GTTCTTAGTTGGTGGGTTGCCTTGTCAGGTTGATTCCGGTAACGAACGAGACCTCAGCCTGCTAAATAGTCAGCATCGCA 1267
JX888472.1 CC-621    GTTCTTAGTTGGTGGGTTGCCTTGTCAGGTTGATTCCGGTAACGAACGAGACCTCAGCCTGCTAAATAGTCAGCATCGCA 1337
KC310450.1 RAC       GTTCTTAGTTGGTGGGTTGCCTTGTCAGGTTGATTCCGGTAACGAACGAGACCTCAGCCTGCTAAATAGTCAGCATCGCA 89
JX888471.1 CC-620    GTTCTTAGTTGGTGGGTTGCCTTGTCAGGTTGATTCCGGTAACGAACGAGACCTCAGCCTGCTAAATAGTCAGCATCGCA 1337

KX781331.1 P8        TTTTGCAGTTGGCCGACTTCTTAGAGGGACTATTGTCGTGTAGGCAATGGAAGTATGAGGCAATAACAGGTCTGTGATGC 1399
KX781328.1 P12       -CCTGCGGTGCGCCGACTTCTTAGAGGGACTATTGGCGTTTAGCCAATGGAAGTATGAGGCGATAACAGGTCTGTGATGC 1205
KX781321.1 P20       TTTTGCAGTTGGCCGACTTCTTAGAGGGACTATTGTCGTGTAGGCAATGGAAGTATGAGGCAATAACAGGTCTGTGATGC 1347
KX781327.1 P13       -CCTGCGGTGCGCCGACTTCTTAGAGGGACTATTGGCGTTTAGCCAATGGAAGTATGAGGCGATAACAGGTCTGTGATGC 1136
KX781337.1 P2        TTTTGCAGTTGGCCGACTTCTTAGAGGGACTATTGTCGTGTAGGCAATGGAAGTATGAGGCAATAACAGGTCTGTGATGC 1067
JN903984.1 SAG53.72  TTTTGCAGTTGGCCGACTTCTTAGAGGGACTATTGTCGTGTAGGCAATGGAAGTATGAGGCAATAACAGGTCTGTGATGC 1417
KR904894.1 CC-849    -CCTGCGGTGCGCCGACTTCTTAGAGGGACTATTGGCGTTTAGCCAATGGAAGTATGAGGCGATAACAGGTCTGTGATGC 1042
KC149968.1 GTD4C     -CCTGCGGTGCGCCGACTTCTTAGAGGGACTATTGGCGTTTAGCCAATGGAAGTATGAGGCGATAACAGGTCTGTGATGC 1434
KX781333.1 P6        -CCTGCGGTGCGCCGACTTCTTAGAGGGACTATTGGCGTTTAGCCAATGGAAGTATGAGGCGATAACAGGTCTGTGATGC 1276
AY665726.1 CC-1418   -CCTGCGGTGCGCCGACTTCTTAGAGGGACTATTGGCGTTTAGCCAATGGAAGTATGAGGCGATAACAGGTCTGTGATGC 1393
AY665727.1 CC-1952   -CCTGCGGTGCGCCGACTTCTTAGAGGGACTATTGGCGTTTAGCCAATGGAAGTATGAGGCGATAACAGGTCTGTGATGC 1387
AB701550.1 NIES-2235 -CCTGCGGTGCGCCGACTTCTTAGAGGGACTATTGGCGTTTAGCCAATGGAAGTATGAGGCGATAACAGGTCTGTGATGC 1351
AB701554.1 NIES-2239 -CCTGCGGTGCGCCGACTTCTTAGAGGGACTATTGGCGTTTAGCCAATGGAAGTATGAGGCGATAACAGGTCTGTGATGC 1333
AB701553.1 NIES-2238 -CCTGCGGTGCGCCGACTTCTTAGAGGGACTATTGGCGTTTAGCCAATGGAAGTATGAGGCGATAACAGGTCTGTGATGC 1345
AB753040.1 PS-2708   -CCTGCGGTGCGCCGACTTCTTAGAGGGACTATTGGCGTTTAGCCAATGGAAGTATGAGGCGATAACAGGTCTGTGATGC 1333
AB701552.1 NIES-2237 -CCTGCGGTGCGCCGACTTCTTAGAGGGACTATTGGCGTTTAGCCAATGGAAGTATGAGGCGATAACAGGTCTGTGATGC 1344
AB701551.1 NIES-2236 -CCTGCGGTGCGCCGACTTCTTAGAGGGACTATTGGCGTTTAGCCAATGGAAGTATGAGGCGATAACAGGTCTGTGATGC 1336
AB701555.1 NIES-2463 -CCTGCGGTGCGCCGACTTCTTAGAGGGACTATTGGCGTTTAGCCAATGGAAGTATGAGGCGATAACAGGTCTGTGATGC 1347
KR092109.1 CC-125    -CCTGCGGTGCGCCGACTTCTTAGAGGGACTATTGGCGTTTAGCCAATGGAAGTATGAGGCGATAACAGGTCTGTGATGC 1373
KX781322.1 P18       -CCTGCGGTGCGCCGACTTCTTAGAGGGACTATTGGCGTTTAGCCAATGGAAGTATGAGGCGATAACAGGTCTGTGATGC 1203
KX781326.1 P14       -CCTGCGGTGCGCCGACTTCTTAGAGGGACTATTGGCGTTTAGCCAATGGAAGTATGAGGCGATAACAGGTCTGTGATGC 1344
KX781329.1 P11       -CCTGCGGTGCGCCGACTTCTTAGAGGGACTATTGGCGTTTAGCCAATGGAAGTATGAGGCGATAACAGGTCTGTGATGC 1135
KX781330.1 P10       -CCTGCGGTGCGCCGACTTCTTAGAGGGACTATTGGCGTTTAGCCAATGGAAGTATGAGGCGATAACAGGTCTGTGATGC 1233
KX781332.1 P7        -CCTGCGGTGCGCCGACTTCTTAGAGGGACTATTGGCGTTTAGCCAATGGAAGTATGAGGCGATAACAGGTCTGTGATGC 839
AB511837.1 KkS0801D2 -CCTGCGGTGCGCCGACTTCTTAGAGGGACTATTGGCGTTTAGCCAATGGAAGTATGAGGCGATAACAGGTCTGTGATGC 1387
AB511836.1 KkS0801B1 -CCTGCGGTGCGCCGACTTCTTAGAGGGACTATTGGCGTTTAGCCAATGGAAGTATGAGGCGATAACAGGTCTGTGATGC 1387
AB511835.1 SAG 11-32 -CCTGCGGTGCGCCGACTTCTTAGAGGGACTATTGGCGTTTAGCCAATGGAAGTATGAGGCGATAACAGGTCTGTGATGC 1387
AB511834.1 UTEX 90   -CCTGCGGTGCGCCGACTTCTTAGAGGGACTATTGGCGTTTAGCCAATGGAAGTATGAGGCGATAACAGGTCTGTGATGC 1387
KC166137.1 K01       -CCTGCGGTGCGCCGACTTCTTAGAGGGACTATTGGCGTTTAGCCAATGGAAGTATGAGGCGATAACAGGTCTGTGATGC 1219
KX781338.1 P1        TTTTGCAGTTGGCCGACTTCTTAGAGGGACTATTGTCGTGTAGGCAATGGAAGTATGAGGCAATAACAGGTCTGTGATGC 717
KF864473.1 JinCheon1 -CCTGCGGTGCGCAGACTTCTTAGAGGGACTATTGGCGTTCAGCCAATGGAAGTATGAGGCGATAACAGGTCTGTGATGC 1433
EU925397.1 CC-124    -CCTGCGGTGCGCCGACTTCTTAGAGGGACTATTGGCGTTTAGCCAATGGAAGTATGAGGCGATAACAGGTCTGTGATGC 1067
JN903974.1 SAG18.79  -CCTGCGGTGCGCCGACTTCTTAGAGGGACTATTGGCGTTTAGCCAATGGAAGTATGAGGCGATAACAGGTCTGTGATGC 1413
JN903978.1 SAG11-32c -CCTGCGGTGCGCCGACTTCTTAGAGGGACTATTGGCGTTTAGCCAATGGAAGTATGAGGCGATAACAGGTCTGTGATGC 1414
KX781325.1 P15       -CCTGCGGTGCGCCGACTTCTTAGAGGGACTATTGGCGTTTAGCCAATGGAAGTATGAGGCGATAACAGGTCTGTGATGC 1346
JN863299.1 KNUA021   -CCTGCGGTGCGCCGACTTCTTAGAGGGACTATTGGCGTTTAGCCAATGGAAGTATGAGGCGATAACAGGTCTGTGATGC 1416
```

```
KR904893.1 CBS152280 -CCTGCGGTGCGCCGACTTCTTAGAGGGACTATTGGCGTTTAGCCAATGGAAGTATGAGGCGATAACAGGTCTGTGATGC 1057
KX781335.1 P4        -CCTGCGGTGCGCCGACTTCTTAGAGGGACTATTGGCGTTTAGCCAATGGAAGTATGAGGCGATAACAGGTCTGTGATGC 1346
JX888472.1 CC-621    -CCTGCGGTGCGCCGACTTCTTAGAGGGACTATTGGCGTTTAGCCAATGGAAGTATGAGGCGATAACAGGTCTGTGATGC 1416
KC310450.1 RAC       -CCTGCGGTGCGCCGACTTCTTAGAGGGACTATTGGCGTTTAGCCAATGGAAGTATGAGGCGATAACAGGTCTGTGATGC 168
JX888471.1 CC-620    -CCTGCGGTGCGCCGACTTCTTAGAGGGACTATTGGCGTTTAGCCAATGGAAGTATGAGGCGATAACAGGTCTGTGATGC 1416

KX781331.1 P8        CCTTAGATGTTCTGGGCCGCACGCGCGCTACACTGACGCATTCAACGAGCCTATCCTTGGCCGAGAGGCCCGGGTAATCT 1479
KX781328.1 P12       CCTTAGATGTTCTGGGCCGCACGCGCGCTACACTGACGCGACCAACGAGCCTATCCTTGGCCGAGAGGCCCGGGTAATCT 1285
KX781321.1 P20       CCTTAGATGTTCTGGGCCGCACGCGCGCTACACTGACGCATTCAACGAGCCTATCCTTGGCCGAGAGGCCCGGGTAATCT 1427
KX781327.1 P13       CCTTAGATGTTCTGGGCCGCACGCGCGCTACACTGACGCGACCAACGAGCCTATCCTTGGCCGAGAGGCCCGGGTAATCT 1216
KX781337.1 P2        CCTTAGATGTTCTGGGCCGCACGCGCGCTACACTGACGCATTCAACGAGCCTATCCTTGGCCGAGAGGCCCGGGTAATCT 1147
JN903984.1 SAG53.72  CCTTAGATGTTCTGGGCCGCACGCGCGCTACACTGACGCATTCAACGAGCCTATCCTTGGCCGAGAGGCCCGGGTAATCT 1497
KR904894.1 CC-849    CCTTAGATGTTCTGGGCCGCACGCGCGCTACACTGACGCGACCAACGAGCCTATCCTTGGCCGAGAGGCCCGGGTAATCT 1122
KC149968.1 GTD4C     CCTTAGATGTTCTGGGCCGCACGCGCGCTACACTGACGCGATCAACGAGCCTATCCTTGGCCGAGAGGCCCGGGTAATCT 1514
KX781333.1 P6        CCTTAGATGTTCTGGGCCGCACGCGCGCTACACTGACGCGACCAACGAGCCTATCCTTGGCCGAGAGGCCCGGGTAATCT 1356
AY665726.1 CC-1418   CCTTAGATGTTCTGGGCCGCACGCGCGCTACACTGACGCGACCAACGAGCCTATCCTTGGCCGAGAGGCCCGGGTAATCT 1473
AY665727.1 CC-1952   CCTTAGATGTTCTGGGCCGCACGCGCGCTACACTGACGCGACCAACGAGCCTATCCTTGGCCGAGAGGCCCGGGTAATCT 1467
AB701550.1 NIES-2235 CCTTAGATGTTCTGGGCCGCACGCGCGCTACACTGACGCGACCAACGAGCCTATCCTTGGCCGAGAGGCCCGGGTAATCT 1431
AB701554.1 NIES-2239 CCTTAGATGTTCTGGGCCGCACGCGCGCTACACTGACGCGACCAACGAGCCTATCCTTGGCCGAGAGGCCCGGGTAATCT 1413
AB701553.1 NIES-2238 CCTTAGATGTTCTGGGCCGCACGCGCGCTACACTGACGCGACCAACGAGCCTATCCTTGGCCGAGAGGCCCGGGTAATCT 1425
AB753040.1 PS-2708   CCTTAGATGTTCTGGGCCGCACGCGCGCTACACTGACGCGACCAACGAGCCTATCCTTGGCCGAGAGGCCCGGGTAATCT 1413
AB701552.1 NIES-2237 CCTTAGATGTTCTGGGCCGCACGCGCGCTACACTGACGCGACCAACGAGCCTATCCTTGGCCGAGAGGCCCGGGTAATCT 1424
AB701551.1 NIES-2236 CCTTAGATGTTCTGGGCCGCACGCGCGCTACACTGACGCGACCAACGAGCCTATCCTTGGCCGAGAGGCCCGGGTAATCT 1416
AB701555.1 NIES-2463 CCTTAGATGTTCTGGGCCGCACGCGCGCTACACTGACGCGACCAACGAGCCTATCCTTGGCCGAGAGGCCCGGGTAATCT 1427
KR092109.1 CC-125    CCTTAGATGTTCTGGGCCGCACGCGCGCTACACTGACGCGACCAACGAGCCTATCCTTGGCCGAGAGGCCCGGGTAATCT 1453
KX781322.1 P18       CCTTAGATGTTCTGGGCCGCACGCGCGCTACACTGACGCGACCAACGAGCCTATCCTTGGCCGAGAGGCCCGGGTAATCT 1283
KX781326.1 P14       CCTTAGATGTTCTGGGCCGCACGCGCGCTACACTGACGCGACCAACGAGCCTATCCTTGGCCGAGAGGCCCGGGTAATCT 1424
KX781329.1 P11       CCTTAGATGTTCTGGGCCGCACGCGCGCTACACTGACGCGACCAACGAGCCTATCCTTGGCCGAGAGGCCCGGGTAATCT 1215
KX781330.1 P10       CCTTAGATGTTCTGGGCCGCACGCGCGCTACACTGACGCGACCAACGAGCCTATCCTTGGCCGAGAGGCCCGGGTAATCT 1313
KX781332.1 P7        CCTTAGATGTTCTGGGCCGCACGCGCGCTACACTGACGCGACCAACGAGCCTATCCTTGGCCGAGAGGCCCGGGTAATCT 919
AB511837.1 KkS0801D2 CCTTAGATGTTCTGGGCCGCACGCGCGCTACACTGACGCGACCAACGAGCCTATCCTTGGCCGAGAGGCCCGGGTAATCT 1467
AB511836.1 KkS0801B1 CCTTAGATGTTCTGGGCCGCACGCGCGCTACACTGACGCGACCAACGAGCCTATCCTTGGCCGAGAGGCCCGGGTAATCT 1467
AB511835.1 SAG 11-32 CCTTAGATGTTCTGGGCCGCACGCGCGCTACACTGACGCGACCAACGAGCCTATCCTTGGCCGAGAGGCCCGGGTAATCT 1467
AB511834.1 UTEX 90   CCTTAGATGTTCTGGGCCGCACGCGCGCTACACTGACGCGACCAACGAGCCTATCCTTGGCCGAGAGGCCCGGGTAATCT 1467
KC166137.1 K01       CCTTAGATGTTCTGGGCCGCACGCGCGCTACACTGACGCGACCAACGAGCCTATCCTTGGCCGAGAGGCCCGGGTAATCT 1299
KX781338.1 P1        CCTTAGATGTTCTGGGCCGCACGCGCGCTACACTGACGCATTCAACGAGCCTATCCTTGGCCGAGAGGCCCGGGTAATCT 797
KF864473.1 JinCheon1 CCTTAGATGTTCTGGGCCGCACGCGCGCTACACTGACGCGACCAACGAGCCTATCCTTGGCCGAGAGGCCCGGGTAATCT 1513
EU925397.1 CC-124    CCTTAGATGTTCTGGGCCGCACGCGCGCTACACTGACGCGACCAACGAGCCTATCCTTGGCCGAGAGGCCCGGGTAATCT 1147
JN903974.1 SAG18.79  CCTTAGATGTTCTGGGCCGCACGCGCGCTACACTGACGCGACCAACGAGCCTATCCTTGGCCGAGAGGCCCGGGTAATCT 1493
JN903978.1 SAG11-32c CCTTAGATGTTCTGGGCCGCACGCGCGCTACACTGACGCGACCAACGAGCCTATCCTTGGCCGAGAGGCCCGGGTAATCT 1494
KX781325.1 P15       CCTTAGATGTTCTGGGCCGCACGCGCGCTACACTGACGCGACCAACGAGCCTATCCTTGGCCGAGAGGCCCGGGTAATCT 1426
JN863299.1 KNUA021   CCTTAGATGTTCTGGGCCGCACGCGCGCTACACTGACGCGACCAACGAGCCTATCCTTGGCCGAGAGGCCCGGGTAATCT 1496
KR904893.1 CBS152280 CCTTAGATGTTCTGGGCCGCACGCGCGCTACACTGACGCGACCAACGAGCCTATCCTTGGCCGAGAGGCCCGGGTAATCT 1137
KX781335.1 P4        CCTTAGATGTTCTGGGCCGCACGCGCGCTACACTGACGCGACCAACGAGCCTATCCTTGGCCGAGAGGCCCGGGTAATCT 1426
JX888472.1 CC-621    CCTTAGATGTTCTGGGCCGCACGCGCGCTACACTGACGCGACCAACGAGCCTATCCTTGGCCGAGAGGCCCGGGTAATCT 1496
KC310450.1 RAC       CCTTAGATGTTCTGGGCCGCACGCGCGCTACACTGACGCGACCAACGAGCCTATCCTTGGCCGAGAGGCCCGGGTAATCT 248
JX888471.1 CC-620    CCTTAGATGTTCTGGGCCGCACGCGCGCTACACTGACGCGACCAACGAGCCTATCCTTGGCCGAGAGGCCCGGGTAATCT 1496

KX781331.1 P8        TTGAAACTGCGTCGTGATGGGGATAGATTATTGCAATTATTAGTCTTCAACGAGGAATGCCTAGTAAGCGCGAGTCATCA 1559
KX781328.1 P12       TGTAAACCGCGTCGTGATGGGGATAGATTATTGCAATTATTAGTCTTCAACGAGGAATGCCTAGTAAGCGCGAGTCATCA 1365
KX781321.1 P20       TTGAAACTGCGTCGTGATGGGGATAGATTATTGCAATTATTAGTCTTCAACGAGGAATGCCTAGTAAGCGCGAGTCATCA 1507
KX781327.1 P13       TGTAAACCGCGTCGTGATGGGGATAGATTATTGCAATTATTAGTCTTCAACGAGGAATGCCTAGTAAGCGCGAGTCATCA 1296
KX781337.1 P2        TTGAAACTGCGTCGTGATGGGGATAGATTATTGCAATTATTAGTCTTCAACGAGGAATGCCTAGTAAGCGCGAGTCATCA 1227
JN903984.1 SAG53.72  TTGAAACTGCGTCGTGATGGGGATAGATTATTGCAATTATTAGTCTTCAACGAGGAATGCCTAGTAAGCGCGAGTCATCA 1577
KR904894.1 CC-849    TGTAAACCGCGTCGTGATGGGGATAGATTATTGCAATTATTAGTCTTCAACGAGGAATGCCTAGTAAGCGCGAGTCATCA 1202
KC149968.1 GTD4C     TGTAAACCGCGTCGTGATGGGGATAGACTATTGCAATTATTAGTCTTCAACGAGGAATGCCTAGTAAGCGCGAGTCATCA 1594
KX781333.1 P6        TGTAAACCGCGTCGTGATGGGGATAGATTATTGCAATTATTAGTCTTCAACGAGGAATGCCTAGTAAGCGCGAGTCATCA 1436
AY665726.1 CC-1418   TGTAAACCGCGTCGTGATGGGGATAGATTATTGCAATTATTAGTCTTCAACGAGGAATGCCTAGTAAGCGCGAGTCATCA 1553
AY665727.1 CC-1952   TGTAAACCGCGTCGTGATGGGGATAGATTATTGCAATTATTAGTCTTCAACGAGGAATGCCTAGTAAGCGCGAGTCATCA 1547
AB701550.1 NIES-2235 TGTAAACCGCGTCGTGATGGGGATAGATTATTGCAATTATTAGTCTTCAACGAGGAATGCCTAGTAAGCGCGAGTCATCA 1511
AB701554.1 NIES-2239 TGTAAACCGCGTCGTGATGGGGATAGATTATTGCAATTATTAGTCTTCAACGAGGAATGCCTAGTAAGCGCGAGTCATCA 1493
AB701553.1 NIES-2238 TGTAAACCGCGTCGTGATGGGGATAGATTATTGCAATTATTAGTCTTCAACGAGGAATGCCTAGTAAGCGCGAGTCATCA 1505
AB753040.1 PS-2708   TGTAAACCGCGTCGTGATGGGGATAGATTATTGCAATTATTAGTCTTCAACGAGGAATGCCTAGTAAGCGCGAGTCATCA 1493
AB701552.1 NIES-2237 TGTAAACCGCGTCGTGATGGGGATAGATTATTGCAATTATTAGTCTTCAACGAGGAATGCCTAGTAAGCGCGAGTCATCA 1504
AB701551.1 NIES-2236 TGTAAACCGCGTCGTGATGGGGATAGATTATTGCAATTATTAGTCTTCAACGAGGAATGCCTAGTAAGCGCGAGTCATCA 1496
AB701555.1 NIES-2463 TGTAAACCGCGTCGTGATGGGGATAGATTATTGCAATTATTAGTCTTCAACGAGGAATGCCTAGTAAGCGCGAGTCATCA 1507
KR092109.1 CC-125    TGTAAACCGCGTCGTGATGGGGATAGATTATTGCAATTATTAGTCTTCAACGAGGAATGCCTAGTAAGCGCGAGTCATCA 1533
KX781322.1 P18       TGTAAACCGCGTCGTGATGGGGATAGATTATTGCAATTATTAGTCTTCAACGAGGAATGCCTAGTAAGCGCGAGTCATCA 1363
KX781326.1 P14       TGTAAACCGCGTCGTGATGGGGATAGATTATTGCAATTATTAGTCTTCAACGAGGAATGCCTAGTAAGCGCGAGTCATCA 1504
KX781329.1 P11       TGTAAACCGCGTCGTGATGGGGATAGATTATTGCAATTATTAGTCTTCAACGAGGAATGCCTAGTAAGCGCGAGTCATCA 1295
KX781330.1 P10       TGTAAACCGCGTCGTGATGGGGATAGATTATTGCAATTATTAGTCTTCAACGAGGAATGCCTAGTAAGCGCGAGTCATCA 1393
KX781332.1 P7        TGTAAACCGCGTCGTGATGGGGATAGATTATTGCAATTATTAGTCTTCAACGAGGAATGCCTAGTAAGCGCGAGTCATCA 999
AB511837.1 KkS0801D2 TGTAAACCGCGTCGTGATGGGGATAGATTATTGCAATTATTAGTCTTCAACGAGGAATGCCTAGTAAGCGCGAGTCATCA 1547
AB511836.1 KkS0801B1 TGTAAACCGCGTCGTGATGGGGATAGATTATTGCAATTATTAGTCTTCAACGAGGAATGCCTAGTAAGCGCGAGTCATCA 1547
AB511835.1 SAG 11-32 TGTAAACCGCGTCGTGATGGGGATAGATTATTGCAATTATTAGTCTTCAACGAGGAATGCCTAGTAAGCGCGAGTCATCA 1547
AB511834.1 UTEX 90   TGTAAACCGCGTCGTGATGGGGATAGATTATTGCAATTATTAGTCTTCAACGAGGAATGCCTAGTAAGCGCGAGTCATCA 1547
KC166137.1 K01       TGTAAACCGCGTCGTGATGGGGATAGATTATTGCAATTATTAGTCTTCAACGAGGAATGCCTAGTAAGCGCGAGTCATCA 1379
KX781338.1 P1        TTGAAACTGCGTCGTGATGGGGATAGATTATTGCAATTATTAGTCTTCAACGAGGAATGCCTAGTAAGCGCGAGTCATCA 877
KF864473.1 JinCheon1 TGTAAACCGCGTCGTGATGGGGATAGATTATTGCAATTATTAGTCTTCAACGAGGAATGCCTAGTAAGCGCGAGTCATCA 1593
EU925397.1 CC-124    TGTAAACCGCGTCGTGATGGGGATAGATTATTGCAATTATTAGTCTTCAACGAGGAATGCCTAGTAAGCGCGAGTCATCA 1227
JN903974.1 SAG18.79  TGTAAACCGCGTCGTGATGGGGATAGATTATTGCAATTATTAGTCTTCAACGAGGAATGCCTAGTAAGCGCGAGTCATCA 1573
JN903978.1 SAG11-32c TGTAAACCGCGTCGTGATGGGGATAGATTATTGCAATTATTAGTCTTCAACGAGGAATGCCTAGTAAGCGCGAGTCATCA 1574
KX781325.1 P15       TGTAAACCGCGTCGTGATGGGGATAGATTATTGCAATTATTAGTCTTCAACGAGGAATGCCTAGTAAGCGCGAGTCATCA 1506
JN863299.1 KNUA021   TGTAAACCGCGTCGTGATGGGGATAGATTATTGCAATTATTAGTCTTCAACGAGGAATGCCTAGTAAGCGCGAGTCATCA 1576
KR904893.1 CBS152280 TGTAAACCGCGTCGTGATGGGGATAGATTATTGCAATTATTAGTCTTCAACGAGGAATGCCTAGTAAGCGCGAGTCATCA 1217
KX781335.1 P4        TGTAAACCGCGTCGTGATGGGGATAGATTATTGCAATTATTAGTCTTCAACGAGGAATGCCTAGTAAGCGCGAGTCATCA 1506
JX888472.1 CC-621    TGTAAACCGCGTCGTGATGGGGATAGATTATTGCAATTATTAGTCTTCAACGAGGAATGCCTAGTAAGCGCGAGTCATCA 1576
KC310450.1 RAC       TGTAAACCGCGTCGTGATGGGGATAGATTATTGCAATTATTAGTCTTCAACGAGGAATGCCTAGTAAGCGCGAGTCATCA 328
JX888471.1 CC-620    TGTAAACCGCGTCGTGATGGGGATAGATTATTGCAATTATTAGTCTTCAACGAGGAATGCCTAGTAAGCGCGAGTCATCA 1576

KX781331.1 P8        GCTCGCGTTGATTACGTCCCTGCCCTTTGTA------------------------------------------------- 1590
KX781328.1 P12       GCTCGCG------------------------------------------------------------------------- 1372
KX781321.1 P20       GCTCGCGTTGATTACGTCCCTGCCCTTTGTACACACCGCCCGTCGCTCCTACCGATTGGGTGTGCTGGTGAAGTGTTCGG 1587
KX781327.1 P13       GCTCGCGTTGACTACGTCCCTGCCCTTTGTACACACCGCCCGTCGCTCCTACCGATTGGGTGTGCTGGTGAAGTGTTCGG 1376
```

```
KX781337.1 P2        GCTCGCGTTGATTACGTCCCTGCCCTTTGTACACACCGCCCGTCGCTCCTACCGATTGGGTGTGCTGGTGAAGTGTTCGG 1307
JN903984.1 SAG53.72  GCTCGCGTTGATTACGTCCCTGCCCTTTGTACACACCGCCCGTCGCTCCTACCGATTGGGTGTGCTGGTGAAGTGTTCGG 1657
KR904894.1 CC-849    NCTCGCGTTGATTACNTCCCTGCCCTTTGTACACACCGCCCGTCGCTCCTACCGATTGGGTGTGCTGGTGAAGTGTTCGG 1282
KC149968.1 GTD4C     GCTCGCGTTGATTACGTCCCTGCCCTTTGTACACACCGCCCGTCGCTCCTACCGATTGAATGTGCTGGTGAAGTGTTCGG 1674
KX781333.1 P6        GCTCGCGTTGACTACGTCCCTGCCCTTTGTACACACCGCCCGTCGCTCCTACCGATTGGGTGTGCTGGTGAAGTGTTCGG 1516
AY665726.1 CC-1418   GCTCGCGTTGATTACGTCCCTGCCCTTTGTACACACCGCCCGTCGCTCCTACCGATTGGGTGTGCTGGTGAAGTGTTCGG 1633
AY665727.1 CC-1952   GCTCGCGTTGATTACGTCCCTGCCCTTTGTACACACCGCCCGTCGCTCCTACCGATTGGGTGTGCTGGTGAAGTGTTCGG 1627
AB701550.1 NIES-2235 GCTCGCGTTGATTACGTCCCTGCCCTTTGTACACACCGCCCGTCGCTCCTACCGATTGGGTGTGCTGGTGAAGTGTTCGG 1591
AB701554.1 NIES-2239 GCTCGCGTTGATTACGTCCCTGCCCTTTGTACACACCGCCCGTCGCTCCTACCGATTGGGTGTGCTGGTGAAGTGTTCGG 1573
AB701553.1 NIES-2238 GCTCGCGTTGATTACGTCCCTGCCCTTTGTACACACCGCCCGTCGCTCCTACCGATTGGGTGTGCTGGTGAAGTGTTCGG 1585
AB753040.1 PS-2708   GCTCGCGTTGATTACGTCCCTGCCCTTTGTACACACCGCCCGTCGCTCCTACCGATTGGGTGTGCTGGTGAAGTGTTCGG 1573
AB701552.1 NIES-2237 GCTCGCGTTGATTACGTCCCTGCCCTTTGTACACACCGCCCGTCGCTCCTACCGATTGGGTGTGCTGGTGAAGTGTTCGG 1584
AB701551.1 NIES-2236 GCTCGCGTTGATTACGTCCCTGCCCTTTGTACACACCGCCCGTCGCTCCTACCGATTGGGTGTGCTGGTGAAGTGTTCGG 1576
AB701555.1 NIES-2463 GCTCGCGTTGATTACGTCCCTGCCCTTTGTACACACCGCCCGTCGCTCCTACCGATTGGGTGTGCTGGTGAAGTGTTCGG 1587
KR092109.1 CC-125    GCTCGCGTTGATTACGTCCCTGCCCTTTGTACACACCGCCCGTCGCTCCTACCGATTGGGTGTGCTGGTGAAGTGTTCGG 1613
KX781322.1 P18       GCTCGCGTTGATTACGTCCCTGCCCTTTGTACACACCGCCCGTCGCTCCTACCGATTGGGTGTGCTGGTGAAGTGTTCGG 1443
KX781326.1 P14       GCTCGCGTTGATTACGTCCCTGCCCTTTGTACACACCGCCCGTCGCTCCTACCGATTGGGTGTGCTGGTGAAGTGTTCGG 1584
KX781329.1 P11       GCTCGCGTTGATTACGTCCCTGCCCTTTGTACACACCGCCCGTCGCTCCTACCGATTGGGTGTGCTGGTGAAGTGTTCGG 1375
KX781330.1 P10       GCTCGCGTTGATTACGTCCCTGCCCTTTGTACACACCGCCCGTCGCTCCTACCGATTGGGTGTGCTGGTGAAGTGTTCGG 1473
KX781332.1 P7        GCTCGCGTTGATTACGTCCCTGCCCTTTGTACACACCGCCCGTCGCTCCTACCGATTGGGTGTGCTGGTGAAGTGTTCGG 1079
AB511837.1 KkS0801D2 GCTCGCGTTGATTACGTCCCTGCCCTTTGTACACACCGCCCGTCGCTCCTACCGATTGGGTGTGCTGGTGAAGTGTTCGG 1627
AB511836.1 KkS0801B1 GCTCGCGTTGATTACGTCCCTGCCCTTTGTACACACCGCCCGTCGCTCCTACCGATTGGGTGTGCTGGTGAAGTGTTCGG 1627
AB511835.1 SAG 11-32 GCTCGCGTTGATTACGTCCCTGCCCTTTGTACACACCGCCCGTCGCTCCTACCGATTGGGTGTGCTGGTGAAGTGTTCGG 1627
AB511834.1 UTEX 90   GCTCGCGTTGATTACGTCCCTGCCCTTTGTACACACCGCCCGTCGCTCCTACCGATTGGGTGTGCTGGTGAAGTGTTCGG 1627
KC166137.1 K01       GCTCGCGTTGATTACGTCCCTGCCCTTTGTACACACCGCCCGTCGCTCCTACCGATTGGGTGTGCTGGTGAAGTGTTCGG 1459
KX781338.1 P1        GCTCGCGTTGATTACGTCCCTGCCCTTTGTACACACCGCCCGTCGCTCCTACCG-------------------------- 931
KF864473.1 JinCheon1 GCTCGCATTGATTACGTCCCTGCCCTTTGTACACACCGCCCGTCGCTCCTACCGATTGGGTGTGCTGGTGAAGTGTTCGG 1673
EU925397.1 CC-124    GCTCGCGTTGATTACGTCCCTGCCCTTTGTACACACCGCCCGTCGCTCCTACCGATTGGGTGTGCTGGTGAAGTGTTCGG 1307
JN903974.1 SAG18.79  GCTCGCGTTGATTACGTCCCTGCCCTTTGTACACACCGCCCGTCGCTCCTACCGATTGGGTGTGCTGGTGAAGTGTTCGG 1653
JN903978.1 SAG11-32c GCTCGCGTTGATTACGTCCCTGCCCTTTGTACACACCGCCCGTCGCTCCTACCGATTGGGTGTGCTGGTGAAGTGTTCGG 1654
KX781325.1 P15       GCTCGCGTTGATTACGTCCCTGCCCTTTGTACACACCGCCCGTCGCTCCTACCGATTGGGTGTGCTGGTGAAGTGTTCGG 1586
JN863299.1 KNUA021   GCTCGCGTTGACTACGTCCCTGCCCTTTGTACACACCGCCCGTCGCTCCTACCGATTGGGTGTGCTGGTGAAGTGTTCGG 1656
KR904893.1 CBS152280 GCTCGCGTTGATTACGTCCCTGCCCTTTGTACACACCGCCCGTCGCTCCTACCGATTGGGTGTGCTGGTGAAGTGTTCGG 1297
KX781335.1 P4        GCTCGCGTTGATTACGTCCCTGCCCTTTGTACACACCGCCCGTCGCTCCTACCGATTGGGTGTGCTGGTGAAGTGTTCGG 1586
JX888472.1 CC-621    GCTCGCGTTGATTACGTCCCTGCCCTTTGTACACACCGCCCGTCGCTCCTACCGATTGGGTGTGCTGGTGAAGTGTTCGG 1656
KC310450.1 RAC       GCTCGCGTTGATTACGTCCCTGCCCTTTGTACACACCGCCCGTCGCTCCTACCGATTGGGTGTGCTGGTGAAGTGTTCGG 408
JX888471.1 CC-620    GCTCGCGTTGATTACGTCCCTGCCCTTTGTACACACCGCCCGTCGCTCCTACCGATTGGGTGTGCTGGTGAAGTGTTCGG 1656

KX781331.1 P8        -------------------------------------------------------------------------------- 1590
KX781328.1 P12       -------------------------------------------------------------------------------- 1372
KX781321.1 P20       ATTGGCTTCAGGTGATGGCAACA--------------------------------------------------------- 1610
KX781327.1 P13       ATTGAGCTTGGCTGG-GGCAACCTGGCC-TTGCTTGAGAAGTTCATTAAACCCT-------------------------- 1428
KX781337.1 P2        ATTGGCTTCAGGTGATGGCAACA--------------------------------------------------------- 1330
JN903984.1 SAG53.72  ATTGGCTTCAGGTGATGGCAACATCGCCTGTTGCTGAGAAGTTCATTAAACCCTCCCACCTAGAGGAAGGAGAAGTCGTA 1737
KR904894.1 CC-849    ATTG---------------------------------------------------------------------------- 1286
KC149968.1 GTD4C     ATTGGCCTTGATT-GGGGCAACTCGGTC-TTGGCTGAAAAGTTCATTAAACCCTCCCATTTAGAGGAAGGAGAAGTCGTA 1752
KX781333.1 P6        ATTGAGCTTGGCT-GGGGCAACCTGGCC-TTGCTTGAGAAGTTCATTAAACCCT-------------------------- 1568
AY665726.1 CC-1418   ATTGAGCTTGGCT-GGGGCAACCTGGCC-TTGCTTGAGAAGTTCATTAAACCCTCCCACCTA------------------ 1693
AY665727.1 CC-1952   ATTGAGCTTGGCT-GGGGCAACCTGGCC-TTGCTTGAGAAGTTCATTAAACCCTCCCACCTA------------------ 1687
AB701550.1 NIES-2235 ATTGAGCTTGGCT-GGGGCAACCTGGCC-TTGCTTGAGAAGT-CATTAAACCCTCCCACCTAGAGGAA------------ 1656
AB701554.1 NIES-2239 ATTGAGCTTGGCT-GGGGCAACCTGGCC-TTGCTTGAGAAGT-------------------------------------- 1613
AB701553.1 NIES-2238 ATTGAGCTTGGCT-GGGGCAACCTGGCC-TTGCTTGAGAAGTTCATTAAACCCTCCCACCTAGAGGAA------------ 1651
AB753040.1 PS-2708   ATTGAGCTTGGCT-GGGGCAACCTGGCC-TTGCTTGAGAAGTTCATTAAACCCTCCCACTA------------------- 1632
AB701552.1 NIES-2237 ATTGAGCTTGGCT-GGGGCAACCTGGCC-TTGCTTGAGAAGTTCATTAAACCCTCCCACCTA------------------ 1644
AB701551.1 NIES-2236 ATTGAGCTTGGCT-GGGGCAACCTGGCC-TTGCTTGAGAAGTTCATTAAACCCTCCCACCTA------------------ 1636
AB701555.1 NIES-2463 ATTGAGCTTGGCT-GGGGCAACCTGGCC-TTGCTTGAGAAGTTCATTAAACCCTCC------------------------ 1641
KR092109.1 CC-125    ATTGAGCTTGGCT-GGGGCAACCTGGCC-TTGCTTGAGAAGTTCATTAAACCCTCCCACCTAGAG--------------- 1676
KX781322.1 P18       ATTGAGCTTGGCT-GGGGCAACCTGGCC-TTGCTTGAGAAGTTCATTAAACCCTCCCACCTA------------------ 1503
KX781326.1 P14       ATTGAGCTTGGCT-GGGGCAACCTGGCC-TTGCTTGAGAAGTTCATTAAACCCTCCCACCTA------------------ 1644
KX781329.1 P11       ATTGAGCTTGGCT-GGGGCAACCTGGCC-TTGCTTGAGAAGTTCATTAAACCCTCCCACCTAGAGGAA------------ 1441
KX781330.1 P10       ATTGAGCTTGGCT-GGGGCAACCTGGCC-TTGCTTGAGAAGTTCATTAAACCCTCCCACCTAGAG--------------- 1536
KX781332.1 P7        ATTGAGCTTGGCT-GGGGCAACCTGGCC-TTGCTTGAGAAGTTCATTAAACCCTCCCACCTA------------------ 1139
AB511837.1 KkS0801D2 ATTGAGCTTGGCT-GGGGCAACCTGGCC-TTGCTTGAGAAGTTCATTAAACCCTCCCACCTA------------------ 1687
AB511836.1 KkS0801B1 ATTGAGCTTGGCT-GGGGCAACCTGGCC-TTGCTTGAGAAGTTCATTAAACCCTCCCACCTA------------------ 1687
AB511835.1 SAG 11-32 ATTGAGCTTGGCT-GGGGCAACCTGGCC-TTGCTTGAGAAGTTCATTAAACCCTCCCACCTA------------------ 1687
AB511834.1 UTEX 90   ATTGAGCTTGGCT-GGGGCAACCTGGCC-TTGCTTGAGAAGTTCATTAAACCCTCCCACCTA------------------ 1687
KC166137.1 K01       AT-GAGCTTGGCT-G-GGCAACCT-------------------------------------------------------- 1480
KX781338.1 P1        -------------------------------------------------------------------------------- 931
KF864473.1 JinCheon1 ATTGAGTCTGAGT-GGGGCAACCTGGTC-AGATTCGAGAAGTTCATTAAACCCTCCCACCTAGAGGAAGGAGAAGTCGTA 1751
EU925397.1 CC-124    ATTGAGCTTGGCT-GGGGCAACCTGGCC-TTGCTTGAGAAGTTCATTAAACCCTCCCACCTAGAGGAAGGAGAAGTCGTA 1385
JN903974.1 SAG18.79  ATTGAGCTTGGCT-GGGGCAACCTGGCC-TTGCTTGAGAAGTTCATTAAACCCTCCCACCTAGAGGAAGGAGAAGTCGTA 1731
JN903978.1 SAG11-32c ATTGAGCTTGGCT-GGGGCAACCTGGCC-TTGCTTGAGAAGTTCATTAAACCCTCCCACCTAGAGGAAGGAGAAGTCGTA 1732
KX781325.1 P15       ATTGAGCTTGGCT-GGGGCAACCTGGCC-TTGCTTGAGAAGTTCATTAAACCCTCCCACCTAGAGGAAGGAGAAGTCGTA 1664
JN863299.1 KNUA021   ATTGAGCTTGGCT-GGGGCAACCTGGCC-TTGCTTGAGAAGTTCATTAAACCCTCCCACCTAGAGGAAGGAGAAGTCGTA 1734
KR904893.1 CBS152280 ATTGAGCTTGGCT-GGGGCAACCTGGCC-TTGCTTGAGAAGTTCATTAAACCCTCCCACCTAGAGGAAGGAGAAGTCGTA 1375
KX781335.1 P4        ATTGAGCTTGGCT-GGGGCAACCTGGCC-TTGCTTGAGAAGTTCATTAAACCCTCCCACCTAGAGGAAGGAGAAGTCGTA 1664
JX888472.1 CC-621    ATTGAGCTTGGCT-GGGGCAACCTGGCC-TTGCTTGAGAAGTTCATTAAACCCTCCCACCTAGAGGAAGGAGAAGTCGTA 1734
KC310450.1 RAC       ATTGAGCTTGGCT-GGGGCAACTTGGCC-TTGCTTGAGAAGTTCATTAAACCCTCCCACCTAGAGGAAGGAGAAGTCGTA 486
JX888471.1 CC-620    ATTGAGCTTGGCT-GGGGCAACCTGGCC-TTGCTTGAGAAGTTCATTAAACCCTCCCACCTAGAGGAAGGAGAAGTCGTA 1734

KX781331.1 P8        ------------------------------------- 1590
KX781328.1 P12       ------------------------------------- 1372
KX781321.1 P20       ------------------------------------- 1610
KX781327.1 P13       ------------------------------------- 1428
KX781337.1 P2        ------------------------------------- 1330
JN903984.1 SAG53.72  ACAAGGTTTC--------------------------- 1747
KR904894.1 CC-849    ------------------------------------- 1286
KC149968.1 GTD4C     ACAAGGT------------------------------ 1759
KX781333.1 P6        ------------------------------------- 1568
AY665726.1 CC-1418   ------------------------------------- 1693
AY665727.1 CC-1952   ------------------------------------- 1687
AB701550.1 NIES-2235 ------------------------------------- 1656
AB701554.1 NIES-2239 ------------------------------------- 1613
AB701553.1 NIES-2238 ------------------------------------- 1651
```

```
AB753040.1 PS-2708   ------------------------------------- 1632
AB701552.1 NIES-2237 ------------------------------------- 1644
AB701551.1 NIES-2236 ------------------------------------- 1636
AB701555.1 NIES-2463 ------------------------------------- 1641
KR092109.1 CC-125    ------------------------------------- 1676
KX781322.1 P18       ------------------------------------- 1503
KX781326.1 P14       ------------------------------------- 1644
KX781329.1 P11       ------------------------------------- 1441
KX781330.1 P10       ------------------------------------- 1536
KX781332.1 P7        ------------------------------------- 1139
AB511837.1 KkS0801D2 ------------------------------------- 1687
AB511836.1 KkS0801B1 ------------------------------------- 1687
AB511835.1 SAG 11-32 ------------------------------------- 1687
AB511834.1 UTEX 90   ------------------------------------- 1687
KC166137.1 K01       ------------------------------------- 1480
KX781338.1 P1        ------------------------------------- 931
KF864473.1 JinCheon1 ACAAGGTTTCCGTAGGTGAACCTGCAGAAGGATCA-- 1786
EU925397.1 CC-124    ACAAGGTAAGGG----CGAATC--------------- 1403
JN903974.1 SAG18.79  ACAAGGTTTC--------------------------- 1741
JN903978.1 SAG11-32c ACAAGGTTTC--------------------------- 1742
KX781325.1 P15       ACAAGGTTTCCGTAG---------------------- 1679
JN863299.1 KNUA021   ACAAGGTTTCCGTAGGTGAACCTGCGGA--------- 1762
KR904893.1 CBS152280 ACAAGGTTTCCGTAGGTGAACCTGCGGAAGGATCATT 1412
KX781335.1 P4        ACAAGGTTTCCGTAGGTGAACCTGCGGA--------- 1692
JX888472.1 CC-621    ACAAGGTTTCCGTAGGTGAACCTGCGGA--------- 1762
KC310450.1 RAC       ACAAGGTTTCCGTAGGTGAA----------------- 506
JX888471.1 CC-620    ACAAGGTTTCCGTAGGTGAACCTGCGGA--------- 1762
```
