# Supplementary material for: Overexpression of 18S rRNA methyltransferase CrBUD23 enhances biomass and lutein content in Chlamydomonas reinhardtii
Source: Front Bioeng Biotechnol. 2023 Feb 3;11:1102098. doi: 10.3389/fbioe.2023.1102098 (PMC9935685; doi:10.3389/fbioe.2023.1102098)
Supplement: Supplementary file 7 [file Table2.DOCX]

XR_007090847.1

>Hs18SrRNA

TACCTGGTTGATCCTGCCAGTAGCATATGCTTGTCTCAAAGATTAAGCCATGCATGTCTAAGTACGCACGGCCGGTACAGTGAAACTGCGAATGGCTCATTAAATCAGTTATGGTTCCTTTGGTCGCTCGCTCCTCTCCTACTTGGATAACTGTGGTAATTCTAGAGCTAATACATGCCGACGGGCGCTGACCCCCTTCGCGGGGGGGATGCGTGCATTTATCAGATCAAAACCAACCCGGTCAGCCCCTCTCCGGCCCCGGCCGGGGGGCGGGCGCCGGCGGCTTTGGTGACTCTAGATAACCTCGGGCCGATCGCACGCCCCCCGTGGCGGCGACGACCCATTCGAACGTCTGCCCTATCAACTTTCGATGGTAGTCGCCGTGCCTACCATGGTGACCACGGGTGACGGGGAATCAGGGTTCGATTCCGGAGAGGGAGCCTGAGAAACGGCTACCACATCCAAGGAAGGCAGCAGGCGCGCAAATTACCCACTCCCGACCCGGGGAGGTAGTGACGAAAAATAACAATACAGGACTCTTTCGAGGCCCTGTAATTGGAATGAGTCCACTTTAAATCCTTTAACGAGGATCCATTGGAGGGCAAGTCTGGTGCCAGCAGCCGCGGTAATTCCAGCTCCAATAGCGTATATTAAAGTTGCTGCAGTTAAAAAGCTCGTAGTTGGATCTTGGGAGCGGGCGGGCGGTCCGCCGCGAGGCGAGCCACCGCCCGTCCCCGCCCCTTGCCTCTCGGCGCCCCCTCGATGCTCTTAGCTGAGTGTCCCGCGGGGCCCGAAGCGTTTACTTTGAAAAAATTAGAGTGTTCAAAGCAGGCCCGAGCCGCCTGGATACCGCAGCTAGGAATAATGGAATAGGACCGCGGTTCTATTTTGTTGGTTTTCGGAACTGAGGCCATGATTAAGAGGGACGGCCGGGGGCATTCGTATTGCGCCGCTAGAGGTGAAATTCTTGGACCGGCGCAAGACGGACCAGAGCGAAAGCATTTGCCAAGAATGTTTTCATTAATCAAGAACGAAAGTCGGAGGTTCGAAGACGATCAGATACCGTCGTAGTTCCGACCATAAACGATGCCGACCGGCGATGCGGCGGCGTTATTCCCATGACCCGCCGGGCAGCTTCCGGGAAACCAAAGTCTTTGGGTTCCGGGGGGAGTATGGTTGCAAAGCTGAAACTTAAAGGAATTGACGGAAGGGCACCACCAGGAGTGGAGCCTGCGGCTTAATTTGACTCAACACGGGAAACCTCACCCGGCCCGGACACGGACAGGATTGACAGATTGATAGCTCTTTCTCGATTCCGTGGGTGGTGGTGCATGGCCGTTCTTAGTTGGTGGAGCGATTTGTCTGGTTAATTCCGATAACGAACGAGACTCTGGCATGCTAACTAGTTACGCGACCCCCGAGCGGTCGGCGTCCCCCAACTTCTTAGAGGGACAAGTGGCGTTCAGCCACCCGAGATTGAGCAATAACAGGTCTGTGATGCCCTTAGATGTCCGGGGCTGCACGCGCGCTACACTGACTGGCTCAGCGTGTGCCTACCCTACGCCGGCAGGCGCGGGTAACCCGTTGAACCCCATTCGTGATGGGGATCGGGGATTGCAATTATTCCCCATGAACGAGGAATTCCCAGTAAGTGCGGGTCATAAGCTTGCGTTGATTAAGTCCCTGCCCTTTGTACACACCGCCCGTCGCTACTACCGATTGGATGGTTTAGTGAGGCCCTCGGATCGGCCCCGCCGGGGTCGGCCCACGGCCCTGGCGGAGCGCTGAGAAGACGGTCGAACTTGACTATCTAGAGGAAGTAAAAGTCGTAACAAGGTTTCCGTAGGTGAACCTGCGGAAGGATCATTA

KR904894.1

CC-849

>Cr18SrRNA

TGAGAGATGGCTACCACATCCAAGGAAGGCAGCAGGCGCGCAAATTACCCAATCCCGACACGGGGAGGTAGTGACAATAAATAACAATACCGGGCGCTTCGCGTCTGGTAATTGGAATGAGTACAATCTAAATCCCTTAACGAGGATCCATTGGAGGGCAAGTCTGGTGCCAGCAGCCGCGGTAATTCCAGCTCCAATAGCGTATATTTAAGTTGTTGCAGTTAAAAAGCTCGTAGTTGGATTTCGGGTGGGGTGGTGCGGTCCGCCTCTGGTGTGCACTGCTCTGCTCCACCTTCCTGCCGGGGACGGGCTCCTGGGCTTCACTGTCTGGGACTCGGAGTCGGCGAGGTTACTTTGAGTAAATTAGAGTGTTCAAAGCAGGCCTACGCTCTGAATACATTAGCATGGAATAACACGATAGGACTCTGGCCTATCTGTTGGTCTGTGGGACCGGAGTAATGATTAAGAGGGGTAGTCGGGGGCATTCGTATTCCGTTGTCAGAGGTGAAATTCTTGGATTTACGGAAGACGAACATCTGCGAAAGCATTTGCCAAGGATACTTTCATTGATCAAGAACGAAAGTTGGGGGCTCGAAGACGATTAGATACCGTCGTAGTCTCAACCATAAACGATGCCGACTAGGGATTGGCAGATGTTCTTTTGATGACTCTGCCAGCACCTTATGAGAAATCAAAGTTTTTGGGTTCCGGGGGGAGTATGGTCGCAAGGCTGAAACTTAAAGGAATTGACGGAAGGGCACCACCAGGCGTGGAGCCTGCGGCTTAATTTGACTCAACACGGGGAAACTTACCAGGTCCAGACACGGGAAGGATTGACAGATTGAGAGCTCTTTCTTGATTCTGTGGGTGGTGGTGCATGGCCGTTCTTAGTTGGTGGGTTGCCTTGTCAGGTTGATTCCGGTAACAAACGAGACCTCAGCCTGCTAAATAGTCAGCATCGCACCTGCGGTGCGCCGACTTCTTAGAGGGACTATTGGCGTTTAGCCAATGGAAGTATGAGGCGATAACAGGTCTGTGATGCCCTTAGATGTTCTGGGCCGCACGCGCGCTACACTGACGCGACCAACGAGCCTATCCTTGGCCGAGAGGCCCGGGTAATCTTGTAAACCGCGTCGTGATGGGGATAGATTATTGCAATTATTAGTCTTCAACGAGGAATGCCTAGTAAGCGCGAGTCATCANCTCGCGTTGATTACNTCCCTGCCCTTTGTACACACCGCCCGTCGCTCCTACCGATTGGGTGTGCTGGTGAAGTGTTCGGATTG

X16077.1

>At18SrRNA

AAAAGATGACGGTCAAGACCTCGTCCTTTCTCTCTTTCCATTGCGTTTGAGAGGATGTGGCGGGGAATTGCCGTGATCGATGAATGCTACCTGGTTGATCCTGCCAGTAGTCATATGCTTGTCTCAAAGATTAAGCCATGCATGTGTAAGTATGAACGAATTCAGACTGTGAAACTGCGAATGGCTCATTAAATCAGTTATAGTTTGTTTGATGGTAACTACTACTCGGATAACCGTAGTAATTCTAGAGCTAATACGTGCAACAAACCCCGACTTATGGAAGGGACGCATTTATTAGATAAAAGGTCGACGCGGGCTCTGGCTTGCTCTGATGATTCATGATAACTCGACGGATCGCATGGCCTCTGTGCTGGCGACGCATCATTCAAATTTCTGCCCTATCAACTTTCGATGGTAGGATAGTGGCCTACCATGGTGGTAACGGGTGACGGAGAATTAGGGTTCGATTCCGGAGAGGGAGCCTGAGAAACGGCTACCACATCCAAGGAAGGCAGCAGGCGCGCAAATTACCCAATCCTGACACGGGGAGGTAGTGACAATAAATAACAATACCGGGCTCTTTCGAGTCTGGTAATTGGAATGAGTACAATCTAAATCCCTTAACGAGGATCCATTGGAGGGCAAGTCTGGTGCCAGCAGCCGCGGTAATTCCAGCTCCAATAGCGTATATTTAAGTTGTTGCAGTTAAAAAGCTCGTAGTTGAACCTTGGGATGGGTCGGCCGGTCCGCCTTTGGTGTGCATTGGTCGGCTTGTCCCTTCGGTCGGCGATACGCTCCTGGTCTTAATTGGCCGGGTCGTGCCTCCGGCGCTGTTACTTTGAAGAAATTAGAGTGCTCAAAGCAAGCCTACGCTCTGGATACATTAGCATGGGATAACATCATAGGATTTCGATCCTATTGTGTTGGCTTCGGGATCGGAGTAATGATTAACAGGGACAGTCGGGGGCATTCGTATTTCATAGTCAGAGGTGAAATTCTTGGATTTATGAAAGACGAACAACTGCGAAAGCATTTGCCAAGGATGTTTTCATTAATCAAGAACGAAAGTTGGGGGCTCGAAGACGATCAGATACCGTCCTAGTCTCAACCATAAACGATGCCGACCAGGGATCAGCGGATGTTGCTTATAGGACTCCGCTGGCACCTTATGAGAAATCAAAGTTTTTGGGTTCCGGGGGGAGTATGGTCGCAAGGCTGAAACTTAAAGGAATTGACGGAAGGGCACCACCAGGAGTGGAGCCTGCGGCTTAATTTGACTCAACACGGGGAAACTTACCAGGTCCAGACATAGTAAGGATTGACAGACTGAGAGCTCTTTCTTGATTCTATGGGTGGTGGTGCATGGCCGTTCTTAGTTGGTGGAGCGATTTGTCTGGTTAATTCCGTTAACGAACGAGACCTCAGCCTGCTAACTAGCTACGTGGAGGCATCCCTTCACGGCCGGCTTCTTAGAGGGACTATGGCCGTTTAGGCCAAGGAAGTTTGAGGCAATAACAGGTCTGTGATGCCCTTAGATGTTCTGGGCCGCACGCGCGCTACACTGATGTATTCAACGAGTTCACACCTTGCCGACAGGCCCGGGTAATCTTTGAAATTTCATCGTGATGGGGATAGATCATTGCAATTGTTGGTCTTCAACGAGGAATTCCTAGTAAGCGCGAGTCATCAGCTCGCGTTGACTACGTCCCTGCCCTTTGTACACACCGCCCGTCGCTCCTACCGATTGAATGATCCGGTGAAGTGTTCGGATCGCGGCGACGTGGGTGGTTCGCCGCCCGCGACGTCGCGAGAAGTCCACTAAACCTTATCATTTAGAGGAAGGAGAAGTCGTAACAAGGTTTCCGTAGGTGAACCTGCGGAAGGATCATTGTCGATACCTGT
