# Supplementary material for: Overexpression of 18S rRNA methyltransferase CrBUD23 enhances biomass and lutein content in Chlamydomonas reinhardtii
Source: Front Bioeng Biotechnol. 2023 Feb 3;11:1102098. doi: 10.3389/fbioe.2023.1102098 (PMC9935685; doi:10.3389/fbioe.2023.1102098)
Supplement: Supplementary file 8 [file DataSheet1.PDF]

Color Align Conservation results

```
BbBUD23    -------------------------------------------------------------------------------- 0
DsBUD23    -------------------------------------------------------------------------------- 0
CsBUD23    -------------------------------------------------------------------------------- 0
CzBUD23    -------------------------------------------------------------------------------- 0
CrBUD23    -------------------------------------------------------------------------------- 0
VcBUD23    -------------------------------------------------------------------------------- 0
ScBUD23    -------------------------------------------------------------------------------- 0
CeBUD23    -------------------------------------------------------------------------------- 0
DmBUD23    -------------------------------------------------------------------------------- 0
DrWBSCR22  -------------------------------------------------------------------------------- 0
HsWBSCR22  -------------------------------------------------------------------------------- 0
MmWBSCR22  -------------------------------------------------------------------------------- 0
MpBUD23    -------------------------------------------------------------------------------- 0
OlBUD23    -------------------------------------------------------------------------------- 0
MtBUD23b   -------------------------------------------------------------------------------- 0
MtBUD23a   -------------------------------------------------------------------------------- 0
PpBUD23    -------------------------------------------------------------------------------- 0
AtRID2     -------------------------------------------------------------------------------- 0
OsBUD23    MVTHGGGGGGGARTIWDDEAAAAAGVMRRSPRCVGEFIGDPLLGRDIVLGLWDSRPISRVSLHRRPMKSPVERRSVVGTL 80
ZmBUD23    -------------------------------------------------------------------------------- 0

BbBUD23    -------------------------------------------------------------------------------- 0
DsBUD23    ----------------------------MGKGERPEH---QAPPEIFYSETEAAKYTSNSRIMQIQ-------------- 35
CsBUD23    ----------------------------MTRGDRPEN---AAPPEVFYNEQEARKYTTNSRMMAIQAQLTERALELLA-L 48
CzBUD23    ----------------------------MGKSERPEH---TAPPEIFYNEDEARKYTTNSRMINIQSALTQRALELLA-L 48
CrBUD23    ----------------------------MGKGERPEH---MAPPDIFYNEDEARKYTTNSRMINIQSTLTERALELLA-L 48
VcBUD23    ----------------------------MGKGERPEH---MAPPDIFYNADEARKYTTNSRMIAIQSSLTERALELLA-L 48
ScBUD23    -------------------------------MSRPEE---LAPPEIFYNDSEAHKYTGSTRVQHIQAKMTLRALELLNLQ 46
CeBUD23    ---------------------------MASFKVKPEH---TGPPDLYYNETEAAKYASNSHITAIQHEMAERALELLA-L 49
DmBUD23    ------------------------------MARRPEH---SAPPEIFYNDDEAKKYSTNTRIIEIQVEMAERALELLA-L 46
DrWBSCR22  ---------------------------MSSSCRRPEH---MAPPEVFYNEEEAKKYSQNSRMIEIQTQMSERAVELLN-L 49
HsWBSCR22  ---------------------------MASRGRRPEH---GGPPELFYDETEARKYVRNSRMIDIQTRMAGRALELLY-L 49
MmWBSCR22  ---------------------------MASRSRRPEH---SGPPELFYDQNEARKYVRNSRMIDIQTKMTERALELLC-L 49
MpBUD23    -------------------------------MSRPEF---TAPPQIFYNDVEARKYTHSSRVVEIQERLTERAVELLN-I 45
OlBUD23    -------------------------------MSRPEL---TAPADVFYNDTEARKYSQSSRVVEIQERLTERAVELLN-F 45
MtBUD23b   ------------------------------MIPKLASILLLPVLSIFRPHSQNEHWS---YLLYLKTVFPNYSLTLVLYF 47
MtBUD23a   ------------------------------MGSRPEA---VAPPEIFYDDDTARKYTSNSRNIQIQTSMTERALELLN-L 46
PpBUD23    ------------------------------MSIRPER---QAPPEIFYNDTEARKYTTSSRIVNIQAKLSERALELLA-L 46
AtRID2     ------------------------------MSNRPEL---LAPPEIFYDDTEARKYTSSSRIVEIQAKLSERALELLA-L 46
OsBUD23    GESRREHTTDLCGGGEERRGEKPRRRQRGERMPRPEV---QAPPEIFYNESEARKYTTSSRIIEIQSRITERALELLA-L 156
ZmBUD23    -------------------------------MPRPEF---QAPPDVFYNESEARKYTTSSRIIEIQSRISERALELLA-L 45

BbBUD23    -------------------------------------------------------------------------------- 0
DsBUD23    ----------------------------------------------------------AEGDLALHDLGHGLPFRPGTFD 57
CsBUD23    PD--------DGRMKMLLDLGCGSGLSGEALTEQG-HCWVGMDISEAMLDVARERE--VEGDLCLHDLGDGLPLRTGAFD 117
CzBUD23    PD--------DGTPRLLLDLGCGSGLSGEALTEAG-HMWIGMDISPAMLDVAIERE--VEGDVALHDLGHGLPLRMGTFD 117
CrBUD23    PQ--------DGLPRLLLDLGCGSGLSGEALSEAG-QVWVGLDISAAMLDVAHERE--VEGDLVLGDLGHGLPLRPGSFD 117
VcBUD23    PQ--------DGIPRLLLDLGCGSGLSGETLSEAG-HMWLGVDISEAMLDVAVERE--IEGDLVLGDLGHGLPLRPGAFD 117
ScBUD23    PC------------SFILDIGCGSGLSGEILTQEGDHVWCGLDISPSMLATGLSRE--LEGDLMLQDMGTGIPFRAGSFD 112
CeBUD23    PE---------GKSGFLLDIGCGTGMSSEVILDAG-HMFVGVDVSRPMLEIARQDEDLESGDFIHQDMGLGMPFRPGSFD 119
DmBUD23    PD--------DDESRLILDIGCGSGLSGSVLEDSE-HMWIGIDISKSMLDIAVERE--VAGDVILGDMGEGMPFKPGTFD 115
DrWBSCR22  PE--------DQP-CYLLDVGCGSGLSGDYLSEAG-HYWVGVDISTAMLDVALERE--VEGDLLLGDMGEGMPFRPGMFD 117
HsWBSCR22  PE--------NKP-CYLLDIGCGTGLSGSYLSDEG-HYWVGLDISPAMLDEAVDRE--IEGDLLLGDMGQGIPFKPGTFD 117
MmWBSCR22  PE--------GQP-SYLLDIGCGSGLSGDYISEEG-HYWVGIDISPAMLDAALDRD--TEGDLLLGDMGQGVPFRPGSFD 117
MpBUD23    PD--------DGVPRLLLDVGCGSGLSGERLTSLG-HEWIGTDISMNMLEVAQERE--VLGGVVQYDMGHGCPFRPGVFD 114
OlBUD23    PD--------DGVPRLLLDVGCGSGLSGDRLTELG-HEWIGMDLSASMLEVAKERE--VEGDVLRNDMGHGVPFRPGVFD 114
MtBUD23b   NFNSFSHFFFHFQLSCLIFVGCGSGLSGETLSEEG-HHWIGLDISPSMLNIALERE--VEGDLLLSDMGQGLGLRPGVID 124
MtBUD23a   PK--------DGVPKLLLDIGCGSGLSGEVITESG-HHWVGLDIAPSMLDIALDRE--VEGDLLLGDMGQGLGLRFGMFD 115
PpBUD23    PD--------DGVSRLLLDIGCGSGLSGETLSENG-HHWIGMDISEAMLDVALERE--TEGDLLLSDIGQGMPFRPGTLD 115
AtRID2     PE--------DGVPRFLLDIGCGSGLSGETLSEDG-HHWIGLDISASMLHVAVERE--VEGDLLLGDMGQGLGLRSGVID 115
OsBUD23    PN--------DGVPKLLLDIGCGSGLSGETLTEQG-HHWIGYDISKSMLDVALERE--AEGDLLLADMGQGLGLRPGVID 225
ZmBUD23    PN--------DGVPKLLLDIGCGSGLSGETLTEHG-HHWIGYDISKSMLDVALERE--TEGDLLLADMGQGLGLRPGVID 114

BbBUD23    ------------------------MRRFFETLYACLVRGARAVLQIYPEDASQAQLLTSAAMRAGFSGGLVVDFPHSTRA 56
DsBUD23    GAISISAVQWLCNADKRCNEPRKRLKRFFETLYACLNRGARAVLQVYPDSPAQAEMMVAAAMKVGFSGGLVVDYPHSTRA 137
CsBUD23    GAISISAVQWLCNADRRGADPRRRMRRFFETMYSCLTRGGRAVLQIYPADSSQAEMLTSAAMRAGFSGGLVVDFPHSTRA 197
CzBUD23    GAISISAVQWLCNADKASHEPRKRMKRFFETLYSSLTRGARAVLQIYPENTAQAEMLVSSAIKVGFSGGLVVDYPHSTRA 197
CrBUD23    GAISISAVQWLCNADRAGHDPRKRMKRFFETLYMSLRRGARAVLQIYPENHKQAEMLVAAAMKVGFSGGLVVDYPHSTRA 197
VcBUD23    GAISISAVQWLCNADRTGHDPRKRMKRFFETLYMSLRRGARAVLQIYPENPQQAEMLVAAAMKVGFSGGLVVDYPHSTRA 197
ScBUD23    AAISISAIQWLCNADTSYNDPKQRLMRFFNTLYAALKKGGKFVAQFYPKNDDQVDDILQSAKVAGFSGGLVVDDPESKKN 192
CeBUD23    GAISISAIQWLCHANASDENPRKRLLFFFQSLYGCLGRGSRAVFQFYPENDEQCDLIMGQAHKAGFNGGLVVDFPEAAKR 199
DmBUD23    GAISISALQWLCNADKSYHNPHKRLLKFFTTLFSCLTRTARAVFQFYPENSDQIEMVTSQAMKAGFYGGLVVDYPNSAKA 195
DrWBSCR22  GCISISALQWLCNADKKTHSPPKRLYRFFSTLYSSLARGARAVFQIYPENSEQLELITAQAMKAGFTGGMVVDYPNSSKA 197
HsWBSCR22  GCISISAVQWLCNANKKSENPAKRLYCFFASLFSVLVRGSRAVLQLYPENSEQLELITTQATKAGFSGGMVVDYPNSAKA 197
MmWBSCR22  GCISISAVQWLCNANKKSDVPARRLYCFFSSLYSALVRGARAVLQLYPENSEQLELITTQATRAGFTGGVVVDFPNSAKA 197
MpBUD23    GCISISAIQWLCNADNSLHRPRRRLASFFNHLYRCLKRGSKAVLQFYPDNAEQVEMITTSALRVGFSGGLVVDYPNSTRA 194
OlBUD23    GCVSISAVQWLCNADNSAHVPQRRLKTFFTQLYKSLKRGAKAILQIYPDGPRQAEMITTAALRVGFSGGLVVDYPNSTRA 194
MtBUD23b   GAISISAVQWLCNADRSSHNPRLRLKAFFTSLYRCLANGAKAVFQVYPENVDQRELILNAAMHAGFSGGIVVDFPHSSKK 204
MtBUD23a   GAIGISTIQWLCNADKSFHNPHLRLKAFFTSLYKCLTNGARAVFQVYPENDDQRELLSSPAMKAGFSGGIVVDYKDSPKK 195
PpBUD23    GAISISAVQWLCNADKSCNNPRLRLKAFFGTLYKCLARGARAVLQIYPESPQQLEMISSAAMKSGFSGGLVVDYPHSTRA 195
AtRID2     GAISISAVQWLCNADKSSHEPRLRLKAFFGSLYRCLSRGARAVFQVYPENIAQRELILRQALQAGFGGGLVVDYPHSTKK 195
OsBUD23    GAISISAVQWLCNADKSCHNPRLRLKAFFGSLYRCLARGARAVLQFYADNVKQSEMIVTAAMRAGFAGGVVVDWPHSSKA 305
ZmBUD23    GAISISAIQWLCNADKSSHDPRLRLKAFFGSLYRCLARGARAVLQFYADNVKQSEMIVTFAMRAGFAGGVVVDWPHSSKA 194

BbBUD23    KKYFLVLMVGSTAA---LPQAKGLHGEASDDEEELEE-------------VPVGARLSGGPHKRRKVKHFVGMGSARHPQ 120
DsBUD23    KKFFLVLMA------------KGLDGQDPEDSDEGAQ----------------HVKVSGRERKHAGKRK--HAGKPGKAP 187
CsBUD23    KKYFLVLMVGGSAA---MPAPRGMSGEASDEEEG-------------------TVAVAGRQH--SSKRR--R-VGADSAR 250
CzBUD23    KKYFLVLMVGSSSM---VPQPKGLDGIEPESDAEMAD-------------AD-GVQVMGSRH--RNKRR--KS--ADSSG 254
CrBUD23    KKYFLVLMVGTSIS---VPTAKGLDGGEPEDEEEEAA-------------AE-HVQVAGRDR--NKRRR--TGSG-K-GD 254
VcBUD23    KKYFLVLMVGTSAA---TPQAKGLDGSEPEDEEEEA-----------------QVKVAGRDR--HKRRK--TAGGGSGSG 253
ScBUD23    KKYYLVLSSGAPPQGEE----------QVNLD---------GVTM-----DEENVNLKKQ---LRQR------LKGGKDK 239
CeBUD23    KKVYLVLMTGGVVQ---LPQALTEDGEESRTQ----------IDN------------AGRRFV-WNS------RKNEKVA 247
DmBUD23    KKYYLVLMTGGSAE---LPQALGSPEEERRVN----YIK--------------------KRDACREA------R--GKAP 240
DrWBSCR22  KKFFLCLFAGVSGV---LPKGLDSETAVRGVVNQAQFTAQR--------------------SRFKNM------K--GKSA 246
HsWBSCR22  KKFYLCLFSGPSTF---IPEGLSENQDEVEP-RESVFTNEREGGAFE---RRGIRGHQTRRFPLRMS------R--RGMV 262
MmWBSCR22  KKFYLCLFSGPSTS---LPKGLTESQDADQA-SESMFTSERA--------------------PHKKA------R--RDLV 245
MpBUD23    KKYFLVLAAGSESSATEFSASTHICG-LVGDE---LL-NKPGINIVGRKKARKNLSPSRKISSVSHS------RSKRHPD 263
OlBUD23    KKYFLALAAGPPEQLPTPK-----GE-FDDDD---DI-ERRGMRMDGRKSDRS-------------G------KYKKGKN 245
MtBUD23b   RKEFLVLGCGQLSTKASLSKGKIEDEEKLSDE---------ESEDEE----NQTVRLSDRHRPVKKQ------RK-NNKS 264
MtBUD23a   RKEYLFLVCGQEVP-LPLPEGRTED--------------NDDSGSET----NKTVHVLDRRRPWKIQ------K--NNKS 248
PpBUD23    KKYFLVLSCGPPSTATALPRAKEGNEM--SED---EE-SGSECDEDG----GTTVNVSERQRPSKKQ------RKDSKKS 259
AtRID2     RKEFLVLTCGTVQ--TSIQTSKNEYDESCSED---DNSDDE-----E----SEEVGVSDRNRPRKRQ------R--TNTK 253
OsBUD23    KKSYLVLTCGPPSLNSSLPKGKGQDGAMCSDD---DESDDGSGDEDG----AQTVGIYERNRPKKRQ------K--TKKN 370
ZmBUD23    KKSYLVLTCGPPSVTTSLPKGKGENGEGCSDD---DDNE--SSGEDG----DRTVGIYERNRPKKRQ------K--TKKN 257

BbBUD23    AKDKAWVLKKKAQQRERGYPNIPADTKYTGRKRKTRF* 158
DsBUD23    TGSKEWIVHKKDTMRKRGYTEIPRDTKYTGRKRKKTV* 225
CsBUD23    AKGKAWILKKKEQARHKGYVGIPGDTKYTGRKRKTKF* 288
CzBUD23    GKGKEWLLKKKERMRHKGYEHIPPDTKYSGRKRRRTL* 292
CrBUD23    GKGREWVLRKKEQMRKKGYD-IAPDSKYTARKRKRVV* 291
VcBUD23    TKGREWILKKKEQMRKKGYD-IAADSKYTGRKRKRLV* 290
ScBUD23    ESAKSFILRKKELMKRRG-RKVAKDSKFTGRKRRHRF- 275
CeBUD23    KGSKAWIEAKRQRQIKQG-RDVRHESKYSGRKRKTKF- 283
DmBUD23    KKSRDWILAKKERRRRQG-LETRPDTKYTARKRSGKF- 276
DrWBSCR22  KKSKDWILDKKERRRRQG-KDVRADTKYTGRHRKPKF- 282
HsWBSCR22  RKSRAWVLEKKERHRRQG-REVRPDTQYTGRKRKPRF- 298
MmWBSCR22  KKSREWVLEKKERRRRQG-K------------------ 264
MpBUD23    NKGRAWIEKKKSQAQLKG-KETARNSKYTGRKRKDRI* 300
OlBUD23    IKGKAWVHKKKEQYRNRG-VQVASDSKFTGRKRKDRL* 282
MtBUD23b   GKGKEWILRKKDQMRRRG-NDVPLDTKYTGRKRKGRF* 301
MtBUD23a   EKGREWIKRKKEQMRRRG-DDVPPDTKYTGRKRKNHF* 285
PpBUD23    GKGRSWILKKKEQRRHRGYTNVPDDSKYTGRKRKAHF* 297
AtRID2     VKGREWVLRKKEQSRRKG-KNVPADSKFTSRKRRTRF* 290
OsBUD23    GKGKAWLLNKKEQLRRRG-REVPADTKYTGRKRKSYF* 407
ZmBUD23    GKGKDWLLRKKEQMRRRG-HDVPADTKYTGRKRKGYF* 294
```
