# Supplementary material for: Overexpression of 18S rRNA methyltransferase CrBUD23 enhances biomass and lutein content in Chlamydomonas reinhardtii
Source: Front Bioeng Biotechnol. 2023 Feb 3;11:1102098. doi: 10.3389/fbioe.2023.1102098 (PMC9935685; doi:10.3389/fbioe.2023.1102098)
Supplement: Supplementary file 9 [file Table3.DOCX]

>KX781337.1 P2

GGTTCGATTCCGGAGAGGGAGCCTGAGAGATGGCTACCACATCCAAGGAAGGCAGCAGGCGCGCAAATTACCCAATCCCAACACGGGGAGGTAGTGACAATAAATAACAATACCGGGCATTTCATGTCTGGTAATTGGAATGAGTACAATCTAAATCCCTTAACGAGGATCCATTGGAGGGCAAGTCTGGTGCCAGCAGCCGCGGTAATTCCAGCTCCAATAGCGTATATTTAAGTTGTTGCAGTTAAAAAGCTCGTAGTTGGATTTCGGGTGGGTCTTAGCGGTCCGCCTCTGGTGTGTACTGCTAGGGCCTATCTTTCTGCCGGGGACGGGCTCCTGGGTTTAATCGCCTGGGACTCGGAGTCGGCGAGGTTACTTTGAGTAAATTAGAGTGTTCAAAGCAAGCCTACGCTCTGAATACATTAGCATGGAATAACACGATAGGACTCTGGCCTATCTTGTTGGTCTGTAGGACCGGAGTAATGATTAAGAGGGACAGTCGGGGGCATTCGTATTTCATTGTCAGAGGTGAAATTCTTGGATTTATGAAAGACGAACTTCTGCGAAAGCATTTGCCAAGGATGTTTTCATTAATCAAGAACGAAAGTTGGGGGCTCGAAGACGATTAGATACCGTCGTAGTCTCAACCATAAACGATGCCGACTAGGGATTGGCAGATGTTTCATTGATGACTCTGCCAGCACCTTATGAGAAATCAAAGTTTTTGGGTTCCGGGGGGAGTATGGTCGCAAGGCTGAAACTTAAAGGAATTGACGGAAGGGCACCACCAGGCGTGGAGCCTGCGGCTTAATTTGACTCAACACGGGAAAACTTACCAGGTCCAGACACGGGAAGGATTGACAGATTGAGAGCTCTTTCTTGATTCTGTGGGTGGTGGTGCATGGCCGTTCTTAGTTGGTGGGTTGCCTTGTCAGGTTGATTCCGGTAACGAACGAGACCTCAGCCTGCTAAATAGTCACGACTGCTTTTTGCAGTTGGCCGACTTCTTAGAGGGACTATTGTCGTGTAGGCAATGGAAGTATGAGGCAATAACAGGTCTGTGATGCCCTTAGATGTTCTGGGCCGCACGCGCGCTACACTGACGCATTCAACGAGCCTATCCTTGGCCGAGAGGCCCGGGTAATCTTTGAAACTGCGTCGTGATGGGGATAGATTATTGCAATTATTAGTCTTCAACGAGGAATGCCTAGTAAGCGCGAGTCATCAGCTCGCGTTGATTACGTCCCTGCCCTTTGTACACACCGCCCGTCGCTCCTACCGATTGGGTGTGCTGGTGAAGTGTTCGGATTGGCTTCAGGTGATGGCAACA

>KX781338.1 P1

CTGGGACTCGGAGTCGGCGAGGTTACTTTGAGTAAATTAGAGTGTTCAAAGCAAGCCTACGCTCTGAATACATTAGCATGGAATAACACGATAGGACTCTGGCCTATCTTGTTGGTCTGTAGGACCGGAGTAATGATTAAGAGGGACAGTCGGGGGCATTCGTATTTCATTGTCAGAGGTGAAATTCTTGGATTTATGAAAGACGAACTTCTGCGAAAGCATTTGCCAAGGATGTTTTCATTAATCAAGAACGAAAGTTGGGGGCTCGAAGACGATTAGATACCGTCGTAGTCTCAACCATAAACGATGCCGACTAGGGATTGGCAGATGTTTCATTGATGACTCTGCCAGCACCTTATGAGAAATCAAAGTTTTTGGGTTCCGGGGGGAGTATGGTCGCAAGGCTGAAACTTAAAGGAATTGACGGAAGGGCACCACCAGGCGTGGAGCCTGCGGCTTAATTTGACTCAACACGGGAAAACTTACCAGGTCCAGACACGGGAAGGATTGACAGATTGAGAGCTCTTTCTTGATTCTGTGGGTGGTGGTGCATGGCCGTTCTTAGTTGGTGGGTTGCCTTGTCAGGTTGATTCCGGTAACGAACGAGACCTCAGCCTGCTAAATAGTCACGACTGCTTTTTGCAGTTGGCCGACTTCTTAGAGGGACTATTGTCGTGTAGGCAATGGAAGTATGAGGCAATAACAGGTCTGTGATGCCCTTAGATGTTCTGGGCCGCACGCGCGCTACACTGACGCATTCAACGAGCCTATCCTTGGCCGAGAGGCCCGGGTAATCTTTGAAACTGCGTCGTGATGGGGATAGATTATTGCAATTATTAGTCTTCAACGAGGAATGCCTAGTAAGCGCGAGTCATCAGCTCGCGTTGATTACGTCCCTGCCCTTTGTACACACCGCCCGTCGCTCCTACCG

>JN903978.1 SAG11-32c

AGTCATATGCTTGTCTCAAAGATTAAGCCATGCATGTCTAAGTATAAACTGCTTATACTGTGAAACTGCGAATGGCTCATTAAATCAGTTATAGTTTATTTGATGGTACCTACTACTCGGATAACCGTAGTAATTCTAGAGCTAATACGTGCGCACAACCCGACTTCTGGAAGGGTCGTATTTATTAGATAAAAGGCCAGCCGGGCTCTGCCCGACCTGCGGTGAATCATGATAACTTCACGAATCGTATGGGCTCGTCCCGACGATGTTTCATTCAAATTTCTGCCCTATCAACTTTCGATGGTAGGATAGAGGCCTACCATGGTGGTAACGGGTGACGGAGGATTAGGGTTCGATTCCGGAGAGGGAGCCTGAGAGATGGCTACCACATCCAAGGAAGGCAGCAGGCGCGCAAATTACCCAATCCCGACACGGGGAGGTAGTGACAATAAATAACAATACCGGGCGCTTCGCGTCTGGTAATTGGAATGAGTACAATCTAAATCCCTTAACGAGGATCCATTGGAGGGCAAGTCTGGTGCCAGCAGCCGCGGTAATTCCAGCTCCAATAGCGTATATTTAAGTTGTTGCAGTTAAAAAGCTCGTAGTTGGATTTCGGGTGGGGTGGTGCGGTCCGCCTCTGGTGTGCACTGCTCTGCTCCACCTTCCTGCCGGGGACGGGCTCCTGGGCTTCACTGTCTGGGACTCGGAGTCGGCGAGGTTACTTTGAGTAAATTAGAGTGTTCAAAGCAGGCCTACGCTCTGAATACATTAGCATGGAATAACACGATAGGACTCTGGCCTATCTGTTGGTCTGTGGGACCGGAGTAATGATTAAGAGGGGTAGTCGGGGGCATTCGTATTCCGTTGTCAGAGGTGAAATTCTTGGATTTACGGAAGACGAACATCTGCGAAAGCATTTGCCAAGGATACTTTCATTGATCAAGAACGAAAGTTGGGGGCTCGAAGACGATTAGATACCGTCGTAGTCTCAACCATAAACGATGCCGACTAGGGATTGGCAGATGTTCTTTTGATGACTCTGCCAGCACCTTATGAGAAATCAAAGTTTTTGGGTTCCGGGGGGAGTATGGTCGCAAGGCTGAAACTTAAAGGAATTGACGGAAGGGCACCACCAGGCGTGGAGCCTGCGGCTTAATTTGACTCAACACGGGGAAACTTACCAGGTCCAGACACGGGAAGGATTGACAGATTGAGAGCTCTTTCTTGATTCTGTGGGTGGTGGTGCATGGCCGTTCTTAGTTGGTGGGTTGCCTTGTCAGGTTGATTCCGGTAACGAACGAGACCTCAGCCTGCTAAATAGTCAGCATCGCACCTGCGGTGCGCCGACTTCTTAGAGGGACTATTGGCGTTTAGCCAATGGAAGTATGAGGCGATAACAGGTCTGTGATGCCCTTAGATGTTCTGGGCCGCACGCGCGCTACACTGACGCGACCAACGAGCCTATCCTTGGCCGAGAGGCCCGGGTAATCTTGTAAACCGCGTCGTGATGGGGATAGATTATTGCAATTATTAGTCTTCAACGAGGAATGCCTAGTAAGCGCGAGTCATCAGCTCGCGTTGATTACGTCCCTGCCCTTTGTACACACCGCCCGTCGCTCCTACCGATTGGGTGTGCTGGTGAAGTGTTCGGATTGAGCTTGGCTGGGGCAACCTGGCCTTGCTTGAGAAGTTCATTAAACCCTCCCACCTAGAGGAAGGAGAAGTCGTAACAAGGTTTC

>JN903974.1 SAG18.79

AGTCATATGCTGTCTCAAAGATTAAGCCATGCATGTCTAAGTATAAACTGCTTATACTGTGAAACTGCGAATGGCTCATTAAATCAGTTATAGTTTATTTGATGGTACCTACTACTCGGATAACCGTAGTAATTCTAGAGCTAATACGTGCGCACAACCCGACTTCTGGAAGGGTCGTATTTATTAGATAAAAGGCCAGCCGGGCTCTGCCCGACCTGCGGTGAATCATGATAACTTCACGAATCGTATGGGCTCGTCCCGACGATGTTTCATTCAAATTTCTGCCCTATCAACTTTCGATGGTAGGATAGAGGCCTACCATGGTGGTAACGGGTGACGGAGGATTAGGGTTCGATTCCGGAGAGGGAGCCTGAGAGATGGCTACCACATCCAAGGAAGGCAGCAGGCGCGCAAATTACCCAATCCCGACACGGGGAGGTAGTGACAATAAATAACAATACCGGGCGCTTCGCGTCTGGTAATTGGAATGAGTACAATCTAAATCCCTTAACGAGGATCCATTGGAGGGCAAGTCTGGTGCCAGCAGCCGCGGTAATTCCAGCTCCAATAGCGTATATTTAAGTTGTTGCAGTTAAAAAGCTCGTAGTTGGATTTCGGGTGGGGTGGTGCGGTCCGCCTCTGGTGTGCACTGCTCTGCTCCACCTTCCTGCCGGGGACGGGCTCCTGGGCTTCACTGTCTGGGACTCGGAGTCGGCGAGGTTACTTTGAGTAAATTAGAGTGTTCAAAGCAGGCCTACGCTCTGAATACATTAGCATGGAATAACACGATAGGACTCTGGCCTATCTGTTGGTCTGTGGGACCGGAGTAATGATTAAGAGGGGTAGTCGGGGGCATTCGTATTCCGTTGTCAGAGGTGAAATTCTTGGATTTACGGAAGACGAACATCTGCGAAAGCATTTGCCAAGGATACTTTCATTGATCAAGAACGAAAGTTGGGGGCTCGAAGACGATTAGATACCGTCGTAGTCTCAACCATAAACGATGCCGACTAGGGATTGGCAGATGTTCTTTTGATGACTCTGCCAGCACCTTATGAGAAATCAAAGTTTTTGGGTTCCGGGGGGAGTATGGTCGCAAGGCTGAAACTTAAAGGAATTGACGGAAGGGCACCACCAGGCGTGGAGCCTGCGGCTTAATTTGACTCAACACGGGGAAACTTACCAGGTCCAGACACGGGAAGGATTGACAGATTGAGAGCTCTTTCTTGATTCTGTGGGTGGTGGTGCATGGCCGTTCTTAGTTGGTGGGTTGCCTTGTCAGGTTGATTCCGGTAACGAACGAGACCTCAGCCTGCTAAATAGTCAGCATCGCACCTGCGGTGCGCCGACTTCTTAGAGGGACTATTGGCGTTTAGCCAATGGAAGTATGAGGCGATAACAGGTCTGTGATGCCCTTAGATGTTCTGGGCCGCACGCGCGCTACACTGACGCGACCAACGAGCCTATCCTTGGCCGAGAGGCCCGGGTAATCTTGTAAACCGCGTCGTGATGGGGATAGATTATTGCAATTATTAGTCTTCAACGAGGAATGCCTAGTAAGCGCGAGTCATCAGCTCGCGTTGATTACGTCCCTGCCCTTTGTACACACCGCCCGTCGCTCCTACCGATTGGGTGTGCTGGTGAAGTGTTCGGATTGAGCTTGGCTGGGGCAACCTGGCCTTGCTTGAGAAGTTCATTAAACCCTCCCACCTAGAGGAAGGAGAAGTCGTAACAAGGTTTC

>JN903984.1 SAG53.72

AGTCATATGCTTGTCTCAAAGATTAAGCCATGCATGTCTAAGTATAAACTGCTTTATACTGTGAAACTGCGAATGGCTCATTAAATCAGTTATAGTTTATTTGATGGTACCTACTACTCGGATAACCGTAGTAATTCTAGAGCTAATACGTGCGTAAATCCCGACTTCTGGAAGGGACGTATTTATTAGATAAAAGGCCAGCCGGGCTTTGCCCGACCTGCGGTGAATCATGATAACTTCACGAATCGCATGGCCTTGCGCCGGCGATGTTTCATTCAAATTTCTGCCCTATCAACTTTCGATGGTAGGATAGAGGCCTACCATGGTGGTAACGGGTGACGGAGGATTAGGGTTCGATTCCGGAGAGGGAGCCTGAGAGATGGCTACCACATCCAAGGAAGGCAGCAGGCGCGCAAATTACCCAATCCCAACACGGGGAGGTAGTGACAATAAATAACAATACCGGGCATTTCATGTCTGGTAATTGGAATGAGTACAATCTAAATCCCTTAACGAGGATCCATTGGAGGGCAAGTCTGGTGCCAGCAGCCGCGGTAATTCCAGCTCCAATAGCGTATATTTAAGTTGTTGCAGTTAAAAAGCTCGTAGTTGGATTTCGGGTGGGTCTTAGCGGTCCGCCTCTGGTGTGTACTGCTAGGGCCTATCTTTCTGCCGGGGACGGGCTCCTGGGTTTAATCGCCTGGGACTCGGAGTCGGCGAGGTTACTTTGAGTAAATTAGAGTGTTCAAAGCAAGCCTACGCTCTGAATACATTAGCATGGAATAACACGATAGGACTCTGGCCTATCTTGTTGGTCTGTAGGACCGGAGTAATGATTAAGAGGGACAGTCGGGGGCATTCGTATTTCATTGTCAGAGGTGAAATTCTTGGATTTATGAAAGACGAACTTCTGCGAAAGCATTTGCCAAGGATGTTTTCATTAATCAAGAACGAAAGTTGGGGGCTCGAAGACGATTAGATACCGTCGTAGTCTCAACCATAAACGATGCCGACTAGGGATTGGCAGATGTTTCATTGATGACTCTGCCAGCACCTTATGAGAAATCAAAGTTTTTGGGTTCCGGGGGGAGTATGGTCGCAAGGCTGAAACTTAAAGGAATTGACGGAAGGGCACCACCAGGCGTGGAGCCTGCGGCTTAATTTGACTCAACACGGGAAAACTTACCAGGTCCAGACACGGGAAGGATTGACAGATTGAGAGCTCTTTCTTGATTCTGTGGGTGGTGGTGCATGGCCGTTCTTAGTTGGTGGGTTGCCTTGTCAGGTTGATTCCGGTAACGAACGAGACCTCAGCCTGCTAAATAGTCACGACTGCTTTTTGCAGTTGGCCGACTTCTTAGAGGGACTATTGTCGTGTAGGCAATGGAAGTATGAGGCAATAACAGGTCTGTGATGCCCTTAGATGTTCTGGGCCGCACGCGCGCTACACTGACGCATTCAACGAGCCTATCCTTGGCCGAGAGGCCCGGGTAATCTTTGAAACTGCGTCGTGATGGGGATAGATTATTGCAATTATTAGTCTTCAACGAGGAATGCCTAGTAAGCGCGAGTCATCAGCTCGCGTTGATTACGTCCCTGCCCTTTGTACACACCGCCCGTCGCTCCTACCGATTGGGTGTGCTGGTGAAGTGTTCGGATTGGCTTCAGGTGATGGCAACATCGCCTGTTGCTGAGAAGTTCATTAAACCCTCCCACCTAGAGGAAGGAGAAGTCGTAACAAGGTTTC

>KC166137.1 K01

GCAGNCGGGCTCTGCCCGACCTGCGGTGAATCATGATAACTTCACGAATCGTATGGGCTCGTCCCGACGATGTTTCATTCAAATTTCTGCCCTATCAACTTTCGATGGTAGGATAGAGGCCTACCATGGTGGTAACGGGTGACGGAGGATTAGGGTTCGATTCCGGAGAGGGAGCCTGAGAGATGGCTACCACATCCAAGGAAGGCAGCAGGCGCGCAAATTACCCAATCCCGACACGGGGAGGTAGTGACAATAAATAACAATACCGGGCGCTTCGCGTCTGGTAATTGGAATGAGTACAATCTAAATCCCTTAACGAGGATCCATTGGAGGGCAAGTCTGGTGCCAGCAGCCGCGGTAATTCCAGCTCCAATAGCGTATATTTAAGTTGTTGCAGTTAAAAAGCTCGTAGTTGGATTTCGGGTGGGGGTGGTGCGGTCCGCCTCTGGTGTGCACTGCTCTGCTCCACCTTCCTGCCGGGGACGGGCTCCTGGGCTTCACTGTCTGGGACTCGGAGTCGGCGAGGTTACTTTGAGTAAATTAGAGTGTTCAAAGCAGGCCTACGCTCTGAATACATTAGCATGGAATAACACGATAGGACTCTGGCCTATCTGTTGGTCTGTGGGACCGGAGTAATGATTAAGAGGGGTAGTCGGGGGCATTCGTATTCCGTTGTCAGAGGTGAAATTCTTGGATTTACGGAAGACGAACATCTGCGAAAGCATTTGCCAAGGATACTTTCATTGATCAAGAACGAAAGTTGGGGGCTCGAAGACGATTAGATACCGTCGTAGTCTCAACCATAAACGATGCCGACTAGGGATTGGCAGATGTTCTTTTGATGACTCTGCCAGCACCTTATGAGAAATCAAAGTTTTTGGGTTCCGGGGGGAGTATGGTCGCAAGGCTGAAACTTAAAGGAATTGACGGAAGGGCACCACCAGGCGTGGAGCCTGCGGCTTAATTTGACTCAACACGGGGAAACTTACCAGGTCCAGACACGGGAAGGATTGACAGATTGAGAGCTCTTTCTTGATTCTGTGGGTGGTGGTGCATGGCCGTTCTTAGTTGGTGGGTTGCCTTGTCAGGTTGATTCCGGTAACGAACGAGACCTCAGCCTGCTAAATAGTCAGCATCGCACCTGCGGTGCGCCGACTTCTTAGAGGGACTATTGGCGTTTAGCCAATGGAAGTATGAGGCGATAACAGGTCTGTGATGCCCTTAGATGTTCTGGGCCGCACGCGCGCTACACTGACGCGACCAACGAGCCTATCCTTGGCCGAGAGGCCCGGGTAATCTTGTAAACCGCGTCGTGATGGGGATAGATTATTGCAATTATTAGTCTTCAACGAGGAATGCCTAGTAAGCGCGAGTCATCAGCTCGCGTTGATTACGTCCCTGCCCTTTGTACACACCGCCCGTCGCTCCTACCGATTGGGTGTGCTGGTGAAGTGTTCGGATGAGCTTGGCTGGGCAACCT

>KC310450.1 RAC

TGCATGGCCGTTCTTAGTTGGTGGGTTGCCTTGTCAGGTTGATTCCGGTAACGAACGAGACCTCAGCCTGCTAAATAGTCAGCATCGCACCTGCGGTGCGCCGACTTCTTAGAGGGACTATTGGCGTTTAGCCAATGGAAGTATGAGGCGATAACAGGTCTGTGATGCCCTTAGATGTTCTGGGCCGCACGCGCGCTACACTGACGCGACCAACGAGCCTATCCTTGGCCGAGAGGCCCGGGTAATCTTGTAAACCGCGTCGTGATGGGGATAGATTATTGCAATTATTAGTCTTCAACGAGGAATGCCTAGTAAGCGCGAGTCATCAGCTCGCGTTGATTACGTCCCTGCCCTTTGTACACACCGCCCGTCGCTCCTACCGATTGGGTGTGCTGGTGAAGTGTTCGGATTGAGCTTGGCTGGGGCAACTTGGCCTTGCTTGAGAAGTTCATTAAACCCTCCCACCTAGAGGAAGGAGAAGTCGTAACAAGGTTTCCGTAGGTGAA

>JN863299.1 KNUA021

GTAGTCATATGCTTGTCTCAAAGATTAAGCCATGCATGTCTAAGTATAAACTGCTTATACTGTGAAACTGCGAATGGCTCATTAAATCAGTTATAGTTTATTTGATGGTACCTACTACTCGGATAACCGTAGTAATTCTAGAGCTAATACGTGCGCACAACCCGACTTCTGGAAGGGTCGTATTTATTAGATAAAAGGCCAGCCGGGCTCTGCCCGACCTGCGGTGAATCATGATAACTTCACGAATCGTATGGGCTCGTCCCGACGATGTTTCATTCAAATTTCTGCCCTATCAACTTTCGATGGTAGGATAGAGGCCTACCATGGTGGTAACGGGTGACGGAGGATTAGGGTTCGATTCCGGAGAGGGAGCCTGAGAGATGGCTACCACATCCAAGGAAGGCAGCAGGCGCGCAAATTACCCAATCCCGACACGGGGAGGTAGTGACAATAAATAACAATACCGGGCGCTTCGCGTCTGGTAATTGGAATGAGTACAATCTAAATCCCTTAACGAGGATCCATTGGAGGGCAAGTCTGGTGCCAGCAGCCGCGGTAATTCCAGCTCCAATAGCGTATATTTAAGTTGTTGCAGTTAAAAAGCTCGTAGTTGGATTTCGGGTGGGGTGGTGCGGTCCGCCTCTGGTGTGCACTGCTCTGCTCCACCTTCCTGCCGGGGACGGGCTCCTGGGCTTCACTGTCTGGGACTCGGAGTCGGCGAGGTTACTTTGAGTAAATTAGAGTGTTCAAAGCAGGCCTACGCTCTGAATACATTAGCATGGAATAACACGATAGGACTCTGGCCTATCTGTTGGTCTGTGGGACCGGAGTAATGATTAAGAGGGGTAGTCGGGGGCATTCGTATTCCGTTGTCAGAGGTGAAATTCTTGGATTTACGGAAGACGAACATCTGCGAAAGCATTTGCCAAGGATACTTTCATTGATCAAGAACGAAAGTTGGGGGCTCGAAGACGATTAGATACCGTCGTAGTCTCAACCATAAACGATGCCGACTAGGGATTGGCAGATGTTCTTTTGATGACTCTGCCAGCACCTTATGAGAAATCAAAGTTTTTGGGTTCCGGGGGGAGTATGGTCGCAAGGCTGAAACTTAAAGGAATTGACGGAAGGGCACCACCAGGCGTGGAGCCTGCGGCTTAATTTGACTCAACACGGGGAAACTTACCAGGTCCAGACACGGGAAGGATTGACAGATTGAGAGCTCTTTCTTGATTCTGTGGGTGGTGGTGCATGGCCGTTCTTAGTTGGTGGGTTGCCTTGTCAGGTTGATTCCGGTAACGAACGAGACCTCAGCCTGCTAAATAGTCAGCATCGCACCTGCGGTGCGCCGACTTCTTAGAGGGACTATTGGCGTTTAGCCAATGGAAGTATGAGGCGATAACAGGTCTGTGATGCCCTTAGATGTTCTGGGCCGCACGCGCGCTACACTGACGCGACCAACGAGCCTATCCTTGGCCGAGAGGCCCGGGTAATCTTGTAAACCGCGTCGTGATGGGGATAGATTATTGCAATTATTAGTCTTCAACGAGGAATGCCTAGTAAGCGCGAGTCATCAGCTCGCGTTGACTACGTCCCTGCCCTTTGTACACACCGCCCGTCGCTCCTACCGATTGGGTGTGCTGGTGAAGTGTTCGGATTGAGCTTGGCTGGGGCAACCTGGCCTTGCTTGAGAAGTTCATTAAACCCTCCCACCTAGAGGAAGGAGAAGTCGTAACAAGGTTTCCGTAGGTGAACCTGCGGA

>JX888471.1 CC-620

GTAGTCATATGCTTGTCTCAAAGATTAAGCCATGCATGTCTAAGTATAAACTGCTTATACTGTGAAACTGCGAATGGCTCATTAAATCAGTTATAGTTTATTTGATGGTACCTACTACTCGGATAACCGTAGTAATTCTAGAGCTAATACGTGCGCACAACCCGACTTCTGGAAGGGTCGTATTTATTAGATAAAAGGCCAGCCGGGCTCTGCCCGACCTGCGGTGAATCATGATAACTTCACGAATCGTATGGGCTCGTCCCGACGATGTTTCATTCAAATTTCTGCCCTATCAACTTTCGATGGTAGGATAGAGGCCTACCATGGTGGTAACGGGTGACGGAGGATTAGGGTTCGATTCCGGAGAGGGAGCCTGAGAGATGGCTACCACATCCAAGGAAGGCAGCAGGCGCGCAAATTACCCAATCCCGACACGGGGAGGTAGTGACAATAAATAACAATACCGGGCGCTTCGCGTCTGGTAATTGGAATGAGTACAATCTAAATCCCTTAACGAGGATCCATTGGAGGGCAAGTCTGGTGCCAGCAGCCGCGGTAATTCCAGCTCCAATAGCGTATATTTAAGTTGTTGCAGTTAAAAAGCTCGTAGTTGGATTTCGGGTGGGGTGGTGCGGTCCGCCTCTGGTGTGCACTGCTCTGCTCCACCTTCCTGCCGGGGACGGGCTCCTGGGCTTCACTGTCTGGGACTCGGAGTCGGCGAGGTTACTTTGAGTAAATTAGAGTGTTCAAAGCAGGCCTACGCTCTGAATACATTAGCATGGAATAACACGATAGGACTCTGGCCTATCTGTTGGTCTGTGGGACCGGAGTAATGATTAAGAGGGGTAGTCGGGGGCATTCGTATTCCGTTGTCAGAGGTGAAATTCTTGGATTTACGGAAGACGAACATCTGCGAAAGCATTTGCCAAGGATACTTTCATTGATCAAGAACGAAAGTTGGGGGCTCGAAGACGATTAGATACCGTCGTAGTCTCAACCATAAACGATGCCGACTAGGGATTGGCAGATGTTCTTTTGATGACTCTGCCAGCACCTTATGAGAAATCAAAGTTTTTGGGTTCCGGGGGGAGTATGGTCGCAAGGCTGAAACTTAAAGGAATTGACGGAAGGGCACCACCAGGCGTGGAGCCTGCGGCTTAATTTGACTCAACACGGGGAAACTTACCAGGTCCAGACACGGGAAGGATTGACAGATTGAGAGCTCTTTCTTGATTCTGTGGGTGGTGGTGCATGGCCGTTCTTAGTTGGTGGGTTGCCTTGTCAGGTTGATTCCGGTAACGAACGAGACCTCAGCCTGCTAAATAGTCAGCATCGCACCTGCGGTGCGCCGACTTCTTAGAGGGACTATTGGCGTTTAGCCAATGGAAGTATGAGGCGATAACAGGTCTGTGATGCCCTTAGATGTTCTGGGCCGCACGCGCGCTACACTGACGCGACCAACGAGCCTATCCTTGGCCGAGAGGCCCGGGTAATCTTGTAAACCGCGTCGTGATGGGGATAGATTATTGCAATTATTAGTCTTCAACGAGGAATGCCTAGTAAGCGCGAGTCATCAGCTCGCGTTGATTACGTCCCTGCCCTTTGTACACACCGCCCGTCGCTCCTACCGATTGGGTGTGCTGGTGAAGTGTTCGGATTGAGCTTGGCTGGGGCAACCTGGCCTTGCTTGAGAAGTTCATTAAACCCTCCCACCTAGAGGAAGGAGAAGTCGTAACAAGGTTTCCGTAGGTGAACCTGCGGA

>JX888472.1 CC-621

GTAGTCATATGCTTGTCTCAAAGATTAAGCCATGCATGTCTAAGTATAAACTGCTTATACTGTGAAACTGCGAATGGCTCATTAAATCAGTTATAGTTTATTTGATGGTACCTACTACTCGGATAACCGTAGTAATTCTAGAGCTAATACGTGCGCACAACCCGACTTCTGGAAGGGTCGTATTTATTAGATAAAAGGCCAGCCGGGCTCTGCCCGACCTGCGGTGAATCATGATAACTTCACGAATCGTATGGGCTCGTCCCGACGATGTTTCATTCAAATTTCTGCCCTATCAACTTTCGATGGTAGGATAGAGGCCTACCATGGTGGTAACGGGTGACGGAGGATTAGGGTTCGATTCCGGAGAGGGAGCCTGAGAGATGGCTACCACATCCAAGGAAGGCAGCAGGCGCGCAAATTACCCAATCCCGACACGGGGAGGTAGTGACAATAAATAACAATACCGGGCGCTTCGCGTCTGGTAATTGGAATGAGTACAATCTAAATCCCTTAACGAGGATCCATTGGAGGGCAAGTCTGGTGCCAGCAGCCGCGGTAATTCCAGCTCCAATAGCGTATATTTAAGTTGTTGCAGTTAAAAAGCTCGTAGTTGGATTTCGGGTGGGGTGGTGCGGTCCGCCTCTGGTGTGCACTGCTCTGCTCCACCTTCCTGCCGGGGACGGGCTCCTGGGCTTCACTGTCTGGGACTCGGAGTCGGCGAGGTTACTTTGAGTAAATTAGAGTGTTCAAAGCAGGCCTACGCTCTGAATACATTAGCATGGAATAACACGATAGGACTCTGGCCTATCTGTTGGTCTGTGGGACCGGAGTAATGATTAAGAGGGGTAGTCGGGGGCATTCGTATTCCGTTGTCAGAGGTGAAATTCTTGGATTTACGGAAGACGAACATCTGCGAAAGCATTTGCCAAGGATACTTTCATTGATCAAGAACGAAAGTTGGGGGCTCGAAGACGATTAGATACCGTCGTAGTCTCAACCATAAACGATGCCGACTAGGGATTGGCAGATGTTCTTTTGATGACTCTGCCAGCACCTTATGAGAAATCAAAGTTTTTGGGTTCCGGGGGGAGTATGGTCGCAAGGCTGAAACTTAAAGGAATTGACGGAAGGGCACCACCAGGCGTGGAGCCTGCGGCTTAATTTGACTCAACACGGGGAAACTTACCAGGTCCAGACACGGGAAGGATTGACAGATTGAGAGCTCTTTCTTGATTCTGTGGGTGGTGGTGCATGGCCGTTCTTAGTTGGTGGGTTGCCTTGTCAGGTTGATTCCGGTAACGAACGAGACCTCAGCCTGCTAAATAGTCAGCATCGCACCTGCGGTGCGCCGACTTCTTAGAGGGACTATTGGCGTTTAGCCAATGGAAGTATGAGGCGATAACAGGTCTGTGATGCCCTTAGATGTTCTGGGCCGCACGCGCGCTACACTGACGCGACCAACGAGCCTATCCTTGGCCGAGAGGCCCGGGTAATCTTGTAAACCGCGTCGTGATGGGGATAGATTATTGCAATTATTAGTCTTCAACGAGGAATGCCTAGTAAGCGCGAGTCATCAGCTCGCGTTGATTACGTCCCTGCCCTTTGTACACACCGCCCGTCGCTCCTACCGATTGGGTGTGCTGGTGAAGTGTTCGGATTGAGCTTGGCTGGGGCAACCTGGCCTTGCTTGAGAAGTTCATTAAACCCTCCCACCTAGAGGAAGGAGAAGTCGTAACAAGGTTTCCGTAGGTGAACCTGCGGA

>AB511834.1 UTEX 90

CCATGCATGTCTAAGTATAAACTGCTTATACTGTGAAACTGCGAATGGCTCATTAAATCAGTTATAGTTTATTTGATGGTACCTACTACTCGGATAACCGTAGTAATTCTAGAGCTAATACGTGCGCACAACCCGACTTCTGGAAGGGTCGTATTTATTAGATAAAAGGCCAGCCGGGCTCTGCCCGACCTGCGGTGAATCATGATAACTTCACGAATCGTATGGGCTCGTCCCGACGATGTTTCATTCAAATTTCTGCCCTATCAACTTTCGATGGTAGGATAGAGGCCTACCATGGTGGTAACGGGTGACGGAGGATTAGGGTTCGATTCCGGAGAGGGAGCCTGAGAGATGGCTACCACATCCAAGGAAGGCAGCAGGCGCGCAAATTACCCAATCCCGACACGGGGAGGTAGTGACAATAAATAACAATACCGGGCGCTTCGCGTCTGGTAATTGGAATGAGTACAATCTAAATCCCTTAACGAGGATCCATTGGAGGGCAAGTCTGGTGCCAGCAGCCGCGGTAATTCCAGCTCCAATAGCGTATATTTAAGTTGTTGCAGTTAAAAAGCTCGTAGTTGGATTTCGGGTGGGGTGGTGCGGTCCGCCTCTGGTGTGCACTGCTCTGCTCCACCTTCCTGCCGGGGACGGGCTCCTGGGCTTCACTGTCTGGGACTCGGAGTCGGCGAGGTTACTTTGAGTAAATTAGAGTGTTCAAAGCAGGCCTACGCTCTGAATACATTAGCATGGAATAACACGATAGGACTCTGGCCTATCTGTTGGTCTGTGGGACCGGAGTAATGATTAAGAGGGGTAGTCGGGGGCATTCGTATTCCGTTGTCAGAGGTGAAATTCTTGGATTTACGGAAGACGAACATCTGCGAAAGCATTTGCCAAGGATACTTTCATTGATCAAGAACGAAAGTTGGGGGCTCGAAGACGATTAGATACCGTCGTAGTCTCAACCATAAACGATGCCGACTAGGGATTGGCAGATGTTCTTTTGATGACTCTGCCAGCACCTTATGAGAAATCAAAGTTTTTGGGTTCCGGGGGGAGTATGGTCGCAAGGCTGAAACTTAAAGGAATTGACGGAAGGGCACCACCAGGCGTGGAGCCTGCGGCTTAATTTGACTCAACACGGGGAAACTTACCAGGTCCAGACACGGGAAGGATTGACAGATTGAGAGCTCTTTCTTGATTCTGTGGGTGGTGGTGCATGGCCGTTCTTAGTTGGTGGGTTGCCTTGTCAGGTTGATTCCGGTAACGAACGAGACCTCAGCCTGCTAAATAGTCAGCATCGCACCTGCGGTGCGCCGACTTCTTAGAGGGACTATTGGCGTTTAGCCAATGGAAGTATGAGGCGATAACAGGTCTGTGATGCCCTTAGATGTTCTGGGCCGCACGCGCGCTACACTGACGCGACCAACGAGCCTATCCTTGGCCGAGAGGCCCGGGTAATCTTGTAAACCGCGTCGTGATGGGGATAGATTATTGCAATTATTAGTCTTCAACGAGGAATGCCTAGTAAGCGCGAGTCATCAGCTCGCGTTGATTACGTCCCTGCCCTTTGTACACACCGCCCGTCGCTCCTACCGATTGGGTGTGCTGGTGAAGTGTTCGGATTGAGCTTGGCTGGGGCAACCTGGCCTTGCTTGAGAAGTTCATTAAACCCTCCCACCTA

>AB511835.1 SAG 11-32a

CCATGCATGTCTAAGTATAAACTGCTTATACTGTGAAACTGCGAATGGCTCATTAAATCAGTTATAGTTTATTTGATGGTACCTACTACTCGGATAACCGTAGTAATTCTAGAGCTAATACGTGCGCACAACCCGACTTCTGGAAGGGTCGTATTTATTAGATAAAAGGCCAGCCGGGCTCTGCCCGACCTGCGGTGAATCATGATAACTTCACGAATCGTATGGGCTCGTCCCGACGATGTTTCATTCAAATTTCTGCCCTATCAACTTTCGATGGTAGGATAGAGGCCTACCATGGTGGTAACGGGTGACGGAGGATTAGGGTTCGATTCCGGAGAGGGAGCCTGAGAGATGGCTACCACATCCAAGGAAGGCAGCAGGCGCGCAAATTACCCAATCCCGACACGGGGAGGTAGTGACAATAAATAACAATACCGGGCGCTTCGCGTCTGGTAATTGGAATGAGTACAATCTAAATCCCTTAACGAGGATCCATTGGAGGGCAAGTCTGGTGCCAGCAGCCGCGGTAATTCCAGCTCCAATAGCGTATATTTAAGTTGTTGCAGTTAAAAAGCTCGTAGTTGGATTTCGGGTGGGGTGGTGCGGTCCGCCTCTGGTGTGCACTGCTCTGCTCCACCTTCCTGCCGGGGACGGGCTCCTGGGCTTCACTGTCTGGGACTCGGAGTCGGCGAGGTTACTTTGAGTAAATTAGAGTGTTCAAAGCAGGCCTACGCTCTGAATACATTAGCATGGAATAACACGATAGGACTCTGGCCTATCTGTTGGTCTGTGGGACCGGAGTAATGATTAAGAGGGGTAGTCGGGGGCATTCGTATTCCGTTGTCAGAGGTGAAATTCTTGGATTTACGGAAGACGAACATCTGCGAAAGCATTTGCCAAGGATACTTTCATTGATCAAGAACGAAAGTTGGGGGCTCGAAGACGATTAGATACCGTCGTAGTCTCAACCATAAACGATGCCGACTAGGGATTGGCAGATGTTCTTTTGATGACTCTGCCAGCACCTTATGAGAAATCAAAGTTTTTGGGTTCCGGGGGGAGTATGGTCGCAAGGCTGAAACTTAAAGGAATTGACGGAAGGGCACCACCAGGCGTGGAGCCTGCGGCTTAATTTGACTCAACACGGGGAAACTTACCAGGTCCAGACACGGGAAGGATTGACAGATTGAGAGCTCTTTCTTGATTCTGTGGGTGGTGGTGCATGGCCGTTCTTAGTTGGTGGGTTGCCTTGTCAGGTTGATTCCGGTAACGAACGAGACCTCAGCCTGCTAAATAGTCAGCATCGCACCTGCGGTGCGCCGACTTCTTAGAGGGACTATTGGCGTTTAGCCAATGGAAGTATGAGGCGATAACAGGTCTGTGATGCCCTTAGATGTTCTGGGCCGCACGCGCGCTACACTGACGCGACCAACGAGCCTATCCTTGGCCGAGAGGCCCGGGTAATCTTGTAAACCGCGTCGTGATGGGGATAGATTATTGCAATTATTAGTCTTCAACGAGGAATGCCTAGTAAGCGCGAGTCATCAGCTCGCGTTGATTACGTCCCTGCCCTTTGTACACACCGCCCGTCGCTCCTACCGATTGGGTGTGCTGGTGAAGTGTTCGGATTGAGCTTGGCTGGGGCAACCTGGCCTTGCTTGAGAAGTTCATTAAACCCTCCCACCTA

>AB511836.1 KkS0801B1

CCATGCATGTCTAAGTATAAACTGCTTATACTGTGAAACTGCGAATGGCTCATTAAATCAGTTATAGTTTATTTGATGGTACCTACTACTCGGATAACCGTAGTAATTCTAGAGCTAATACGTGCGCACAACCCGACTTCTGGAAGGGTCGTATTTATTAGATAAAAGGCCAGCCGGGCTCTGCCCGACCTGCGGTGAATCATGATAACTTCACGAATCGTATGGGCTCGTCCCGACGATGTTTCATTCAAATTTCTGCCCTATCAACTTTCGATGGTAGGATAGAGGCCTACCATGGTGGTAACGGGTGACGGAGGATTAGGGTTCGATTCCGGAGAGGGAGCCTGAGAGATGGCTACCACATCCAAGGAAGGCAGCAGGCGCGCAAATTACCCAATCCCGACACGGGGAGGTAGTGACAATAAATAACAATACCGGGCGCTTCGCGTCTGGTAATTGGAATGAGTACAATCTAAATCCCTTAACGAGGATCCATTGGAGGGCAAGTCTGGTGCCAGCAGCCGCGGTAATTCCAGCTCCAATAGCGTATATTTAAGTTGTTGCAGTTAAAAAGCTCGTAGTTGGATTTCGGGTGGGGTGGTGCGGTCCGCCTCTGGTGTGCACTGCTCTGCTCCACCTTCCTGCCGGGGACGGGCTCCTGGGCTTCACTGTCTGGGACTCGGAGTCGGCGAGGTTACTTTGAGTAAATTAGAGTGTTCAAAGCAGGCCTACGCTCTGAATACATTAGCATGGAATAACACGATAGGACTCTGGCCTATCTGTTGGTCTGTGGGACCGGAGTAATGATTAAGAGGGGTAGTCGGGGGCATTCGTATTCCGTTGTCAGAGGTGAAATTCTTGGATTTACGGAAGACGAACATCTGCGAAAGCATTTGCCAAGGATACTTTCATTGATCAAGAACGAAAGTTGGGGGCTCGAAGACGATTAGATACCGTCGTAGTCTCAACCATAAACGATGCCGACTAGGGATTGGCAGATGTTCTTTTGATGACTCTGCCAGCACCTTATGAGAAATCAAAGTTTTTGGGTTCCGGGGGGAGTATGGTCGCAAGGCTGAAACTTAAAGGAATTGACGGAAGGGCACCACCAGGCGTGGAGCCTGCGGCTTAATTTGACTCAACACGGGGAAACTTACCAGGTCCAGACACGGGAAGGATTGACAGATTGAGAGCTCTTTCTTGATTCTGTGGGTGGTGGTGCATGGCCGTTCTTAGTTGGTGGGTTGCCTTGTCAGGTTGATTCCGGTAACGAACGAGACCTCAGCCTGCTAAATAGTCAGCATCGCACCTGCGGTGCGCCGACTTCTTAGAGGGACTATTGGCGTTTAGCCAATGGAAGTATGAGGCGATAACAGGTCTGTGATGCCCTTAGATGTTCTGGGCCGCACGCGCGCTACACTGACGCGACCAACGAGCCTATCCTTGGCCGAGAGGCCCGGGTAATCTTGTAAACCGCGTCGTGATGGGGATAGATTATTGCAATTATTAGTCTTCAACGAGGAATGCCTAGTAAGCGCGAGTCATCAGCTCGCGTTGATTACGTCCCTGCCCTTTGTACACACCGCCCGTCGCTCCTACCGATTGGGTGTGCTGGTGAAGTGTTCGGATTGAGCTTGGCTGGGGCAACCTGGCCTTGCTTGAGAAGTTCATTAAACCCTCCCACCTA

>AB511837.1 KkS0801D2

CCATGCATGTCTAAGTATAAACTGCTTATACTGTGAAACTGCGAATGGCTCATTAAATCAGTTATAGTTTATTTGATGGTACCTACTACTCGGATAACCGTAGTAATTCTAGAGCTAATACGTGCGCACAACCCGACTTCTGGAAGGGTCGTATTTATTAGATAAAAGGCCAGCCGGGCTCTGCCCGACCTGCGGTGAATCATGATAACTTCACGAATCGTATGGGCTCGTCCCGACGATGTTTCATTCAAATTTCTGCCCTATCAACTTTCGATGGTAGGATAGAGGCCTACCATGGTGGTAACGGGTGACGGAGGATTAGGGTTCGATTCCGGAGAGGGAGCCTGAGAGATGGCTACCACATCCAAGGAAGGCAGCAGGCGCGCAAATTACCCAATCCCGACACGGGGAGGTAGTGACAATAAATAACAATACCGGGCGCTTCGCGTCTGGTAATTGGAATGAGTACAATCTAAATCCCTTAACGAGGATCCATTGGAGGGCAAGTCTGGTGCCAGCAGCCGCGGTAATTCCAGCTCCAATAGCGTATATTTAAGTTGTTGCAGTTAAAAAGCTCGTAGTTGGATTTCGGGTGGGGTGGTGCGGTCCGCCTCTGGTGTGCACTGCTCTGCTCCACCTTCCTGCCGGGGACGGGCTCCTGGGCTTCACTGTCTGGGACTCGGAGTCGGCGAGGTTACTTTGAGTAAATTAGAGTGTTCAAAGCAGGCCTACGCTCTGAATACATTAGCATGGAATAACACGATAGGACTCTGGCCTATCTGTTGGTCTGTGGGACCGGAGTAATGATTAAGAGGGGTAGTCGGGGGCATTCGTATTCCGTTGTCAGAGGTGAAATTCTTGGATTTACGGAAGACGAACATCTGCGAAAGCATTTGCCAAGGATACTTTCATTGATCAAGAACGAAAGTTGGGGGCTCGAAGACGATTAGATACCGTCGTAGTCTCAACCATAAACGATGCCGACTAGGGATTGGCAGATGTTCTTTTGATGACTCTGCCAGCACCTTATGAGAAATCAAAGTTTTTGGGTTCCGGGGGGAGTATGGTCGCAAGGCTGAAACTTAAAGGAATTGACGGAAGGGCACCACCAGGCGTGGAGCCTGCGGCTTAATTTGACTCAACACGGGGAAACTTACCAGGTCCAGACACGGGAAGGATTGACAGATTGAGAGCTCTTTCTTGATTCTGTGGGTGGTGGTGCATGGCCGTTCTTAGTTGGTGGGTTGCCTTGTCAGGTTGATTCCGGTAACGAACGAGACCTCAGCCTGCTAAATAGTCAGCATCGCACCTGCGGTGCGCCGACTTCTTAGAGGGACTATTGGCGTTTAGCCAATGGAAGTATGAGGCGATAACAGGTCTGTGATGCCCTTAGATGTTCTGGGCCGCACGCGCGCTACACTGACGCGACCAACGAGCCTATCCTTGGCCGAGAGGCCCGGGTAATCTTGTAAACCGCGTCGTGATGGGGATAGATTATTGCAATTATTAGTCTTCAACGAGGAATGCCTAGTAAGCGCGAGTCATCAGCTCGCGTTGATTACGTCCCTGCCCTTTGTACACACCGCCCGTCGCTCCTACCGATTGGGTGTGCTGGTGAAGTGTTCGGATTGAGCTTGGCTGGGGCAACCTGGCCTTGCTTGAGAAGTTCATTAAACCCTCCCACCTA

>KX781335.1 P4

CGAATGGCTCATTAAATCAGTTATAGTTTATTTGATGGTACCTACTACTCGGATAACCGTAGTAATTCTAGAGCTAATACGTGCGCACAACCCGACTTCTGGAAGGGTCGTATTTATTAGATAAAAGGCCAGCCGGGCTCTGCCCGACCTGCGGTGAATCATGATAACTTCACGAATCGTATGGGCTCGTCCCGACGATGTTTCATTCAAATTTCTGCCCTATCAACTTTCGATGGTAGGATAGAGGCCTACCATGGTGGTAACGGGTGACGGAGGATTAGGGTTCGATTCCGGAGAGGGAGCCTGAGAGATGGCTACCACATCCAAGGAAGGCAGCAGGCGCGCAAATTACCCAATCCCGACACGGGGAGGTAGTGACAATAAATAACAATACCGGGCGCTTCGCGTCTGGTAATTGGAATGAGTACAATCTAAATCCCTTAACGAGGATCCATTGGAGGGCAAGTCTGGTGCCAGCAGCCGCGGTAATTCCAGCTCCAATAGCGTATATTTAAGTTGTTGCAGTTAAAAAGCTCGTAGTTGGATTTCGGGTGGGGTGGTGCGGTCCGCCTCTGGTGTGCACTGCTCTGCTCCACCTTCCTGCCGGGGACGGGCTCCTGGGCTTCACTGTCTGGGACTCGGAGTCGGCGAGGTTACTTTGAGTAAATTAGAGTGTTCAAAGCAGGCCTACGCTCTGAATACATTAGCATGGAATAACACGATAGGACTCTGGCCTATCTGTTGGTCTGTGGGACCGGAGTAATGATTAAGAGGGGTAGTCGGGGGCATTCGTATTCCGTTGTCAGAGGTGAAATTCTTGGATTTACGGAAGACGAACATCTGCGAAAGCATTTGCCAAGGATACTTTCATTGATCAAGAACGAAAGTTGGGGGCTCGAAGACGATTAGATACCGTCGTAGTCTCAACCATAAACGATGCCGACTAGGGATTGGCAGATGTTCTTTTGATGACTCTGCCAGCACCTTATGAGAAATCAAAGTTTTTGGGTTCCGGGGGGAGTATGGTCGCAAGGCTGAAACTTAAAGGAATTGACGGAAGGGCACCACCAGGCGTGGAGCCTGCGGCTTAATTTGACTCAACACGGGGAAACTTACCAGGTCCAGACACGGGAAGGATTGACAGATTGAGAGCTCTTTCTTGATTCTGTGGGTGGTGGTGCATGGCCGTTCTTAGTTGGTGGGTTGCCTTGTCAGGTTGATTCCGGTAACGAACGAGACCTCAGCCTGCTAAATAGTCAGCATCGCACCTGCGGTGCGCCGACTTCTTAGAGGGACTATTGGCGTTTAGCCAATGGAAGTATGAGGCGATAACAGGTCTGTGATGCCCTTAGATGTTCTGGGCCGCACGCGCGCTACACTGACGCGACCAACGAGCCTATCCTTGGCCGAGAGGCCCGGGTAATCTTGTAAACCGCGTCGTGATGGGGATAGATTATTGCAATTATTAGTCTTCAACGAGGAATGCCTAGTAAGCGCGAGTCATCAGCTCGCGTTGATTACGTCCCTGCCCTTTGTACACACCGCCCGTCGCTCCTACCGATTGGGTGTGCTGGTGAAGTGTTCGGATTGAGCTTGGCTGGGGCAACCTGGCCTTGCTTGAGAAGTTCATTAAACCCTCCCACCTAGAGGAAGGAGAAGTCGTAACAAGGTTTCCGTAGGTGAACCTGCGGA

>KX781333.1 P6

GAGCTAATACGTGCGCACAACCCGACTTCTGGAAGGGTCGTATTTATTAGATAAAAGGCCAGCCGGGCTCTGCCCGACCTGCGGTGAATCATGATAACTTCACGAATCGTATGGGCTCGTCCCGACGATGTTTCATTCAAATTTCTGCCCTATCAACTTTCGATGGTAGGATAGAGGCCTACCATGGTGGTAACGGGTGACGGAGGATTAGGGTTCGATTCCGGAGAGGGAGCCTGAGAGATGGCTACCACATCCAAGGAAGGCAGCAGGCGCGCAAATTACCCAATCCCGACACGGGGAGGTAGTGACAATAAATAACAATACCGGGCGCTTCGCGTCTGGTAATTGGAATGAGTACAATCTAAATCCCTTAACGAGGATCCATTGGAGGGCAAGTCTGGTGCCAGCAGCCGCGGTAATTCCAGCTCCAATAGCGTATATTTAAGTTGTTGCAGTTAAAAAGCTCGTAGTTGGATTTCGGGTGGGGTGGTGCGGTCCGCCTCTGGTGTGCACTGCTCTGCTCCACCTTCCTGCCGGGGACGGGCTCCTGGGCTTCACTGTCTGGGACTCGGAGTCGGCGAGGTTACTTTGAGTAAATTAGAGTGTTCAAAGCAGGCCTACGCTCTGAATACATTAGCATGGAATAACACGATAGGACTCTGGCCTATCTGTTGGTCTGTGGGACCGGAGTAATGATTAAGAGGGGTAGTCGGGGGCATTCGTATTCCGTTGTCAGAGGTGAAATTCTTGGATTTACGGAAGACGAACATCTGCGAAAGCATTTGCCAAGGATACTTTCATTGATCAAGAACGAAAGTTGGGGGCTCGAAGACGATTAGATACCGTCGTAGTCTCAACCATAAACGATGCCGACTAGGGATTGGCAGATGTTCTTTTGATGACTCTGCCAGCACCTTATGAGAAATCAAAGTTTTTGGGTTCCGGGGGGAGTATGGTCGCAAGGCTGAAACTTAAAGGAATTGACGGAAGGGCACCACCAGGCGTGGAGCCTGCGGCTTAATTTGACTCAACACGGGGAAACTTACCAGGTCCAGACACGGGAAGGATTGACAGATTGAGAGCTCTTTCTTGATTCTGTGGGTGGTGGTGCATGGCCGTTCTTAGTTGGTGGGTTGCCTTGTCAGGTTGATTCCGGTAACGAACGAGACCTCAGCCTGCTAAATAGTCAGCATCGCACCTGCGGTGCGCCGACTTCTTAGAGGGACTATTGGCGTTTAGCCAATGGAAGTATGAGGCGATAACAGGTCTGTGATGCCCTTAGATGTTCTGGGCCGCACGCGCGCTACACTGACGCGACCAACGAGCCTATCCTTGGCCGAGAGGCCCGGGTAATCTTGTAAACCGCGTCGTGATGGGGATAGATTATTGCAATTATTAGTCTTCAACGAGGAATGCCTAGTAAGCGCGAGTCATCAGCTCGCGTTGACTACGTCCCTGCCCTTTGTACACACCGCCCGTCGCTCCTACCGATTGGGTGTGCTGGTGAAGTGTTCGGATTGAGCTTGGCTGGGGCAACCTGGCCTTGCTTGAGAAGTTCATTAAACCCT

>KX781332.1 P7

ATATTTAAGTTGTTGCAGTTAAAAAGCTCGTAGTTGGATTTCGGGTGGGGTGGTGCGGTCCGCCTCTGGTGTGCACTGCTCTGCTCCACCTTCCTGCCGGGGACGGGCTCCTGGGCTTCACTGTCTGGGACTCGGAGTCGGCGAGGTTACTTTGAGTAAATTAGAGTGTTCAAAGCAGGCCTACGCTCTGAATACATTAGCATGGAATAACACGATAGGACTCTGGCCTATCTGTTGGTCTGTGGGACCGGAGTAATGATTAAGAGGGGTAGTCGGGGGCATTCGTATTCCGTTGTCAGAGGTGAAATTCTTGGATTTACGGAAGACGAACATCTGCGAAAGCATTTGCCAAGGATACTTTCATTGATCAAGAACGAAAGTTGGGGGCTCGAAGACGATTAGATACCGTCGTAGTCTCAACCATAAACGATGCCGACTAGGGATTGGCAGATGTTCTTTTGATGACTCTGCCAGCACCTTATGAGAAATCAAAGTTTTTGGGTTCCGGGGGGAGTATGGTCGCAAGGCTGAAACTTAAAGGAATTGACGGAAGGGCACCACCAGGCGTGGAGCCTGCGGCTTAATTTGACTCAACACGGGGAAACTTACCAGGTCCAGACACGGGAAGGATTGACAGATTGAGAGCTCTTTCTTGATTCTGTGGGTGGTGGTGCATGGCCGTTCTTAGTTGGTGGGTTGCCTTGTCAGGTTGATTCCGGTAACGAACGAGACCTCAGCCTGCTAAATAGTCAGCATCGCACCTGCGGTGCGCCGACTTCTTAGAGGGACTATTGGCGTTTAGCCAATGGAAGTATGAGGCGATAACAGGTCTGTGATGCCCTTAGATGTTCTGGGCCGCACGCGCGCTACACTGACGCGACCAACGAGCCTATCCTTGGCCGAGAGGCCCGGGTAATCTTGTAAACCGCGTCGTGATGGGGATAGATTATTGCAATTATTAGTCTTCAACGAGGAATGCCTAGTAAGCGCGAGTCATCAGCTCGCGTTGATTACGTCCCTGCCCTTTGTACACACCGCCCGTCGCTCCTACCGATTGGGTGTGCTGGTGAAGTGTTCGGATTGAGCTTGGCTGGGGCAACCTGGCCTTGCTTGAGAAGTTCATTAAACCCTCCCACCTA

>KX781331.1 P8

AAGATTAAGCCATGCATGTCTAAGTATAAACTGCTTTATACTGTGAAACTGCGAATGGCTCATTAAATCAGTTATAGTTTATTTGATGGTACCTACTACTCGGATAACCGTAGTAATTCTAGAGCTAATACGTGCGTAAATCCCGACTTCTGGAAGGGACGTATTTATTAGATAAAAGGCCAGCCGGGCTTTGCCCGACCTGCGGTGAATCATGATAACTTCACGAATCGCATGGCCTTGCGCCGGCGATGTTTCATTCAAATTTCTGCCCTATCAACTTTCGATGGTAGGATAGAGGCCTACCATGGTGGTAACGGGTGACGGAGGATTAGGGTTCGATTCCGGAGAGGGAGCCTGAGAGATGGCTACCACATCCAAGGAAGGCAGCAGGCGCGCAAATTACCCAATCCCAACACGGGGAGGTAGTGACAATAAATAACAATACCGGGCATTTCATGTCTGGTAATTGGAATGAGTACAATCTAAATCCCTTAACGAGGATCCATTGGAGGGCAAGTCTGGTGCCAGCAGCCGCGGTAATTCCAGCTCCAATAGCGTATATTTAAGTTGTTGCAGTTAAAAAGCTCGTAGTTGGATTTCGGGTGGGTCTTAGCGGTCCGCCTCTGGTGTGTACTGCTAGGGCCTATCTTTCTGCCGGGGACGGGCTCCTGGGTTTAATCGCCTGGGACTCGGAGTCGGCGAGGTTACTTTGAGTAAATTAGAGTGTTCAAAGCAAGCCTACGCTCTGAATACATTAGCATGGAATAACACGATAGGACTCTGGCCTATCTTGTTGGTCTGTAGGACCGGAGTAATGATTAAGAGGGACAGTCGGGGGCATTCGTATTTCATTGTCAGAGGTGAAATTCTTGGATTTATGAAAGACGAACTTCTGCGAAAGCATTTGCCAAGGATGTTTTCATTAATCAAGAACGAAAGTTGGGGGCTCGAAGACGATTAGATACCGTCGTAGTCTCAACCATAAACGATGCCGACTAGGGATTGGCAGATGTTTCATTGATGACTCTGCCAGCACCTTATGAGAAATCAAAGTTTTTGGGTTCCGGGGGGAGTATGGTCGCAAGGCTGAAACTTAAAGGAATTGACGGAAGGGCACCACCAGGCGTGGAGCCTGCGGCTTAATTTGACTCAACACGGGAAAACTTACCAGGTCCAGACACGGGAAGGATTGACAGATTGAGAGCTCTTTCTTGATTCTGTGGGTGGTGGTGCATGGCCGTTCTTAGTTGGTGGGTTGCCTTGTCAGGTTGATTCCGGTAACGAACGAGACCTCAGCCTGCTAAATAGTCACGACTGCTTTTTGCAGTTGGCCGACTTCTTAGAGGGACTATTGTCGTGTAGGCAATGGAAGTATGAGGCAATAACAGGTCTGTGATGCCCTTAGATGTTCTGGGCCGCACGCGCGCTACACTGACGCATTCAACGAGCCTATCCTTGGCCGAGAGGCCCGGGTAATCTTTGAAACTGCGTCGTGATGGGGATAGATTATTGCAATTATTAGTCTTCAACGAGGAATGCCTAGTAAGCGCGAGTCATCAGCTCGCGTTGATTACGTCCCTGCCCTTTGTA

>KX781330.1 P10

TTATTAGATAAAAGGCCAGCCGGGCTCTGCCCGACCTGCGGTGAATCATGATAACTTCACGAATCGTATGGGCTCGTCCCGACGATGTTTCATTCAAATTTCTGCCCTATCAACTTTCGATGGTAGGATAGAGGCCTACCATGGTGGTAACGGGTGACGGAGGATTAGGGTTCGATTCCGGAGAGGGAGCCTGAGAGATGGCTACCACATCCAAGGAAGGCAGCAGGCGCGCAAATTACCCAATCCCGACACGGGGAGGTAGTGACAATAAATAACAATACCGGGCGCTTCGCGTCTGGTAATTGGAATGAGTACAATCTAAATCCCTTAACGAGGATCCATTGGAGGGCAAGTCTGGTGCCAGCAGCCGCGGTAATTCCAGCTCCAATAGCGTATATTTAAGTTGTTGCAGTTAAAAAGCTCGTAGTTGGATTTCGGGTGGGGTGGTGCGGTCCGCCTCTGGTGTGCACTGCTCTGCTCCACCTTCCTGCCGGGGACGGGCTCCTGGGCTTCACTGTCTGGGACTCGGAGTCGGCGAGGTTACTTTGAGTAAATTAGAGTGTTCAAAGCAGGCCTACGCTCTGAATACATTAGCATGGAATAACACGATAGGACTCTGGCCTATCTGTTGGTCTGTGGGACCGGAGTAATGATTAAGAGGGGTAGTCGGGGGCATTCGTATTCCGTTGTCAGAGGTGAAATTCTTGGATTTACGGAAGACGAACATCTGCGAAAGCATTTGCCAAGGATACTTTCATTGATCAAGAACGAAAGTTGGGGGCTCGAAGACGATTAGATACCGTCGTAGTCTCAACCATAAACGATGCCGACTAGGGATTGGCAGATGTTCTTTTGATGACTCTGCCAGCACCTTATGAGAAATCAAAGTTTTTGGGTTCCGGGGGGAGTATGGTCGCAAGGCTGAAACTTAAAGGAATTGACGGAAGGGCACCACCAGGCGTGGAGCCTGCGGCTTAATTTGACTCAACACGGGGAAACTTACCAGGTCCAGACACGGGAAGGATTGACAGATTGAGAGCTCTTTCTTGATTCTGTGGGTGGTGGTGCATGGCCGTTCTTAGTTGGTGGGTTGCCTTGTCAGGTTGATTCCGGTAACGAACGAGACCTCAGCCTGCTAAATAGTCAGCATCGCACCTGCGGTGCGCCGACTTCTTAGAGGGACTATTGGCGTTTAGCCAATGGAAGTATGAGGCGATAACAGGTCTGTGATGCCCTTAGATGTTCTGGGCCGCACGCGCGCTACACTGACGCGACCAACGAGCCTATCCTTGGCCGAGAGGCCCGGGTAATCTTGTAAACCGCGTCGTGATGGGGATAGATTATTGCAATTATTAGTCTTCAACGAGGAATGCCTAGTAAGCGCGAGTCATCAGCTCGCGTTGATTACGTCCCTGCCCTTTGTACACACCGCCCGTCGCTCCTACCGATTGGGTGTGCTGGTGAAGTGTTCGGATTGAGCTTGGCTGGGGCAACCTGGCCTTGCTTGAGAAGTTCATTAAACCCTCCCACCTAGAG

>KX781328.1 P12

GCCCGACCTGCGGTGAATCATGATAACTTCACGAATCGTATGGGCTCGTCCCGACGATGTTTCATTCAAATTTCTGCCCTATCAACTTTCGATGGTAGGATAGAGGCCTACCATGGTGGTAACGGGTGACGGAGGATTAGGGTTCGATTCCGGAGAGGGAGCCTGAGAGATGGCTACCACATCCAAGGAAGGCAGCAGGCGCGCAAATTACCCAATCCCGACACGGGGAGGTAGTGACAATAAATAACAATACCGGGCGCTTCGCGTCTGGTAATTGGAATGAGTACAATCTAAATCCCTTAACGAGGATCCATTGGAGGGCAAGTCTGGTGCCAGCAGCCGCGGTAATTCCAGCTCCAATAGCGTATATTTAAGTTGTTGCAGTTAAAAAGCTCGTAGTTGGATTTCGGGTGGGGTGGTGCGGTCCGCCTCTGGTGTGCACTGCTCTGCTCCACCTTCCTGCCGGGGACGGGCTCCTGGGCTTCACTGTCTGGGACTCGGAGTCGGCGAGGTTACTTTGAGTAAATTAGAGTGTTCAAAGCAGGCCTACGCTCTGAATACATTAGCATGGAATAACACGATAGGACTCTGGCCTATCTGTTGGTCTGTGGGACCGGAGTAATGATTAAGAGGGGTAGTCGGGGGCATTCGTATTCCGTTGTCAGAGGTGAAATTCTTGGATTTACGGAAGACGAACATCTGCGAAAGCATTTGCCAAGGATACTTTCATTGATCAAGAACGAAAGTTGGGGGCTCGAAGACGATTAGATACCGTCGTAGTCTCAACCATAAACGATGCCGACTAGGGATTGGCAGATGTTCTTTTGATGACTCTGCCAGCACCTTATGAGAAATCAAAGTTTTTGGGTTCCGGGGGGAGTATGGTCGCAAGGCTGAAACTTAAAGGAATTGACGGAAGGGCACCACCAGGCGTGGAGCCTGCGGCTTAATTTGACTCAACACGGGGAAACTTACCAGGTCCAGACACGGGAAGGATTGACAGATTGAGAGCTCTTTCTTGATTCTGTGGGTGGTGGTGCATGGCCGTTCTTAGTTGGTGGGTTGCCTTGTCAGGTTGATTCCGGTAACGAACGAGACCTCAGCCTGCTAAATAGTCAGCATCGCACCTGCGGTGCGCCGACTTCTTAGAGGGACTATTGGCGTTTAGCCAATGGAAGTATGAGGCGATAACAGGTCTGTGATGCCCTTAGATGTTCTGGGCCGCACGCGCGCTACACTGACGCGACCAACGAGCCTATCCTTGGCCGAGAGGCCCGGGTAATCTTGTAAACCGCGTCGTGATGGGGATAGATTATTGCAATTATTAGTCTTCAACGAGGAATGCCTAGTAAGCGCGAGTCATCAGCTCGCG

>KX781329.1 P11

TTTCTGCCCTATCAACTTTCGATGGTAGGATAGAGGCCTACCATGGTGGTAACGGGTGACGGAGGATTAGGGTTCGATTCCGGAGAGGGAGCCTGAGAGATGGCTACCACATCCAAGGAAGGCAGCAGGCGCGCAAATTACCCAATCCCGACACGGGGAGGTAGTGACAATAAATAACAATACCGGGCGCTTCGCGTCTGGTAATTGGAATGAGTACAATCTAAATCCCTTAACGAGGATCCATTGGAGGGCAAGTCTGGTGCCAGCAGCCGCGGTAATTCCAGCTCCAATAGCGTATATTTAAGTTGTTGCAGTTAAAAAGCTCGTAGTTGGATTTCGGGTGGGGTGGTGCGGTCCGCCTCTGGTGTGCACTGCTCTGCTCCACCTTCCTGCCGGGGACGGGCTCCTGGGCTTCACTGTCTGGGACTCGGAGTCGGCGAGGTTACTTTGAGTAAATTAGAGTGTTCAAAGCAGGCCTACGCTCTGAATACATTAGCATGGAATAACACGATAGGACTCTGGCCTATCTGTTGGTCTGTGGGACCGGAGTAATGATTAAGAGGGGTAGTCGGGGGCATTCGTATTCCGTTGTCAGAGGTGAAATTCTTGGATTTACGGAAGACGAACATCTGCGAAAGCATTTGCCAAGGATACTTTCATTGATCAAGAACGAAAGTTGGGGGCTCGAAGACGATTAGATACCGTCGTAGTCTCAACCATAAACGATGCCGACTAGGGATTGGCAGATGTTCTTTTGATGACTCTGCCAGCACCTTATGAGAAATCAAAGTTTTTGGGTTCCGGGGGGAGTATGGTCGCAAGGCTGAAACTTAAAGGAATTGACGGAAGGGCACCACCAGGCGTGGAGCCTGCGGCTTAATTTGACTCAACACGGGGAAACTTACCAGGTCCAGACACGGGAAGGATTGACAGATTGAGAGCTCTTTCTTGATTCTGTGGGTGGTGGTGCATGGCCGTTCTTAGTTGGTGGGTTGCCTTGTCAGGTTGATTCCGGTAACGAACGAGACCTCAGCCTGCTAAATAGTCAGCATCGCACCTGCGGTGCGCCGACTTCTTAGAGGGACTATTGGCGTTTAGCCAATGGAAGTATGAGGCGATAACAGGTCTGTGATGCCCTTAGATGTTCTGGGCCGCACGCGCGCTACACTGACGCGACCAACGAGCCTATCCTTGGCCGAGAGGCCCGGGTAATCTTGTAAACCGCGTCGTGATGGGGATAGATTATTGCAATTATTAGTCTTCAACGAGGAATGCCTAGTAAGCGCGAGTCATCAGCTCGCGTTGATTACGTCCCTGCCCTTTGTACACACCGCCCGTCGCTCCTACCGATTGGGTGTGCTGGTGAAGTGTTCGGATTGAGCTTGGCTGGGGCAACCTGGCCTTGCTTGAGAAGTTCATTAAACCCTCCCACCTAGAGGAA

>KX781327.1 P13

TGCCCGACCTGCGGTGAATCATGATAACTTCACGAATCGTATGGGCTCGTCCCGACGATGTTTCATTCAAATTTCTGCCCTATCAACTTTCGATGGTAGGATAGAGGCCTACCATGGTGGTAACGGGTGACGGAGGATTAGGGTTCGATTCCGGAGAGGGAGCCTGAGAGATGGCTACCACATCCAAGGAAGGCAGCAGGCGCGCAAATTACCCAATCCCGACACGGGGAGGTAGTGACAATAAATAACAATACCGGGCGCTTCGCGTCTGGTAATTGGAATGAGTACAATCTAAATCCCTTAACGAGGATCCATTGGAGGGCAAGTCTGGTGCCAGCAGCCGCGGTAATTCCAGCTCCAATAGCGTATATTTAAGTTGTTGCAGTTAAAAAGCTCGTAGTTGGATTTCGGGTGGGGTGGTGCGGTCCGCCTCTGGTGTGCACTGCTCTGCTCCACCTTCCTGCCGGGGACGGGCTCCTGGGCTTCACTGTCTGGGACTCGGAGTCGGCGAGGTTACTTTGAGTAAATTAGAGTGTTCAAAGCAGGCCTACGCTCTGAATACATTAGCATGGAATAACACGATAGGACTCTGGCCTATCTGTTGGTCTGTGGGACCGGAGTAATGATTAAGAGGGGTAGTCGGGGGCATTCGTATTCCGTTGTCAGAGGTGAAATTCTTGGATTTACGGAAGACGAACATTACCGTCGTAGTCTCAACCATAAACGATGCCGACTAGGGATTGGCAGATGTTCTTTTGATGACTCTGCCAGCACCTTATGAGAAATCAAAGTTTTTGGGTTCCGGGGGGAGTATGGTCGCAAGGCTGAAACTTAAAGGAATTGACGGAAGGGCACCACCAGGCGTGGAGCCTGCGGCTTAATTTGACTCAACACGGGGAAACTTACCAGGTCCAGACACGGGAAGGATTGACAGATTGAGAGCTCTTTCTTGATTCTGTGGGTGGTGGTGCATGGCCGTTCTTAGTTGGTGGGTTGCCTTGTCAGGTTGATTCCGGTAACGAACGAGACCTCAGCCTGCTAAATAGTCAGCATCGCACCTGCGGTGCGCCGACTTCTTAGAGGGACTATTGGCGTTTAGCCAATGGAAGTATGAGGCGATAACAGGTCTGTGATGCCCTTAGATGTTCTGGGCCGCACGCGCGCTACACTGACGCGACCAACGAGCCTATCCTTGGCCGAGAGGCCCGGGTAATCTTGTAAACCGCGTCGTGATGGGGATAGATTATTGCAATTATTAGTCTTCAACGAGGAATGCCTAGTAAGCGCGAGTCATCAGCTCGCGTTGACTACGTCCCTGCCCTTTGTACACACCGCCCGTCGCTCCTACCGATTGGGTGTGCTGGTGAAGTGTTCGGATTGAGCTTGGCTGGGGCAACCTGGCCTTGCTTGAGAAGTTCATTAAACCCT

>KX781326.1 P14

AATGGCTCATTATATCAGTTATAGTTTATTTGATGGTACCTACTACTCGGATAACCGTAGTAATTCTAGAGCTAATACGTGCGCACAACCCGACTTCTGGAAGGGTCGTATTTATTAGATAAAAGGCCAGCCGGGCTCTGCCCGACCTGCGGTGAATCATGATAACTTCACGAATCGTATGGGCTCGTCCCGACGATGTTTCATTCAAATTTCTGCCCTATCAACTTTCGATGGTAGGATAGAGGCCTACCATGGTGGTAACGGGTGACGGAGGATTAGGGTTCGATTCCGGAGAGGGAGCCTGAGAGATGGCTACCACATCCAAGGAAGGCAGCAGGCGCGCAAATTACCCAATCCCGACACGGGGAGGTAGTGACAATAAATAACAATACCGGGCGCTTCGCGTCTGGTAATTGGAATGAGTACAATCTAAATCCCTTAACGAGGATCCATTGGAGGGCAAGTCTGGTGCCAGCAGCCGCGGTAATTCCAGCTCCAATAGCGTATATTTAAGTTGTTGCAGTTAAAAAGCTCGTAGTTGGATTTCGGGTGGGGTGGTGCGGTCCGCCTCTGGTGTGCACTGCTCTGCTCCACCTTCCTGCCGGGGACGGGCTCCTGGGCTTCACTGTCTGGGACTCGGAGTCGGCGAGGTTACTTTGAGTAAATTAGAGTGTTCAAAGCAGGCCTACGCTCTGAATACATTAGCATGGAATAACACGATAGGACTCTGGCCTATCTGTTGGTCTGTGGGACCGGAGTAATGATTAAGAGGGGTAGTCGGGGGCATTCGTATTCCGTTGTCAGAGGTGAAATTCTTGGATTTACGGAAGACGAACATCTGCGAAAGCATTTGCCAAGGATACTTTCATTGATCAAGAACGAAAGTTGGGGGCTCGAAGACGATTAGATACCGTCGTAGTCTCAACCATAAACGATGCCGACTAGGGATTGGCAGATGTTCTTTTGATGACTCTGCCAGCACCTTATGAGAAATCAAAGTTTTTGGGTTCCGGGGGGAGTATGGTCGCAAGGCTGAAACTTAAAGGAATTGACGGAAGGGCACCACCAGGCGTGGAGCCTGCGGCTTAATTTGACTCAACACGGGGAAACTTACCAGGTCCAGACACGGGAAGGATTGACAGATTGAGAGCTCTTTCTTGATTCTGTGGGTGGTGGTGCATGGCCGTTCTTAGTTGGTGGGTTGCCTTGTCAGGTTGATTCCGGTAACGAACGAGACCTCAGCCTGCTAAATAGTCAGCATCGCACCTGCGGTGCGCCGACTTCTTAGAGGGACTATTGGCGTTTAGCCAATGGAAGTATGAGGCGATAACAGGTCTGTGATGCCCTTAGATGTTCTGGGCCGCACGCGCGCTACACTGACGCGACCAACGAGCCTATCCTTGGCCGAGAGGCCCGGGTAATCTTGTAAACCGCGTCGTGATGGGGATAGATTATTGCAATTATTAGTCTTCAACGAGGAATGCCTAGTAAGCGCGAGTCATCAGCTCGCGTTGATTACGTCCCTGCCCTTTGTACACACCGCCCGTCGCTCCTACCGATTGGGTGTGCTGGTGAAGTGTTCGGATTGAGCTTGGCTGGGGCAACCTGGCCTTGCTTGAGAAGTTCATTAAACCCTCCCACCTA>KX781325.1 P15CGAATGGCTCATTAAATCAGTTATAGTTTATTTGATGGTACCTACTACTCGGATAACCGTAGTAATTCTAGAGCTAATACGTGCGCACAACCCGACTTCTGGAAGGGTCGTATTTATTAGATAAAAGGCCAGCCGGGCTCTGCCCGACCTGCGGTGAATCATGATAACTTCACGAATCGTATGGGCTCGTCCCGACGATGTTTCATTCAAATTTCTGCCCTATCAACTTTCGATGGTAGGATAGAGGCCTACCATGGTGGTAACGGGTGACGGAGGATTAGGGTTCGATTCCGGAGAGGGAGCCTGAGAGATGGCTACCACATCCAAGGAAGGCAGCAGGCGCGCAAATTACCCAATCCCGACACGGGGAGGTAGTGACAATAAATAACAATACCGGGCGCTTCGCGTCTGGTAATTGGAATGAGTACAATCTAAATCCCTTAACGAGGATCCATTGGAGGGCAAGTCTGGTGCCAGCAGCCGCGGTAATTCCAGCTCCAATAGCGTATATTTAAGTTGTTGCAGTTAAAAAGCTCGTAGTTGGATTTCGGGTGGGGTGGTGCGGTCCGCCTCTGGTGTGCACTGCTCTGCTCCACCTTCCTGCCGGGGACGGGCTCCTGGGCTTCACTGTCTGGGACTCGGAGTCGGCGAGGTTACTTTGAGTAAATTAGAGTGTTCAAAGCAGGCCTACGCTCTGAATACATTAGCATGGAATAACACGATAGGACTCTGGCCTATCTGTTGGTCTGTGGGACCGGAGTAATGATTAAGAGGGGTAGTCGGGGGCATTCGTATTCCGTTGTCAGAGGTGAAATTCTTGGATTTACGGAAGACGAACATCTGCGAAAGCATTTGCCAAGGATACTTTCATTGATCAAGAACGAAAGTTGGGGGCTCGAAGACGATTAGATACCGTCGTAGTCTCAACCATAAACGATGCCGACTAGGGATTGGCAGATGTTCTTTTGATGACTCTGCCAGCACCTTATGAGAAATCAAAGTTTTTGGGTTCCGGGGGGAGTATGGTCGCAAGGCTGAAACTTAAAGGAATTGACGGAAGGGCACCACCAGGCGTGGAGCCTGCGGCTTAATTTGACTCAACACGGGGAAACTTACCAGGTCCAGACACGGGAAGGATTGACAGATTGAGAGCTCTTTCTTGATTCTGTGGGTGGTGGTGCATGGCCGTTCTTAGTTGGTGGGTTGCCTTGTCAGGTTGATTCCGGTAACGAACGAGACCTCAGCCTGCTAAATAGTCAGCATCGCACCTGCGGTGCGCCGACTTCTTAGAGGGACTATTGGCGTTTAGCCAATGGAAGTATGAGGCGATAACAGGTCTGTGATGCCCTTAGATGTTCTGGGCCGCACGCGCGCTACACTGACGCGACCAACGAGCCTATCCTTGGCCGAGAGGCCCGGGTAATCTTGTAAACCGCGTCGTGATGGGGATAGATTATTGCAATTATTAGTCTTCAACGAGGAATGCCTAGTAAGCGCGAGTCATCAGCTCGCGTTGATTACGTCCCTGCCCTTTGTACACACCGCCCGTCGCTCCTACCGATTGGGTGTGCTGGTGAAGTGTTCGGATTGAGCTTGGCTGGGGCAACCTGGCCTTGCTTGAGAAGTTCATTAAACCCTCCCACCTAGAGGAAGGAGAAGTCGTAACAAGGTTTCCGTAG

>KX781322.1 P18

CCGACCTGCGGTGAATCATGATAACTTCACGAATCGTATGGGCTCGTCCCGACGATGTTTCATTCAAATTTCTGCCCTATCAACTTTCGATGGTAGGATAGAGGCCTACCATGGTGGTAACGGGTGACGGAGGATTAGGGTTCGATTCCGGAGAGGGAGCCTGAGAGATGGCTACCACATCCAAGGAAGGCAGCAGGCGCGCAAATTACCCAATCCCGACACGGGGAGGTAGTGACAATAAATAACAATACCGGGCGCTTCGCGTCTGGTAATTGGAATGAGTACAATCTAAATCCCTTAACGAGGATCCATTGGAGGGCAAGTCTGGTGCCAGCAGCCGCGGTAATTCCAGCTCCAATAGCGTATATTTAAGTTGTTGCAGTTAAAAAGCTCGTAGTTGGATTTCGGGTGGGGTGGTGCGGTCCGCCTCTGGTGTGCACTGCTCTGCTCCACCTTCCTGCCGGGGACGGGCTCCTGGGCTTCACTGTCTGGGACTCGGAGTCGGCGAGGTTACTTTGAGTAAATTAGAGTGTTCAAAGCAGGCCTACGCTCTGAATACATTAGCATGGAATAACACGATAGGACTCTGGCCTATCTGTTGGTCTGTGGGACCGGAGTAATGATTAAGAGGGGTAGTCGGGGGCATTCGTATTCCGTTGTCAGAGGTGAAATTCTTGGATTTACGGAAGACGAACATCTGCGAAAGCATTTGCCAAGGATACTTTCATTGATCAAGAACGAAAGTTGGGGGCTCGAAGACGATTAGATACCGTCGTAGTCTCAACCATAAACGATGCCGACTAGGGATTGGCAGATGTTCTTTTGATGACTCTGCCAGCACCTTATGAGAAATCAAAGTTTTTGGGTTCCGGGGGGAGTATGGTCGCAAGGCTGAAACTTAAAGGAATTGACGGAAGGGCACCACCAGGCGTGGAGCCTGCGGCTTAATTTGACTCAACACGGGGAAACTTACCAGGTCCAGACACGGGAAGGATTGACAGATTGAGAGCTCTTTCTTGATTCTGTGGGTGGTGGTGCATGGCCGTTCTTAGTTGGTGGGTTGCCTTGTCAGGTTGATTCCGGTAACGAACGAGACCTCAGCCTGCTAAATAGTCAGCATCGCACCTGCGGTGCGCCGACTTCTTAGAGGGACTATTGGCGTTTAGCCAATGGAAGTATGAGGCGATAACAGGTCTGTGATGCCCTTAGATGTTCTGGGCCGCACGCGCGCTACACTGACGCGACCAACGAGCCTATCCTTGGCCGAGAGGCCCGGGTAATCTTGTAAACCGCGTCGTGATGGGGATAGATTATTGCAATTATTAGTCTTCAACGAGGAATGCCTAGTAAGCGCGAGTCATCAGCTCGCGTTGATTACGTCCCTGCCCTTTGTACACACCGCCCGTCGCTCCTACCGATTGGGTGTGCTGGTGAAGTGTTCGGATTGAGCTTGGCTGGGGCAACCTGGCCTTGCTTGAGAAGTTCATTAAACCCTCCCACCTA

>KX781321.1 P20

GAATGGCTCATTAAATCAGTTATAGTTTATTTGATGGTACCTACTACTCGGATAACCGTAGTAATTCTAGAGCTAATACGTGCGTAAATCCCGACTTCTGGAAGGGACGTATTTATTAGATAAAAGGCCAGCCGGGCTTTGCCCGACCTGCGGTGAATCATGATAACTTCACGAATCGCATGGCCTTGCGCCGGCGATGTTTCATTCAAATTTCTGCCCTATCAACTTTCGATGGTAGGATAGAGGCCTACCATGGTGGTAACGGGTGACGGAGGATTAGGGTTCGATTCCGGAGAGGGAGCCTGAGAGATGGCTACCACATCCAAGGAAGGCAGCAGGCGCGCAAATTACCCAATCCCAACACGGGGAGGTAGTGACAATAAATAACAATACCGGGCATTTCATGTCTGGTAATTGGAATGAGTACAATCTAAATCCCTTAACGAGGATCCATTGGAGGGCAAGTCTGGTGCCAGCAGCCGCGGTAATTCCAGCTCCAATAGCGTATATTTAAGTTGTTGCAGTTAAAAAGCTCGTAGTTGGATTTCGGGTGGGTCTTAGCGGTCCGCCTCTGGTGTGTACTGCTAGGGCCTATCTTTCTGCCGGGGACGGGCTCCTGGGTTTAATCGCCTGGGACTCGGAGTCGGCGAGGTTACTTTGAGTAAATTAGAGTGTTCAAAGCAAGCCTACGCTCTGAATACATTAGCATGGAATAACACGATAGGACTCTGGCCTATCTTGTTGGTCTGTAGGACCGGAGTAATGATTAAGAGGGACAGTCGGGGGCATTCGTATTTCATTGTCAGAGGTGAAATTCTTGGATTTATGAAAGACGAACTTCTGCGAAAGCATTTGCCAAGGATGTTTTCATTAATCAAGAACGAAAGTTGGGGGCTCGAAGACGATTAGATACCGTCGTAGTCTCAACCATAAACGATGCCGACTAGGGATTGGCAGATGTTTCATTGATGACTCTGCCAGCACCTTATGAGAAATCAAAGTTTTTGGGTTCCGGGGGGAGTATGGTCGCAAGGCTGAAACTTAAAGGAATTGACGGAAGGGCACCACCAGGCGTGGAGCCTGCGGCTTAATTTGACTCAACACGGGAAAACTTACCAGGTCCAGACACGGGAAGGATTGACAGATTGAGAGCTCTTTCTTGATTCTGTGGGTGGTGGTGCATGGCCGTTCTTAGTTGGTGGGTTGCCTTGTCAGGTTGATTCCGGTAACGAACGAGACCTCAGCCTGCTAAATAGTCACGACTGCTTTTTGCAGTTGGCCGACTTCTTAGAGGGACTATTGTCGTGTAGGCAATGGAAGTATGAGGCAATAACAGGTCTGTGATGCCCTTAGATGTTCTGGGCCGCACGCGCGCTACACTGACGCATTCAACGAGCCTATCCTTGGCCGAGAGGCCCGGGTAATCTTTGAAACTGCGTCGTGATGGGGATAGATTATTGCAATTATTAGTCTTCAACGAGGAATGCCTAGTAAGCGCGAGTCATCAGCTCGCGTTGATTACGTCCCTGCCCTTTGTACACACCGCCCGTCGCTCCTACCGATTGGGTGTGCTGGTGAAGTGTTCGGATTGGCTTCAGGTGATGGCAACA

>KR092109.1 CC-125

GTATAAACTGCTTATACTGTGAAACTGCGAATGGCTCATTAAATCAGTTATAGTTTATTTGATGGTACCTACTACTCGGATAACCGTAGTAATTCTAGAGCTAATACGTGCGCACAACCCGACTTCTGGAAGGGTCGTATTTATTAGATAAAAGGCCAGCCGGGCTCTGCCCGACCTGCGGTGAATCATGATAACTTCACGAATCGTATGGGCTCGTCCCGACGATGTTTCATTCAAATTTCTGCCCTATCAACTTTCGATGGTAGGATAGAGGCCTACCATGGTGGTAACGGGTGACGGAGGATTAGGGTTCGATTCCGGAGAGGGAGCCTGAGAGATGGCTACCACATCCAAGGAAGGCAGCAGGCGCGCAAATTACCCAATCCCGACACGGGGAGGTAGTGACAATAAATAACAATACCGGGCGCTTCGCGTCTGGTAATTGGAATGAGTACAATCTAAATCCCTTAACGAGGATCCATTGGAGGGCAAGTCTGGTGCCAGCAGCCGCGGTAATTCCAGCTCCAATAGCGTATATTTAAGTTGTTGCAGTTAAAAAGCTCGTAGTTGGATTTCGGGTGGGGTGGTGCGGTCCGCCTCTGGTGTGCACTGCTCTGCTCCACCTTCCTGCCGGGGACGGGCTCCTGGGCTTCACTGTCTGGGACTCGGAGTCGGCGAGGTTACTTTGAGTAAATTAGAGTGTTCAAAGCAGGCCTACGCTCTGAATACATTAGCATGGAATAACACGATAGGACTCTGGCCTATCTGTTGGTCTGTGGGACCGGAGTAATGATTAAGAGGGGTAGTCGGGGGCATTCGTATTCCGTTGTCAGAGGTGAAATTCTTGGATTTACGGAAGACGAACATCTGCGAAAGCATTTGCCAAGGATACTTTCATTGATCAAGAACGAAAGTTGGGGGCTCGAAGACGATTAGATACCGTCGTAGTCTCAACCATAAACGATGCCGACTAGGGATTGGCAGATGTTCTTTTGATGACTCTGCCAGCACCTTATGAGAAATCAAAGTTTTTGGGTTCCGGGGGGAGTATGGTCGCAAGGCTGAAACTTAAAGGAATTGACGGAAGGGCACCACCAGGCGTGGAGCCTGCGGCTTAATTTGACTCAACACGGGGAAACTTACCAGGTCCAGACACGGGAAGGATTGACAGATTGAGAGCTCTTTCTTGATTCTGTGGGTGGTGGTGCATGGCCGTTCTTAGTTGGTGGGTTGCCTTGTCAGGTTGATTCCGGTAACGAACGAGACCTCAGCCTGCTAAATAGTCAGCATCGCACCTGCGGTGCGCCGACTTCTTAGAGGGACTATTGGCGTTTAGCCAATGGAAGTATGAGGCGATAACAGGTCTGTGATGCCCTTAGATGTTCTGGGCCGCACGCGCGCTACACTGACGCGACCAACGAGCCTATCCTTGGCCGAGAGGCCCGGGTAATCTTGTAAACCGCGTCGTGATGGGGATAGATTATTGCAATTATTAGTCTTCAACGAGGAATGCCTAGTAAGCGCGAGTCATCAGCTCGCGTTGATTACGTCCCTGCCCTTTGTACACACCGCCCGTCGCTCCTACCGATTGGGTGTGCTGGTGAAGTGTTCGGATTGAGCTTGGCTGGGGCAACCTGGCCTTGCTTGAGAAGTTCATTAAACCCTCCCACCTAGAG

>KR904894.1 CC-849

TGAGAGATGGCTACCACATCCAAGGAAGGCAGCAGGCGCGCAAATTACCCAATCCCGACACGGGGAGGTAGTGACAATAAATAACAATACCGGGCGCTTCGCGTCTGGTAATTGGAATGAGTACAATCTAAATCCCTTAACGAGGATCCATTGGAGGGCAAGTCTGGTGCCAGCAGCCGCGGTAATTCCAGCTCCAATAGCGTATATTTAAGTTGTTGCAGTTAAAAAGCTCGTAGTTGGATTTCGGGTGGGGTGGTGCGGTCCGCCTCTGGTGTGCACTGCTCTGCTCCACCTTCCTGCCGGGGACGGGCTCCTGGGCTTCACTGTCTGGGACTCGGAGTCGGCGAGGTTACTTTGAGTAAATTAGAGTGTTCAAAGCAGGCCTACGCTCTGAATACATTAGCATGGAATAACACGATAGGACTCTGGCCTATCTGTTGGTCTGTGGGACCGGAGTAATGATTAAGAGGGGTAGTCGGGGGCATTCGTATTCCGTTGTCAGAGGTGAAATTCTTGGATTTACGGAAGACGAACATCTGCGAAAGCATTTGCCAAGGATACTTTCATTGATCAAGAACGAAAGTTGGGGGCTCGAAGACGATTAGATACCGTCGTAGTCTCAACCATAAACGATGCCGACTAGGGATTGGCAGATGTTCTTTTGATGACTCTGCCAGCACCTTATGAGAAATCAAAGTTTTTGGGTTCCGGGGGGAGTATGGTCGCAAGGCTGAAACTTAAAGGAATTGACGGAAGGGCACCACCAGGCGTGGAGCCTGCGGCTTAATTTGACTCAACACGGGGAAACTTACCAGGTCCAGACACGGGAAGGATTGACAGATTGAGAGCTCTTTCTTGATTCTGTGGGTGGTGGTGCATGGCCGTTCTTAGTTGGTGGGTTGCCTTGTCAGGTTGATTCCGGTAACAAACGAGACCTCAGCCTGCTAAATAGTCAGCATCGCACCTGCGGTGCGCCGACTTCTTAGAGGGACTATTGGCGTTTAGCCAATGGAAGTATGAGGCGATAACAGGTCTGTGATGCCCTTAGATGTTCTGGGCCGCACGCGCGCTACACTGACGCGACCAACGAGCCTATCCTTGGCCGAGAGGCCCGGGTAATCTTGTAAACCGCGTCGTGATGGGGATAGATTATTGCAATTATTAGTCTTCAACGAGGAATGCCTAGTAAGCGCGAGTCATCANCTCGCGTTGATTACNTCCCTGCCCTTTGTACACACCGCCCGTCGCTCCTACCGATTGGGTGTGCTGGTGAAGTGTTCGGATTG

>KR904893.1 CBS152280

TCGGAGAGGGAAGCATGAGAGATGGCTACCACATCCAAGGAAGGCAGCAGGCGCGCAAATTACCCAATCCCGACACGGGGAGGTAGTGACAATAAATAACAATACCGGGCGCTTCGCGTCTGGTAATTGGAATGAGTACAATCTAAATCCCTTAACGAGGATCCATTGGAGGGCAAGTCTGGTGCCAGCAGCCGCGGTAATTCCAGCTCCAATAGCGTATATTTAAGTTGTTGCAGTTAAAAAGCTCGTAGTTGGATTTCGGGTGGGGTGGTGCGGTCCGCCTCTGGTGTGCACTGCTCTGCTCCACCTTCCTGCCGGGGACGGGCTCCTGGGCTTCACTGTCTGGGACTCGGAGTCGGCGAGGTTACTTTGAGTAAATTAGAGTGTTCAAAGCAGGCCTACGCTCTGAATACATTAGCATGGAATAACACGATAGGACTCTGGCCTATCTGTTGGTCTGTGGGACCGGAGTAATGATTAAGAGGGGTAGTCGGGGGCATTCGTATTCCGTTGTCAGAGGTGAAATTCTTGGATTTACGGAAGACGAACATCTGCGAAAGCATTTGCCAAGGATACTTTCATTGATCAAGAACGAAAGTTGGGGGCTCGAAGACGATTAGATACCGTCGTAGTCTCAACCATAAACGATGCCGACTAGGGATTGGCAGATGTTCTTTTGATGACTCTGCCAGCACCTTATGAGAAATCAAAGTTTTTGGGTTCCGGGGGGAGTATGGTCGCAAGGCTGAAACTTAAAGGAATTGACGGAAGGGCACCACCAGGCGTGGAGCCTGCGGCTTAATTTGACTCAACACGGGGAAACTTACCAGGTCCAGACACGGGAAGGATTGACAGATTGAGAGCTCTTTCTTGATTCTGTGGGTGGTGGTGCATGGCCGTTCTTAGTTGGTGGGTTGCCTTGTCAGGTTGATTCCGGTAACGAACGAGACCTCAGCCTGCTAAATAGTCAGCATCGCACCTGCGGTGCGCCGACTTCTTAGAGGGACTATTGGCGTTTAGCCAATGGAAGTATGAGGCGATAACAGGTCTGTGATGCCCTTAGATGTTCTGGGCCGCACGCGCGCTACACTGACGCGACCAACGAGCCTATCCTTGGCCGAGAGGCCCGGGTAATCTTGTAAACCGCGTCGTGATGGGGATAGATTATTGCAATTATTAGTCTTCAACGAGGAATGCCTAGTAAGCGCGAGTCATCAGCTCGCGTTGATTACGTCCCTGCCCTTTGTACACACCGCCCGTCGCTCCTACCGATTGGGTGTGCTGGTGAAGTGTTCGGATTGAGCTTGGCTGGGGCAACCTGGCCTTGCTTGAGAAGTTCATTAAACCCTCCCACCTAGAGGAAGGAGAAGTCGTAACAAGGTTTCCGTAGGTGAACCTGCGGAAGGATCATT

>KF864473.1 JinCheon1

CCTGGTTGATCCTGCCAGTAGTCATATGCTTGTCTCAAAGATTAAGCCATGCATGTCTAAGTATAAACTGCTTATACTGTGAAACTGCGAATGGCTCATTAAATCAGTTATAGTTTATTTGATGGTACCTACTACTCGGATAACCGTAGTAATTCTAGAGCTAATACGTGCGCACAACCCGACTTCTGGAAGGGTCGTATTTATTAGATAAAAGGTCAGCCGGGCTCTGCCCGACCTGCGGTGAATCATGATAACTTCACGAATCGTATGGCCTCGTGCCGACGATGTTTCATTCAAATTTCTGCCCTATCAACTTTCGATGGTAGGATAGAGGCCTACCATGGTGGTAACGGGTGACGGAGGATTAGGGTTCGATTCCGGAGAGGGAGCCTGAGAGATGGCTACCACATCCAAGGAAGGCAGCAGGCGCGCAAATTACCCAATCCCGACACGGGGAGGTAGTGACAATAAATAACAATACCGGGCGCTTCGCGTCTGGTAATTGGAATGAGTACAATCTAAATCCCTTAACGAGGATCCATTGGAGGGCAAGTCTGGTGCCAGCAGCCGCGGTAATTCCAGCTCCAATAGCGTATATTTAAGTTGTTGCAGTTAAAAAGCTCGTAGTTGGATTTCGGGTGGGGTGGTGCGGTCCGCCTCTGGTGTGCACTGCTCTGCTCCACCTTCCTGCCGGGGACGGGCTCCTGGGCTTAACTGTCTGGGACTCGGAGTCGGCGAGGTTACTTTGAGTAAATTAGAGTGTTCAAAGCAGGCCTACGCTCTGAATACATTAGCATGGAATAACACGATAGGACTCTGGCCTATCTGTTGTTCTGTGGGACCGGAGTAATGATTAAGAGGGGTAGTCGGGGGCATTCGTATTCCGTTGTCAGAGGTGAAATTCTTGGATTTACGGAAGACGAACATCTGCGAAAGCATTTGCCAAGGATACTTTCATTGACCAAGAACGAAAGTTGGGGGCTCGAAGACGATTAGATACCGTCGTAGTCTCAACCATAAACGATGCCGACTAGGGATTGGCAGATGTTCCTTTGATGACTCTGCCAGCACCTTATGAGAAATCAAAGTTTTTGGGTTCCGGGGGGAGTATGGTCGCAAGGCTGAAACTTAAAGGAATTGACGGAAGGGCACCACCAGGCGTGGAGCCTGCGGCTTAATTTGACTCAACACGGGGAAACTTACCAGGTCCAGACACGGGAAGGATTGACAGATTGAGAGCTCTTTCTTGATTCTGTGGGTGGTGGTGCATGGCCGTTCTTAGTTGGTGGGTTGCCTTGTCAGGTTGATTCCGGTAACGAACGAGACCTCAGCCTGCTAAATAGTCAGCATCGCACCTGCGGTGCGCAGACTTCTTAGAGGGACTATTGGCGTTCAGCCAATGGAAGTATGAGGCGATAACAGGTCTGTGATGCCCTTAGATGTTCTGGGCCGCACGCGCGCTACACTGACGCGACCAACGAGCCTATCCTTGGCCGAGAGGCCCGGGTAATCTTGTAAACCGCGTCGTGATGGGGATAGATTATTGCAATTATTAGTCTTCAACGAGGAATGCCTAGTAAGCGCGAGTCATCAGCTCGCATTGATTACGTCCCTGCCCTTTGTACACACCGCCCGTCGCTCCTACCGATTGGGTGTGCTGGTGAAGTGTTCGGATTGAGTCTGAGTGGGGCAACCTGGTCAGATTCGAGAAGTTCATTAAACCCTCCCACCTAGAGGAAGGAGAAGTCGTAACAAGGTTTCCGTAGGTGAACCTGCAGAAGGATCA

>AB701555.1 NIES-2463

GCGAATGGCTCATTATATCAGTTATAGTTTATTTGATGGTACCTACTACTCGGATAACCGTAGTAATTCTAGAGCTAATACGTGCGCACAACCCGACTTCTGGAAGGGTCGTATTTATTAGATAAAAGGCCAGCCGGGCTCTGCCCGACCTGCGGTGAATCATGATAACTTCACGAATCGTATGGGCTCGTCCCGACGATGTTTCATTCAAATTTCTGCCCTATCAACTTTCGATGGTAGGATAGAGGCCTACCATGGTGGTAACGGGTGACGGAGGATTAGGGTTCGATTCCGGAGAGGGAGCCTGAGAGATGGCTACCACATCCAAGGAAGGCAGCAGGCGCGCAAATTACCCAATCCCGACACGGGGAGGTAGTGACAATAAATAACAATACCGGGCGCTTCGCGTCTGGTAATTGGAATGAGTACAATCTAAATCCCTTAACGAGGATCCATTGGAGGGCAAGTCTGGTGCCAGCAGCCGCGGTAATTCCAGCTCCAATAGCGTATATTTAAGTTGTTGCAGTTAAAAAGCTCGTAGTTGGATTTCGGGTGGGGTGGTGCGGTCCGCCTCTGGTGTGCACTGCTCTGCTCCACCTTCCTGCCGGGGACGGGCTCCTGGGCTTCACTGTCTGGGACTCGGAGTCGGCGAGGTTACTTTGAGTAAATTAGAGTGTTCAAAGCAGGCCTACGCTCTGAATACATTAGCATGGAATAACACGATAGGACTCTGGCCTATCTGTTGGTCTGTGGGACCGGAGTAATGATTAAGAGGGGTAGTCGGGGGCATTCGTATTCCGTTGTCAGAGGTGAAATTCTTGGATTTACGGAAGACGAACATCTGCGAAAGCATTTGCCAAGGATACTTTCATTGATCAAGAACGAAAGTTGGGGGCTCGAAGACGATTAGATACCGTCGTAGTCTCAACCATAAACGATGCCGACTAGGGATTGGCAGATGTTCTTTTGATGACTCTGCCAGCACCTTATGAGAAATCAAAGTTTTTGGGTTCCGGGGGGAGTATGGTCGCAAGGCTGAAACTTAAAGGAATTGACGGAAGGGCACCACCAGGCGTGGAGCCTGCGGCTTAATTTGACTCAACACGGGGAAACTTACCAGGTCCAGACACGGGAAGGATTGACAGATTGAGAGCTCTTTCTTGATTCTGTGGGTGGTGGTGCATGGCCGTTCTTAGTTGGTGGGTTGCCTTGTCAGGTTGATTCCGGTAACGAACGAGACCTCAGCCTGCTAAATAGTCAGCATCGCACCTGCGGTGCGCCGACTTCTTAGAGGGACTATTGGCGTTTAGCCAATGGAAGTATGAGGCGATAACAGGTCTGTGATGCCCTTAGATGTTCTGGGCCGCACGCGCGCTACACTGACGCGACCAACGAGCCTATCCTTGGCCGAGAGGCCCGGGTAATCTTGTAAACCGCGTCGTGATGGGGATAGATTATTGCAATTATTAGTCTTCAACGAGGAATGCCTAGTAAGCGCGAGTCATCAGCTCGCGTTGATTACGTCCCTGCCCTTTGTACACACCGCCCGTCGCTCCTACCGATTGGGTGTGCTGGTGAAGTGTTCGGATTGAGCTTGGCTGGGGCAACCTGGCCTTGCTTGAGAAGTTCATTAAACCCTCC

>AB701551.1 NIES-2236

AATATATCAGTTATAGTTTATTTGATGGTACCTACTACTCGGATAACCGTAGTAATTCTAGAGCTAATACGTGCGCACAACCCGACTTCTGGAAGGGTCGTATTTATTAGATAAAAGGCCAGCCGGGCTCTGCCCGACCTGCGGTGAATCATGATAACTTCACGAATCGTATGGGCTCGTCCCGACGATGTTTCATTCAAATTTCTGCCCTATCAACTTTCGATGGTAGGATAGAGGCCTACCATGGTGGTAACGGGTGACGGAGGATTAGGGTTCGATTCCGGAGAGGGAGCCTGAGAGATGGCTACCACATCCAAGGAAGGCAGCAGGCGCGCAAATTACCCAATCCCGACACGGGGAGGTAGTGACAATAAATAACAATACCGGGCGCTTCGCGTCTGGTAATTGGAATGAGTACAATCTAAATCCCTTAACGAGGATCCATTGGAGGGCAAGTCTGGTGCCAGCAGCCGCGGTAATTCCAGCTCCAATAGCGTATATTTAAGTTGTTGCAGTTAAAAAGCTCGTAGTTGGATTTCGGGTGGGGTGGTGCGGTCCGCCTCTGGTGTGCACTGCTCTGCTCCACCTTCCTGCCGGGGACGGGCTCCTGGGCTTCACTGTCTGGGACTCGGAGTCGGCGAGGTTACTTTGAGTAAATTAGAGTGTTCAAAGCAGGCCTACGCTCTGAATACATTAGCATGGAATAACACGATAGGACTCTGGCCTATCTGTTGGTCTGTGGGACCGGAGTAATGATTAAGAGGGGTAGTCGGGGGCATTCGTATTCCGTTGTCAGAGGTGAAATTCTTGGATTTACGGAAGACGAACATCTGCGAAAGCATTTGCCAAGGATACTTTCATTGATCAAGAACGAAAGTTGGGGGCTCGAAGACGATTAGATACCGTCGTAGTCTCAACCATAAACGATGCCGACTAGGGATTGGCAGATGTTCTTTTGATGACTCTGCCAGCACCTTATGAGAAATCAAAGTTTTTGGGTTCCGGGGGGAGTATGGTCGCAAGGCTGAAACTTAAAGGAATTGACGGAAGGGCACCACCAGGCGTGGAGCCTGCGGCTTAATTTGACTCAACACGGGGAAACTTACCAGGTCCAGACACGGGAAGGATTGACAGATTGAGAGCTCTTTCTTGATTCTGTGGGTGGTGGTGCATGGCCGTTCTTAGTTGGTGGGTTGCCTTGTCAGGTTGATTCCGGTAACGAACGAGACCTCAGCCTGCTAAATAGTCAGCATCGCACCTGCGGTGCGCCGACTTCTTAGAGGGACTATTGGCGTTTAGCCAATGGAAGTATGAGGCGATAACAGGTCTGTGATGCCCTTAGATGTTCTGGGCCGCACGCGCGCTACACTGACGCGACCAACGAGCCTATCCTTGGCCGAGAGGCCCGGGTAATCTTGTAAACCGCGTCGTGATGGGGATAGATTATTGCAATTATTAGTCTTCAACGAGGAATGCCTAGTAAGCGCGAGTCATCAGCTCGCGTTGATTACGTCCCTGCCCTTTGTACACACCGCCCGTCGCTCCTACCGATTGGGTGTGCTGGTGAAGTGTTCGGATTGAGCTTGGCTGGGGCAACCTGGCCTTGCTTGAGAAGTTCATTAAACCCTCCCACCTA

>AB701552.1 NIES-2237

AATGGCTCATTATATCAGTTATAGTTTATTTGATGGTACCTACTACTCGGATAACCGTAGTAATTCTAGAGCTAATACGTGCGCACAACCCGACTTCTGGAAGGGTCGTATTTATTAGATAAAAGGCCAGCCGGGCTCTGCCCGACCTGCGGTGAATCATGATAACTTCACGAATCGTATGGGCTCGTCCCGACGATGTTTCATTCAAATTTCTGCCCTATCAACTTTCGATGGTAGGATAGAGGCCTACCATGGTGGTAACGGGTGACGGAGGATTAGGGTTCGATTCCGGAGAGGGAGCCTGAGAGATGGCTACCACATCCAAGGAAGGCAGCAGGCGCGCAAATTACCCAATCCCGACACGGGGAGGTAGTGACAATAAATAACAATACCGGGCGCTTCGCGTCTGGTAATTGGAATGAGTACAATCTAAATCCCTTAACGAGGATCCATTGGAGGGCAAGTCTGGTGCCAGCAGCCGCGGTAATTCCAGCTCCAATAGCGTATATTTAAGTTGTTGCAGTTAAAAAGCTCGTAGTTGGATTTCGGGTGGGGTGGTGCGGTCCGCCTCTGGTGTGCACTGCTCTGCTCCACCTTCCTGCCGGGGACGGGCTCCTGGGCTTCACTGTCTGGGACTCGGAGTCGGCGAGGTTACTTTGAGTAAATTAGAGTGTTCAAAGCAGGCCTACGCTCTGAATACATTAGCATGGAATAACACGATAGGACTCTGGCCTATCTGTTGGTCTGTGGGACCGGAGTAATGATTAAGAGGGGTAGTCGGGGGCATTCGTATTCCGTTGTCAGAGGTGAAATTCTTGGATTTACGGAAGACGAACATCTGCGAAAGCATTTGCCAAGGATACTTTCATTGATCAAGAACGAAAGTTGGGGGCTCGAAGACGATTAGATACCGTCGTAGTCTCAACCATAAACGATGCCGACTAGGGATTGGCAGATGTTCTTTTGATGACTCTGCCAGCACCTTATGAGAAATCAAAGTTTTTGGGTTCCGGGGGGAGTATGGTCGCAAGGCTGAAACTTAAAGGAATTGACGGAAGGGCACCACCAGGCGTGGAGCCTGCGGCTTAATTTGACTCAACACGGGGAAACTTACCAGGTCCAGACACGGGAAGGATTGACAGATTGAGAGCTCTTTCTTGATTCTGTGGGTGGTGGTGCATGGCCGTTCTTAGTTGGTGGGTTGCCTTGTCAGGTTGATTCCGGTAACGAACGAGACCTCAGCCTGCTAAATAGTCAGCATCGCACCTGCGGTGCGCCGACTTCTTAGAGGGACTATTGGCGTTTAGCCAATGGAAGTATGAGGCGATAACAGGTCTGTGATGCCCTTAGATGTTCTGGGCCGCACGCGCGCTACACTGACGCGACCAACGAGCCTATCCTTGGCCGAGAGGCCCGGGTAATCTTGTAAACCGCGTCGTGATGGGGATAGATTATTGCAATTATTAGTCTTCAACGAGGAATGCCTAGTAAGCGCGAGTCATCAGCTCGCGTTGATTACGTCCCTGCCCTTTGTACACACCGCCCGTCGCTCCTACCGATTGGGTGTGCTGGTGAAGTGTTCGGATTGAGCTTGGCTGGGGCAACCTGGCCTTGCTTGAGAAGTTCATTAAACCCTCCCACCTA

>AB753040.1 PS-2708

ATATCAGTTATAGTTTATTTGATGGTACCTACTACTCGGATAACCGTAGTAATTCTAGAGCTAATACGTGCGCACAACCCGACTTCTGGAAGGGTCGTATTTATTAGATAAAAGGCCAGCCGGGCTCTGCCCGACCTGCGGTGAATCATGATAACTTCACGAATCGTATGGGCTCGTCCCGACGATGTTTCATTCAAATTTCTGCCCTATCAACTTTCGATGGTAGGATAGAGGCCTACCATGGTGGTAACGGGTGACGGAGGATTAGGGTTCGATTCCGGAGAGGGAGCCTGAGAGATGGCTACCACATCCAAGGAAGGCAGCAGGCGCGCAAATTACCCAATCCCGACACGGGGAGGTAGTGACAATAAATAACAATACCGGGCGCTTCGCGTCTGGTAATTGGAATGAGTACAATCTAAATCCCTTAACGAGGATCCATTGGAGGGCAAGTCTGGTGCCAGCAGCCGCGGTAATTCCAGCTCCAATAGCGTATATTTAAGTTGTTGCAGTTAAAAAGCTCGTAGTTGGATTTCGGGTGGGGTGGTGCGGTCCGCCTCTGGTGTGCACTGCTCTGCTCCACCTTCCTGCCGGGGACGGGCTCCTGGGCTTCACTGTCTGGGACTCGGAGTCGGCGAGGTTACTTTGAGTAAATTAGAGTGTTCAAAGCAGGCCTACGCTCTGAATACATTAGCATGGAATAACACGATAGGACTCTGGCCTATCTGTTGGTCTGTGGGACCGGAGTAATGATTAAGAGGGGTAGTCGGGGGCATTCGTATTCCGTTGTCAGAGGTGAAATTCTTGGATTTACGGAAGACGAACATCTGCGAAAGCATTTGCCAAGGATACTTTCATTGATCAAGAACGAAAGTTGGGGGCTCGAAGACGATTAGATACCGTCGTAGTCTCAACCATAAACGATGCCGACTAGGGATTGGCAGATGTTCTTTTGATGACTCTGCCAGCACCTTATGAGAAATCAAAGTTTTTGGGTTCCGGGGGGAGTATGGTCGCAAGGCTGAAACTTAAAGGAATTGACGGAAGGGCACCACCAGGCGTGGAGCCTGCGGCTTAATTTGACTCAACACGGGGAAACTTACCAGGTCCAGACACGGGAAGGATTGACAGATTGAGAGCTCTTTCTTGATTCTGTGGGTGGTGGTGCATGGCCGTTCTTAGTTGGTGGGTTGCCTTGTCAGGTTGATTCCGGTAACGAACGAGACCTCAGCCTGCTAAATAGTCAGCATCGCACCTGCGGTGCGCCGACTTCTTAGAGGGACTATTGGCGTTTAGCCAATGGAAGTATGAGGCGATAACAGGTCTGTGATGCCCTTAGATGTTCTGGGCCGCACGCGCGCTACACTGACGCGACCAACGAGCCTATCCTTGGCCGAGAGGCCCGGGTAATCTTGTAAACCGCGTCGTGATGGGGATAGATTATTGCAATTATTAGTCTTCAACGAGGAATGCCTAGTAAGCGCGAGTCATCAGCTCGCGTTGATTACGTCCCTGCCCTTTGTACACACCGCCCGTCGCTCCTACCGATTGGGTGTGCTGGTGAAGTGTTCGGATTGAGCTTGGCTGGGGCAACCTGGCCTTGCTTGAGAAGTTCATTAAACCCTCCCACTA

>AB701553.1 NIES-2238

GAATGGCTCATTATATCAGTTATAGTTTATTTGATGGTACCTACTACTCGGATAACCGTAGTAATTCTAGAGCTAATACGTGCGCACAACCCGACTTCTGGAAGGGTCGTATTTATTAGATAAAAGGCCAGCCGGGCTCTGCCCGACCTGCGGTGAATCATGATAACTTCACGAATCGTATGGGCTCGTCCCGACGATGTTTCATTCAAATTTCTGCCCTATCAACTTTCGATGGTAGGATAGAGGCCTACCATGGTGGTAACGGGTGACGGAGGATTAGGGTTCGATTCCGGAGAGGGAGCCTGAGAGATGGCTACCACATCCAAGGAAGGCAGCAGGCGCGCAAATTACCCAATCCCGACACGGGGAGGTAGTGACAATAAATAACAATACCGGGCGCTTCGCGTCTGGTAATTGGAATGAGTACAATCTAAATCCCTTAACGAGGATCCATTGGAGGGCAAGTCTGGTGCCAGCAGCCGCGGTAATTCCAGCTCCAATAGCGTATATTTAAGTTGTTGCAGTTAAAAAGCTCGTAGTTGGATTTCGGGTGGGGTGGTGCGGTCCGCCTCTGGTGTGCACTGCTCTGCTCCACCTTCCTGCCGGGGACGGGCTCCTGGGCTTCACTGTCTGGGACTCGGAGTCGGCGAGGTTACTTTGAGTAAATTAGAGTGTTCAAAGCAGGCCTACGCTCTGAATACATTAGCATGGAATAACACGATAGGACTCTGGCCTATCTGTTGGTCTGTGGGACCGGAGTAATGATTAAGAGGGGTAGTCGGGGGCATTCGTATTCCGTTGTCAGAGGTGAAATTCTTGGATTTACGGAAGACGAACATCTGCGAAAGCATTTGCCAAGGATACTTTCATTGATCAAGAACGAAAGTTGGGGGCTCGAAGACGATTAGATACCGTCGTAGTCTCAACCATAAACGATGCCGACTAGGGATTGGCAGATGTTCTTTTGATGACTCTGCCAGCACCTTATGAGAAATCAAAGTTTTTGGGTTCCGGGGGGAGTATGGTCGCAAGGCTGAAACTTAAAGGAATTGACGGAAGGGCACCACCAGGCGTGGAGCCTGCGGCTTAATTTGACTCAACACGGGGAAACTTACCAGGTCCAGACACGGGAAGGATTGACAGATTGAGAGCTCTTTCTTGATTCTGTGGGTGGTGGTGCATGGCCGTTCTTAGTTGGTGGGTTGCCTTGTCAGGTTGATTCCGGTAACGAACGAGACCTCAGCCTGCTAAATAGTCAGCATCGCACCTGCGGTGCGCCGACTTCTTAGAGGGACTATTGGCGTTTAGCCAATGGAAGTATGAGGCGATAACAGGTCTGTGATGCCCTTAGATGTTCTGGGCCGCACGCGCGCTACACTGACGCGACCAACGAGCCTATCCTTGGCCGAGAGGCCCGGGTAATCTTGTAAACCGCGTCGTGATGGGGATAGATTATTGCAATTATTAGTCTTCAACGAGGAATGCCTAGTAAGCGCGAGTCATCAGCTCGCGTTGATTACGTCCCTGCCCTTTGTACACACCGCCCGTCGCTCCTACCGATTGGGTGTGCTGGTGAAGTGTTCGGATTGAGCTTGGCTGGGGCAACCTGGCCTTGCTTGAGAAGTTCATTAAACCCTCCCACCTAGAGGAA

>AB701554.1 NIES-2239

ATATCAGTTATAGTTTATTTGATGGTACCTACTACTCGGATAACCGTAGTAATTCTAGAGCTAATACGTGCGCACAACCCGACTTCTGGAAGGGTCGTATTTATTAGATAAAAGGCCAGCCGGGCTCTGCCCGACCTGCGGTGAATCATGATAACTTCACGAATCGTATGGGCTCGTCCCGACGATGTTTCATTCAAATTTCTGCCCTATCAACTTTCGATGGTAGGATAGAGGCCTACCATGGTGGTAACGGGTGACGGAGGATTAGGGTTCGATTCCGGAGAGGGAGCCTGAGAGATGGCTACCACATCCAAGGAAGGCAGCAGGCGCGCAAATTACCCAATCCCGACACGGGGAGGTAGTGACAATAAATAACAATACCGGGCGCTTCGCGTCTGGTAATTGGAATGAGTACAATCTAAATCCCTTAACGAGGATCCATTGGAGGGCAAGTCTGGTGCCAGCAGCCGCGGTAATTCCAGCTCCAATAGCGTATATTTAAGTTGTTGCAGTTAAAAAGCTCGTAGTTGGATTTCGGGTGGGGTGGTGCGGTCCGCCTCTGGTGTGCACTGCTCTGCTCCACCTTCCTGCCGGGGACGGGCTCCTGGGCTTCACTGTCTGGGACTCGGAGTCGGCGAGGTTACTTTGAGTAAATTAGAGTGTTCAAAGCAGGCCTACGCTCTGAATACATTAGCATGGAATAACACGATAGGACTCTGGCCTATCTGTTGGTCTGTGGGACCGGAGTAATGATTAAGAGGGGTAGTCGGGGGCATTCGTATTCCGTTGTCAGAGGTGAAATTCTTGGATTTACGGAAGACGAACATCTGCGAAAGCATTTGCCAAGGATACTTTCATTGATCAAGAACGAAAGTTGGGGGCTCGAAGACGATTAGATACCGTCGTAGTCTCAACCATAAACGATGCCGACTAGGGATTGGCAGATGTTCTTTTGATGACTCTGCCAGCACCTTATGAGAAATCAAAGTTTTTGGGTTCCGGGGGGAGTATGGTCGCAAGGCTGAAACTTAAAGGAATTGACGGAAGGGCACCACCAGGCGTGGAGCCTGCGGCTTAATTTGACTCAACACGGGGAAACTTACCAGGTCCAGACACGGGAAGGATTGACAGATTGAGAGCTCTTTCTTGATTCTGTGGGTGGTGGTGCATGGCCGTTCTTAGTTGGTGGGTTGCCTTGTCAGGTTGATTCCGGTAACGAACGAGACCTCAGCCTGCTAAATAGTCAGCATCGCACCTGCGGTGCGCCGACTTCTTAGAGGGACTATTGGCGTTTAGCCAATGGAAGTATGAGGCGATAACAGGTCTGTGATGCCCTTAGATGTTCTGGGCCGCACGCGCGCTACACTGACGCGACCAACGAGCCTATCCTTGGCCGAGAGGCCCGGGTAATCTTGTAAACCGCGTCGTGATGGGGATAGATTATTGCAATTATTAGTCTTCAACGAGGAATGCCTAGTAAGCGCGAGTCATCAGCTCGCGTTGATTACGTCCCTGCCCTTTGTACACACCGCCCGTCGCTCCTACCGATTGGGTGTGCTGGTGAAGTGTTCGGATTGAGCTTGGCTGGGGCAACCTGGCCTTGCTTGAGAAGT

>AB701550.1 NIES-2235

CCTTGCGAATGGCTCATTATATCAGTTATAGTTTATTTGATGGTACCTACTACTCGGATAACCGTAGTAATTCTAGAGCTAATACGTGCGCACAACCCGACTTCTGGAAGGGTCGTATTTATTAGATAAAAGGCCAGCCGGGCTCTGCCCGACCTGCGGTGAATCATGATAACTTCACGAATCGTATGGGCTCGTCCCGACGATGTTTCATTCAAATTTCTGCCCTATCAACTTTCGATGGTAGGATAGAGGCCTACCATGGTGGTAACGGGTGACGGAGGATTAGGGTTCGATTCCGGAGAGGGAGCCTGAGAGATGGCTACCACATCCAAGGAAGGCAGCAGGCGCGCAAATTACCCAATCCCGACACGGGGAGGTAGTGACAATAAATAACAATACCGGGCGCTTCGCGTCTGGTAATTGGAATGAGTACAATCTAAATCCCTTAACGAGGATCCATTGGAGGGCAAGTCTGGTGCCAGCAGCCGCGGTAATTCCAGCTCCAATAGCGTATATTTAAGTTGTTGCAGTTAAAAAGCTCGTAGTTGGATTTCGGGTGGGGTGGTGCGGTCCGCCTCTGGTGTGCACTGCTCTGCTCCACCTTCCTGCCGGGGACGGGCTCCTGGGCTTCACTGTCTGGGACTCGGAGTCGGCGAGGTTACTTTGAGTAAATTAGAGTGTTCAAAGCAGGCCTACGCTCTGAATACATTAGCATGGAATAACACGATAGGACTCTGGCCTATCTGTTGGTCTGTGGGACCGGAGTAATGATTAAGAGGGGTAGTCGGGGGCATTCGTATTCCGTTGTCAGAGGTGAAATTCTTGGATTTACGGAAGACGAACATCTGCGAAAGCATTTGCCAAGGATACTTTCATTGATCAAGAACGAAAGTTGGGGGCTCGAAGACGATTAGATACCGTCGTAGTCTCAACCATAAACGATGCCGACTAGGGATTGGCAGATGTTCTTTTGATGACTCTGCCAGCACCTTATGAGAAATCAAAGTTTTTGGGTTCCGGGGGGAGTATGGTCGCAAGGCTGAAACTTAAAGGAATTGACGGAAGGGCACCACCAGGCGTGGAGCCTGCGGCTTAATTTGACTCAACACGGGGAAACTTACCAGGTCCAGACACGGGAAGGATTGACAGATTGAGAGCTCTTTCTTGATTCTGTGGGTGGTGGTGCATGGCCGTTCTTAGTTGGTGGGTTGCCTTGTCAGGTTGATTCCGGTAACGAACGAGACCTCAGCCTGCTAAATAGTCAGCATCGCACCTGCGGTGCGCCGACTTCTTAGAGGGACTATTGGCGTTTAGCCAATGGAAGTATGAGGCGATAACAGGTCTGTGATGCCCTTAGATGTTCTGGGCCGCACGCGCGCTACACTGACGCGACCAACGAGCCTATCCTTGGCCGAGAGGCCCGGGTAATCTTGTAAACCGCGTCGTGATGGGGATAGATTATTGCAATTATTAGTCTTCAACGAGGAATGCCTAGTAAGCGCGAGTCATCAGCTCGCGTTGATTACGTCCCTGCCCTTTGTACACACCGCCCGTCGCTCCTACCGATTGGGTGTGCTGGTGAAGTGTTCGGATTGAGCTTGGCTGGGGCAACCTGGCCTTGCTTGAGAAGTCATTAAACCCTCCCACCTAGAGGAA

>KC149968.1 GTD4

CACCTGGTTGATCCTGCCAGTAGTCATATGCTTGTCTCAAAGATTAAGCCATGCATGTCTAAGTATAAACTGCTTATACTGTGAAACTGCGAATGGCTCATTAAATCAGTTATAGTTTATTTGATGGTACCTACTACTCGGATAACCGTAGTAATTCTAGAGCTAATACGTGCGCACAACCCGACTTCTGGAAGGGTCGTATTTATTAGATAAAAGGCCAGCCGGGCTCTGCCCGACCTGCGGTGAATCATGATAACTTCACGAATCGTATGGCCTTGTGCCGACGATGTTTCATTCAAATTTCTGCCCTATCAACTTTCGATGGTAGGATAGAGGCCTACCATGGTGGTAACGGGTGACGGAGGATTAGGGTTCGATTCCGGAGAGGGAGCCTGAGAGATGGCTACCACATCCAAGGAAGGCAGCAGGCGCGCAAATTACCCAATCCCGACACGGGGAGGTAGTGACAATAAATAACAATACCGGGCGCTTAGCGTCTGGTAATTGGAATGAGTACAATCTAAATCCCTTAACGAGGATCCATTGGAGGGCAAGTCTGGTGCCAGCAGCCGCGGTAATTCCAGCTCCAATAGCGTATATTTAAGTTGTTGCAGTTAAAAAGCTCGTAGTTGGATTTCGGGTGGGGTGGTGCGGTCCGCCTCTGGTGTGCACTGCTCCGCTCCACCTTCCTGCCGGGGACGGGCTCCTGGGCTTAACTGTCCGGGACTCGGAGTCGGCGAGGTTACTTTGAGTAAATTAGAGTGTTCAAAGCAGGCCTACGCTCTGAATACATTAGCATGGAATAACACGATAGGACTCTGGCCTATCTGTTGGTCTGTGGGACCGGAGTAATGATTAAGAGGGGTAGTCGGGGGCATTCGTATTCCGTTGTCAGAGGTGAAATTCTTGGATTTACGGAAGACGAACATCTGCGAAAGCATTTGCCAAGGATACTTTCATTGATCAAGAACGAAAGTTGGGGGCTCGAAGACGATTAGATACCGTCGTAGTCTCAACCATAAACGATGCCGACTAGGGATTGGCAGATGTTCTTTTGATGACTCTGCCAGCACCTTATGAGAAATCAAAGTTTTTGGGTTCCGGGGGGAGTATGGTCGCAAGGCTGAAACTTAAAGGAATTGACGGAAGGGCACCACCAGGCGTGGAGCCTGCGGCTTAATTTGACTCAACACGGGGAAACTTACCAGGTCCAGACACGGGAAGGATTGACAGATTGAGAGCTCTTTCTTGATTCTGTGGGTGGTGGTGCATGGCCGTTCTTAGTTGGTGGGTTGCCTTGTCAGGTTGATTCCGGTAACGAACGAGACCTCAGCCTGCTAAATAGTCAGCATCGCACCTGCGGTGCGCCGACTTCTTAGAGGGACTATTGGCGTTTAGCCAATGGAAGTATGAGGCGATAACAGGTCTGTGATGCCCTTAGATGTTCTGGGCCGCACGCGCGCTACACTGACGCGATCAACGAGCCTATCCTTGGCCGAGAGGCCCGGGTAATCTTGTAAACCGCGTCGTGATGGGGATAGACTATTGCAATTATTAGTCTTCAACGAGGAATGCCTAGTAAGCGCGAGTCATCAGCTCGCGTTGATTACGTCCCTGCCCTTTGTACACACCGCCCGTCGCTCCTACCGATTGAATGTGCTGGTGAAGTGTTCGGATTGGCCTTGATTGGGGCAACTCGGTCTTGGCTGAAAAGTTCATTAAACCCTCCCATTTAGAGGAAGGAGAAGTCGTAACAAGGT

>EU925397.1 CC-124

CGATTCGCCCTTCGGAGAGGGAGCATGAGAGATGGCTACCACATCCAAGGAAGGCAGCAGGCGCGCAAATTACCCAATCCCGACACGGGGAGGTAGTGACAATAAATAACAATACCGGGCGCTTCGCGTCTGGTAATTGGAATGAGTACAATCTAAATCCCTTAACGAGGATCCATTGGAGGGCAAGTCTGGTGCCAGCAGCCGCGGTAATTCCAGCTCCAATAGCGTATATTTAAGTTGTTGCAGTTAAAAAGCTCGTAGTTGGATTTCGGGTGGGGTGGTGCGGTCCGCCTCTGGTGTGCACTGCTCTGCTCCACCTTCCTGCCGGGGACGGGCTCCTGGGCTTCACTGTCTGGGACTCGGAGTCGGCGAGGTTACTTTGAGTAAATTAGAGTGTTCAAAGCAGGCCTACGCTCTGAATACATTAGCATGGAATAACACGATAGGACTCTGGCCTATCTGTTGGTCTGTGGGACCGGAGTAATGATTAAGAGGGGTAGTCGGGGGCATTCGTATTCCGTTGTCAGAGGTGAAATTCTTGGATTTACGGAAGACGAACATCTGCGAAAGCATTTGCCAAGGATACTTTCATTGATCAAGAACGAAAGTTGGGGGCTCGAAGACGATTAGATACCGTCGTAGTCTCAACCATAAACGATGCCGACTAGGGATTGGCAGATGTTCTTTTGATGACTCTGCCAGCACCTTATGAGAAATCAAAGTTTTTGGGTTCCGGGGGGAGTATGGTCGCAAGGCTGAAACTTAAAGGAATTGACGGAAGGGCACCACCAGGCGTGGAGCCTGCGGCTTAATTTGACTCAACACGGGGAAACTTACCAGGTCCAGACACGGGAAGGATTGACAGATTGAGAGCTCTTTCTTGATTCTGTGGGTGGTGGTGCATGGCCGTTCTTAGTTGGTGGGTTGCCTTGTCAGGTTGATTCCGGTAACGAACGAGACCTCAGCCTGCTAAATAGTCAGCATCGCACCTGCGGTGCGCCGACTTCTTAGAGGGACTATTGGCGTTTAGCCAATGGAAGTATGAGGCGATAACAGGTCTGTGATGCCCTTAGATGTTCTGGGCCGCACGCGCGCTACACTGACGCGACCAACGAGCCTATCCTTGGCCGAGAGGCCCGGGTAATCTTGTAAACCGCGTCGTGATGGGGATAGATTATTGCAATTATTAGTCTTCAACGAGGAATGCCTAGTAAGCGCGAGTCATCAGCTCGCGTTGATTACGTCCCTGCCCTTTGTACACACCGCCCGTCGCTCCTACCGATTGGGTGTGCTGGTGAAGTGTTCGGATTGAGCTTGGCTGGGGCAACCTGGCCTTGCTTGAGAAGTTCATTAAACCCTCCCACCTAGAGGAAGGAGAAGTCGTAACAAGGTAAGGGCGAATC

>AY665727.1 CC-1952

CCATGCATGTCTAAGTATAAACTGCTTATACTGTGAAACTGCGAATGGCTCATTAAATCAGTTATAGTTTATTTGATGGTACCTACTACTCGGATAACCGTAGTAATTCTAGAGCTAATACGTGCGCACAACCCGACTTCTGGAAGGGTCGTATTTATTAGATAAAAGGCCAGCCGGGCTCTGCCCGACCTGCGGTGAATCATGATAACTTCACGAATCGTATGGGCTCGTCCCGACGATGTTTCATTCAAATTTCTGCCCTATCAACTTTCGATGGTAGGATAGAGGCCTACCATGGTGGTAACGGGTGACGGAGGATTAGGGTTCGATTCCGGAGAGGGAGCCTGAGAGATGGCTACCACATCCAAGGAAGGCAGCAGGCGCGCAAATTACCCAATCCCGACACGGGGAGGTAGTGACAATAAATAACAATACCGGGCGCTTCGCGTCTGGTAATTGGAATGAGTACAATCTAAATCCCTTAACGAGGATCCATTGGAGGGCAAGTCTGGTGCCAGCAGCCGCGGTAATTCCAGCTCCAATAGCGTATATTTAAGTTGTTGCAGTTAAAAAGCTCGTAGTTGGATTTCGGGTGGGGTGGTGCGGTCCGCCTCTGGTGTGCACTGCTCTGCTCCACCTTCCTGCCGGGGACGGGCTCCTGGGCTTCACTGTCTGGGACTCGGAGTCGGCGAGGTTACTTTGAGTAAATTAGAGTGTTCAAAGCAGGCCTACGCTCTGAATACATTAGCATGGAATAACACGATAGGACTCTGGCCTATCTGTTGGTCTGTGGGACCGGAGTAATGATTAAGAGGGGTAGTCGGGGGCATTCGTATTCCGTTGTCAGAGGTGAAATTCTTGGATTTACGGAAGACGAACATCTGCGAAAGCATTTGCCAAGGATACTTTCATTGATCAAGAACGAAAGTTGGGGGCTCGAAGACGATTAGATACCGTCGTAGTCTCAACCATAAACGATGCCGACTAGGGATTGGCAGATGTTCTTTTGATGACTCTGCCAGCACCTTATGAGAAATCAAAGTTTTTGGGTTCCGGGGGGAGTATGGTCGCAAGGCTGAAACTTAAAGGAATTGACGGAAGGGCACCACCAGGCGTGGAGCCTGCGGCTTAATTTGACTCAACACGGGGAAACTTACCAGGTCCAGACACGGGAAGGATTGACAGATTGAGAGCTCTTTCTTGATTCTGTGGGTGGTGGTGCATGGCCGTTCTTAGTTGGTGGGTTGCCTTGTCAGGTTGATTCCGGTAACGAACGAGACCTCAGCCTGCTAAATAGTCAGCATCGCACCTGCGGTGCGCCGACTTCTTAGAGGGACTATTGGCGTTTAGCCAATGGAAGTATGAGGCGATAACAGGTCTGTGATGCCCTTAGATGTTCTGGGCCGCACGCGCGCTACACTGACGCGACCAACGAGCCTATCCTTGGCCGAGAGGCCCGGGTAATCTTGTAAACCGCGTCGTGATGGGGATAGATTATTGCAATTATTAGTCTTCAACGAGGAATGCCTAGTAAGCGCGAGTCATCAGCTCGCGTTGATTACGTCCCTGCCCTTTGTACACACCGCCCGTCGCTCCTACCGATTGGGTGTGCTGGTGAAGTGTTCGGATTGAGCTTGGCTGGGGCAACCTGGCCTTGCTTGAGAAGTTCATTAAACCCTCCCACCTA

>AY665726.1 CC-1418

ATTAAGCCATGCATGTCTAAGTATAAACTGCTTATACTGTGAAACTGCGAATGGCTCATTAAATCAGTTATAGTTTATTTGATGGTACCTACTACTCGGATAACCGTAGTAATTCTAGAGCTAATACGTGCGCACAACCCGACTTCTGGAAGGGTCGTATTTATTAGATAAAAGGCCAGCCGGGCTCTGCCCGACCTGCGGTGAATCATGATAACTTCACGAATCGTATGGGCTCGTCCCGACGATGTTTCATTCAAATTTCTGCCCTATCAACTTTCGATGGTAGGATAGAGGCCTACCATGGTGGTAACGGGTGACGGAGGATTAGGGTTCGATTCCGGAGAGGGAGCCTGAGAGATGGCTACCACATCCAAGGAAGGCAGCAGGCGCGCAAATTACCCAATCCCGACACGGGGAGGTAGTGACAATAAATAACAATACCGGGCGCTTCGCGTCTGGTAATTGGAATGAGTACAATCTAAATCCCTTAACGAGGATCCATTGGAGGGCAAGTCTGGTGCCAGCAGCCGCGGTAATTCCAGCTCCAATAGCGTATATTTAAGTTGTTGCAGTTAAAAAGCTCGTAGTTGGATTTCGGGTGGGGTGGTGCGGTCCGCCTCTGGTGTGCACTGCTCTGCTCCACCTTCCTGCCGGGGACGGGCTCCTGGGCTTCACTGTCTGGGACTCGGAGTCGGCGAGGTTACTTTGAGTAAATTAGAGTGTTCAAAGCAGGCCTACGCTCTGAATACATTAGCATGGAATAACACGATAGGACTCTGGCCTATCTGTTGGTCTGTGGGACCGGAGTAATGATTAAGAGGGGTAGTCGGGGGCATTCGTATTCCGTTGTCAGAGGTGAAATTCTTGGATTTACGGAAGACGAACATCTGCGAAAGCATTTGCCAAGGATACTTTCATTGATCAAGAACGAAAGTTGGGGGCTCGAAGACGATTAGATACCGTCGTAGTCTCAACCATAAACGATGCCGACTAGGGATTGGCAGATGTTCTTTTGATGACTCTGCCAGCACCTTATGAGAAATCAAAGTTTTTGGGTTCCGGGGGGAGTATGGTCGCAAGGCTGAAACTTAAAGGAATTGACGGAAGGGCACCACCAGGCGTGGAGCCTGCGGCTTAATTTGACTCAACACGGGGAAACTTACCAGGTCCAGACACGGGAAGGATTGACAGATTGAGAGCTCTTTCTTGATTCTGTGGGTGGTGGTGCATGGCCGTTCTTAGTTGGTGGGTTGCCTTGTCAGGTTGATTCCGGTAACGAACGAGACCTCAGCCTGCTAAATAGTCAGCATCGCACCTGCGGTGCGCCGACTTCTTAGAGGGACTATTGGCGTTTAGCCAATGGAAGTATGAGGCGATAACAGGTCTGTGATGCCCTTAGATGTTCTGGGCCGCACGCGCGCTACACTGACGCGACCAACGAGCCTATCCTTGGCCGAGAGGCCCGGGTAATCTTGTAAACCGCGTCGTGATGGGGATAGATTATTGCAATTATTAGTCTTCAACGAGGAATGCCTAGTAAGCGCGAGTCATCAGCTCGCGTTGATTACGTCCCTGCCCTTTGTACACACCGCCCGTCGCTCCTACCGATTGGGTGTGCTGGTGAAGTGTTCGGATTGAGCTTGGCTGGGGCAACCTGGCCTTGCTTGAGAAGTTCATTAAACCCTCCCACCTA
