# Supplementary material for: Overexpression of 18S rRNA methyltransferase CrBUD23 enhances biomass and lutein content in Chlamydomonas reinhardtii
Source: Front Bioeng Biotechnol. 2023 Feb 3;11:1102098. doi: 10.3389/fbioe.2023.1102098 (PMC9935685; doi:10.3389/fbioe.2023.1102098)
Supplement: Supplementary file 10 [file Table4.DOCX]

>ScTrm112

MKFLTTNFLKCSVKACDTSNDNFPLQYDGSKCQLVQDESIEFNPEFLLNIVDRVDWPAVLTVAAELGNNALPPTKPSFPSSIQELTDDDMAILNDLHTLLLQTSIAEGEMKCRNCGHIYYIKNGIPNLLLPPHLV

>HsTRMT112

MKLLTHNLLSSHVRGVGSRGFPLRLQATEVRICPVEFNPNFVARMIPKVEWSAFLEAADNVPKGPVEGYEENEEFLRTMHHLLLEVEVIEGTLQCPESGRMFPISRGIPNMLLSEEETES

>MmTRM112

MKLLTHNLLSSHVRGVGTRGFPLRLQATEVRINPVEFNPEFVARMIPKVEWAALVQAADTLNLAEVPKEPTEGYEHDETFLRKMHHVLLEVDVLEGTLQCPESGRLFPISRGIPNMLLNDEETET

>DrTRM112

MKLLTHNMLTSHVKGVTKGYPLIIKATEVKVNELDFNAQFVSRMIPKLEWPALVQAAEWLGQSQELPDTLIPDYENDEEFLRKVHRVLLEVEVIEGCLQCPESGREFPISKGVPNMLLNEGE

>DmTRM112

MKLSTYNFLTSVAIKGVKVGFPLKLTINKKEVVESEFNPTFVERILPKLDWSAVYGAAQVAELTEDIPAVQPENIVENELLLQKLHHLLFEIDVLEGQLECPETGRVFPISDGIPNMLLNEDEV

>CeTRM112

MKLFVHNFMSSRFLKNVTVGYPLNLVVKQFVEKDIEFDRDNTIVMLDRIQYEALIVAAAAVNQSDRIPREKPEKWDELTDEQLRVFHHLLMNIDVIDGELICPETKTVFPIRDGIPNMLKVDAEK

>BbTrm112

MKLLTHNFLTCKIKGVQNGYPFKIVADKVETVTTDFDSDFLKRIFDRVNYQVLKEAAESLGESEGLPEQATLEALEDEAFQKAYHHALLEVVVKEGALVCPETGRRFIVKKGIPNLLLNEDEIA*

>CrTrm112

MKLLTHNMLSCHIKNVRNGYPFLIEVVKVSEHEADFDPDFLKHIFPRINWPAFLQGAQSLGCREGLPEEAAESMLEDEGFLKRFHHALLEVFLEEGSLVCPETGRKFPVTKGIPNMLLNEDEC*

>CzTrm112

MKLLTHNMLSCHIKGVQNGFPFKIQASKIEEREADYDPDFLRHIFPKIEWKAFLEGARALHCADGLPEEVTDEMLQDDGFLQAFHHALLEVHLEEGALICPETGRHFTVAKGIPNLLLNEDEC*

>CsTrm112

MKLLTHNMLSCHIKGVQNGFPFKIEPVKVEQVDADYDPDFLRHIYPRLEWKALCEAAATMGAPGLPEEVSEEMLQDDDFLRSFHHALLELVLEEGALVCPETGRQFPVHKGVPNLLLNEDEN*

>CsTrm112-like

MALARSLVRAQRQFVRTVFTTTRGSSSVVSKGAYNVTEFDEGMLEFLACPLSKTPLRWDEATSSLICDELGVAYPVNNGMPNLRPADGHVIEAQDRESSTSQQPWGDTDAVPK

>DsTrm112

MKLLTHNMLACHIKGITNNYPLLIEATKIETRDADFNPDFLRHIFPRIQWPAFLQGAETLGCRGSLPDDPVPEGSLEDETFLKQFHHALLEVVLEEGFLICPETQRRFPVCKGIPNLLLNEDEC*

>MpTrm112

MRLLTHNLLASNVKGTTAGFPLKLEVLVKEERSTEFDAAFLLHTLPKLNWSAFRAAAESLGVDKLPSTYPERDELTNEFLRVFHHALLEIEVQEGYLICPETGRRFPVRKGIPNMMLNEDEVD*

>OlTrm112

MRLLAHNMLACNAKGVVNGFPLKIVPKETREVEVDFNAEFLTHMLPKMEWSAFVNAAKEIGLEGLPSEIPDDAASDEEFLRTFHHALLEVHVEEGTLVCPESGRKFPINKGIPNMLLNEDEV*

>VcTrm112

MKLLTHNMLSCHIKGVRSGYPFLIEVVKVSEHEADFDTDFLKHIFPRINWPAFLQGAEAMGCREGLPEAPNESALEDEQFQKAFHHALLEVTLEEGSLICPETGRKFPVSKGIPNMLLNEDEC*

>At4g35905

MVRLNRVLLKDAGNVIDKTLSEILVCPLSKQPLRFCEKTKSLVSDTIGVSFPIKDGIPCLVPKDGKILEEEGDASKA*

>At1g22270

MRLITHNMLSCNIKGVTSGFPLRIEAGNVIEKEVDFNPDFIRHMFAKIEWKALVEGARSMGYAELPEESPDAAVLKSDEPFLKKLHHALLELHLEEGALVCPETGRKFPVNKGIPNMLLHEDEV*

>At1g78190

MRLIVHNMLSCNIKGVVNKFPLRIEAEKVTVKEVDFNPDFLRYMFAKIDWKALVDGARSMEYTELPDNAPDTTTLESDETFLRKFHHALLELHLEEGSLVCPETGRKFSVSKGIPNMLLHEDEV*

>Mt1g050345

MGLLTHNMLSSNIRGVVNGFPLRIEAVKVVEKNVEMNTDFLKNMFEKIDWKAFVEASISMGYTELPKEADSSLLDSDDFLNRFHHALLELHLEEGALVCPETRRRFPVSKGIPHMLLHEDEV*

>Mt2g028730

MRLLTHNMLSSNIKGVVNGFPLRIEAEKVVEKNVEMNGDFLKKMFEKIEWKAFVEASRGMGYTELPEEADSSMLDSNEFLNRFHHALLELHLEEGALVCPETGRRFPVKKGIPNMLLHEDEV*

>Mt5g022760

MVRVSKELMQKAATLVSDFLVCPLSKQPLRYCQESNSLISDAIAVSFPIKNGIPCLVPRDGKILNEEDASKPDNDTNL*

>Os10g30300

MRRTAASLLRHSGIGGGGGGSGGGIPQALADALVCPLSKKPLRYCEDSGSLVSDAVGVSFPIVDGIPYLVPKDGKLLDHDQDKSEDCGAKDSSHRH*

>Os07g43020

MRLLTHNMLASNARGAVTGYPLKLQVVKWSTKEAEPNPEFLRGMLPKIDWPALVAATQALGLPELLPEAPPTDAELSAEGAAADEGSALRRLHRALLEIHIEEGALVCPDTDRCFPISRGVPNMLLHEDEVRN*

>Pp3c10_8220

MRLLTHNMLACNIKGVTKGFPLGIEHTRLETKESELNADFLRHIFPKLDWKAFHEAAQSVGVNNIPDQVEPVMLDDDEFLRKFHHALLEVHLEEGALICPETGRRFPVTKGVPNMLLNEDEV*

>Zm00001d014140

MRRTAALLSAHCGRSIPLALADVLVCPLSKQPLRYCEVNGSLVSDSAGVAFPVLDGIPSLVPKDGKLLDDQEVKSEQESSTRDSSG*

>Zm00001d006904

MRLLTHNMLASNVRGATTGYPLTLEATNWCTKEVELNSDFIRGLLPKIDWRALVAATRAVGLPELLPEEQPPEEEIFADGAADVEGSAIRRIHHALLEVHVQEGSLVCPDTSRCFPINKGIPNMMLHEDEV*
